# Key role of *Desulfobacteraceae* in C/S cycles of marine sediments is based on congeneric catabolic-regulatory networks

**DOI:** 10.1126/sciadv.ads5631

**Published:** 2025-03-07

**Authors:** Lars Wöhlbrand, Marvin Dörries, Roberto Siani, Arturo Medrano-Soto, Vanessa Schnaars, Julian Schumacher, Christina Hilbers, Daniela Thies, Michael Kube, Richard Reinhardt, Michael Schloter, Milton H. Saier, Michael Winklhofer, Ralf Rabus

**Affiliations:** ^1^ Institute for Chemistry and Biology of the Marine Environment (ICBM), School of Mathematics and Science, Carl von Ossietzky Universität Oldenburg, Oldenburg, Germany.; ^2^ Helmholtz Institute for Functional Marine Biodiversity at the Carl von Ossietzky Universität Oldenburg (HIFMB), Oldenburg, Germany.; ^3^ Institute for Comparative Microbiome Analysis (COMI), Department of Environmental Sciences, Helmholtz Zentrum München, Oberschleißheim, Munich, Germany.; ^4^ Chair for Environmental Microbiology, School of Life Sciences, Technical University Munich, Freising, Germany.; ^5^ Department of Molecular Biology, School of Biological Sciences, University of California, San Diego, San Diego, CA, USA.; ^6^ Max Planck Institute for Marine Microbiology, Bremen, Germany.; ^7^ Integrative Infection Biology Crops-Livestocks, Faculty of Agricultural Sciences, University Hohenheim, Stuttgart, Germany.; ^8^ Max Planck Genome Centre Cologne, Cologne, Germany.; ^9^ Institute of Biology and Environmental Sciences (IBU), School of Mathematics and Science, Carl von Ossietzky Universität Oldenburg, Oldenburg, Germany.; ^10^ Research Center Neurosensory Science, School of Mathematics and Science, Carl von Ossietzky Universität Oldenburg, Oldenburg, Germany.

## Abstract

Marine sediments are highly bioactive habitats, where sulfate-reducing bacteria contribute substantially to seabed carbon cycling by oxidizing ~77 Tmol C_org_ year^−1^. This remarkable activity is largely attributable to the deltaproteobacterial family *Desulfobacteraceae* of complete oxidizers (to CO_2_), which our biogeography focused meta-analysis verified as cosmopolitan. However, the catabolic/regulatory networks underlying this ecophysiological feat at the thermodynamic limit are essentially unknown. Integrating cultivation-based (80 conditions) proteogenomics of six representative *Desulfobacteraceae* spp., we identify molecular commonalities explaining the family’s environmental relevance and success. *Desulfobacteraceae* genomes are specifically enriched in substrate uptake, degradation capacities, and regulatory functions including fine-tuned sulfate uptake. Conserved gene arrangements and shared regulatory patterns translate into strikingly similar (sub-)proteome profiles. From 319 proteins, we constructed a meta-network for catabolizing 35 substrates. Therefrom, we defined a *Desulfobacteraceae* characteristic gene subset, which we found prevalent in metagenomes of organic-rich, marine sediments. These genes are promising targets to advance our mechanistic understanding of *Desulfobacteraceae*-driven biogeochemical processes in marine sediments and beyond.

## Teaser

Comparative proteogenomics reveal ecophysiological feats of *Desulfobacteraceae* for carbon and sulfur cycling in marine sediments.

## INTRODUCTION

Sulfate-reducing bacteria (SRB), such as *Desulfobacteraceae*, couple the oxidation of organic carbon to the reduction of sulfate to sulfide (dissimilatory sulfate reduction), thereby linking the carbon and sulfur cycles. This process is particularly important in global marine environments due to very high sulfate concentrations in the oceans [for overview, see (*1*, *2*)]. Here, continental margins, coastal ranges, and shelf sediments stand out by their high input of organic matter, and more than 50% of their mineralization is achieved in the upper sediment layers, coupled to sulfate reduction (*3*, *4*). Furthermore, organic carbon richness of upwelling regions generates oxygen minimum zones in the waterbody where SRB are involved in carbon turnover and a cryptic sulfur cycle (*5*). Globally, of the total carbon flux reaching the ocean floor, 12 to 29% are oxidized via sulfate reduction, as estimated from steady-state sulfate profiles (*6*). However, considering sulfate-reduction rates calculated from ^35^S radiotracer measurements as well as re-oxidation and cryptic sulfur cycle, this share is markedly higher, accounting for an estimated 77 Tmol C_org_ year^−1^ (*7*). These high mineralization rates may only be achieved by SRB capable of complete oxidation to CO_2_ (*8*, *9*). However, the well-studied family *Desulfovibrionaceae* cannot achieve this turnover because these organisms only incompletely oxidize organic substrates to acetate. The discovery of the family *Desulfobacteraceae*, encompassing completely oxidizing SRB (*10*–*12*), solved this biogeochemical paradox. Members of this family use a large variety of organic substrates, ranging from small molecules (e.g., fermentation end products) to long-chain fatty acids and aromatic compounds, including recalcitrant hydrocarbon compounds, such as *n*-alkanes, alkylbenzenes, and -phenols (*1*, *13*, *14*). The degradation routes of these different organic carbon substrates ultimately converge at the level of acetyl–coenzyme A (CoA) that is oxidized to CO_2_ via the Wood-Ljungdahl pathway (WLP) in most cases (*1*). In agreement with their proposed environmental role, *Desulfobacteraceae* members, particularly those of the *Desulfosarcina*/*Desulfococcus* cluster, were shown to dominate the SRB communities in marine shelf sediments (e.g., *15*–*17*).

Generally, SRB thrive at the thermodynamic limit of life, due to the very low redox potential of the sulfate/sulfide redox pair (−228 mV), allowing the generation of only ~10% of the energy obtained as compared to aerobic heterotrophs applying the oxygen/water pair (+818 mV) (*18*). To cope with this challenge, SRB evolved a number of specialized enzymes/complexes that harness biochemically intriguing mechanisms to facilitate endergonic reactions without spending adenosine 5′-triphosphate (ATP), e.g., reduction of sulfite to sulfide via a DsrC trisulfide (*19*), an electrogenic redox loop involving QrcABCD in sulfate reduction (*20*), or ATP-independent reductive dearomatization applying electron bifurcation (*21*). However, this research focus on single remarkable enzymatic mechanisms needs to be complemented by global analyses of the involved catabolic networks and their regulatory modulations to explain the complexity involved in the environmental success and functions of SRB, both in general and for the *Desulfobacteraceae* in particular.

To achieve a holistic understanding of the role of *Desulfobacteraceae* in the marine carbon/sulfur cycles, we first conducted a meta-analysis (literature based) to capture globally the biogeography of this family. Then, we conducted comparative proteogenomic analyses across six members of the *Desulfobacteraceae*, which we selected to cover the versatility, phylogeny, and lifestyles of this family. Among them, *Desulfosarcina variabilis* 3be13 is a particularly versatile, cultivated representative of the *Desulfosarcina/Desulfococcus* cluster, which is why we sequenced its genome and determined the proteomes of 29 substrate conditions. Overall, a total of 80 substrate conditions across the six studied strains allowed us (i) to gain unprecedented insights into commonalities of consistently formed (constitutive) versus substrate-specifically formed (regulated) subsets of the proteome (subproteomes), the formed transportome (entirety of transmembrane transport systems), and the regulation of sulfate uptake and (ii) to construct a catabolic meta-network (synthesis of all reactions/proteins involved in substrate degradation and respiratory energy conservation). Last, genes encoding various enzymes of key pathways in the meta-network were recognized as widespread across 43 *Desulfobacteraceae* reference genomes and also detectable in available metagenomes from geographically far apart and geochemically distinct marine sediments.

## RESULTS

### Biogeography of *Desulfobacteraceae*

**Fig. 1.**
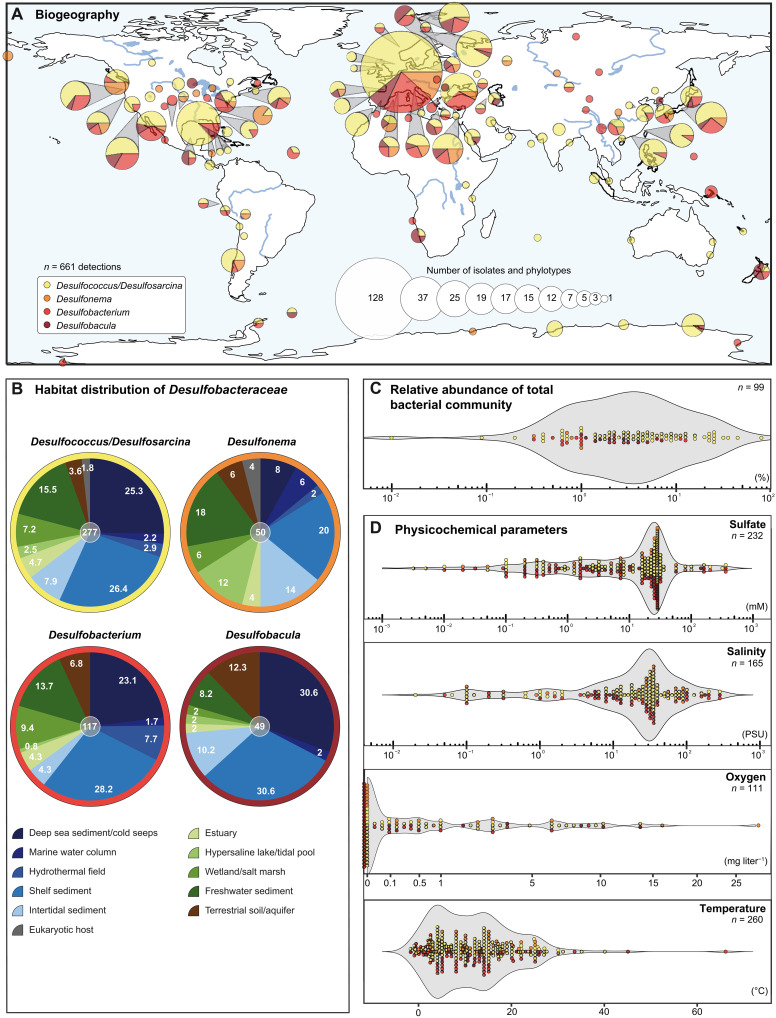
Global occurrence, physicochemical habitat parameters, and habitat prevalence of *Desulfobacteraceae* key genera. (**A**) Global distribution of sites, where the occurrence of isolates and/or phylotypes affiliating with the genera *Desulfococcus*/*Desulfosarcina* (yellow), *Desulfonema* (orange), *Desulfobacterium* (red), or *Desulfobacula* (dark red) are reported (512 sites with a total of 661 occurrences across the genera). Densely spaced sites are aggregated into clusters and represented as circles. The circled areas indicate the number of occurrences/sites in a cluster, and the pie sector area indicates the abundance of a given genus in a cluster. Coloring of the genera is consistently used in (B) to (D). (**B**) Relative frequency of isolate/phylotype detection in different habitats is given for the selected genera. The total numbers of available data are indicated in the central gray circle. (**C**) Relative abundances of the selected genera within the total bacterial community. (**D**) Distribution of physicochemical parameters determined for the sites of detection: sulfate concentration, salinity, oxygen, and temperature. The numbers of available data are indicated. Underlying literature-based data are compiled in tables S1 to S5.

We reviewed the literature to assess the global distribution, environmental context, and habitat conditions of the family *Desulfobacteraceae*, exemplified for the genera *Desulfosarcina*, *Desulfococcus*, *Desulfobacula*, *Desulfobacterium*, and *Desulfonema*. More than 300 publications were analyzed (tables S1 to S5), resulting in the assignment of isolates or phylotypes to 512 geographical locations (Fig. 1A). Documented habitats cover the entire globe, from the Arctic to Antarctic and all longitudes. The higher occurrence in North America (155 sites) and Europe (256 sites) reflects apparent research foci in these areas rather than the absence of these phyla in other regions, e.g., 96 sites in Asia (for details, see also fig. S1 and tables S1 to S5). At most sites the *Desulfosarcina*/*Desulfococcus* group dominates the SRB community (406 isolates and phylotypes versus 56 to 140 for the other genera), agreeing with previous findings [e.g., (*15*, *22*)]. The marine realm represents the most important habitat (54.0 to 77.5%) of the selected five genera, dominated by deep sea and shelf sediments, although the presence in non-marine sites (mainly freshwater sediments) underscores their general importance in global elemental cycling (Fig. 1B). The high occurrence of *Desulfonema* spp. in brackish/freshwater habitats points toward the occupation of a special ecological niche. *Desulfobacteraceae* members account for 1 to 10% of the respective total bacterial communities (Fig. 1C), although substantially higher numbers have been reported [e.g., (*23*, *24*)]. The predominant occurrence of *Desulfobacteraceae* in the marine realm is reflected by the matching profile maxima of physicochemical parameters: high sulfate concentration and moderate-to-typical sea water salinity, mostly anoxia or suboxic conditions, and cold-to-moderate temperatures (Fig. 1D). Moreover, the broad dispersion of the physicochemical parameters suggests that *Desulfobacteraceae* members can cope with a wide variety of habitat conditions (Fig. 1B).

### *Desulfobacteraceae*-shaping genomic traits

**Fig. 2.**
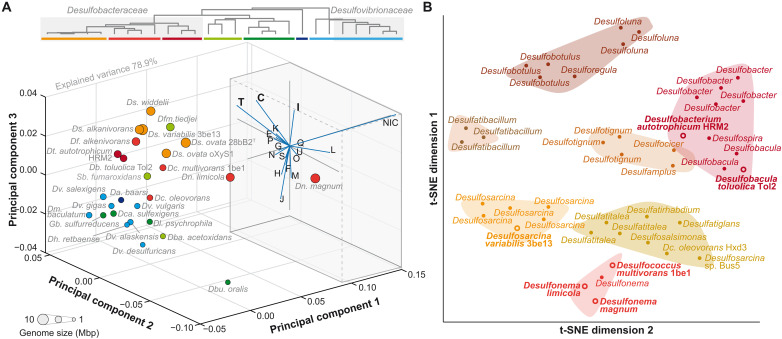
*Desulfobacteraceae* genomes stand out among SRB for their catabolic/regulatory potential while showing genus-specific features. (**A**) 3D PCA based on the genomic share of CDSs allocated to the cluster of orthologous groups (COGs) for completely sequenced *Desulfobacteraceae* genomes and selected other SRB. The color code is based on the indicated 16*S* rRNA-based phylogeny (top) of the selected SRB (see also fig. S3). The inset shows the underlying PCA loadings plot; letters indicate COG categories; NIC, not in COG. (**B**) Comparison of 43 reference *Desulfobacteraceae* genomes applying t-SNE. Genera and clusters are indicated. Genus abbreviations: Da, *Desulfarculus*; Db, *Desulfobacula*; Dba, *Desulfobacca*; Dbu, *Desulfobulbus*; Dc, *Desulfococcus*; Dca, *Desulfocapsa*; Df, *Desulfatibacillum*; Dfm, *Desulfomonile*; Dh, *Desulfohalobium*; Dl, *Desulfotalea*; Dm, *Desulfomicrobium*; Dn, *Desulfonema*; Ds, *Desulfosarcina*; Dt, *Desulfobacterium*; Dv, *Desulfovibrio*; Sb, *Synthrophobacter*.

To determine genome-imprinted traits underlying the environmental success of *Desulfobacteraceae*, we conducted a comparative genome analysis. For this purpose, we selected 27 high-quality genomes of experimentally well-studied SRB, comprising 12 *Desulfobacteraceae*, 5 *Desulfovibrionaceae*, and 10 others (tables S6 and S7). An obvious, prominent feature of *Desulfobacteraceae* is their larger genome sizes on average (5.6 Mbp) compared to those of the other analyzed SRB (average of 4.2 Mbp) (Fig. 2A) and the available (>35,400) bacterial genomes in general (average of 3.6 Mbp; fig. S2). Further, a higher genomic plasticity of *Desulfobacteraceae* genomes is evident from the large number of mobile genetic elements (greatly exceeding that of *Desulfovibrionaceae*), CRISPR-Cas loci, and phage-related genes (tables S6 and S7).

Next, we compared the genomic shares dedicated to specific functional groups [cluster orthologous groups (COGs)] across the 27 selected genome sequences by determining the cumulative nucleotide proportion of each COG category across the respective open reading frame (ORF) set (Fig. 2A and fig. S3). Principle components analysis (PCA) of these proportions separated most *Desulfobacteraceae* from the other SRB, particularly *Desulfovibrionaceae* members. This separation is driven by the categories energy metabolism (C), signal transduction (T), and lipid metabolism (I), as well as by genes not categorized (NIC) (Fig. 2A, inserted loadings plot). Coclustering of completely oxidizing *Desulfomonile tiedjei* and *Syntrophobacter fumaroxidans* reflects their similarly broad nutritional versatility [e.g., (*25*)].

Focussing now specifically on *Desulfobacteraceae*, we studied their genomic diversity by expanding the set of the 12 aforementioned genomes with 31 additional reference genomes (according to GenBank) of experimentally less well-studied strains of this family. t-Distributed stochastic neighbor embedding (t-SNE) analysis of these 43 genomes separated them into seven distinct clusters (Fig. 2B). Two coherent, genus-specific clusters were observed for *Desulfatibacillum* and *Desulfosarcina*; the outlying *Desulfosarcina* sp. strain BuS5 differs from other *Desulfosarcina* spp. by its smaller genome size (4.1 Mbp) and extreme metabolic specialization on short-chained *n*-alkanes (*26*). By contrast, five clusters comprised two to three genera each, while one cluster was most diverse, harboring seven genera. One may speculate that these heterogeneities may have arisen from co-occurrence and linked rates of horizontal gene transfer or lower degree of specialization.

### *Desulfobacteraceae* core/pan genome

On the basis of the 43 *Desulfobacteraceae* reference genomes, we determined a rather small core of 713 clusters of ortho-groups, which account for only 4% of the total pan genome. The corresponding pan genome is open, i.e., new genes are found per genome added. This may be due to the broad definition of the core at the family level (rather than species or genus), sample size, and environment. However, because the alpha value is 0.9 ± 0.01, sample size can be regarded as representative. Modeling the number of ortho-groups found per novel genome shows the collection to be near saturation (fig. S4).

The core genome mainly provides essential cellular functions (DNA replication, transcription, and translation), dissimilatory sulfate reduction together with redox complexes (Dsr, Qmo, and Rnf2), ATP synthase, and enzymes of central metabolism. Notably, 89 proteins of unknown function are included (~12% of the core genome) and 150 regulatory proteins. The *Desulfobacteraceae* typifying capacity for complete oxidation is not explicitly assigned to the core genome because it is implemented by different pathways, i.e., the WLP for most (33 of 42) members, and the tricarboxylic acid (TCA) cycle for the genus *Desulfobacter.* The genera *Desulfobotulus* and *Desulforegula* harbor incomplete oxidizers despite their phylogenetic affiliation with the family *Desulfobacteraceae*.

### Proteomic datasets of six representative *Desulfobacteraceae* members

**Fig. 3.**
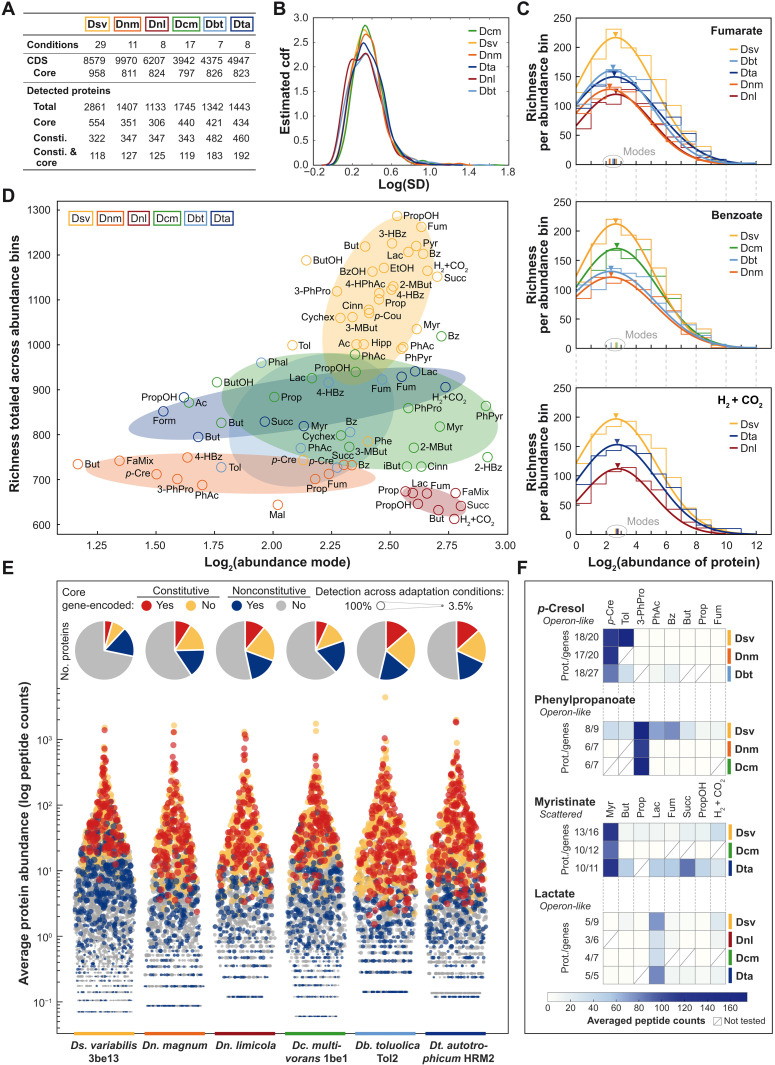
Proteogenomic commonalities among six representative *Desulfobacteraceae* members and across various substrate conditions. (**A**) Summary of the different datasets and protein categories per strain: *Ds. variabilis* 3be13 (Dsv), *Dc. multivorans* 1be1 (Dcm), *Dn. limicola* (Dnl), *Dn. magnum* (Dnm), *Db. toluolica* Tol2 (Dbt), and *Dt. autotrophicum* HRM2 (Dta). (**B**) Kernel smoothed estimated cumulative distribution function (cdf) of log SD of abundance per protein for each of the six strains. (**C**) Histograms (staircases; log bin size of 1) showing the number of different protein species (richness; ordinate) sorted in groups (bins) of increasing abundance (abscissa) in those strains that can use fumarate, benzoate, or H_2_ + CO_2_ for growth. All abundance distributions are log normally distributed (fitted smooth lines). The peak of the distribution (triangles) defines the abundance mode (vertical bars on abscissa), i.e., the abundance value shared by the largest number of different proteins. Histograms for the other growth substrates are presented in figs. S12 and S13. (**D**) Discrimination diagram based on totaled protein richness per strain-substrate combination versus corresponding mode of abundance distribution. Abbreviations of growth substrates are explained in legends to figs. S6 to S11. Ellipses approximate the data distributions for each strain based on eigenvector analysis. (**E**) Swarmchart of substrate-averaged log protein abundances for each strain, emphasizing the categories (Fig. 3A) core-gene encoded and constitutively formed or not (color coding as indicated in insert) with circle sizes proportional to detection across tested substrate conditions. The proportion of the different categories relative to the total number of detected proteins is indicated in the pie chart insets. (**F**) Heatmaps of averaged abundance of proteins involved in the indicated selected catabolic pathways. Respective genomic organization is given as well as the number of identified proteins (prot.) and total of encoding genes. Abbreviations of growth substrates are explained in legends to figs. S6 to S11.

**Fig. 4.**
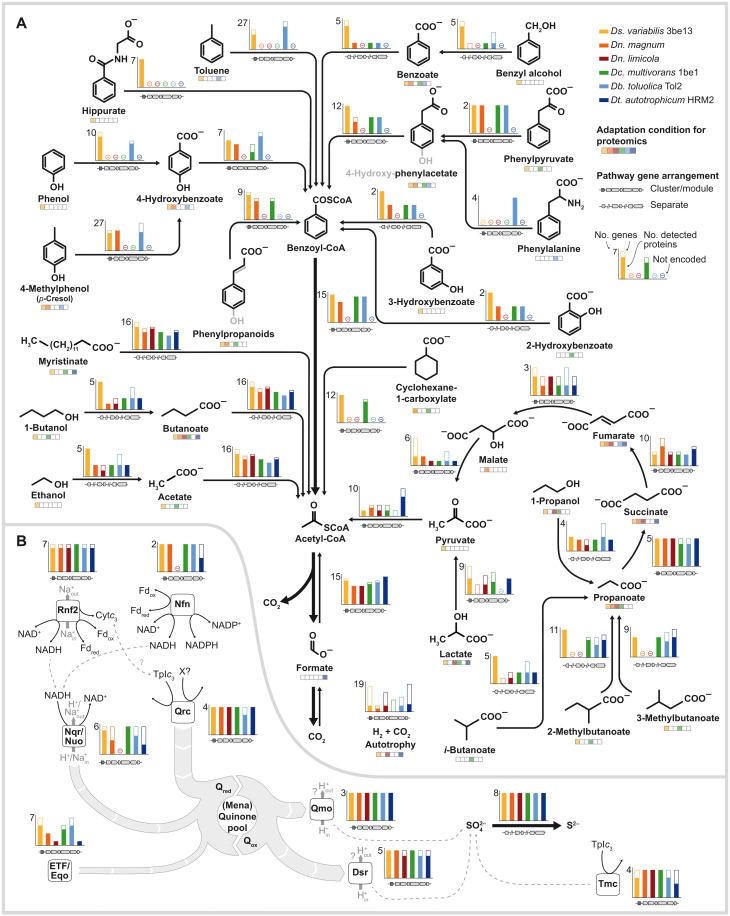
Catabolic meta-network of *Desulfobacteraceae* illustrating their collaborative contribution to the seabed carbon/sulfur cycles. (**A**) Substrate conditions are represented by chemical structures and proteomic analysis of the respective condition per strain is indicated below the structure. Bar charts represent the number of proteins involved in the respective pathway (maximum indicated), and filled bars give the number of proteins detected for this pathway per strain. A minus indicates the absence of the respective genes. Gene arrangement, i.e., cluster/module or separate genes (nonclustered) is indicated below the arrow. (**B**) Dissimilatory sulfate reduction and related redox complexes involved in electron transfer.

**Fig. 5.**
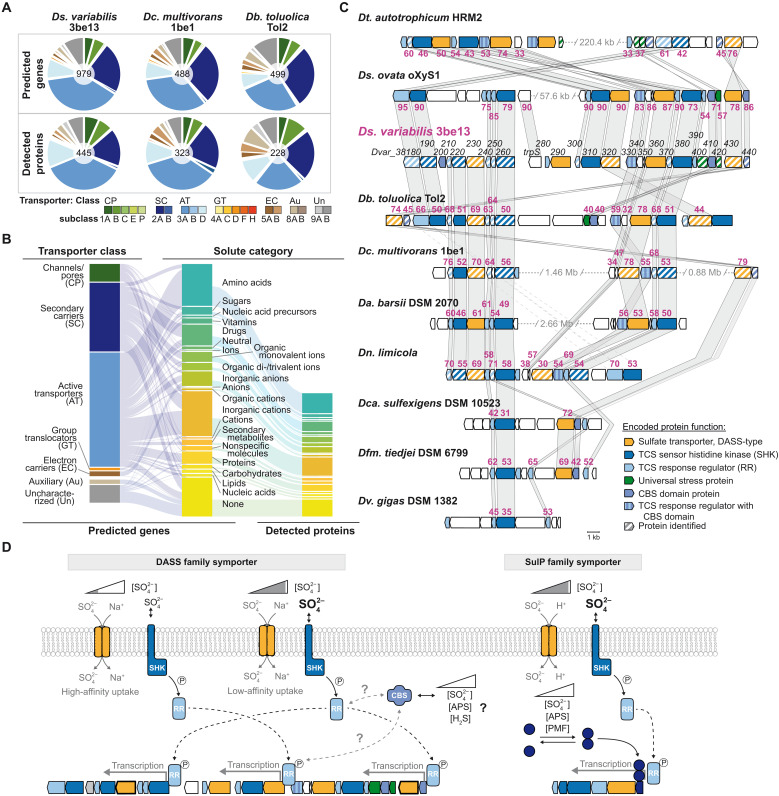
Broad transport capacities of selected *Desulfobacteraceae* and common, fine-tuned regulation of sulfate uptake. (**A**) Proportions of predicted transport proteins according to TCDB in the genomes and experimentally detected proteomes. Transporter class abbreviations: AT, active transporters; Au, auxiliary; CP, channels/pores; EC, electron carriers; GT, group translocators; SC, secondary carriers; Un, uncharacterized (for more details, see fig. S20). (**B**) Mapping of the *Ds. variabilis* 3be13 genome encoded transport proteins of different transporter classes to solute categories and their corresponding proteomically detected fractions. Color coding as given in (A). (**C**) Scale model of the DASS-type sulfate transporter encoding gene cluster of *Ds. variabilis* 3be13 and homologous clusters in other SRBs. Homologous genes are connected by gray shading, and amino acid sequence identities to genes of *Ds. variabilis* 3be13 are presented in violet. Locus-tags (or gene names) are given in black font, and genes highlighted with hedged filling have identified encoded proteins. Color coding of predicted functions is indicated by the inset. (**D**) Proposed model of the regulation circuits controlling sulfate uptake in *Desulfobacteraceae* exemplified for *Ds. variabilis* 3be13. Genes framed with thick lines encode transporters abundantly formed under high sulfate concentrations. Color coding: as given in (C); dark blue, TetR-type repressor. Arrows: black full line, phosphoryl transfer; black dashed line, promotor interaction of activated response regulator or repressor; gray dashed lines, putative protein-protein interaction. SHK, sensory histidine kinase; RR, response regulator; CBS, CBS domain protein.

How is the known nutritional versatility of *Desulfobacteraceae* accomplished on the protein level? The genome-shaping role of metabolism and regulation recognized here (Fig. 2A) prompted us to investigate the general architecture and substrate-dependent dynamics of proteome landscapes (Fig. 3), catabolic networks (Fig. 4), and transportomes (Fig. 5). For this purpose, we selected six *Desulfobacteraceae* members considering differences and commonalities, respectively, in (i) nutritional versatility, (ii) specific lifestyles, (iii) environmental abundances of genera, and (iv) phylogenetic representation of the family (table S6). We studied in total 35 different substrate adaptation conditions, comprising numerous aromatic (including hydrocarbons) and aliphatic compounds as well as H_2_ + CO_2_ (chemolithoautotrophy): 29 for *Ds. variabilis* 3be13 (this study), 7 for *Desulfobacula toluolica* Tol2 (*27*), 17 for *Desulfococcus multivorans* 1be1 (*28*), 8 for *Desulfonema limicola* (*29*), 11 for *Dn. magnum* (*29*), and 8 for *Desulfobacterium autotrophicum* HRM2 (*30*). In total, 80 different proteomic datasets (i.e., strains and their respective substrate conditions) formed the basis for the subsequent comparative analyses. Coherence across these datasets was ascertained by consistently applying approach-specific standards to manual genome annotations, cultivations for substrate adaptations, and full-cycle proteomics, respectively. Combining gel-based and gel-free methods applied to soluble and membrane protein-enriched fractions yielded an exclusive protein detection of ~33% per fraction (fig. S5). These experiments covered 1133 to 2681 different proteins, depending on the strain and, on average, 652 to 1099 different proteins per tested substrate condition (Fig. 3A).

### Proteome-shaping determinants

The high proteomic coverage (Fig. 3A), i.e., the large proportion of detected to genome-predicted proteins, enabled us to determine the abundance range for each detected protein across all substrate-adaptation conditions for each of the six studied strains. We expressed this range as standard deviation and doing so for all proteins yielded a strain-specific distribution of abundance variability as displayed in Fig. 3B. The resulting distributions reveal a common pattern across all strains. As indicated by the peaks in the distributions, centered over low variability (Fig. 3B), the vast majority of proteins do not differ greatly in their abundances across the substrate conditions, and thus, they represent general cellular and metabolic functions. In contrast, only a small fraction of proteins exhibits a large spread in abundance (upper tail of the distribution in Fig. 3B). These highly differentially abundant proteins reflect specific adaptations to the tested substrates. This is exemplified for *Ds. variabilis* 3be13 in fig. S6, showing distinct abundance maxima of the 2681 identified proteins in each of the 29 substrate conditions; analogous heatmaps for the other five strains are provided in figs. S7 to S11.

Across tested substrate conditions and strains, the abundance profiles of detected proteins are markedly similar and of monomodal shape, which can be well approximated by a log-normal distribution (Fig. 3C and figs. S12 and S13). The horizontal location and scale parameters of the profiles are highly similar, indicating a fairly consistent proteome budget implemented by the six strains. Integrating all tested substrate/strain conditions, we find that the strain determines proteome richness (diversity of detected proteins) more than the substrate does (Fig. 3D). *Ds. variabilis* 3be13 produces the greatest diversity of proteins, while the two *Desulfonema* spp. the least.

### Constitutive versus substrate-specific subproteomes

Here, we define the strain-specific constitutive proteome as the set of proteins shared among all studied growth conditions. For the six studied strains, the constitutive proteome ranged from 322 (*Ds. variabilis* 3be13) to 482 (*Db. toluolica* Tol2) different proteins [3.5 to 11.0% of all coding sequence (CDS); Fig. 3A and data S1]. Notably, these proteins account for 72.8 to 91.9% of all peptide counts as a measure of abundance (orange and red in Fig. 3E), and thus, they dominate the cellular protein content. By contrast, the nonconstitutive proteins (blue and gray in Fig. 3E) are much more diverse but represent a considerably smaller proportion of the total cellular protein content. The overlap between core genomes and constitutive proteomes of the six strains is fairly small, with only about a third (34.7 to 41.7%, red in Fig. 3E) of the constitutive proteins encoded within the core genome.

To add a functional dimension, we analyzed the constitutive proteomes for their allocations to Kyoto Encyclopedia of Genes and Genomes (KEGG) pathway modules. In general, the largest portions of the strain-specific constitutive proteomes are associated with energy metabolism (17.4 to 25.5%), carbohydrate metabolism (16.4 to 22.3%), and amino acid metabolism (7.6 to 13.3%) (table S8). In particular, the six constitutive proteomes include *Desulfobacteraceae*-typifying metabolic modules, such as the dissimilatory sulfate-reduction pathway with its diverse associated membrane protein complexes, as well as the WLP for complete substrate oxidation (table S8). Notably, the constitutive proteomes harbor numerous uncharacterized proteins (52 to 85).

Apparently, the six studied strains invest similar resources from their constitutive proteomes in the aforementioned metabolic modules. To assess whether these commonalities also persist when examined in greater functional detail, we performed multidimensional analyses, which now considered abundances (peptide counts) and functions (KEGG orthology) of individual proteins. This higher-resolution analysis again resulted in a strain-specific, viz., substrate-independent separation (figs. S14 and S15), thus substantiating the superior role of each individual strain in defining its constitutive proteome.

Next, we studied the nonconstitutive proteomes by focusing on peripheral degradation routes. Their protein constituents showed similar average abundance maxima across strains using these routes in a substrate-specific manner. This is exemplified in Fig. 3F for *p*-cresol as a phenolic compound with toxic properties, phenylpropanoate as a carboxylated aromatic compound and lignin building block, myristinate as a long-chain fatty acid, and lactate as an abundant fermentation product, widely used by SRB. Notably, various degrees of regulatory stringency were observed across tested strains and substrates, e.g., phenylpropanoate-related proteins with relaxed specificity in *Ds. variabilis* 3be13 versus high specificity in *Dn. magnum* and *Dc. multivorans* 1be1. This points to sensory/regulatory commonalities among members of the *Desulfobacteraceae*, in parts amended by strain-specific features.

### Meta-network of catabolism

To unravel the biochemical basis underlying the substantial contribution of *Desulfobacteraceae* to the marine C/S cycles, we reconstructed de novo the catabolic network of highly versatile *Ds. variabilis* 3be13 and collated it with those previously elaborated for the other five strains (*27*–*30*). The synthesis (meta-)network recruits 119 (*Db. toluolica* Tol2) to 235 (*Ds. variabilis* 3be13) protein-encoding genes (median 2.7% of CDS per genome), which were covered by proteomics to the greatest extent (88.1 to 97.9% per strain). This meta-network comprises in total 319 different proteins and provides a holistic perspective of *Desulfobacteraceae* catabolism.

The degradation part of the meta-network accommodates 35 substrates (Fig. 4A) and is characterized by a high degree of modularity, explaining substrate versatility, combined with a few central modules (benzoyl-CoA pathway and WLP). Together, this part of the network (except for autotrophy) illustrates the manifold contributions to the oxidative side of the marine carbon cycle. Degradation routes for chemically stable aromatic compounds (and cyclohexane carboxylate) are organized in self-containing modules. These are encoded in operon-like structures that harbor not only the genes for degradation enzymes but also genes for associated electron transfer flavoproteins (ETFs), ETF:quinone oxidoreductase (Eqo), sensory/regulatory proteins, and often even transporters. Across studied strains sharing a given module, gene organization, protein sequences, and differential protein abundances are highly similar, as exemplified for the peripheral route of *p*-cresol degradation (up to 86% identity at the protein level; fig. S16, A and B) and the central benzoyl-CoA pathway (up to 85% identity; fig. S16, C and D). By contrast, genes for simpler substrates, e.g., fatty acids and aliphatic alcohols, as well as those for the WLP are usually scattered across the studied genomes. A wealth of alcohol dehydrogenases and oxidoreductases are encoded in all six genomes (e.g., 16 each in *Ds. variabilis* 3be13), which are dedicated to specific functions, e.g., β-oxidation of short- to long-chain fatty acids, and share a high degree of sequence similarity (>70%). The extensive metabolic repertoire also covers chemolithoautotrophy (H_2_ and CO_2_), except for *Dn. magnum* and *Dc. multivorans* 1be1 the genomes of which do not contain genes for hydrogenases. Overall, regulatory proteins encoded in direct proximity or within the gene cluster of a given pathway module are mostly single-component transcriptional activators (e.g., σ^54^-dependent) and are conserved across strains (e.g., TetR type in the case of *p*-cresol).

The energy metabolism section of the meta-network (Fig. 4B) is multilayered comprising general and more specialized functions and illustrates the contribution to the marine sulfur cycle. The equipment for dissimilatory sulfate reduction (APS-synthase/-reductase and sulfite reductase) together with electron-delivering Qmo and Dsr (from the quinone pool) and Tmc complexes is canonical for SRB [e.g., (*1*, *31*)]. Except for Tmc, all components are highly abundant members of the constitutive proteomes of all six strains. We assume that the (mena)quinone pool is replenished, mainly by the Qrc complex, due to its abundant and constitutive formation. However, the Qrc of *Desulfobacteraceae* apparently differs from the well-studied periplasmic hydrogenase-linked Qrc of *Desulfovibrio* spp. due to marked sequence dissimilarities of QrcA (electron entry) and QrcB (structural function) (Supplementary Text and fig. S17). In addition, low abundance, substrate-specific formation of hydrogenases, or even lack of encoding genes in *Desulfobacteraceae* implicate a different electron donor. A further route of electron delivery to the quinone pool involves reduced forms of nicotinamide adenine dinucleotide (NADP^+^) (NADH)–oxidizing Nqr/Nuo complexes. The NADH pool, in turn, is not only replenished via oxidation reactions of the degradation network but also is linked to the ferredoxin and NADP pools via Rnf2 and Nfn. Notably, the transmembrane sodium-pumping Rnf2 complex is abundantly and constitutively formed in all six strains. Rnf2 differs with respect to the ferredoxin/NAD-interacting domains of RnfBC from the Rnf1 complex occurring in *Desulfovibrionaceae*, other bacteria, and archaea (Supplementary Text and fig. S18). Last, electrons are delivered to the quinone pool via ETF/Eqo pairs, which are tailored to specific degradation reactions, encoded in the respective pathway modules and substrate specifically formed—an apparently characteristic feature of *Desulfobacteraceae* members.

We next explored to what degree the genome-inferred regulatory potential (Fig. 2A) of *Desulfobacteraceae* is actually implemented. For this purpose, we subjected the comprehensive differential proteomic datasets of each strain to cluster analysis (figs. S6 to S11), which groups proteins according to their similarity in abundance patterns across substrate conditions. The resultant 85 groups were manually inspected (figs. S26 to S120). A notable observation was the strong correlation between the grouping and the aforementioned modularity of the catabolic networks. That is, most identified protein components of a given module gather in a single group irrespective of strain and genetic organization (operon-like versus scattered; exemplified for Bam proteins of the central benzoyl-CoA pathway in fig. S19). This suggests a high degree of conservation of regulatory systems across members of the *Desulfobacteraceae*.

### Transporter complement

Given the broad versatility of *Desulfobacteraceae*, one should expect a matching transporter repertoire. To determine the transportomes of the six strains, we used the comprehensive Transporter Classification Database (TCDB) (*32*), as COG categories do not consider transporters as distinct categories. In total, 384 to 565 transport systems are encoded (4.2 to 12.3% of CDSs), with secondary carriers and active transporters being the most abundant classes (58.9 to 73.2% of all transport proteins) (Fig. 5A, fig. S20, and table S9). The proportions of the predicted and detected transporter subclasses are essentially congruent among the studied SRB. We further scrutinized the solute spectrum of the six transportomes resolved based on transporter classes (exemplified for *Ds. variabilis* 3be13 in Fig. 5B). Amino acids represent the major transport solute, followed by drugs (mainly antibiotics), inorganic anions (mainly sulfate and phosphate), inorganic cations (mainly protons and sodium), and unknown compounds (Fig. 5B). Notably, the differential proteome dynamics (Fig. 3F) observed for the meta-network of catabolism are mirrored at the transporter level. While substrates of peripheral degradation modules are imported by specifically formed uptake systems (e.g., DctPQM4 for 4-hydroxyphenylacetate), general substrates such as sulfate enter the cell via constitutively synthesized transporters (e.g., sulfate-importing Dvar_38430).

### Sulfate uptake and its regulation in SRB

For concentration-dependent sulfate uptake, SRB mostly use high- and low-affinity, energy-efficient secondary transporters driven by Na^+^ (DASS-type) or H^+^ gradients (SulP types) (*33*–*36*). Using TCDB, we identified a 30.5–kilobase pair (kbp) gene cluster in the genome of *Ds. variabilis* 3be13 encoding the abundantly and constitutively formed DASS-type sulfate transporters (i.e., Dvar_38230 and Dvar_38430) and numerous two component systems (TCSs) and CBS-domain proteins (Fig. 5C and fig. S21). Notably, related gene clusters are present in all studied *Desulfosarcina* spp. (up to 95% identity), *Desulfobacteraceae* (<80%), and 14 other SRB (<50%, in total 26 species; fig. S22A). Another gene cluster encoding the similarly abundant and constitutively formed SulP transporter is present in *Ds. variabilis* 3be13. It is widespread among *Desulfobacteraceae* (and *Dv. salexigens*; fig. S22BC) and also harbor genes for TCSs and a TetR-type repressor. The sensor histidine kinases (SHKs) of both gene clusters share very similar sensory domain structures, including a small molecule recognizing N-terminal cache (IPR033479) domain (*37*). Indeed the 28 SHKs in diverse SRBs share 30 (of 220) identical or similar amino acids in the signal recognition domains, and 47 additional residues are similar in >75% of the sequences (fig. S23). This indicates a common effector, e.g., sulfate. The corresponding response regulators (RRs) are of the WalR type. The observed SHK structure and RR combination is reminiscent of the PhoRB TCS from *Escherichia coli* and many other bacteria, which regulates high-affinity phosphate uptake in response to phosphate limitation (*38*, *39*). Notably, in *Mycobacterium smegmatis* the PhoRB-mediated regulation of phosphate uptake is augmented by the repressor PhnF (*40*). Similarly, the *sulP* gene clusters of *Desulfobacteraceae* encode a TetR-type repressor with a matching upstream operator sequence (i.e., 16-bp palindromic nucleotide sequence located ~20- to 40-bp upstream of the transcriptional start site; fig. S24) (*41*). Last, CBS domains are known to play regulatory roles, e.g., in response to adenosyl group–containing ligands including ATP and *S*-adenosylmethionine or ions (e.g., Mg^2+^) (*42*) or AMP level–dependent activity of the AMP-activated protein kinase (*43*). Hence, one may speculate that the studied SRB link intracellular APS levels with sulfate uptake. It is also conceivable that intracellular sulfate or sulfide levels are sensed, analogous to the Mg^2+^ responsiveness of the magnesium transporter MgtE (*Thermus thermophilus*) mediated by a CBS domain–containing regulatory protein (*44*).

In conclusion, a fine-tuned regulatory mechanism for sulfate uptake shared among SRB may operate as follows (Fig. 5D): SHK-based sensing of external sulfate yields formation of Na^+^-dependent DASS uptake systems (high or low affinity) and H^+^-dependent SulP transporters (at high sulfate levels). A second control level may respond to intracellular levels of sulfate (or S^2−^) or the general energy status of the cells [e.g., APS, ATP, and proton motive force (PMF)] involving CBS domain proteins (DASS cluster) and TetR repressors (SulP cluster). Such a fine-tuned regulatory network of sulfate uptake and, hence, sulfate reduction/energy metabolism may ultimately allow for a higher energy yield and, therefore, represent a building block for the environmental success of *Desulfobacteraceae* and *Desulfosarcina* spp. in particular.

### Characteristic “catabolic” genes of *Desulfobacteraceae* prevail in marine sediments

**Fig. 6.**
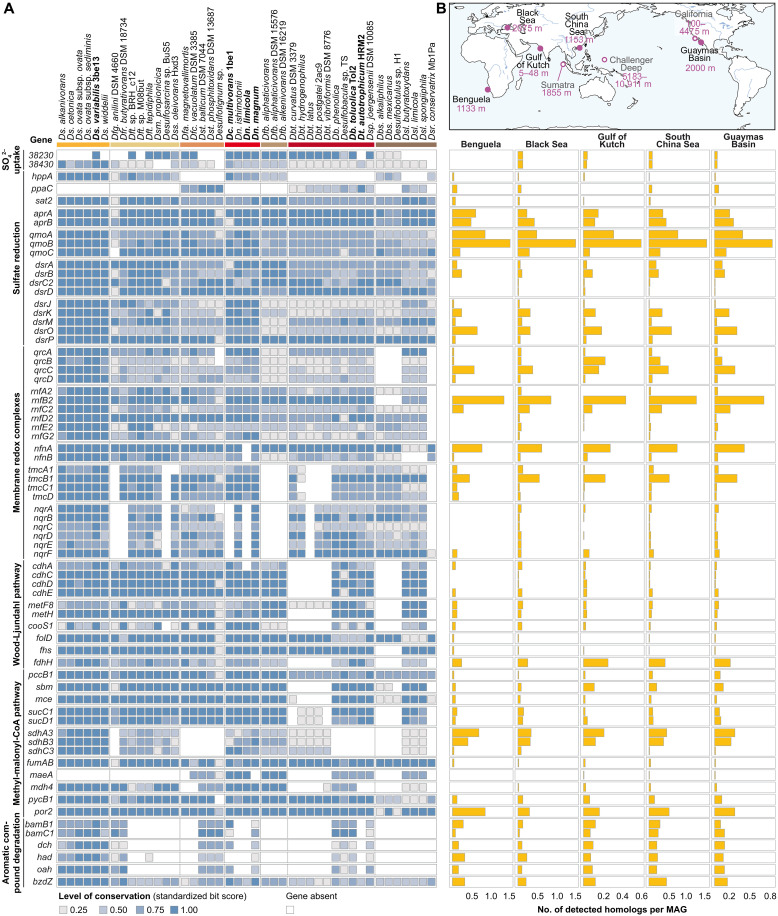
Characteristic *Desulfobacteraceae* catabolic genes are prevalent in marine sediments. (**A**) Comparison of 43 *Desulfobacteraceae* reference genomes. Heatmap of similarity (i.e., level of conservation) of selected genes related to sulfate uptake and reduction, membrane redox complexes, Wood-Ljungdahl pathway, methyl-malonyl-CoA pathway, and aromatic compound degradation. Coloring of species reflects clustering of genomes presented in Fig. 2B. Abbreviations of genus names: Dbt, *Desulfobacter*; Dbs, *Desulfobotulus*; Dfa, *Desulfamplus*; Dfb, *Desulfatibacillum;* Dfc, *Desulfocicer*; Dfr, *Desulfatirhabdium*; Dfg, *Desulfatiglans*; Dft, *Desulfatitalea*; Dsl, *Desulfoluna*; Dsm, *Desulfosalsimonas*; Dsp, *Desulfospira*; Dsr, *Desulforegula*; Dss, *Desulfosudis*; Dst, *Desulfotignum*. Gene names and locus-tags refer to *Ds. variabilis* 3be13. (**B**) Location of queried metagenome samples and corresponding depth below sea surface (top inset). Distribution patterns of detected homologs in MAGs from sites marked by filled circles. Corresponding data of all sites is provided in fig. S25, diversity profiles is available in table S10, and detailed data is available in Data S2.

Integration of the constitutive proteome (Fig. 3) with the experimentally substantiated catabolic meta-network (Fig. 4), allowed us to define a set of characteristic “catabolic genes” shared among the 43 genome-sequenced *Desulfobacteraceae* members (Fig. 6A) and explore their occurrence in reported high quality metagenomes from marine sediments (Fig. 6B).

As mentioned earlier, the machinery of energy metabolism, comprising sulfate uptake, its dissimilatory reduction (pyrophosphatase activity alternatively via HppA or PpaC), and the associated redox complexes (Fig. 4B) are conserved among all investigated *Desulfobacteraceae* species (Fig. 6A). Particularly noteworthy is the widespread occurrence of the Rnf2 complex. With respect to the degradation network (Fig. 4A), the characteristic WLP occurs in all investigated *Desulfobacteraceae* species, except *Desulfobacter* spp., which use the TCA cycle (note that *Desulfobotulus* and *Desulforegula* are incomplete oxidizers). The anaerobic benzoyl-CoA pathway (BamBC) is restricted to selected genera (*Desulfosarcina*, *Desulfobacula*, *Desulfonema*, and *Desulfotignum*) capable of aromatic compound degradation. Overall, meta-network genes are highly conserved within the family *Desulfobacteraceae*, irrespective of how widespread they are across the 43 studied genomes.

We detected essentially all constituents of this deduced gene set in diverse shallow-to-deep-sea sediments (Fig. 6B), i.e., from an upwelling system (Benguela region, off the coast of Namibia); below a stratified, euxinic waterbody (Black Sea); an organic-rich estuarine (Gulf of Kutch, India); a cold-seep field [north-eastern slope of South China Sea (SCS)]; and a deep-sea, hydrothermal area (Guaymas Basin, Gulf of California). We saw similar functional-organismic profiles for the Sumatra upwelling system and the Pacific Ocean off the coast of California (USA) (fig. S25; details for all studied sites are provided in data S2). Together, these findings underpin the metabolic contribution of *Desulfobacteraceae* members to carbon mineralization in these geographically far apart and geochemically different habitats. While Desulfobacterales were consistently observed among the top-ranking orders of the seven sites (table S10), other prominent orders occurred only partially, for example, putatively carbohydrate and/or amino acid–degrading members of anaerobic Aminicenantales (OP8, uncultivated) (*45*, *46*) at sites with high input of organic material due to upwelling (Benguela, Sumatra) or via multiple river mouths in combination with oxygen-depleted bathypelagial (Black Sea). Likewise, in the estuarine environment of the Gulf of Kutch, nutrient input from agricultural discharge apparently leads to a prominent co-occurrence of facultative anaerobic, nitrate-reducing Pseudomonadales and nitrite-oxidizing Nitrospinales (*47*–*49*). Pseudomonadales are also well detected in sediments off the coast of California. The microbial community of SCS harboring the queried genes was most complex with Bacteriodales and Anaerolineales representing the prevailing taxa ranking behind Desulfobacterales, which agrees with the generally appreciated microbial richness of cold-seep ecosystems (*50*, *51*). The prevalence of Desulfobacterales and Methanosarcinales members in the Guayamas Basin sediment could be attributed to their association with anaerobic hydrocarbon degradation (*1*) and the unique role of these sediments as anoxic hydrocarbon ecosystems (*52*). Across these seven different types of marine sediments, the relative abundance of Desulfobacterales members harboring the queried genes ranged from 1.5 to 20.0% (median values). By contrast, the query genes were not detected in the Challenger Deep sediment, where oxic and nutrient-depleted conditions prevail (table S10) (*53*).

## DISCUSSION

Our proteogenomic analysis was devised to understand the catabolic basis of the long-appreciated role of SRB and *Desulfobacteraceae*, particularly in the interwoven carbon and sulfur cycles of marine sediments. The five genera selected for our comparative approach are indeed cosmopolitan thriving in diverse marine habitats, underscoring the transferability of the present findings to the family of *Desulfobacteraceae* in general.

We identified two family-defining metabolic key properties: (i) the pathway-module inherent coupling of substrate-degradation via electron transfer proteins (tailored to individual oxidation reactions) to membrane-embedded redox complexes and dissimilatory sulfate reduction; (ii) a common catabolic network architecture, where multiple substrate specifically regulated pathway modules (peripheral degradation routes) feed into few constitutively formed central modules of degradation and energy metabolism. Both properties together contribute to an energy efficient exploitation of diverse substrates, ultimately enabling life at the thermodynamic limit and fostering environmental success.

The constructed meta-network reveals a broad range of shared and strain-specific degradation capacities among the six studied strains. This may even be underestimated given the breadth of the transporter repertoire, which greatly exceeds the number of tested substrates (plus required nutrients) and may therefore provide access to hitherto unknown substrates/pathways. Furthermore, the inclusion of *Desulfobacteraceae* strains with not yet considered physiologies, e.g., *n*-alkane (*54*) and polymer degraders (*55*), will expand the catabolic diversity of our meta-network even more. Hence, the high sulfate reduction rates in organic-rich, marine sediments do not rely on individual key species, but rather result from the additive degradation capacities of site-specific SRB communities (communal accomplishment). Yet, the *Desulfosarcina* and *Desulfococcus* species currently stand out by exceptionally numerous degradation modules, both reflecting their known broad substrate-spectrum and rationalizing their dominance in SRB communities of marine sediments. The prevalence and conservation of the degradation modules across the 43 representative *Desulfobacteraceae* genomes hints at their niche-defining role. We speculate that the wealth of these modules was achieved by comprehensive horizontal gene transfer implicated by the genomes’ richness in mobile elements. Overall, these congeneric yet tailored genomic, regulatory, and catabolic capacities of *Desulfobacteraceae* shape their environmental function and success, ultimately imprinted in their global biogeography.

In the light of the burgeoning large-scale metagenomic studies [particularly metagenome-assembled genomes (MAGs) [e.g., (*56*)], the here presented metabolism-centered insights into the *Desulfobacteraceae* provide a “treasure-trove” from which to select target genes for functional analysis of SRB communities in natural and technical environments. First, it can complement incubation experiments with labeled substrates that target the active part of the community [e.g., (*57*)]. Second, given their enormous carbon turnover in the seabed, *Desulfobacteraceae* should substantially shape the dissolved organic matter (DOM) (*58*), e.g., by depleting a broad range of organic substrates while enriching the recalcitrant fraction. Such microbial activities in the sediment should also feedback on DOM in the water column (*59*), particularly in shelf seas, when considering vertical exchange processes across the bottom boundary layer (*60*). Third, symbiotic/commensal relations of marine SRB with, e.g., marine oligochaete worms (*61*), benthic foraminifera (*62*), or seagrass/salt marsh cordgrass (*63*) and non-marine SRB with, e.g., the human gut [e.g., (*64*)], can be studied on a functional level. Fourth, the mechanistic understanding of deleterious effects of SRB on technical installations can be improved, e.g., production and processing facilities of the gas and oil industry [e.g., (*65*)], as well as sites of metal and concrete corrosion [e.g., (*66*, *67*)].

## MATERIALS AND METHODS

### Literature-based meta-analysis of habitat information

For meta-analysis of the genera *Desulfococcus*, *Desulfosarcina*, *Desulfonema*, *Desulfobacterium*, and *Desulfobacula* (Fig. 1), geographical and physicochemical parameters were systematically extracted from the primary literature from 1980 until 2019 using entries from PubMed [National Center for Biotechnology Information (NCBI), Bethesda, MD, USA], Scopus (Elsevier B.V., Amsterdam, The Netherlands; until 2017), and the Web of Science (Clarivate, Philadelphia, PA, USA). An advanced keyword search was performed using the terms (i) sulfate reduction, (ii) sulfate reducing or (iii) sulfate reducer* and (iv) bacter*, (v) archaea*, or (vi) desulfo* (also including British spelling using “ph”). Respective citations were submitted to EndNote (version 9X, Clarivate), including deduplication, and obtained pdf files searched for the genera names. Last, all studies (in total 2471) were manually screened for environmental samples, and the detection/description of the respective genera based on isolates, 16*S* ribosomal RNA (rRNA) encoding genes, *aprAB* or *dsrAB* genes, or specific FISH probes. In the case of detection (in total 368 references, tables S1 to S5), physicochemical parameters of the corresponding habitat were extracted from the respective manuscripts and supplementary materials.

On the basis of the GPS coordinates of the sample locations and using the cartopy package for the programming language python 3 (https://scitools.org.uk/cartopy), sites were clustered according to the density-based spatial clustering of applications with noise (DBSCAN) algorithm implemented in the python scikit-learn package. As parameters for DBSCAN, we used a *d*_max_ value of 300 km for global view (Fig. 1A) and 25 km for detailed views (fig. S1), applying great circle distances between sites as metric. *d*_max_ defines the maximal distance between two sites for them to be clustered together. The plate carrée projection (equidistant cylindrical projection) was used for presenting the worldwide distribution of clusters.

### Organism, media, and cultivation

*Ds. variabilis* (strain 3be13, DSM 2060) was originally isolated from anoxic marine sediments of a Mediterranean lagoon (Montpellier, France) (*68*) and obtained from the Deutsche Sammlung von Mikroorganismen und Zellkulturen (DSMZ), Braunschweig, Germany. It was cultivated under strictly anoxic, sulfate-reducing (20 mM sulfate) conditions in 500-ml flat bottles containing 400 ml of defined bicarbonate-buffered, sulfide-reduced (1.5 mM Na_2_S) brackish water medium (*69*) at 28°C with the following growth substrates (in alphabetical order; concentration is given in parenthesis): acetate (20 mM), benzoate (4 mM), benzyl alcohol (4 mM), butanoate (5 mM), 1-butanol (5 mM), cinnamate (3 mM), *p*-coumarate (5 mM), *p*-cresol (3 mM), cyclohexane carboxylate (3 mM), ethanol (15 mM), fumarate (10 mM), H_2_ + CO_2_ (80:20, v/v), hippurate (4 mM), 3-hydroxybenzoate (4 mM), 4-hydroxybenzoate (4 mM), 4-hydroxyphenylacetate (5 mM), lactate (10 mM), 2-methylbutanoate (5 mM), 3-methylbutanoate (5 mM), myristinate (3 mM), phenol (3 mM), phenylacetate (4 mM), phenylpyruvate (5 mM), 3-phenylpropanoate (3 mM), propanoate (6 mM), 1-propanol (8 mM), pyruvate (15 mM), succinate (7 mM), and toluene (1% (v/v) in an inert carrier phase of 2,2,4,4,6,8,8-heptamethylnonane).

Cells of *Ds. variabilis* 3be13 were adapted to each of the above listed 29 substrate conditions over five passages before mass cultivation. Cells were harvested in mid-linear growth phase as described previously (*70*).

### DNA sequencing, assembly, and annotation

DNA was isolated with the Genomic DNA Kit (QIAGEN, Hildesheim, Germany) according to the manufacturer’s instructions. Recombinant plasmid and fosmid shotgun libraries were constructed. Plasmid libraries were generated from sonicated DNA (*71*). In addition, a fosmid library was constructed (>40-fold physical coverage) for data finishing and assembly confirmation (Epicentre Technologies, Madison, WI, USA). Templates for sequencing were obtained by insert amplification via PCR or by plasmid isolation. Sequencing was carried out using the ABI3730XL capillary systems (ABI, Waltham, MA, USA). In total, 88,614 sequencing reads were generated, and the programs PHRAP (Phragment assembly program 1999; www.phrap.org/phredphrapconsed.html) and Consed (*72*) were used to assess sequence quality and perform the assembly with a quality of less than 1 error in 100,000 bases.

Structural rRNAs and transfer RNAs (tRNAs) were determined using RNAmmer (v.1.2) (*73*) and tRNAscan-SE (*74*), respectively. Protein-coding sequences (CDS) were predicted by the ORF-finding program Glimmer3 (*75*) and manually revised and curated using Artemis (v.12.0) (*76*). The generated ORF dataset was screened against nonredundant protein databases (SWISSPROT and TREMBL), and the genome was manually annotated applying Artemis. The genome sequence of *Ds. variabilis* 3be13 has been submitted to GenBank under the BioProject PRJNA319746 with accession number CP159846.

### Bioinformatic analysis of the *Ds. variabilis* genome

Bacterial genome data consulted for genomic comparisons with *Ds. variabilis* 3be13 were accessed from public databases, e.g., Integrated Microbial Genomes (http://img.jgi.doe.gov/) or the NCBI (www.ncbi.nlm.nih.gov/). The Artemis software (v.12.0) (*76*) was applied for sequence and ORF-set visualization. Proteins were screened against SWISSPROT and TrEMBL (*77*) as well as InterPro (*78*) databases. A protein similarity search was performed by means of *Ds. variabilis*–specific blastp analysis (*79*). Genomic islands and islets (less than 10 kbp) were predicted applying IslandViewer 3 (*80*), and the CRISPR recognition tool (v.1.1.) (*81*) served for detection of CRISPR sequences.

### Comparative genomics

For all studied *Desulfobacteraceae* genome sequences (table S6), the respective GenBank files were used as templates to screen for phage-like regions using PHASTER (*82*), and only intact (score > 90) phages were considered. The EggNOG database (v.4.5) (*83*) was consulted for orthology prediction and functional categorization. The number of ORFs with unknown or conserved unknown function was extracted from GenBank files. However, in the case of *Ds. alkanivorans* PL12, *Ds. ovata* 28bB2^T^, *Ds. ovata* oXyS1, and *Ds. widdelii* PP31, no such differentiation was conducted. Genes encoding transposases or integrases were considered as mobile elements and counted per organism.

Genome occupancy (Fig. 2A) was calculated as the relative nucleotide proportion of genes per COG category, and PCA plots were generated using custom MATLAB (version 2022a) code. Thereby, for genes assigned to two or more COG categories, the respective share was equally allotted to the corresponding categories.

Genome selection and prediction of orthologs: Available RefSeq genomes from the order Desulfobacterales (*n* = 51) and their metadata were downloaded from NCBI (dataset v.14.2.2, accessed on 23 May 2023) (*84*) and dereplicated to exclude redundancy using dereplicator.py (v.0.3.0; https://github.com/rrwick/Assembly-Dereplicator), applying default settings. For the resulting collection of *Desulfobacteraceae* members (*n* = 43), protein-coding genes were predicted using Prodigal (v.2.6.3) (*85*). The translated amino acid sequences from the collected genomes and the independently assembled *Ds. variabilis* 3eb13 were clustered into groups of orthologs (ortho-groups) using OrthoFinder (v.2.5.4) (*86*), with diamond reciprocal-best-hit at an ultrasensitive mode and an inflation parameter of 3. Statistical analysis of the ortho-groups’ prevalence and frequency was conducted in RStudio (2023.03.1, build 446) (*87*) using R language (v.4.3.0) and the “tidyverse” package collection (v.2.0.0) (*88*). All scripts are available in the public repository https://github.com/rsiani/woehlbrand2024 and zenodo under doi: 10.5281/zenodo.14039560. Briefly, the ortho-groups count matrix, summarizing the number of protein-coding genes per genome belonging to an ortho-group, was obtained from Ortho-finder’s file “Orthogroups.GeneCount.tsv”. Using Heaps’ power law, as proposed by Tettelin and colleagues (*89*), the saturation rate of gene discovery was inferred. Barnes-Hut’s t-SNE (Rtsne, v.0.16) (*90*) was used to embed the genomes in a two-dimensional (2D) space constructed from the binary distances drawn from the count matrix (Fig. 2B). The genomes were then *k*-means clustered into the optimal number of groups, as determined by the average “silhouette” method (factoextra, v.1.0.7; https://github.com/kassambara/factoextra). A core genome of ortho-groups identified in at least 41 genomes (95% of the collection) was defined.

To understand the level of conservation of *Desulfobacteraceae* catabolic genes (DCGs) (Fig. 6A) in our collection, the procedure, first detailed by Wheeler and colleagues (*91*), was followed. Jackhmmer (hmmer v.3.1b2) (*92*) was used to produce multiple-sequence alignments of *Ds. variabilis* 3be13 DCGs with homologs from Uniref90 (release 2023_02) (*93*) with an inclusion *e* value of 1 × 10^−9^. The alignments were then converted to HMM profiles. The ortho-groups including *Ds. variabilis* 3be13 DCGs were scanned against the respective HMM profiles, and a delta bitscore was calculated by subtracting each sequence bitscore from the reference *Ds. variabilis* 3be13 ortho-group bitscore.

### Metagenomics

To evaluate the prevalence of DCGs in oceanic environments (Fig. 6B), publicly available MAGs were sourced from eight different locations: Benguela upwelling, PRJNA367444; Black Sea, PRJNA405475; Gulf of Kutch, PRJNA598416; Sumatra upwelling, PRJNA367445-446; South China Sea, PRJNA707313; Pacific Ocean off the coast of California, PRJNA620477-780, PRJNA653155, PRJNA654763-764, PRJNA654800-002, and PRJNA654836-837; Guaymas Basin, PRJNA362212; Challenger Deep, PRJNA635214. The previously generated HMM profiles were used to scan the MAGs for DCGs, and hits were quality-filtered based on sequence and domain scores, *e* values, and bias to control false discovery rates (for details, see code provided at zenodo under doi: 10.5281/zenodo.14039560).

### Profiling of soluble proteins by 2D DIGE and protein identification by MALDI-TOF-MS/MS

Extracts of soluble proteins of *Ds. variabilis* 3be13 were prepared, and 2D difference gel electrophoresis (DIGE) was performed essentially as reported previously (*94*). Cell pellets (approximately 100 mg wet weight) from three biological replicates per substrate condition were suspended in lysis buffer [7 M urea, 2 M thiourea, 30 mM tris/HCl, and 4% CHAPS (pH 8.5)], and cell breakage was achieved with the PlusOne sample grinding kit (GE Healthcare, Munich, Germany). The protein concentration was determined according to the method of Bradford (*95*). For minimal labeling, 200 picomoles of Lightning SciDye DIGE fluors (SERVA, Heidelberg, Germany) were used to label 50 μg of protein sample. Protein extracts of lactate-adapted cells served as the reference state and were labeled with Sci5. Protein extracts from the other 27 substrate adaptation conditions represented the test states and were each labeled with Sci3. The internal standard contained equal amounts of all test and the reference state(s) and was labeled with Sci2. Per gel, 50 μg each of the labeled reference state, test state, and internal standard were applied. To account for biological variation (*96*), three parallel gels were run with labeled protein extracts from three individual cultures (i.e., biological replicates) for each test state and reference. First dimension separation by isoelectric focusing (IEF) was conducted with 24-cm-long immobilized pH gradient (IPG) strips (pH 3 to 11 nonlinear; GE Healthcare) run in a Protean i12 system (Bio-Rad, Munich, Germany). The IEF program used was as follows: 50 V for 13 hours, 200 V for 1 hour, 1000 V for 1 hour, gradual gradient to 10,000 V within 2 hours, and 10,000 V until 70,000 Vhs were reached. The second dimension separation of proteins according to molecular size was done by SDS–polyacrylamide gel electrophoresis (12.5% gels, v/v) using an EttanDalttwelve system (GE Healthcare).

2D DIGE gels were digitalized directly after completion of electrophoresis with a charge-coupled device camera system (Intas Advanced 2D Imager, Intas Science Imaging Instruments GmbH, Göttingen, Germany) (*97*). Cropped gel images were analyzed with the DeCyder software (version 7.0; GE Healthcare) in two different work packages: one for aromatic (including cyclohexane carboxylate) and the other for aliphatic substrates. Parameters for spot detection were as described previously (*96*). All three biological replicates were included for the reference and each test state. Changes in the protein abundance of ≥ |1.5|-fold were regarded significant (*96*). Separate preparative colloidal Coomassie brilliant blue (cCBB)–stained gels were run (300 μg of protein load) to obtain sufficient amounts of protein for reliable mass spectrometric identification. Spots of interest were excised using the EXQuest spot cutter (Bio-Rad) from two cCBB-stained gels per analyzed substrate state and subsequently washed and tryptically digested as described (*98*).

Sample digests were spotted onto Anchorchip steel targets (Bruker Daltonik GmbH, Bremen, Germany) and analyzed with an UltrafleXtreme matrix-assisted laser desorption/ionization–time-of-flight (MALDI-TOF)/TOF mass spectrometer (Bruker Daltonik GmbH) as described (*98*). Peptide mass fingerprint (PMF) searches were performed with a Mascot server (version 2.3; Matrix Science, London, UK) against the translated genome of *Ds. variabilis* 3be13 with a mass tolerance of 25 parts per million (ppm). Five lift spectra were collected to confirm PMF identification, and three additional spectra were acquired of unassigned peaks applying feedback by the ProteinScape platform (version 3.1, Bruker Daltonik GmbH). In the case of failed PMF identification, eight lift spectra of suitable precursors were acquired. Mass spectrometry (MS)/MS searches were performed with a mass tolerance of 100 ppm. For both, MS and MS/MS searches, Mascot scores not meeting the 95% certainty criterion were not considered significant. A single miscleavage was allowed (enzyme trypsin), and carbamidomethyl (C) and oxidation (M) were set as fixed and variable modifications, respectively.

### Analyses of the membrane protein–enriched fractions

Total membrane protein fractions were prepared from two biological replicates per substrate condition and analyzed as previously reported (*98*). Essentially, cell extracts generated by means of a French Press (Sim-Aminco Ltd, Rochester, NY, USA) were treated with ice-cold carbonate and membrane proteins solubilized with SDS. Protein content was determined with the RC-DC assay (Bio-Rad), and protein separation was achieved using 12.5% SDS mini gels (10 cm by 7 cm; Bio-Rad). Each sample lane (10 μg of protein load) was divided into four gel slices, and each slice was cut into smaller pieces (about 1 mm^2^) before washing, reduction, alkylation, and tryptic digestion (*98*). The separation of peptides was performed with a nano-LC system. The nano-LC eluent was continuously analyzed by an online-coupled ion trap mass spectrometer (amaZon speed ETD, Bruker Daltonik GmbH) using the captive spray electrospray ion source (Bruker Daltonik GmbH). The instrument was operated in positive mode with a capillary current of 1.3 kV and drygas flow of 3 liter/min at 150°C. Active precursor exclusion was set for 0.2 min. Per full scan MS, 20 MS/MS spectra of the most intense masses were acquired. Protein identification was performed with ProteinScape as described above, including a mass tolerance of 0.3 Da for MS and 0.4 Da for MS/MS searches and applying a target decoy strategy (false discovery rate < 1%).

### Shotgun proteomic analyses

For shotgun analysis, cell pellets from three biological replicates per substrate condition were suspended in lysis buffer [7 M urea, 2 M thiourea, and 30 mM tris/HCl (pH 8.5)]. Cell breakage, removal of cell debris, reduction with dithiothreitol, alkylation with iodoacetamide, and tryptic in-solution digest were performed as previously described (*99*). Separation and detection of total peptide mixtures per sample were performed by nano-LC-ESI-MS/MS (see the “Analyses of the membrane protein–enriched fractions” section), except for applying a linear 240-min gradient. Protein identification was performed via the ProteinScape platform (see the “Profiling of soluble proteins by 2D DIGE and protein identification by MALDI-TOF-MS/MS” section).

### Analyses of the *Desulfobacteraceae* proteomic data

Comparative analyses of proteomic datasets of all six *Desulfobacteraceae* representatives (Figs. 3 to 5) was performed using combined peptide count data of the shotgun (i.e., soluble protein fraction) and the membrane protein–enriched fraction datasets, respectively. Nonredundant lists of detected proteins were created by including only the highest peptide counts of either the soluble or membrane fraction, and only detections in at least two replicates per condition were considered for subsequent analyses. Respective peptide count data were deposited at fairdomhub.org under DOI: 10.15490/fairdomhub.1.investigation.666.1 for *Ds. variabilis* 3be13 (this study), and protein data of the other strains were already reported (*27*–*30*). The shotgun mass spectrometry proteomics data have been deposited to the ProteomeXchange Consortium (http://proteomecentral.proteomexchange.org) via the PRIDE partner repository (*100*) with the dataset identifiers PXD053571, PXD053594, PXD053598, PXD053602, PXD053606, and PXD053610. MATLAB (version R2022b, The MathWorks Inc., Natick, MA, USA) was used for data analyses applying custom code. The comparison of the different proteomic datasets was performed using KEGG annotations of the individual proteins of all bacteria as assigned by EggNOG.

## Supplementary Materials

### The PDF file includes:

Supplementary Text Figs. S1 to S120 Tables S1 to S10 Legends for data S1 and S2 Legend for auxiliary reference list

### Other Supplementary Material for this manuscript includes the following:

Data S1 and S2

Auxiliary Reference List
