# Supplementary material for: Key role of Desulfobacteraceae in C/S cycles of marine sediments is based on congeneric catabolic-regulatory networks
Source: Sci Adv. 2025 Mar 7;11(10):eads5631. doi: 10.1126/sciadv.ads5631 (PMC11887813; doi:10.1126/sciadv.ads5631)
Supplement: Supplementary file 1 — Supplementary Text Figs. S1 to S120 Tables S1 to S10 Legends for data S1 and S2 Legend for auxiliary reference list [file sciadv.ads5631_sm.pdf]

Science Advances
AAAS

Supplementary Materials for

Key role of *Desulfobacteraceae* in C/S cycles of marine sediments is based on congeneric catabolic-regulatory networks

Lars Wöhlbrand *et al.*

Corresponding author: Lars Wöhlbrand, lars.woehlbrand@uol.de; Ralf Rabus, rabus@icbm.de

Sci. Adv. **11**, eads5631 (2025)
DOI: 10.1126/sciadv.ads5631

The PDF file includes:

Supplementary Text
Figs. S1 to S120
Tables S1 to S10
Legends for data S1 and S2
Legend for auxiliary reference list

Other Supplementary Material for this manuscript includes the following:

Data S1 and S2
Auxiliary Reference List

Supplementary Text

<u>*Desulfobacteraceae* contain distinct Qrc and Rnf complexes</u>

Besides the essential Qmo and Dsr redox-complexes, the quinone-reductase complex (QrcABCD) is part of the core genome of *Desulfobacteraceae* as well as the constitutive proteome being abundantly formed. This complex is related to alternative complex III, transferring electrons from reduced TpIc_3 (formed by periplasmic hydyrogen or formate oxidation) via QrcABCD to the menaquinone pool in *Desulfovibrio* species, with interaction of TpIc_3 and QrcA (*101, 102*). Indeed, the Qrc complex was predicted to form a redox loop with the Qmo complex, coupling periplasmic hydrogen (or formate) oxidation to cytoplasmic sulfate reduction (*20, 103*). While *Ds. variabilis* 3be13 apparently constitutively forms periplasmic hydrogenase HynAB, *Db. toluolica* Tol2 does not and *Dn. limicola* as well as *Dc. multivorans* completely lack hydrogenase genes, though abundantly forming QrcABCD during chemoorganotrophic growth (*27-29*). Hence, in case of *Desulfobacteraceae* a different and/or additional electron source has to be present. Notably, phylogenetic analyses revealed that QrcA and QrcB sequences of *Desulfobacteraceae* form distinct branches in the trees, which is more pronounced for QrcB (only 32% sequence identity of *Ds. variabilis* 3be13 as compared to *Dv. vulgaris*), while QrcCD subunits are rather similar between SRB (e.g. 53 % identity for QrcD Supplementary Information Fig. S17). *Desulfobacteraceae* QrcA sequences are ~20 residues longer as compare to *Dv. vulgaris*, containing an expanded cytochrome c_3 domain, that is also related to cytochrome c_7 (c_{551}), which was supposed to act as terminal reductase (*104*). QrcB contains an N-terminal 4Fe-4S domain, a central molybdopterin oxidoreductase and a C-terminal molybdopterin dinucleotide-binding domain (though no molybdenum is present), but its function is unknown at present and was suggested to possibly be only structural (*101, 103*). The different structure of *Desulfobacteraceae* QrcAB may indicate interaction with electron donors other than TpIc_3.

A second, abundantly formed redox complex in *Desulfobacteraaceae*, is a distinct version of RnfABCDEG ferredoxin:NAD$^+$-oxidoreductase complex. This complex was shown to reversibly couple oxidation of reduced ferredoxin (RnfB subunit) to reduction of NAD$^+$ (RnfC subunit) with concomitant generation of a Na$^+$-gradient across the cytoplasmic membrane (involving RnfEDAG) in *Acetobacterium woodii* (*105*). In SRB a second version of Rnf is present, comprising a markedly larger RnfB subunit (~700 aa vs. ~300 aa), that is encoded in all *Desulfobacteraceae* genomes (and also in *Da. baarsii* and *Dv. salexigens*). The RnfB and RnfC subunits of the latter comprise a different domain structure as compared to the *Ac. woodii* types and respective sequences form distinct branches in the phylogenetic tree (Supplementary Information Fig. S18), all other subunits (RnfCDEG) are very similar (*106*). The N-terminus of the large RnfB variant (i.e. residues 1-200) is highly similar to the small one (40 % identity, 53 % positives), containing 4Fe-4S and ferredoxin domains. The remaining 500 specific residues (absent in Rnf1) accommodate a pyridine nucleotide-disulfide oxidoreductase, NAD-binding domain, indicating interaction with NAD$^+$/ NADH + H$^+$ in addition to ferredoxin. In case of RnfC, the central NADH-ubiquinone oxidoreductase, FMN-binding as well as the N-terminal RnfC barrel sandwich domain (sometimes only the first) are absent in the Rnf2 type, challenging a function in NAD$^+$ reduction as known from *A. woodii*. Notably, some SRB genomes contain genes for both versions and form respective proteins, e.g. *Dt. autotrophicum* HRM2 and *Ds. variabilis* 3be13 (Rnf2 and Rnf3, respectively, in case of the latter). Independent of the type of Rnf complex, respective gene clusters of SRB encode a cytochrome c_3 (e.g. Dvar_58310 in case of *Ds. variabilis* 3be13), that is not present in *Ac. woodii* or *Rhodobacter capsulatus*, which may represent an additional electron entry or exit point (*107*). In case of SRB harbouring both, the Rnf1 and Rnf2 type gene cluster, only the *Desulfobacteraceae* Rnf2 type clusters encode this cytochrome.

Taken together, it seems feasible that oxidation of ferredoxin (and maybe also NADH + H$^+$) by the Rnf complex may not only contribute to Na$^+$-gradient formation as described for *Ac. woodii* (*105*), but additionally reduce the associated cytochrome c_3. The latter may act as electron shuttle to the Qrc complex, contributing to reducing the menaquine pool and, hence, sulfate reduction via QmoABC. Non-detection of these cytochromes in the proteomic studies of *Desulfobacteraceae* is most likely due to their small size and hydrophobicity, rendering such proteins hardly detectable by LC-MS. In addition to the abundant formation of Qrc and Rnf2 in *Desulfobacteraceae*, this hypothesis is supported by experimental evidence obtained

with mutants of *Desulfovibrio* sp.: (i) TpIc_3 transposon mutants of *Dv. alaskensis* G20 were unable to grow with hydrogen or formate, but growth with lactate was not effected (*102, 108*), (ii) a *qrcA* mutant did only grow poorly with pyruvate and sulfate while pyruvate fermentation was not effected (*109*), (iii) mutants of the Qrc and Rnf complexes revealed significant deficits during respiratory growth (*110*), and (iv) mutants in Rnf-subunits grew barely or not with sulfate and substrates that do not allow for substrate-level phosphorylation (e.g. malate or fumarate)(*109*).

Details on the transporter complement of *Desulfobacteraceae*

The genomic transport potential (transportome) of the studied six SRB was inferred by the Transporter Classification Database (TCDB)(*32*) that applies a five-tier system based on structure and functions of the transport systems. Five well-defined categories (1) channels/pores, (2) electrochemical potential-driven transporters (secondary carriers), (3) primary active transporters (energy-driven, e.g. by ATP hydrolysis), (4) group translocators (substrate-modifying) and (5) transmembrane electron carriers (transferring electrons from one side of a membrane to the oher) are complemented by two less-well defined ones: (8) auxiliary transport proteins and (9) putative or incompletely characterized transport systems. Transporter systematics allow for classification from subclass to the actual transport system.

The genome of *Ds. variabilis* 3be13 encodes 979 transport systems, the others pronouncedly less (445 – 712; Fig. 5a, Supplementary Information Fig. S20 and Table S9). In all cases, the most abundant classes are secondary carriers and active transporters, accounting for 58.9% – 73.2% of all transport proteins. In general, the relative share of the different transporter subclasses is rather similar in the studied SRB. In *Ds. variabilis* 3be13, 45.5% of the encoded transport proteins were detected in this study, similar to the other SRB (35.5% - 45.7%), only *Dc. multivorans* 1be1 exceeding this range with 66.2% (Fig 5a, Supplementary Information Fig 20). Notably, subclass-distribution of formed proteins mostly reflects the genomic potential, only a slightly increased share of active transporters and channels, and a bit reduced share of secondary carriers is observed (Fig. 5a). Globally comparing the six SRB, the major sub groups (i.e. 1B, 2A, 3A and 3D) reveal only slight differences in the respective relative share, while more pronounced variation is evident in groups encompassing only a low number of proteins. The different transporter types may be involved in transport of similar substrates. Considering the substrate spectrum of the transport proteins of *Ds. variabilis* 3be13, the majority is involved in transport of amino acids, drugs (mainly antibiotics), inorganic cations (mainly electron, sulfate and phosphate), inorganic anions (mainly protons and sodium), and unknown substrates (Fig. 5b). Similar to transporter subclasses, distribution of formed proteins resembles the genomic potential. While transporters of more general substrates show rather stable abundance profiles (e.g. sulfate-importing Dvar_38430), specific transporters revealed a more substrate-dependent formation, e.g. DctPQM4 involved in 4-hydroxy-/phenylacetate uptake, reflecting the regulatory pattern observed for cytoplasmic proteins involved in general metabolism and specific catabolic routes, respectively.

Supplementary Figures

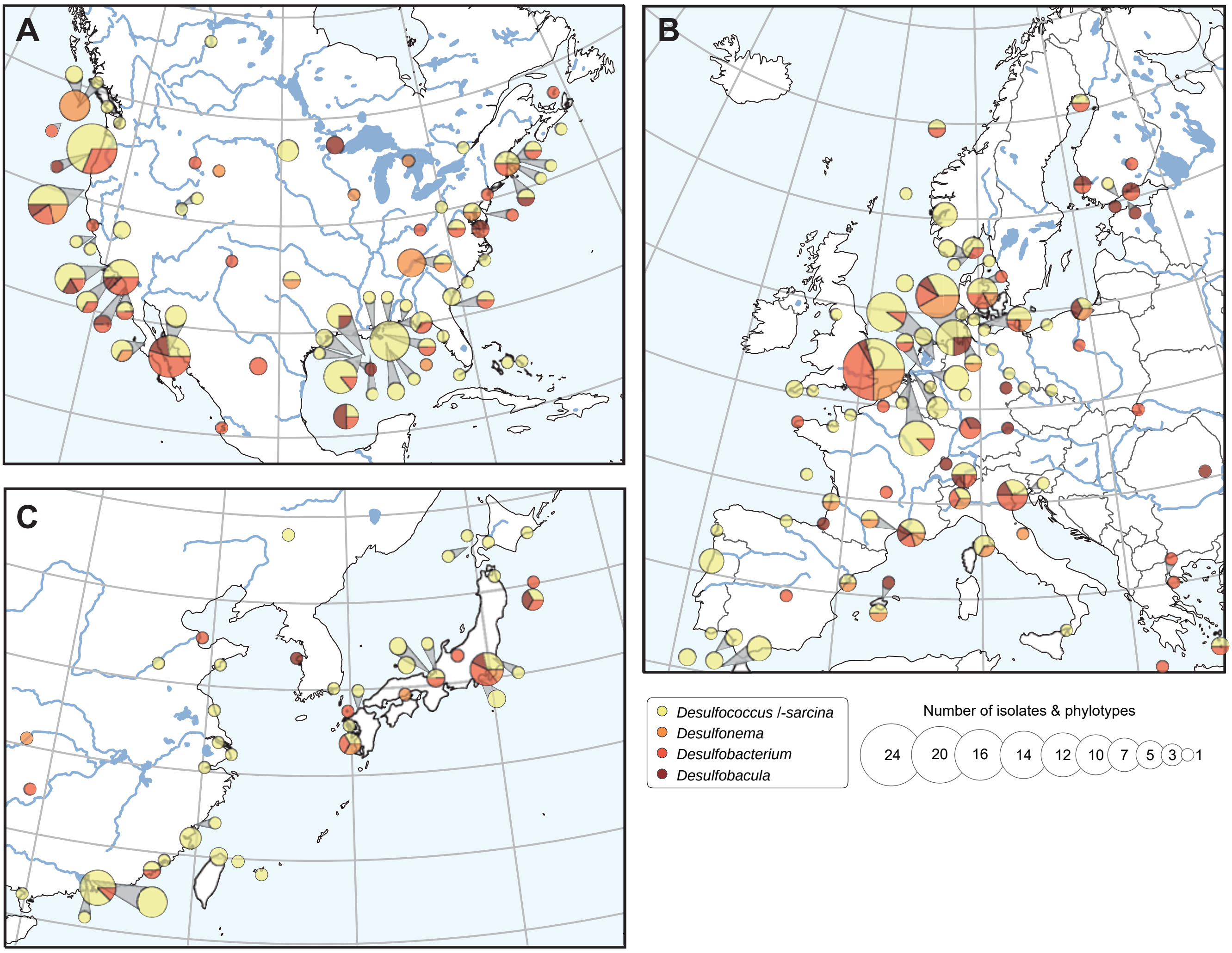

Fig. S1: Occurrence of *Desulfobacteraceae* species isolates and/or phylotypes. Densely spaced sites are aggregated into clusters and represented as circles. The circle area indicates the number of occurences/sites in a cluster and the pie sector area indicates the abundance of a given genus in a cluster. **A**, North America; **B**, Europe; **C**, Asia. Color code: *Desulfococcus/-sarcina* (yellow), *Desulfonema* (orange), *Desulfobacterium* (red), and *Desulfobacula* (dark red).

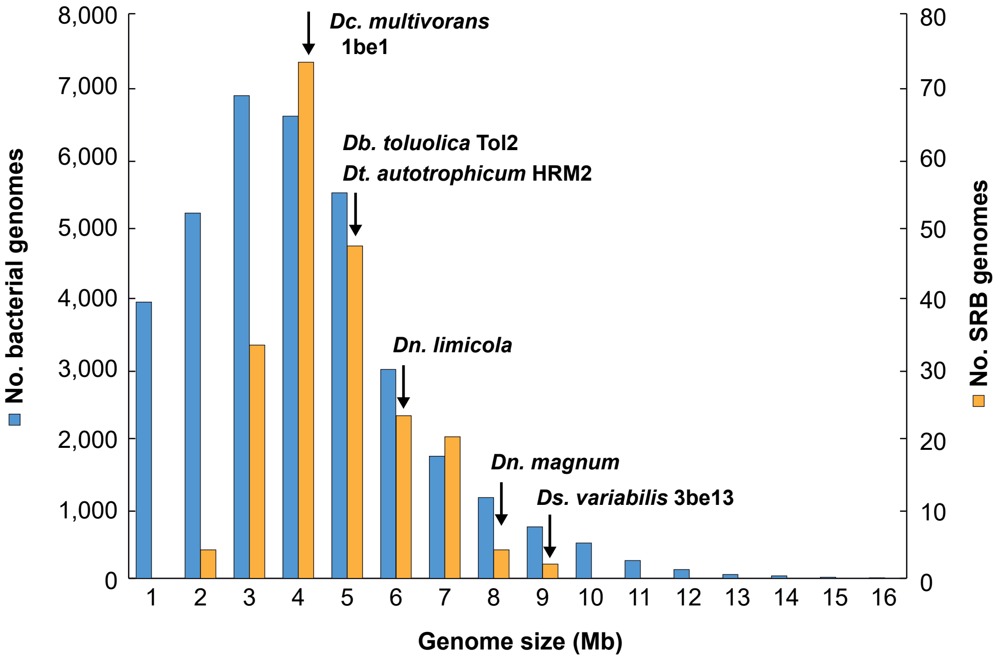

Fig. S2: Compilation of genome sizes of currently sequenced bacteria (blue) and sulfate-reducing bacteria (orange) according to NCBI genomes (Mai 2023). Names of proteogenomically studied *Desulfobacteraceae* spp. are indicated at their respective genome size.

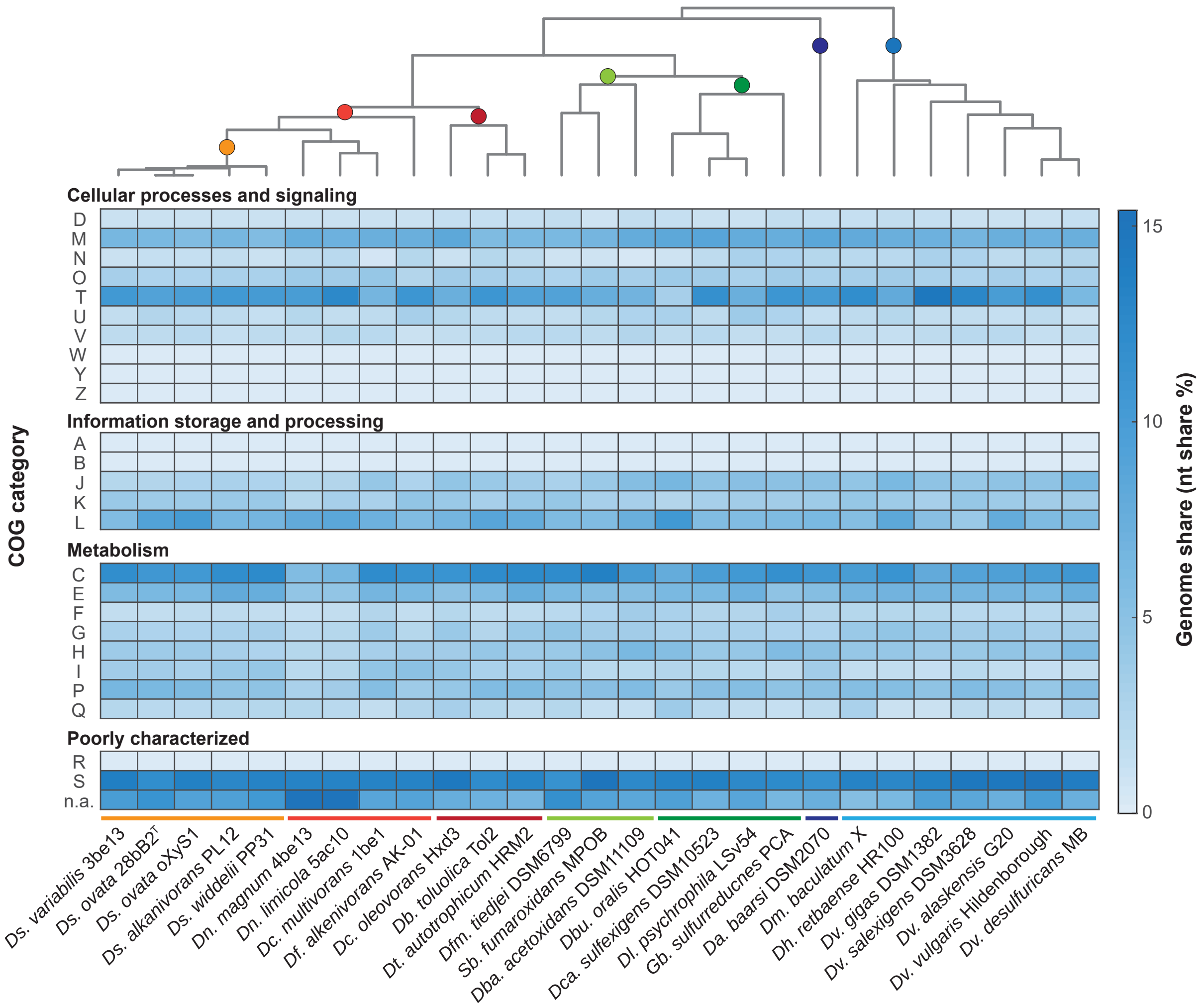

Fig. S3: Relative genome share of coding sequence (CDS) across the cluster of orthologous groups (COG) for completely sequenced *Desulfo-bacteraceae* genomes and selected other sulfate-reducing bacteria. Color-code is based on the indicated 16S rRNA-based phylogeny of the selected SRB (top). Genus abbreviations are as given in legend to Fig. 2.

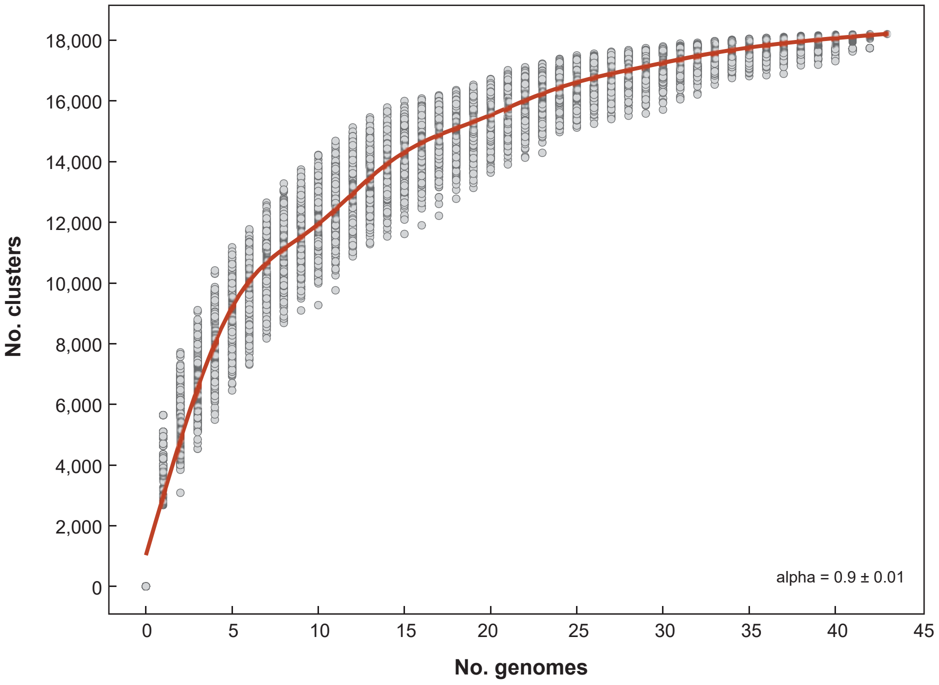

Fig. S4: Rarefaction curve showing the cumulative number of clusters of orthologous genes for every new genome added. To prevent influence from the order of inclusion, 1,000 permutations were conducted. The flattening of the curve shows the saturation of the pangenome, with the inclusion of more genomes returning diminishing numbers of new clusters. The "openness" of the pangenome predicts that novel genes will be discovered by increasing the number of genomes included. However, the saturation of the curve suggests that the collection is representative of the vast majority of *Desulfobacteraceae* gene repertoire.

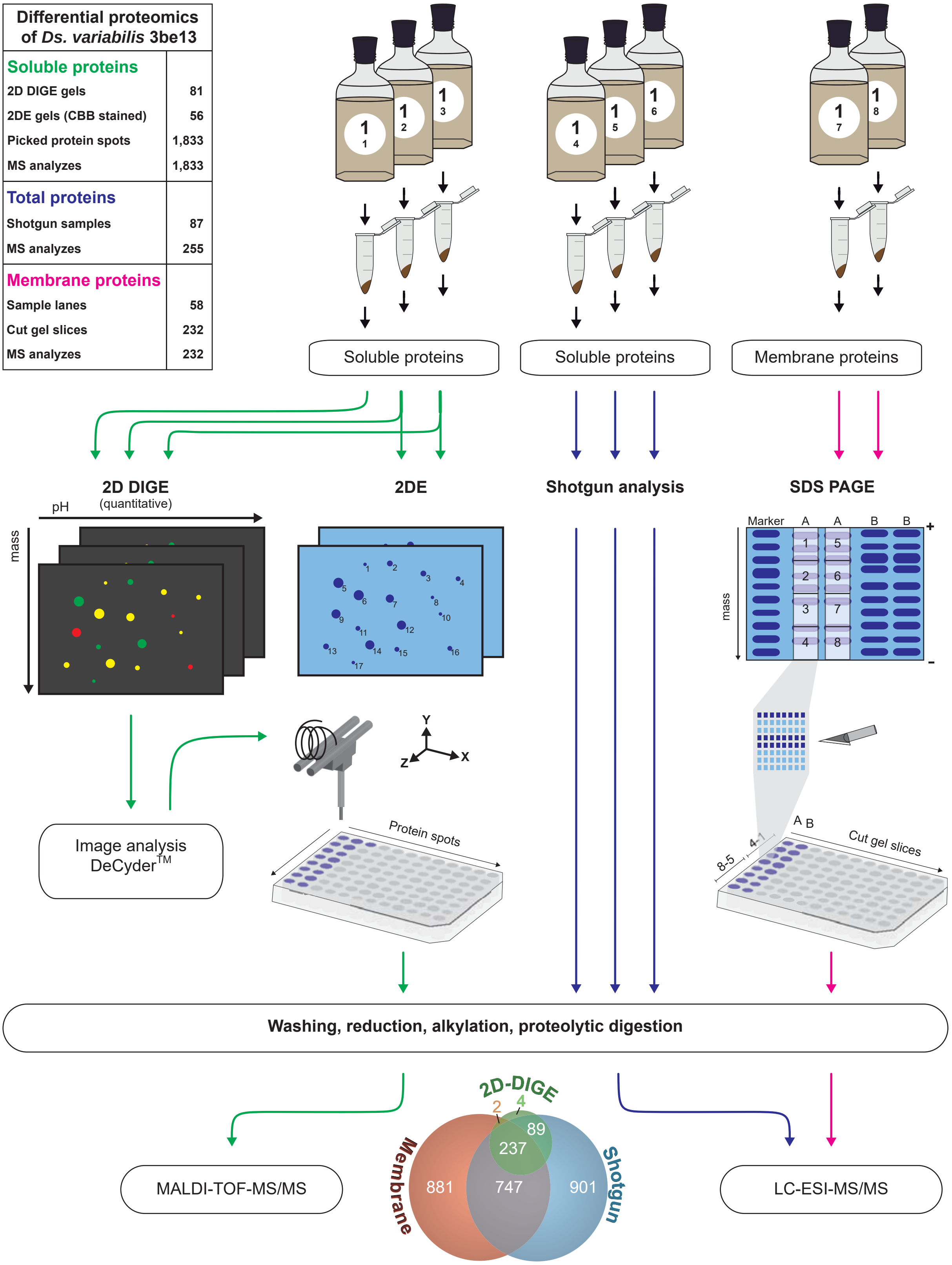

Fig. S5: Scheme of proteomic analyses workflow of *Desufosarcina varibabilis* 3be13 samples that is also representative for the other 5 studied *Desulfobacteraceae*. Replicate cultures of substrate-adapted cells were harvested and subjected to appropriate protein extraction procedures to prepare soluble and membrane protein fractions, respectively, for gel-based or gel-free analysis of the respective protein complements coupled to MS-based protein identification. The Venn diagram illustrates complementarity of the applied methods and the inlet summarizes sample quanta per analysis method.

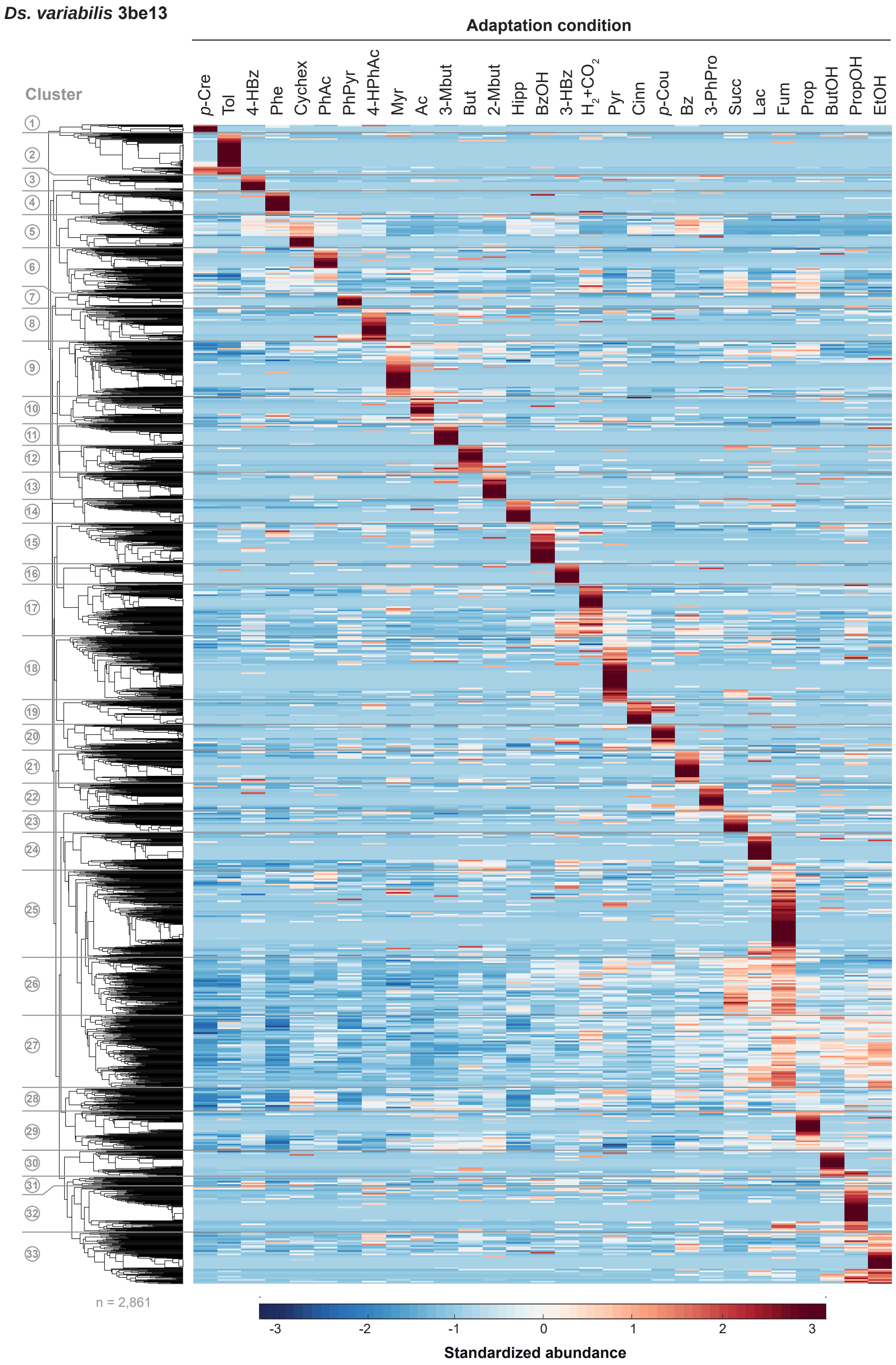

Fig. S6: Clustering of standardized protein abundances of all detected proteins of *Ds. variabilis* 3be13 across the 29 studied adaptation conditions. Detailed representations of the 33 clusters are provided in Supporting Information Figs. 26-58. Substrate abbreviations: Ac, acetate; But, butanoate; ButOH, *n*-butanol; Bz, benzoate; BzOH, benzyl alcohol; Cinn, cinnamate; *p*-Cou, *p*-coumarate; *p*-Cre, *p*-cresol; Cychex, cyclohexane carboxylate; EtOH, ethanol; Fum, fumarate; H_2 + CO_2, lithoautotrophic growth; 3-HBz, 3-hydroxybenzoate; 4-HBz, 4-hydroxybenzoate; Hipp, hippurate; 4-HPhAc, 4-hydroxyphenylacetate; Lac, lactate; 2-Mbut, 2-methylbutanoate; 3-Mbut, 3-methylbutanoate; Myr, myristinate; PhAc, phenylacetate; Phe, phenol; 3-PhPro, 3-phenylpropanoate; PhPyr, phenylpyruvate; Prop, propanoate; PropOH, *n*-propanol; Pyr, pyruvate; Succ, succinate; Tol, toluene.

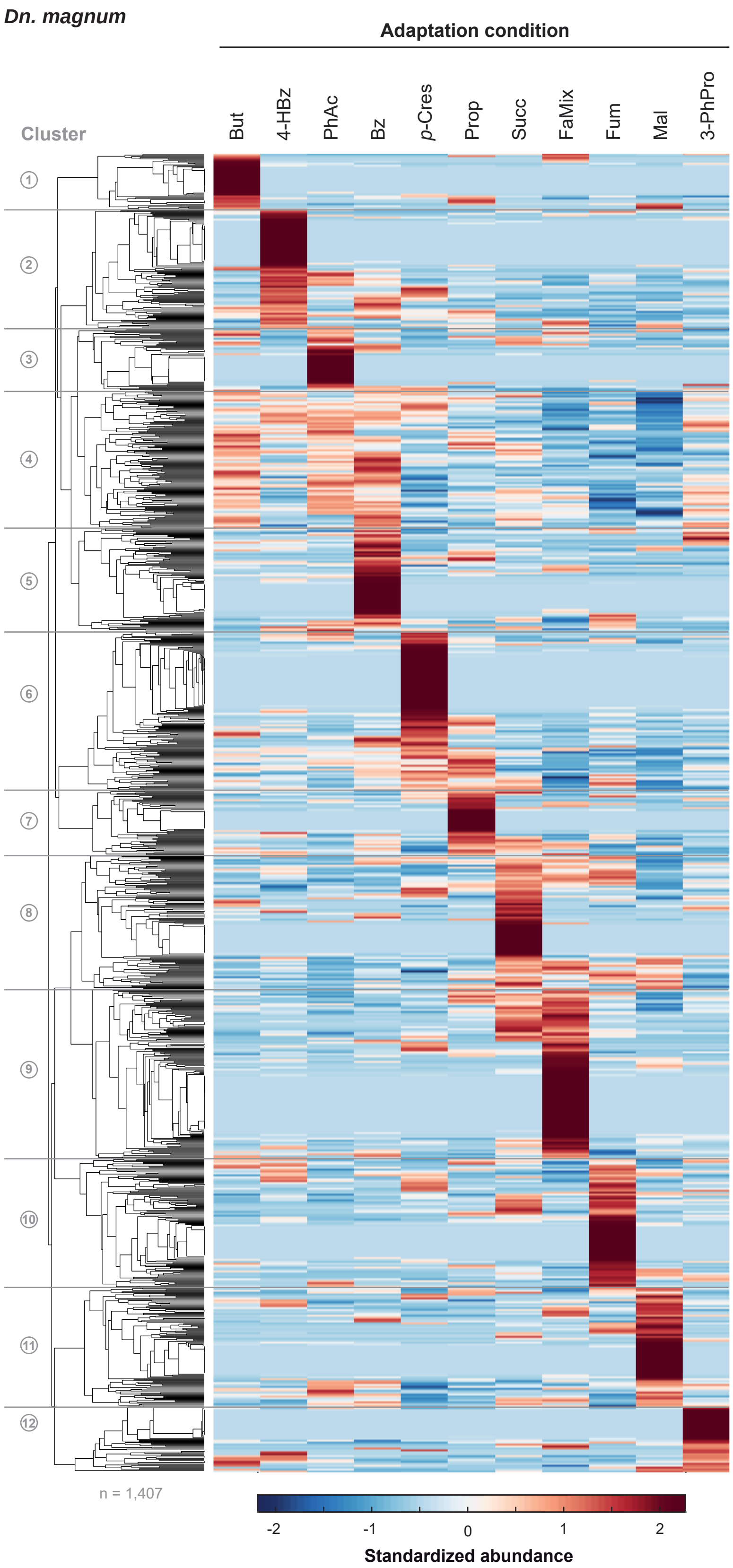

Fig. S7: Clustering of standardized protein abundances of all detected proteins of *Desulfonema magnum* across the 11 studied substrate adaptation conditions. Detailed representations of the 12 clusters are provided in Supplementary Figs. S59-S71. Substrate abbreviations: But, butanoate; Bz, benzoate; *p*-Cre, *p*-cresol; FaMix, fatty acid mix; Fum, fumarate; 4-HBz, 4-hydroxybenzoate; Mal, malate; PhAc, phenylacetate; 3-PhPro, 3-phenylpropanoate; Prop, propanoate; Succ, succinate.

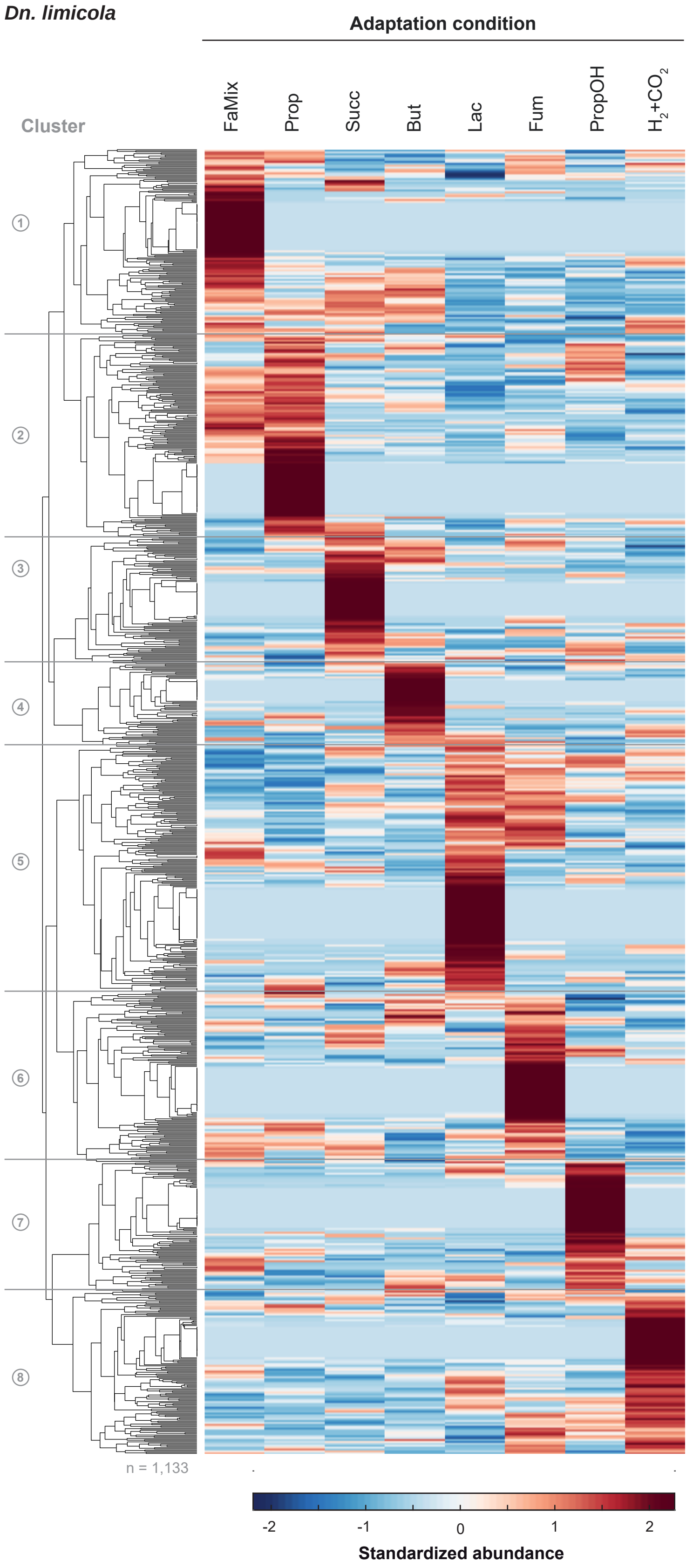

Fig. S8: Clustering of standardized protein abundances of all detected proteins of *Desulfonema limicola* across the 11 studied substrate adaptation conditions. Detailed representations of the 8 clusters are provided in Supplementary Figs. S72-S80. Substrate abbreviations: But, butanoate; FaMix, fatty acid mix; Fum, fumarate; $H_2 + CO_2$, lithoautotrophic growth; Lac, lactate; Prop, propanoate; PropOH, *n*-propanol; Succ, succinate.

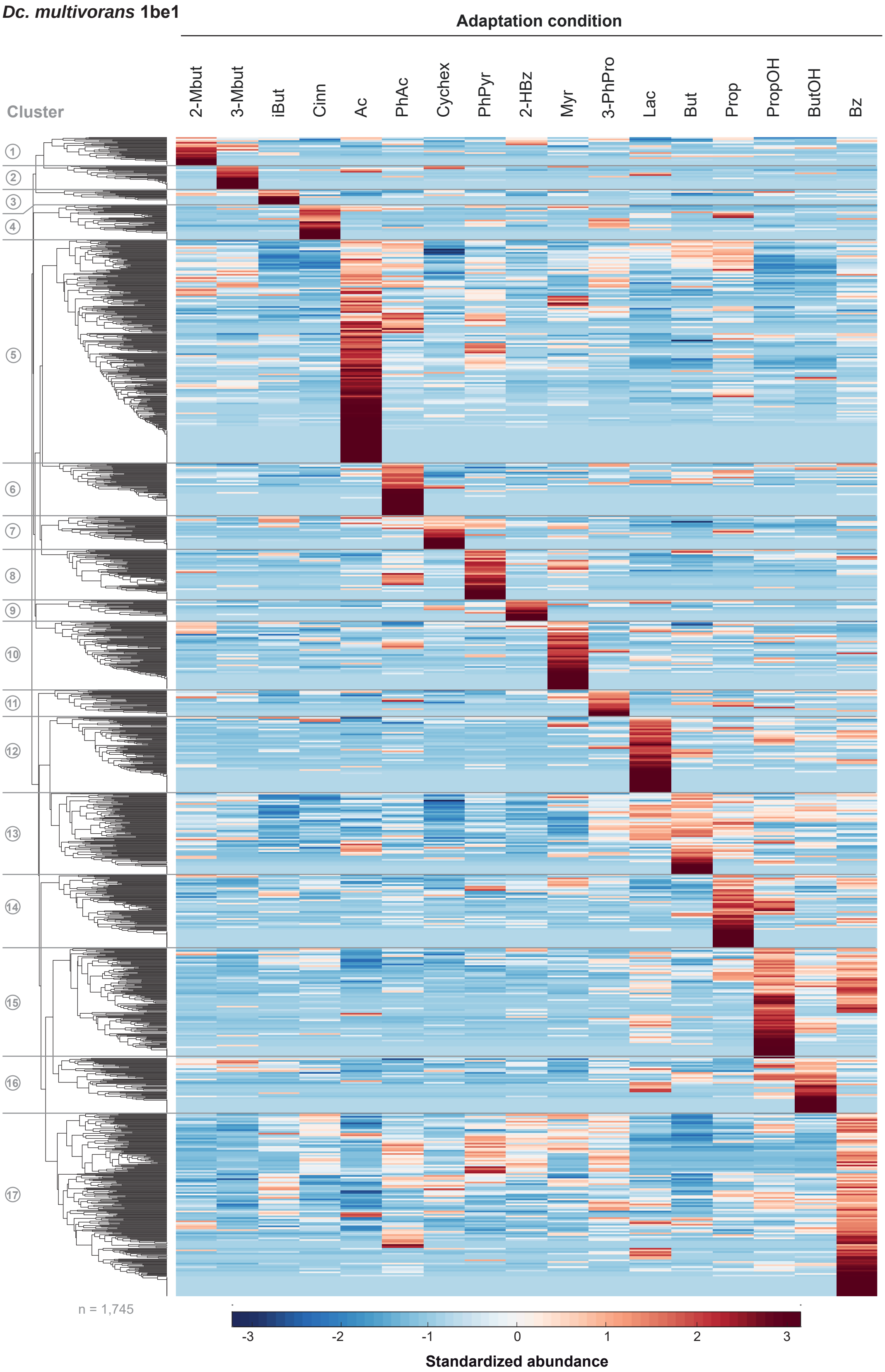

Fig. S9: Clustering of standardized protein abundances of all detected proteins of *Desulfococcus multivorans* 1be1 across the 17 studied substrate adaptation conditions. Detailed representations of the 17 clusters are provided in Supplementary Figs. S81-S99. Substrate abbreviations: Ac, acetate; But, butanoate; ButOH, *n*-butanol; iBut, iso-butuanol; Bz, benzoate; Cinn, cinnamate; Cychex, cyclohexane carboxylate; 2-HBz, 2-hydroxybenzoate; Lac, lactate; 2-Mbut, 2-methylbutanoate; 3-Mbut, 3-methylbutanoate; Myr, myristinate; PhAc, phenylacetate; 3-PhPro, 3-phenylpropanoate; PhPyr, phenylpyruvate; Prop, propanoate; PropOH, *n*-propanol; .

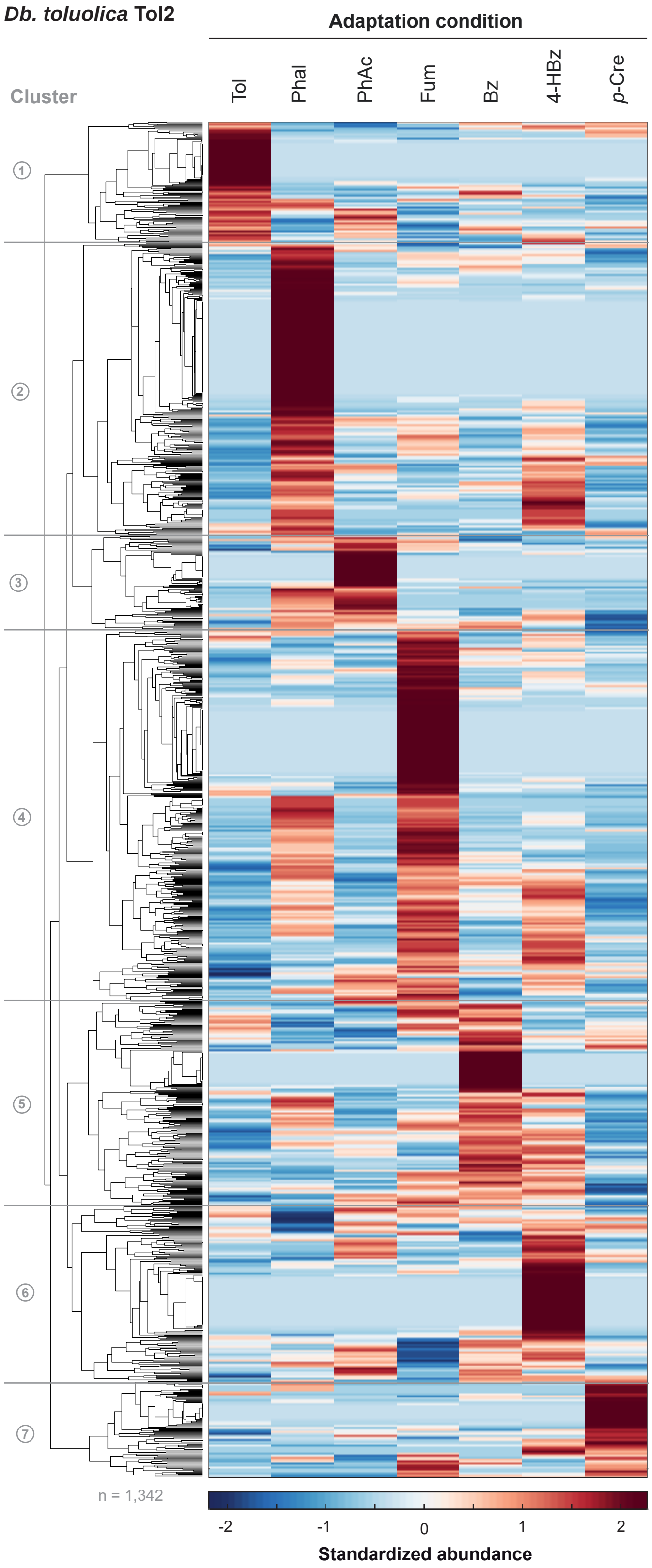

Fig. S10: Clustering of standardized protein abundances of all detected proteins of *Desulfobacula toluolica* Tol2 across the 7 studied substrate adaptation conditions. Detailed representations of the 7 clusters are provided in Supplementary Figs. S100-S109. Substrate abbreviations: Bz, benzoate; *p*-Cre, *p*-cresol; Fum, fumarate; 4-HBz, 4-hydroxybenzoate; PhAc, phenylacetate; Phal, phenylalanine; Tol, toluene.

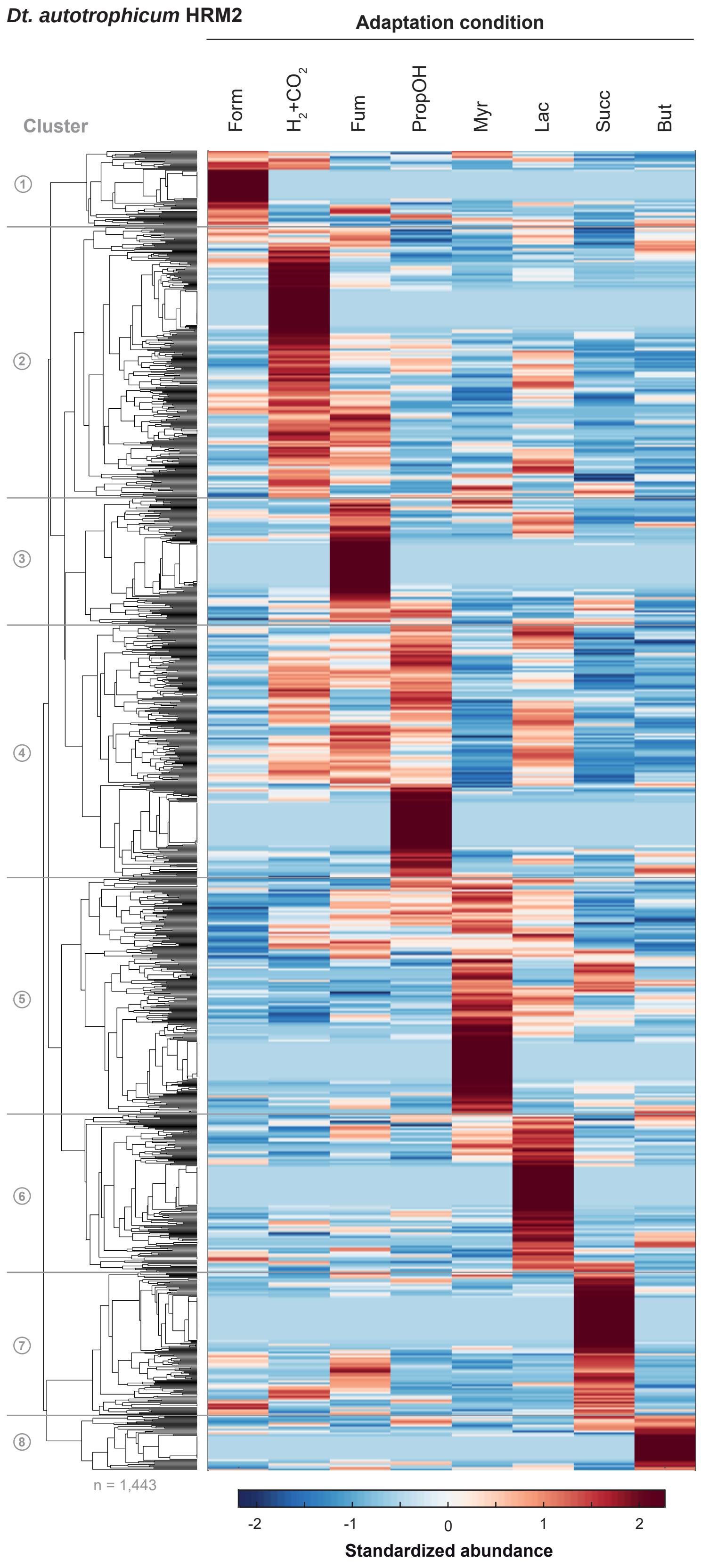

Fig. S11: Clustering of standardized protein abundances of all detected proteins of *Desulfobacterium autotrophicum* HRM2 across the 8 studied substrate adaptation conditions. Detailed representations of the 8 clusters are provided in Supplementary Figs. S110-S120. Substrate abbreviations: But, butanoate; Form, formate; Fum, fumarate; $H_2 + CO_2$, lithoautotrophic growth; Lac, lactate; Myr, myristinate; PropOH, *n*-propanol; Succ, succinate.

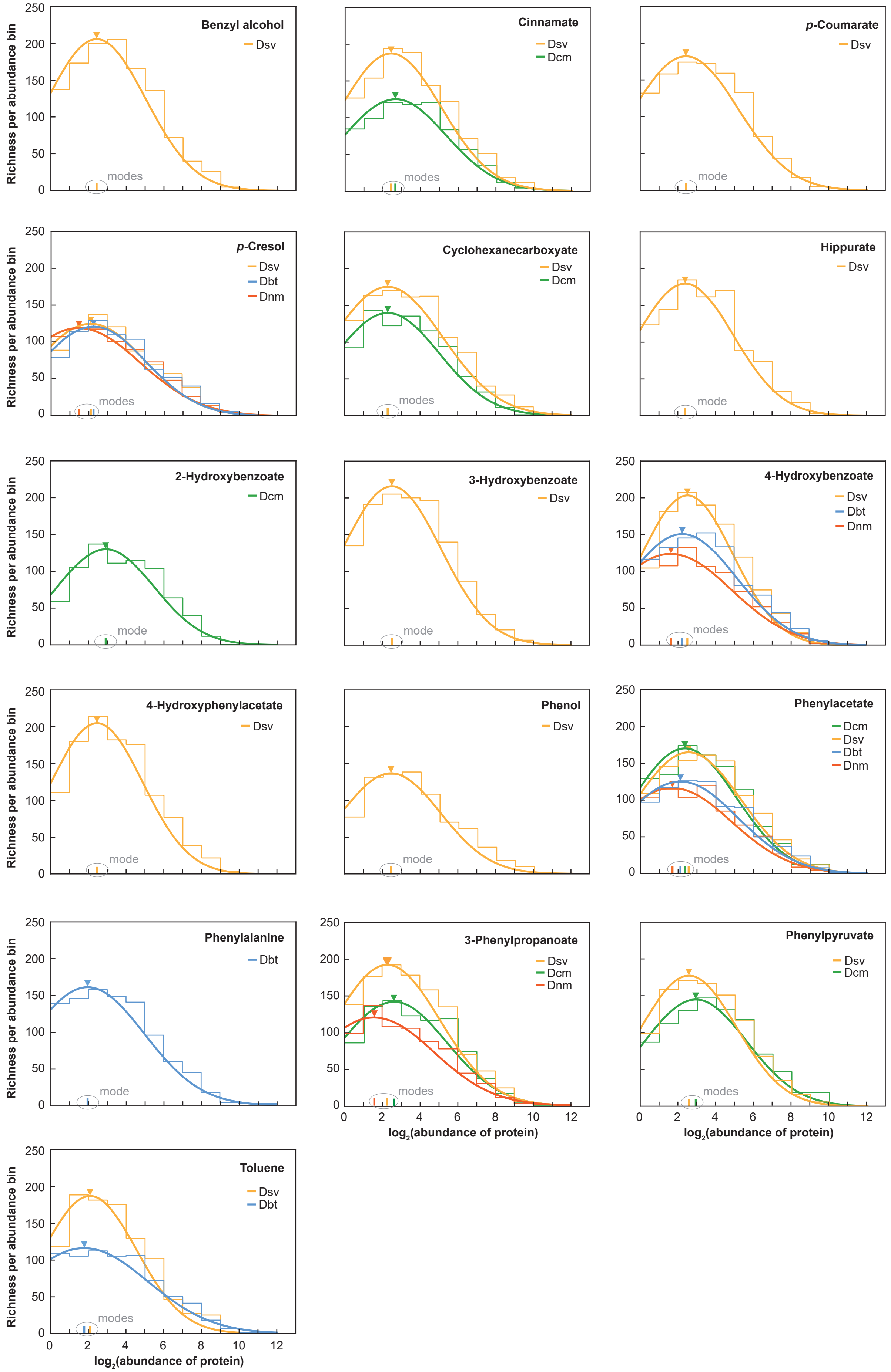

Fig. S12: Histograms (staircases; log bin size 1) of protein abundance for aromatic growth substrates per strain (in alphabetical order). All abundance distributions are log normally distributed (fitted smooth lines); *Ds. variabilis* 3be13 (Dsv), *Dc. multivorans* 1be1 (Dcm), *Dn. limicola* (Dnl), *Dn. magnum* (Dnm), *Db. toluolica* Tol2 (Dbt) and *Dt. autotrophicum* HRM2 (Dta). The peak of the distribution (triangles) determines the abundance mode (vertical bars on abscissa), i.e. the abundance value shared by the largest number of different proteins.

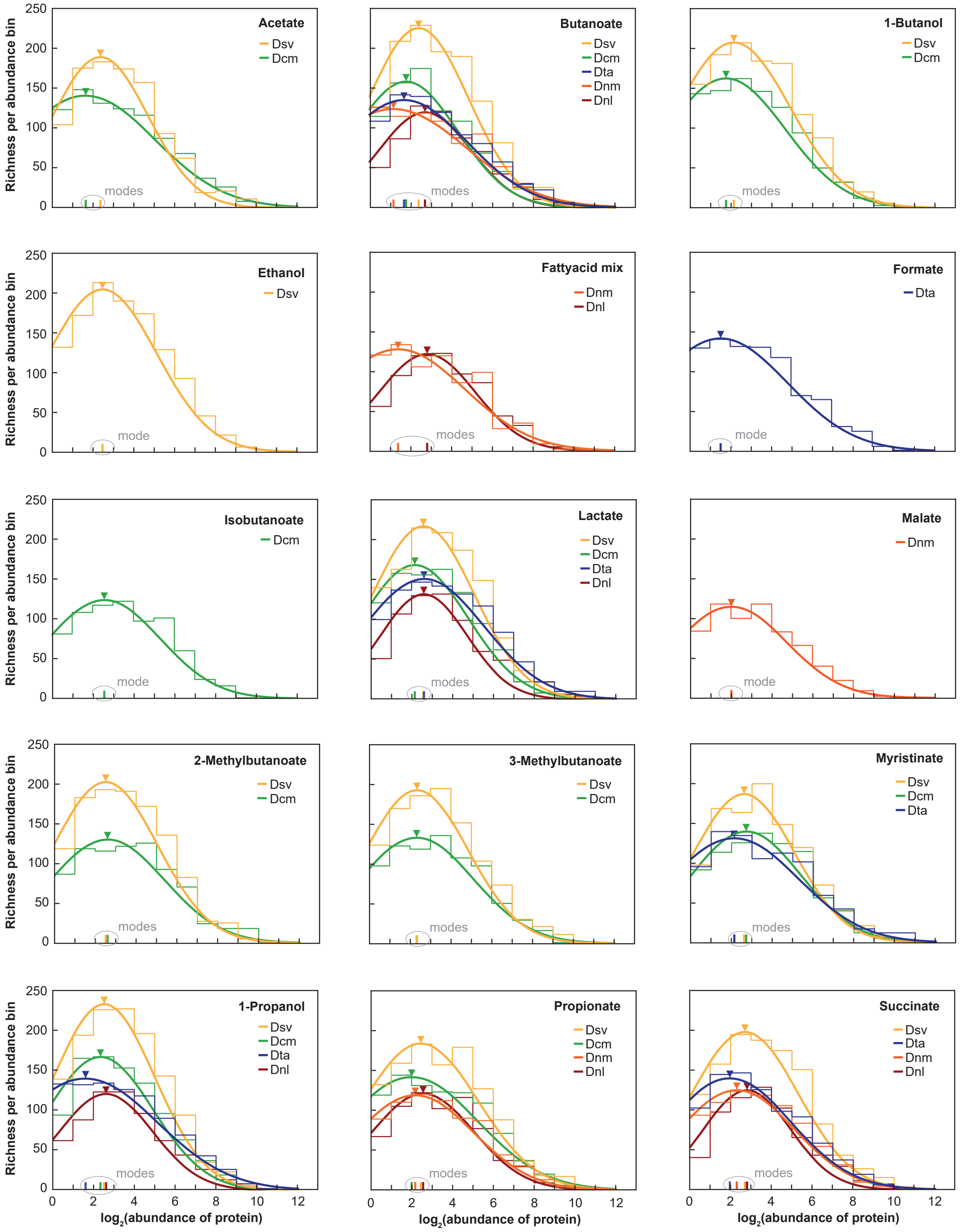

Fig. S13: Histograms (staircases; log bin size 1) of protein abundance for aliphatic growth substrates per strain (in alphabetical order). All abundance distributions are log normally distributed (fitted smooth lines); *Ds. variabilis* 3be13 (Dsv), *Dc. multivorans* 1be1 (Dcm), *Dn. limicola* (Dnl), *Dn. magnum* (Dnm), *Db. toluolica* Tol2 (Dbt) and *Dt. autotrophicum* HRM2 (Dta). The peak of the distribution (triangles) determines the abundance mode (vertical bars on abscissa), i.e. the abundance value shared by the largest number of different proteins.

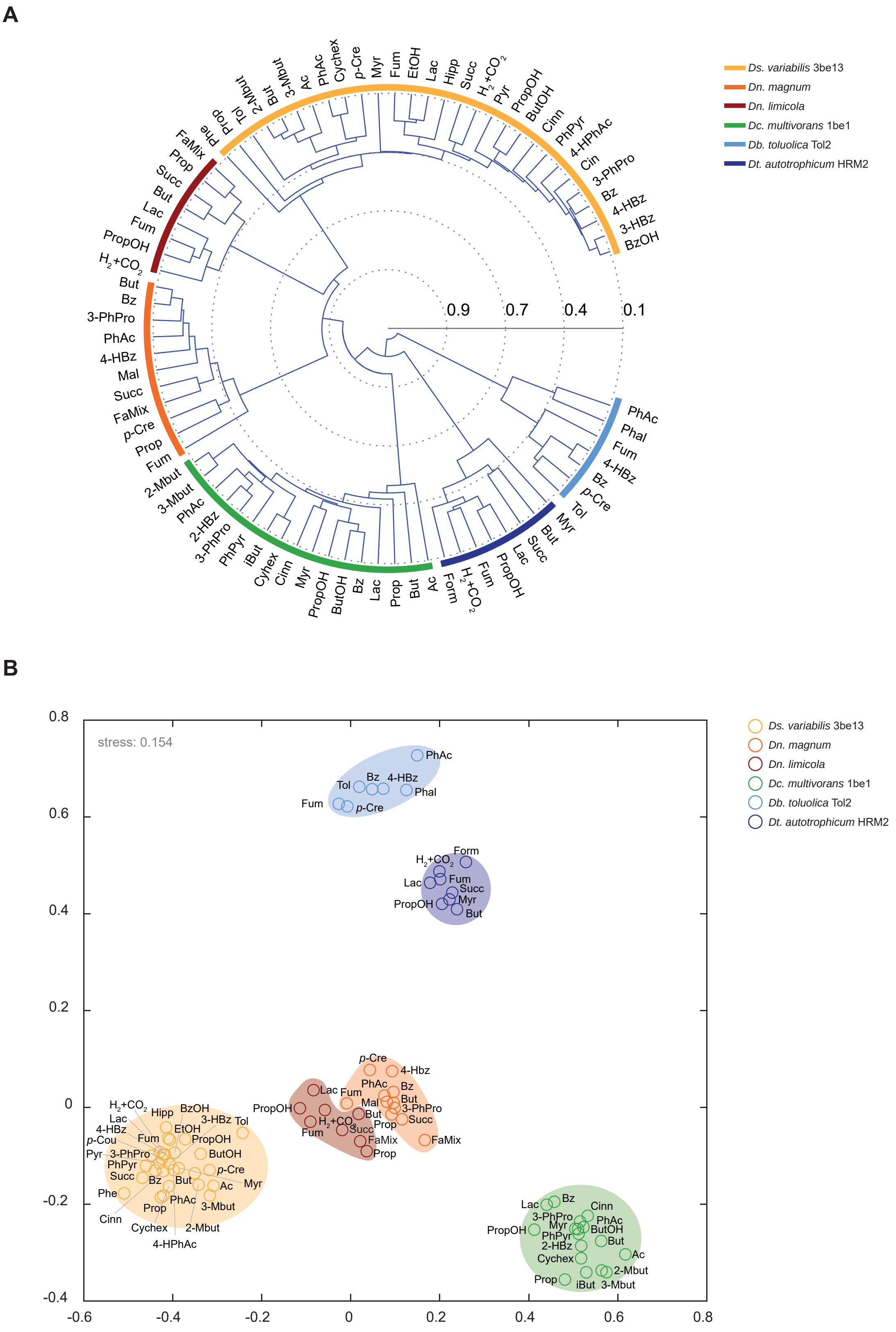

Fig. S14: Interspecies comparison of condition-specific proteomes based on KEGG orthology and pathways. We used the relative abundance of proteins per strain and substrate condition to account for the differently sized datasets. Only proteins with assigned KEGG category and present in all conditions per strain were included in the analysis. **A**, Clustering tree (euclidean distance metric, linkage average) covering 80 different conditions of the six proteomically studied SRB strains. **B**, Classical multidimensional scaling plot (cityblock distance metric). Substrate conditions are indicated, and organisms are color coded. The corresponding Shepard plot is provided in fig. S15.

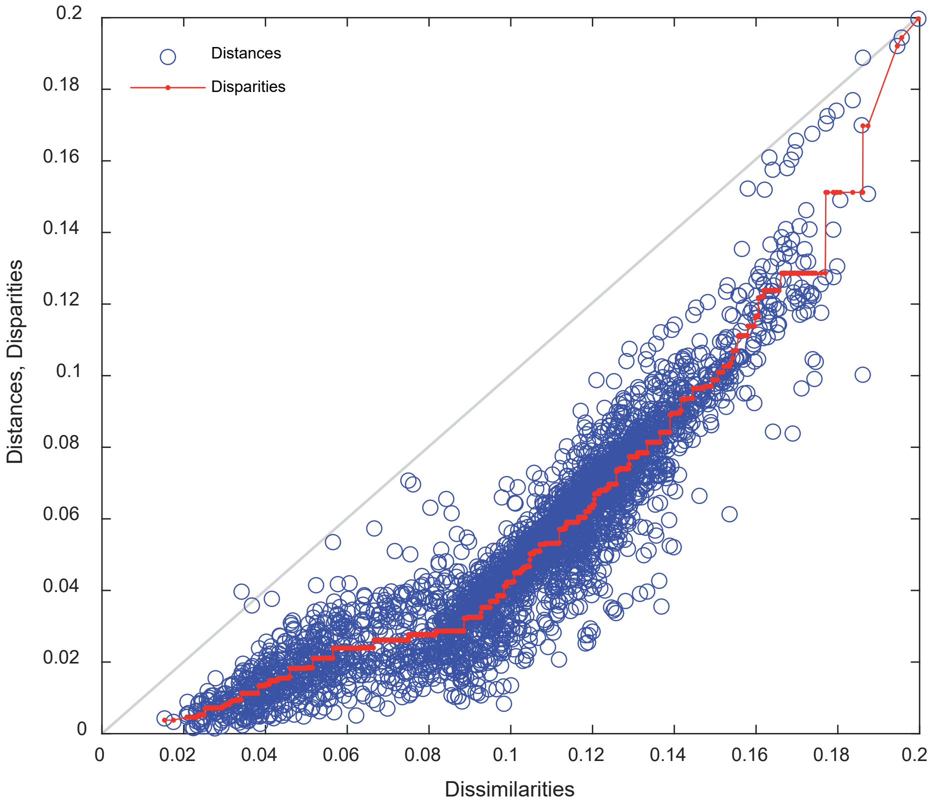

Fig. S15: Shepard diagram of data presented in Fig. S14B, relating dissimilarities with distances (blue circles) and disparities (red dots and line), respectively. Dissimilarities and distances show a piecewise linear dependency with high correlation (e.g. for dissimilarities in interval [0.02, 0.06], [0.06, 0.09], and [0.09, 0.17]).

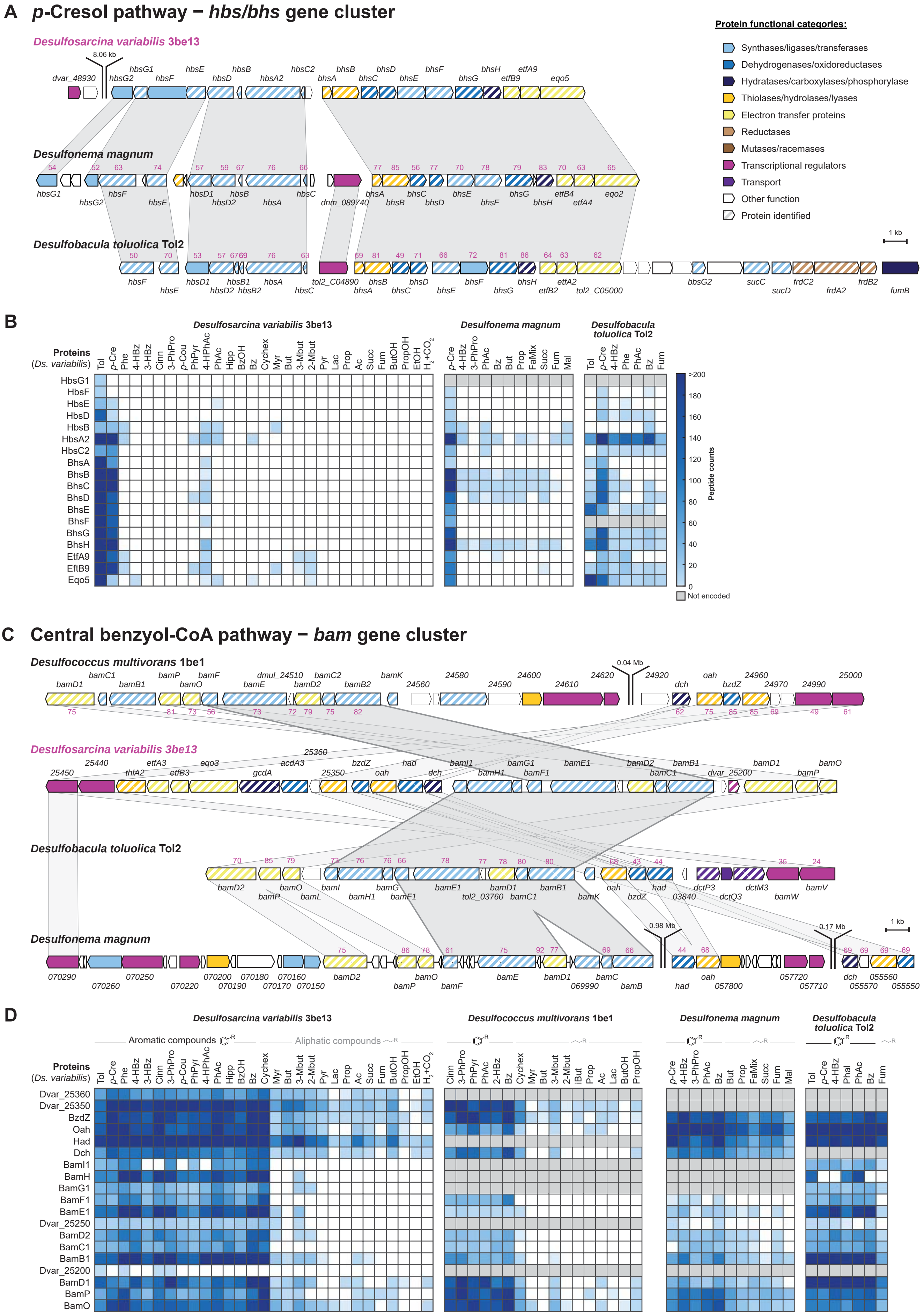

Fig. S16: Catabolic gene modules of *Desulfobacteraceae*. A, Gene clusters encoding proteins involved in anaerobic *p*-cresol degradation of *Ds. variabilis* 3be13, *Dn. magnum*, and *Db. toluolica* Tol2. Amino acid sequence identities as compared to *Ds. variabilis* 3be13 are given in violet. Protein functions are color coded. **B,** Heatmap of abundance of identified proteins related to the *p*-cresol pathway across respective substrate adaptation conditions per strain. **C**, Gene clusters encoding proteins involved in central anaerobic benzoyl-CoA pathway of *Ds. variabilis* 3be13, *Dc. multivorans* 1be1, *Dn. magnum*, and *Db. toluolica* Tol2. Amino acid sequence identities, as compared to *Ds. variabilis* 3be13, are shown in violet. **D**, Heatmap of abundances of identified proteins of the benzoyl-CoA pathway across respective substrate adaptation conditions per strain.

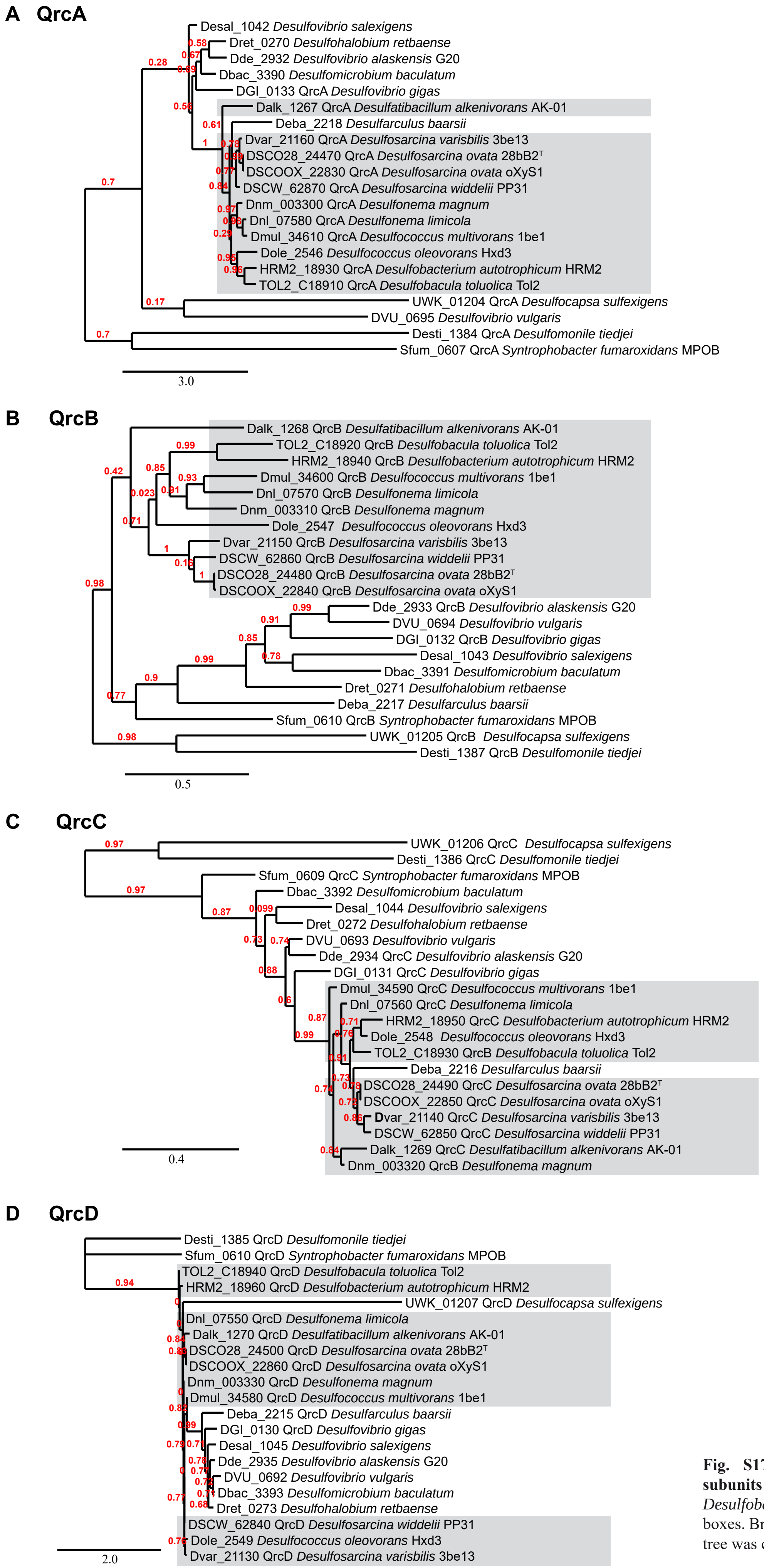

Fig. S17: Phylogenetic tree of Qrc complex subunits (A-D) of selected SRB. Qrc sequences of *Desulfobacteraceae* members are highlighted by grey boxes. Branch support values are indicated in red. The tree was constructed using phylogeny.fr (*111*).

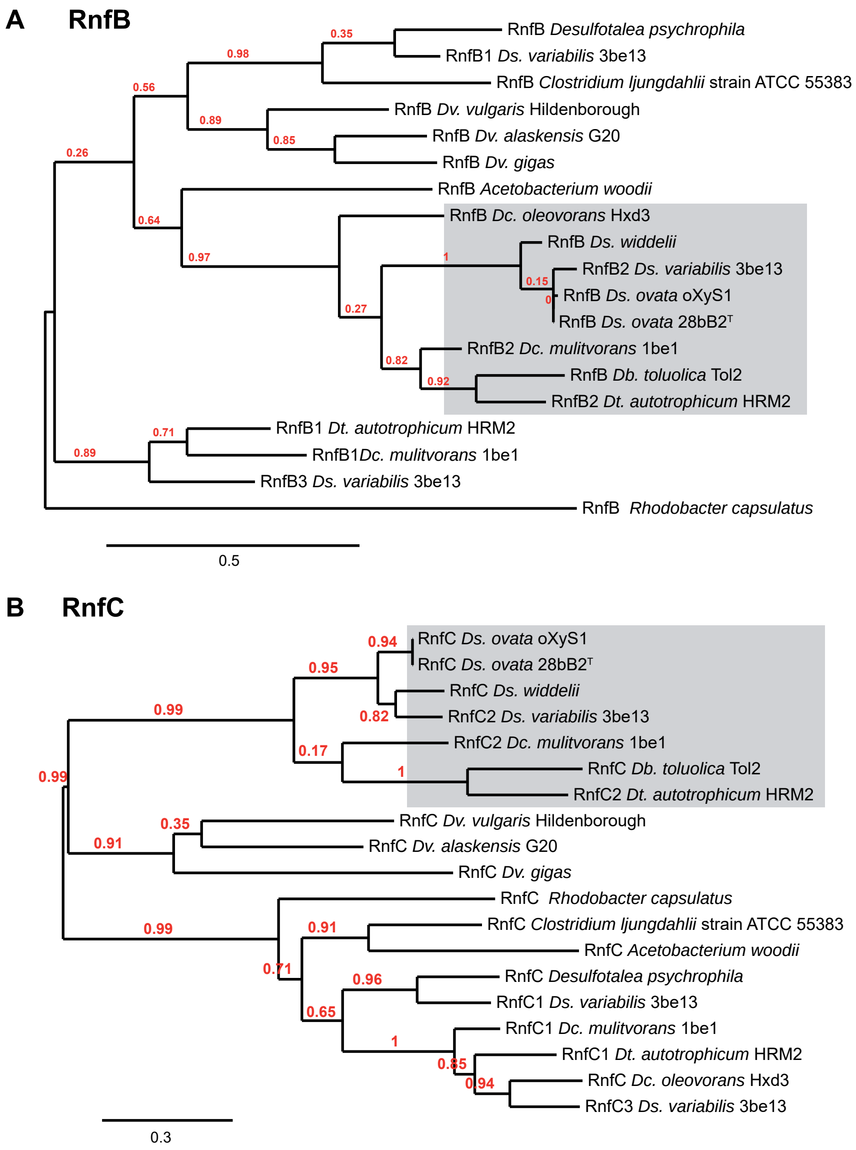

Fig. S18: Phylogenetic tree of Rnf complex subunits RnfB and RnfC of selected SRB. Sequences belonging to Rnf2-type complexes of *Desulfobacteraceae* members are highlighted by grey boxes. Branch support values are indicated in red. The tree was constructed using phylogeny.fr (*111*).

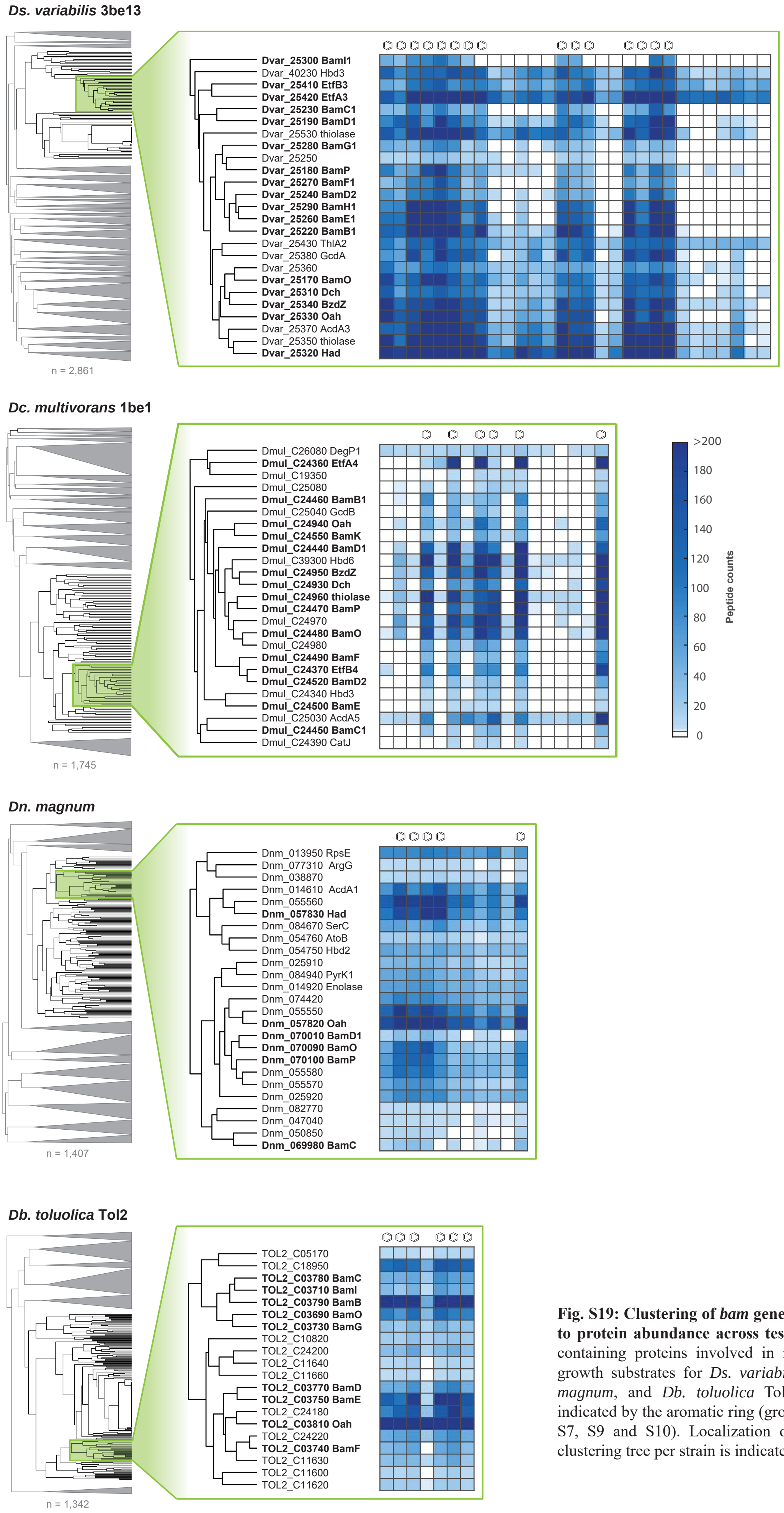

Fig. S19: Clustering of *bam* gene pathway module products according to protein abundance across tested substrate conditions. Sub-clusters containing proteins involved in reductive dearomatization of aromatic growth substrates for *Ds. variabilis* 3be13, *Dc. multivorans* 1be1, *Dn. magnum*, and *Db. toluolica* Tol2. Aromatic substrate conditions are indicated by the aromatic ring (growth conditions are as given in Figs. S6, S7, S9 and S10). Localization of respective clusters in the complete clustering tree per strain is indicated by the green box.

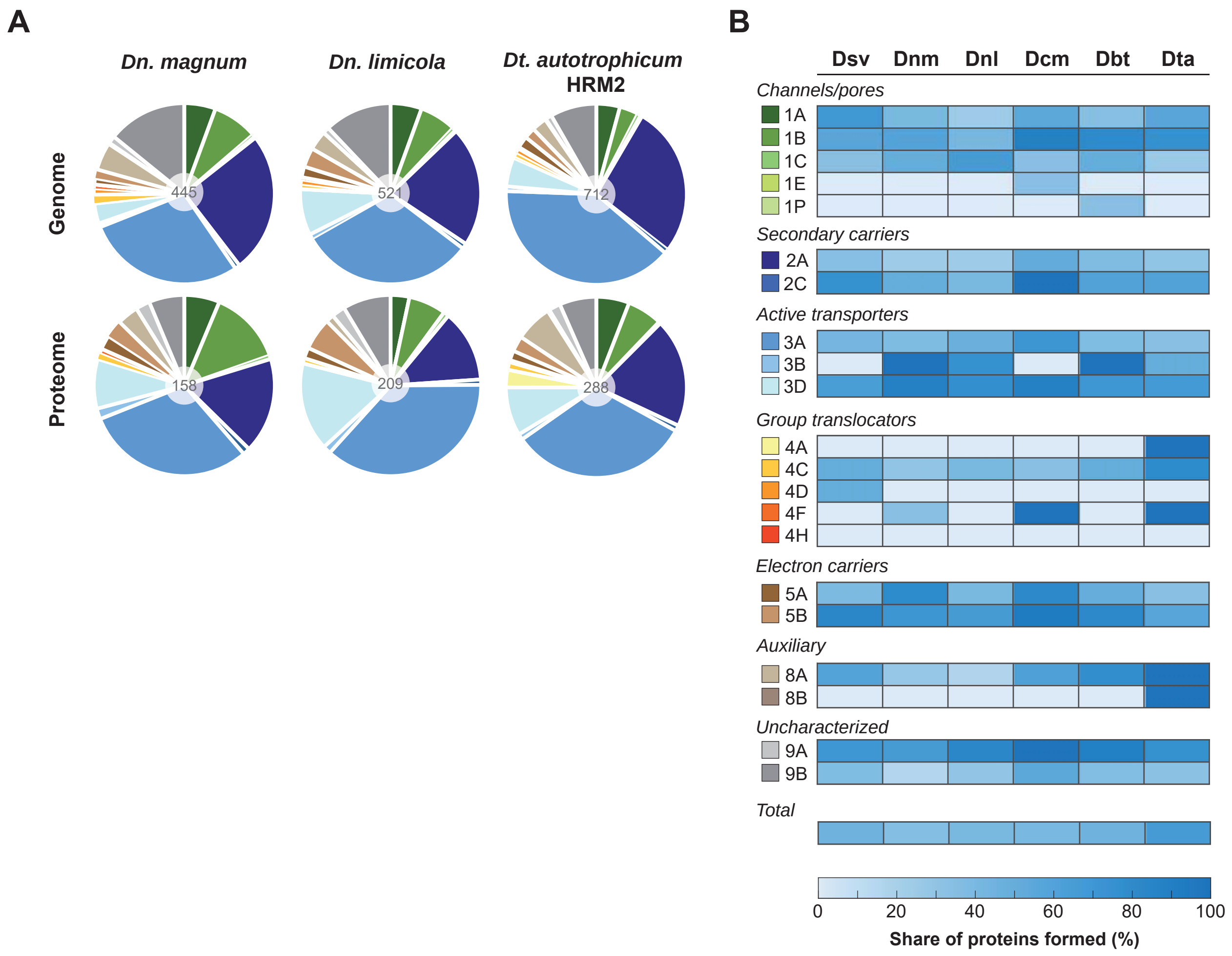

Fig. S20: Transporter capacities of *Dn. magnum*, *Dn. limicola* and *Dt. autotrophicum* HRM2. A, Share of predicted transport proteins according to TCDB in the respective genomes and proteomes. **B**, Share of proteins formed relative to genome encoded proteins per transporter subclass. Subclass codes: 1A, α-type channels; 1B, β-barrel porins; 1C, pore-forming toxins; 1E, holins; 1P, non-envelope virus penetration complex; 2A, uniporters/symporters/antiporters; 2C, ion-gradient-driven energizers; 3A, P-P-bond-hydrolysis-driven transporters; 3B, decarboxylation-driven transporters; 3D, oxidoreduction-driven transporters; 4A, phosphotransfer-driven group translocators; 4C, acyl CoA ligase-coupled transporters; 4D, polysaccharide synthase/exporters; 4F, choline/ethanolamine phosphotransferase 1; 4H, lysylphosphatidylglycerol synthase/flippases; 5A, transmembrane 2-electron transfer carriers; 5B, transmembrane 1-electron transfer carriers; 8A, auxiliary transport proteins; 8B, ribosomally synthesized protein/peptide toxins/agonists that target channels and carriers; 9A, recognized transporters of unknown biochemical mechanism; 9B, putative transport proteins.

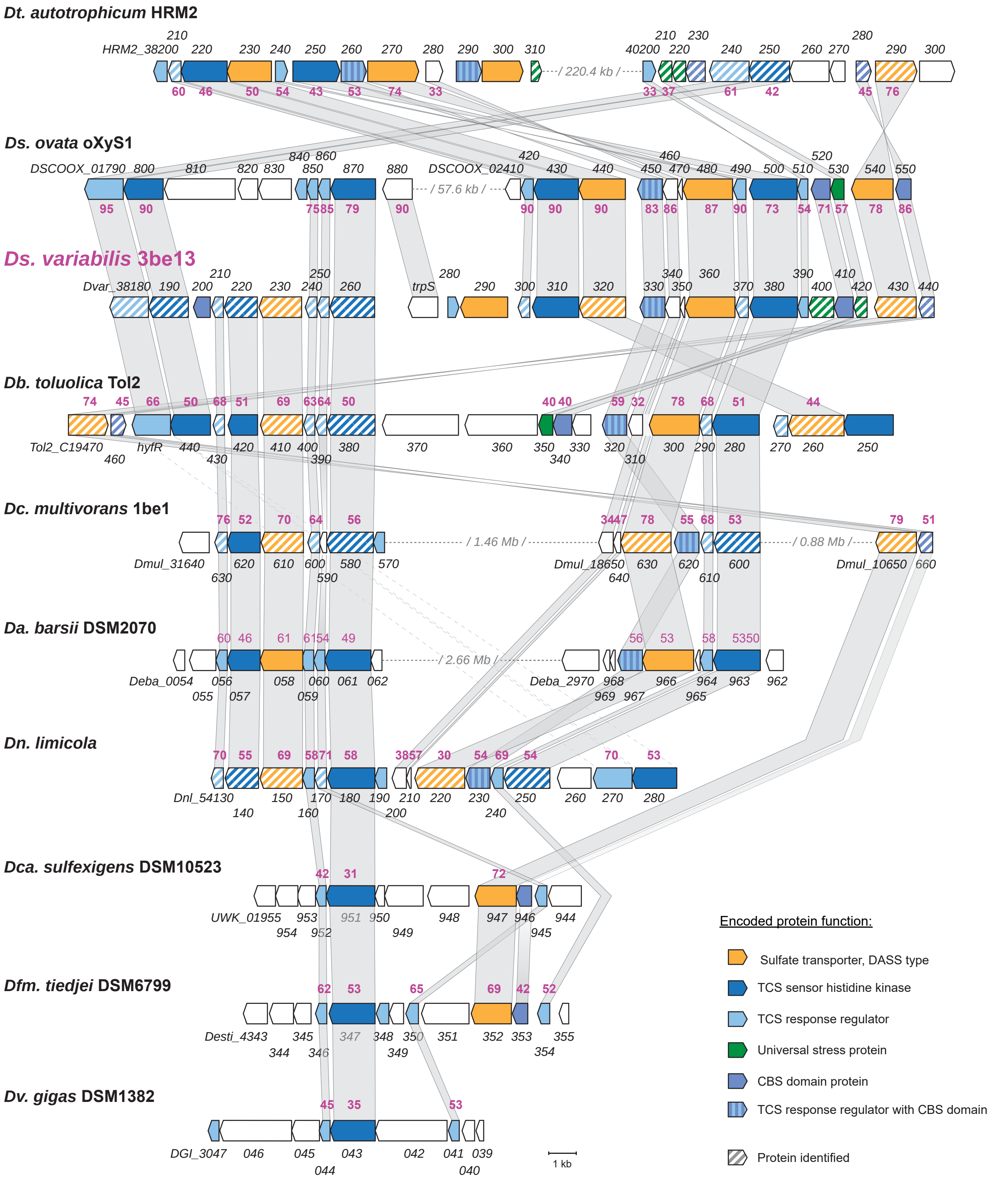

Fig. S21: Scale model of the DASS-type sulfate transporter encoding gene cluster of *Ds. variabilis* 3be13 compared to other selected SRB. Homologous genes are connected by grey shading and amino acid sequence identity to genes of *Ds. variabilis* 3be13 is given in violet. Locus-tags (or gene names) are presented in black and hatching highlights genes with identified encoded proteins. Functions are color coded (bottom right).

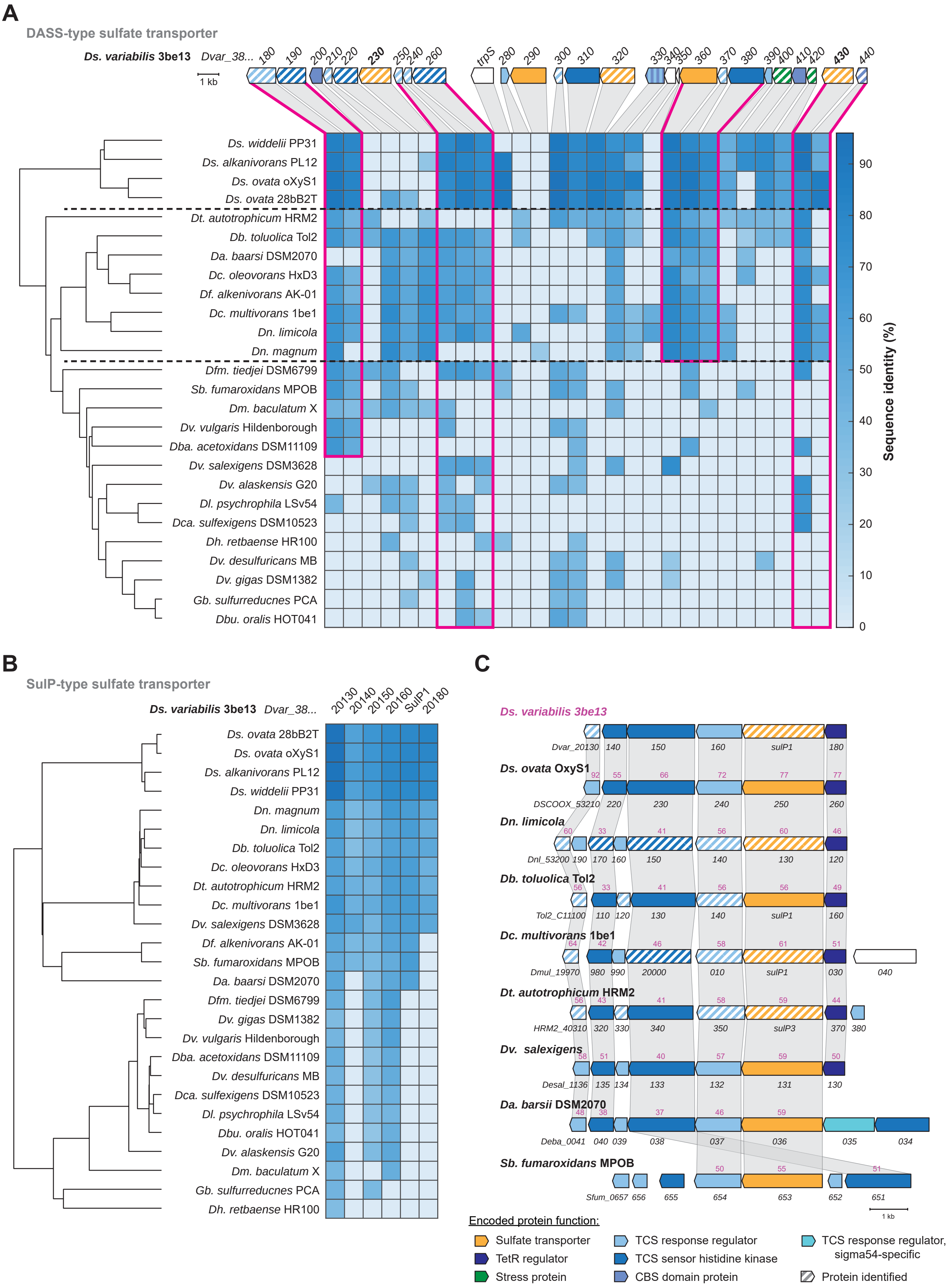

Fig. S22: Sulfate uptake by DASS- and SulP-type transporters in selected SRB. A, Scale model of the DASS-type sulfate transporter encoding gene cluster of *Ds. variabilis* 3be13 and heatmap of amino acid sequence identity between homologs of *Ds. variabilis* 3be13 and other SRB. Hierarchical clustering (Euclidian distance) was applied to identify the relationship among the respective gene repertoires (correlation coefficient 0.91). The cutting points for cluster definition are indicated by the dashed line. **B**, Amino acid sequence identity-based clustering heatmap of predicted SulP-type transporter gene cluster components relative to Ds. *variabilis* 3be3. Hierarchical clustering was applied to identify relationships among the respective gene repertoires of the different SRB (correlation coefficient 0.83). **C**, Scale model of the SulP-type sulfate transporter encoding gene cluster of *Ds. variabilis* 3be3 compared to other selected SRB. Homologous genes are connected by grey shading and amino acid sequence identity to genes of *Ds. variabilis* 3be13 is given in violet. Locus-tags (or gene names) are presented in black and hatching highlights genes with identified encoded proteins. Functions are color coded (bottom right).

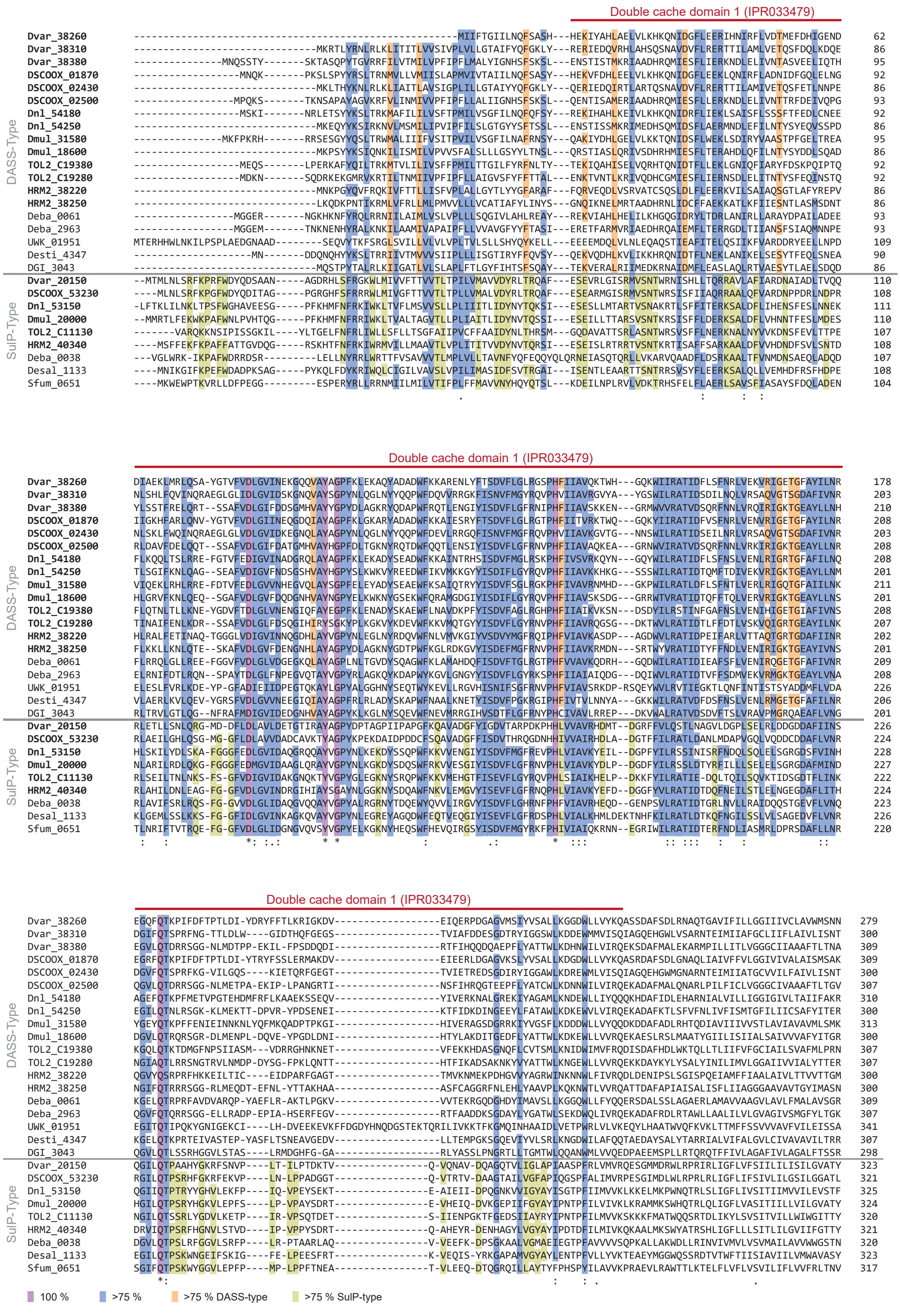

Fig. S23: Protein multiple sequence alignment of sensor histidine kinases predicted to be involved in regulation of sulfate uptake by DASS- and SulP-type transporters (separated by a gray line). Conserved regions among all sequences (blue or purple) or transporter class specific (orange or green for DASS- or SulP-type, respectively) are indicated. The region delineating the location of the effector recognizing double cache domain 1 is given. Numbers to the right of the alignments indicate amino acid positions per protein.

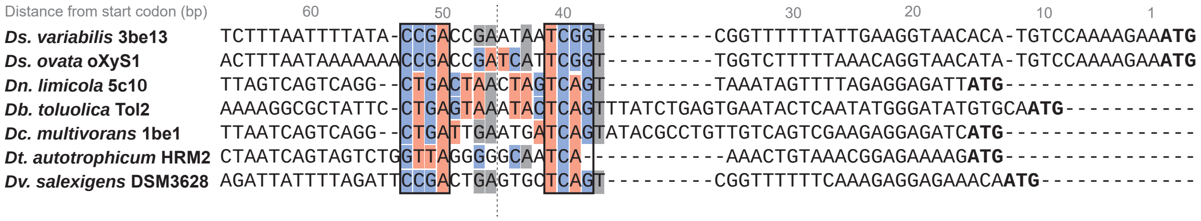

Fig. S24: Nucleic acid sequence alignment of the upstream region of SulP-type transporter encoding gene clusters of selected SRB. The start codon of the corresponding TetR-type regulator gene is indicated in bold font and nucleotide positions indicated for *Ds. variabilis* 3be13. Conserved, palindromic sequences are highlighted in blue (C and G) and red (A and T), respectively.

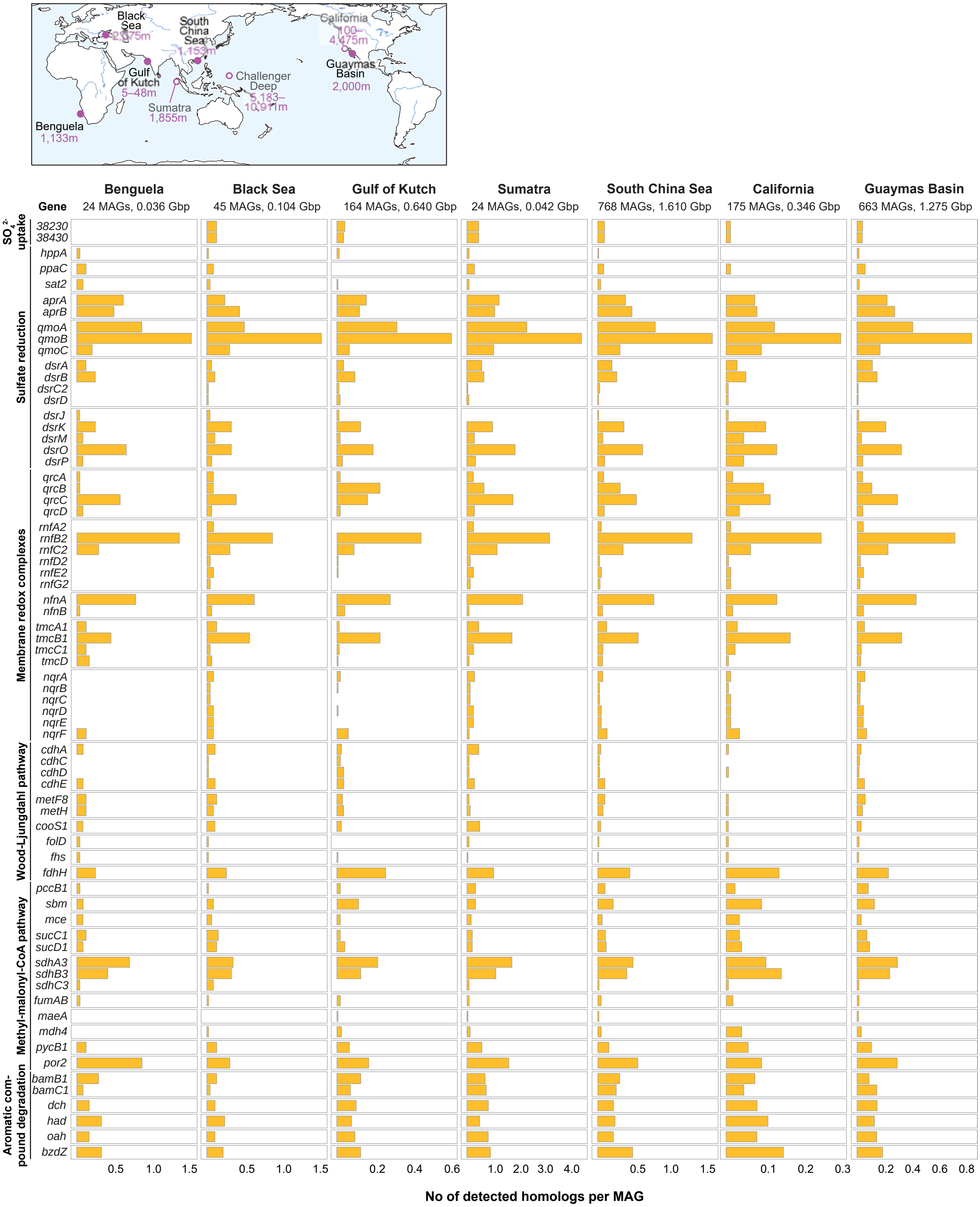

Fig. S25: Characteristic *Desulfobacteraceae* "catabolic" genes are prevalent in marine sediments. Location of queried metagenome samples and corresponding depth below sea surface (top). Distribution patterns of detected homologs in MAGs from indicated sites. Diversity profiles are summarized in table S10 and detailed data is available in Data S2. Gene names and locustags refer to *Ds. variabilis* 3be13. Number of MAGs and corresponding size of all assembled genomes is indicated below the station names.

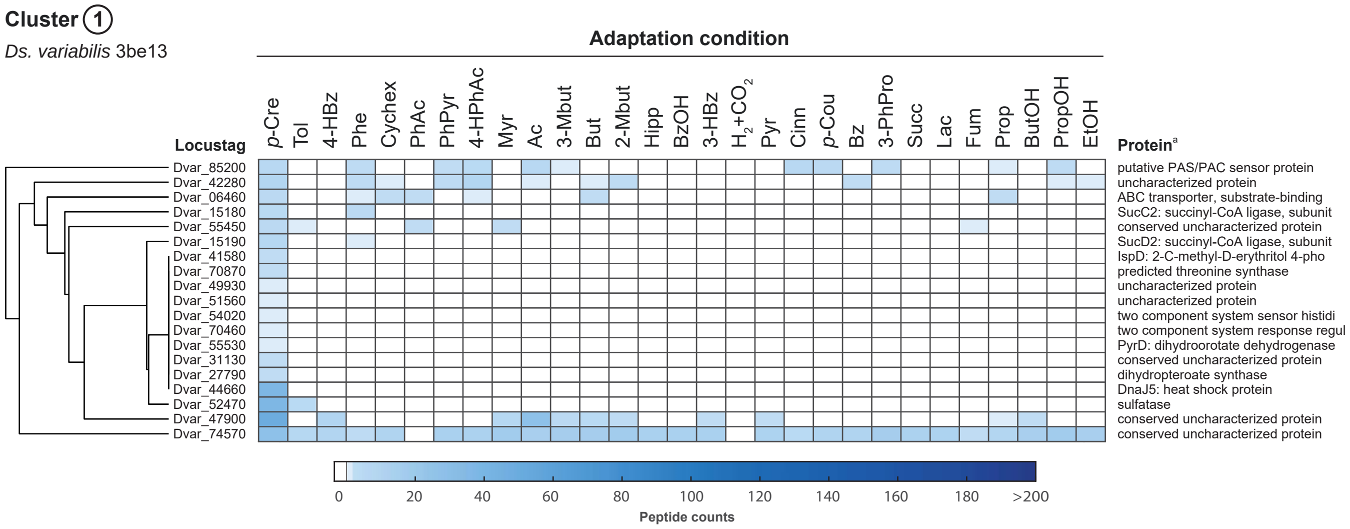

Fig. S26: Detailed view on abundances of proteins forming a sub-cluster in the global clustering depicted in Fig. S6. The global clustering is based on standardized protein abundances of all detected proteins of *Ds. variabilis* 3be13 across the 29 studied substrate adaptation conditions.

[a] Full description of protein functions are available in the genome annotation under accession CP159846.

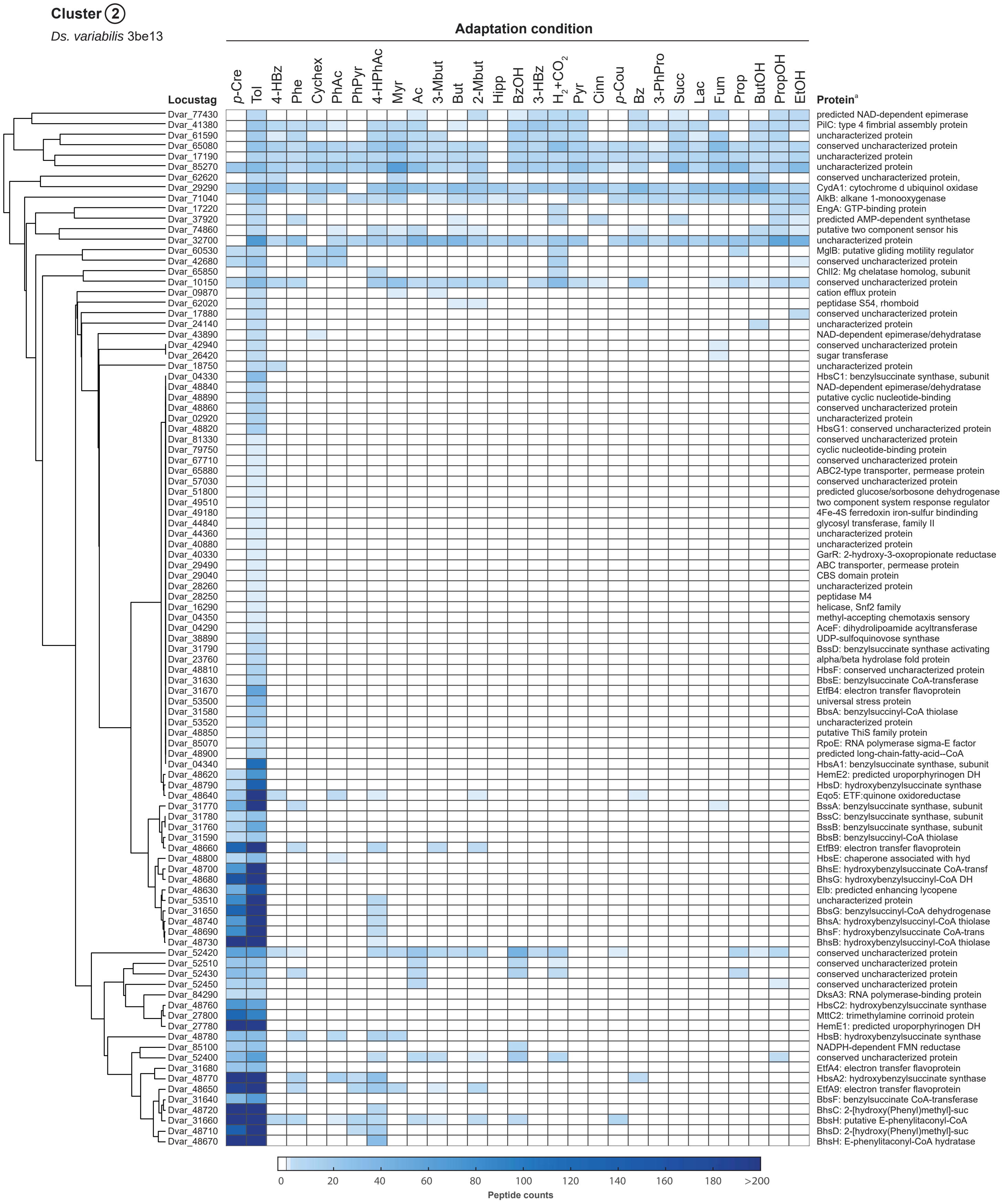

Fig. S27: Detailed view on abundances of proteins forming a sub-cluster in the global clustering depicted in Fig. S6. The global clustering is based on standardized protein abundances of all detected proteins of *Ds. variabilis* 3be13 across the 29 studied substrate adaptation conditions.

[a] Full description of protein functions are available in the genome annotation under accession CP159846.

Cluster 3

Ds. variabilis 3be13

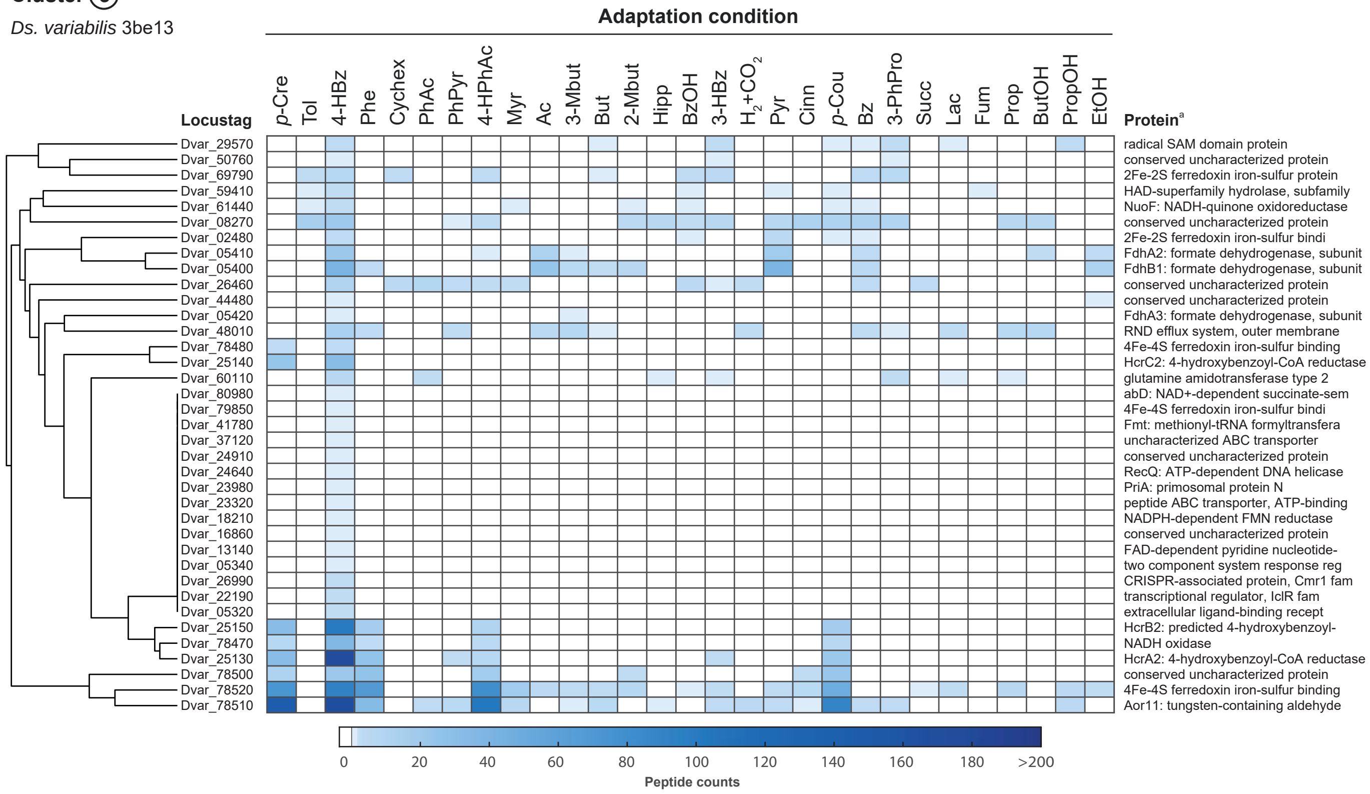

Fig. S28: Detailed view on abundances of proteins forming a sub-cluster in the global clustering depicted in Fig. S6. The global clustering is based on standardized protein abundances of all detected proteins of *Ds. variabilis* 3be13 across the 29 studied substrate adaptation conditions.

[a] Full description of protein functions are available in the genome annotation under accession CP159846.

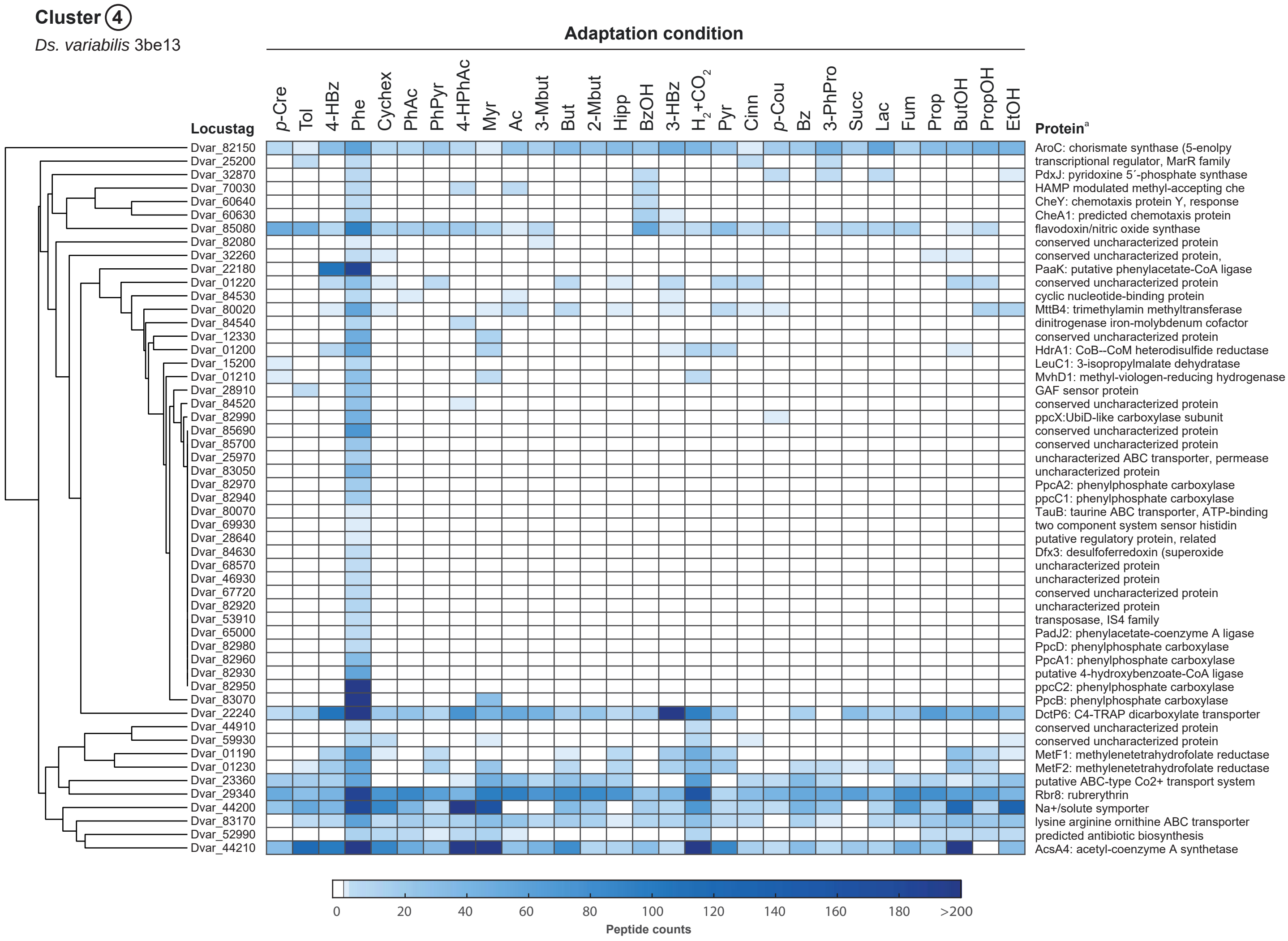

Fig. S29: Detailed view on abundances of proteins forming a sub-cluster in the global clustering depicted in Fig. S6. The global clustering is based on standardized protein abundances of all detected proteins of *Ds. variabilis* 3be13 across the 29 studied substrate adaptation conditions.
[a] Full description of protein functions are available in the genome annotation under accession CP159846.

Cluster 5

Ds. variabilis 3be13

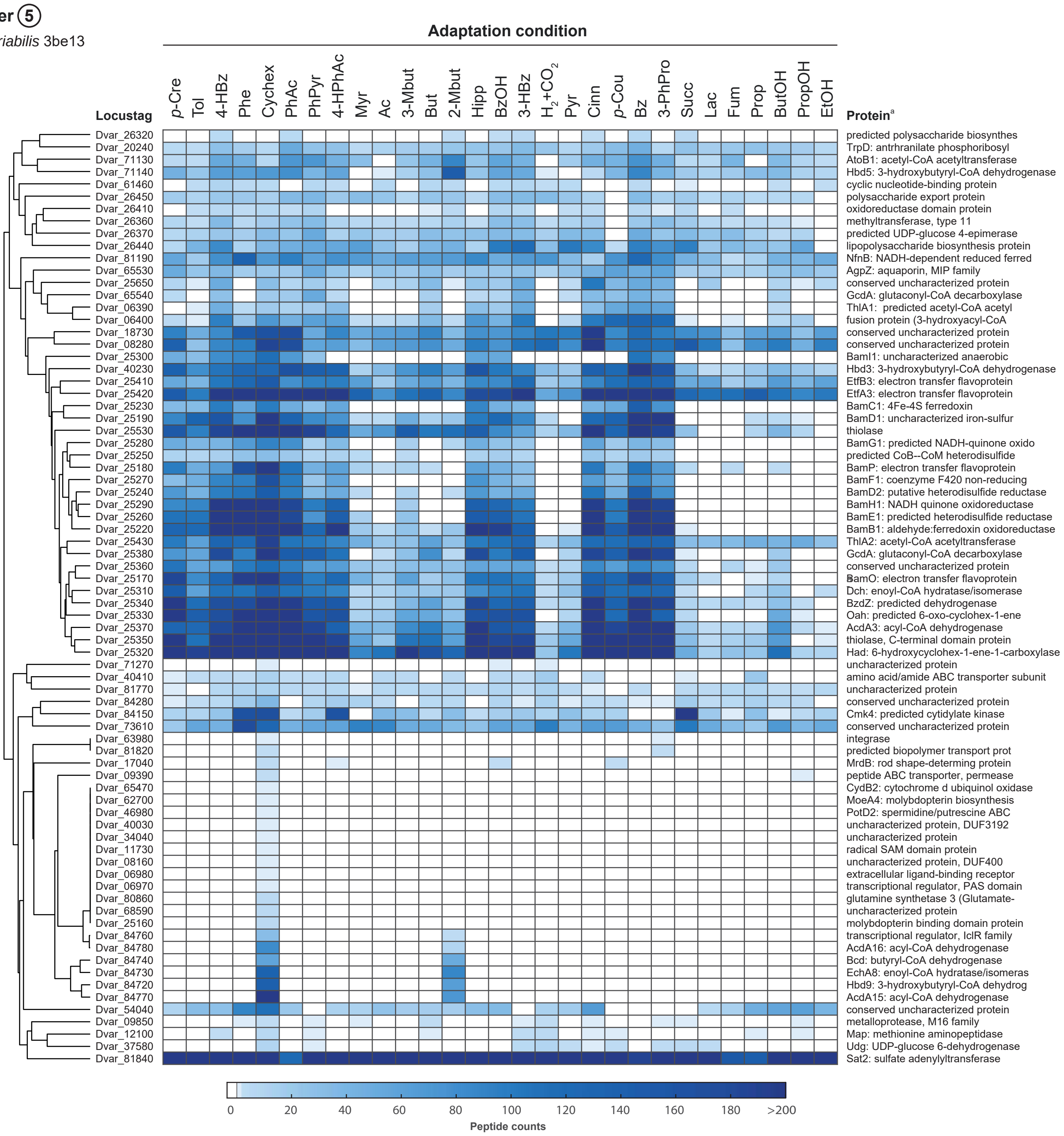

Fig. S30: Detailed view on abundances of proteins forming a sub-cluster in the global clustering depicted in Fig. S6. The global clustering is based on standardized protein abundances of all detected proteins of *Ds. variabilis* 3be13 across the 29 studied substrate adaptation conditions.

[a] Full description of protein functions are available in the genome annotation under accession CP159846.

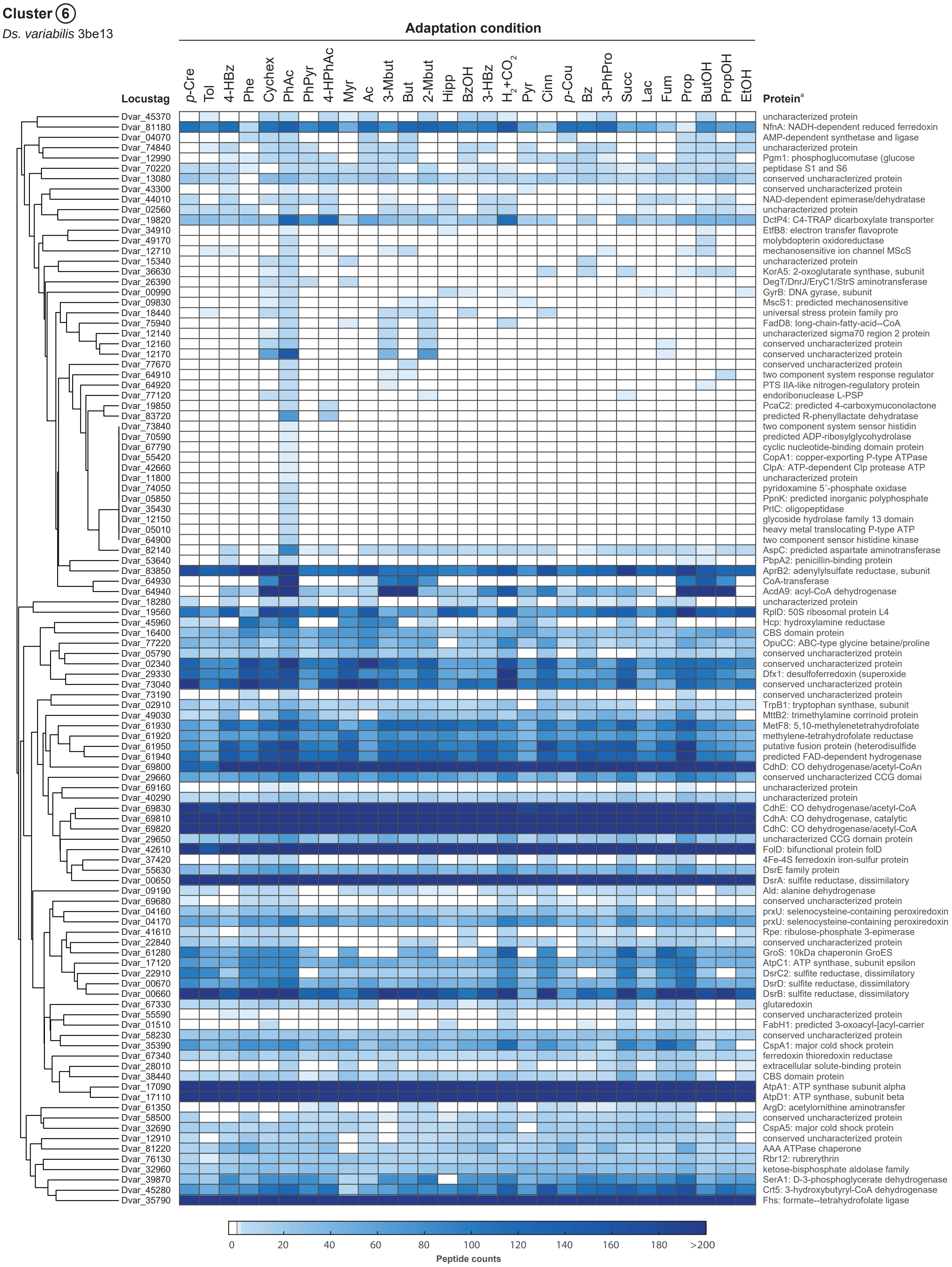

Fig. S31: Detailed view on abundances of proteins forming a sub-cluster in the global clustering depicted in Fig. S6. The global clustering is based on standardized protein abundances of all detected proteins of *Ds. variabilis* 3be13 across the 29 studied substrate adaptation conditions.

[a] Full description of protein functions are available in the genome annotation under accession CP159846.

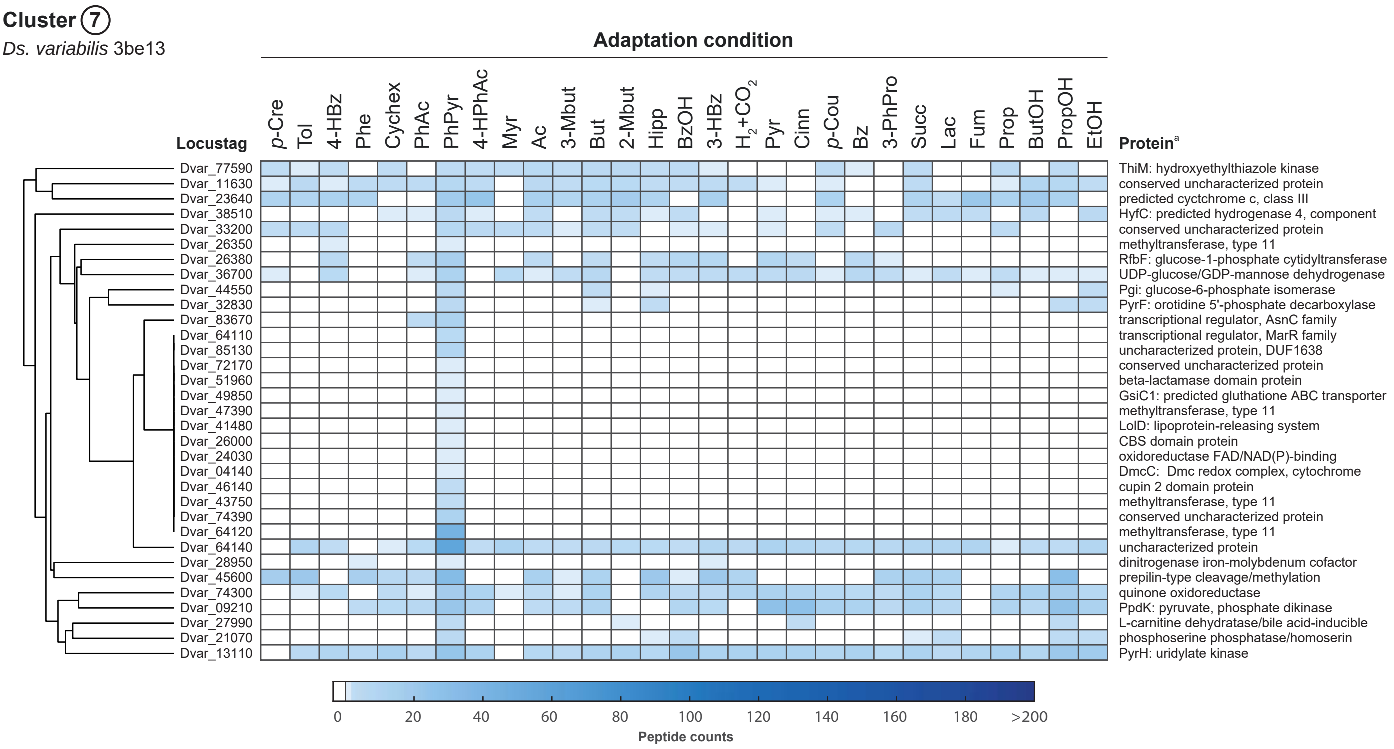

Fig. S32: Detailed view on abundances of proteins forming a sub-cluster in the global clustering depicted in Fig. S6. The global clustering is based on standardized protein abundances of all detected proteins of *Ds. variabilis* 3be13 across the 29 studied substrate adaptation conditions.

[a] Full description of protein functions are available in the genome annotation under accession CP159846.

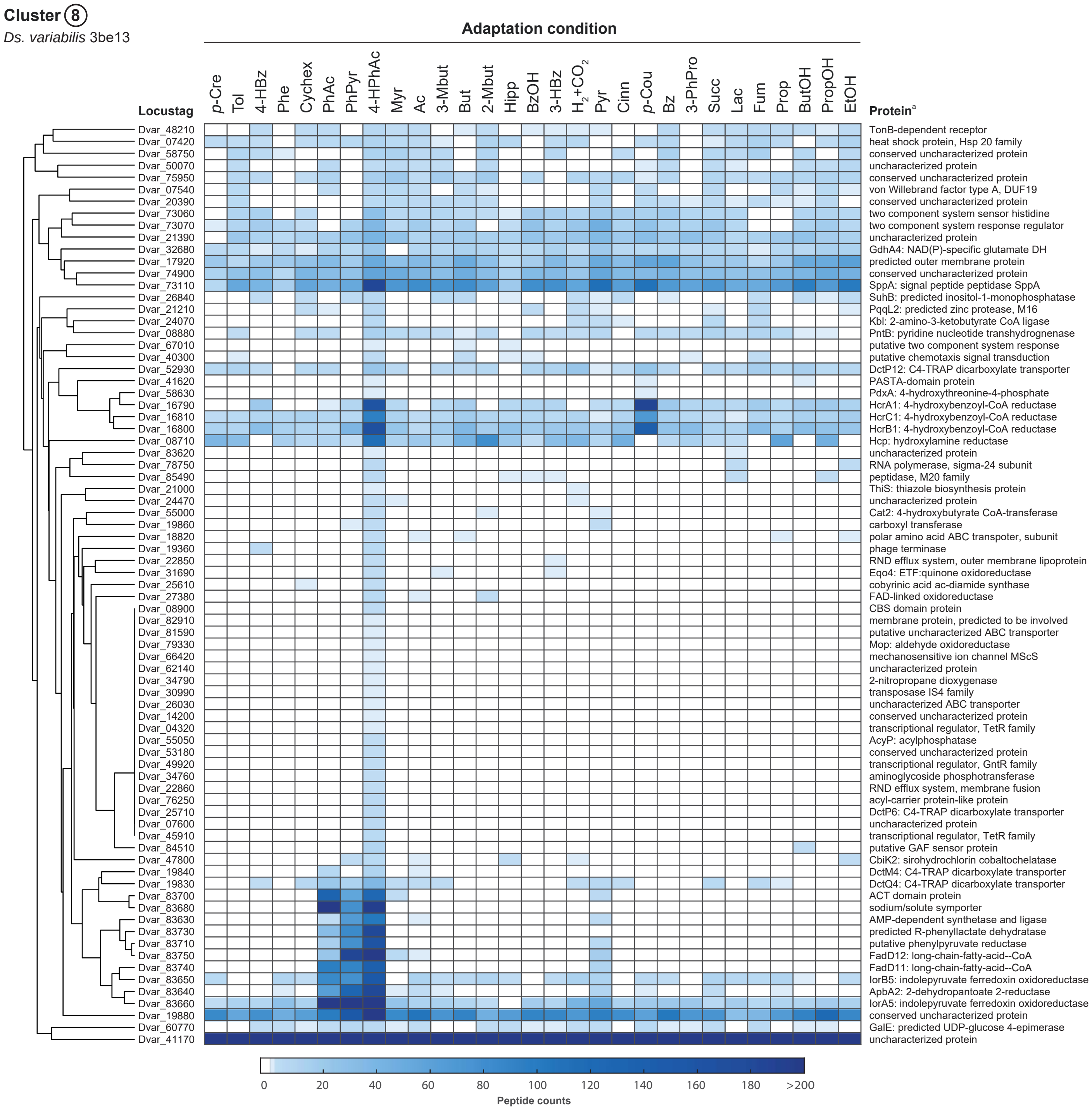

Fig. S33: Detailed view on abundances of proteins forming a sub-cluster in the global clustering depicted in Fig. S6. The global clustering is based on standardized protein abundances of all detected proteins of *Ds. variabilis* 3be13 across the 29 studied substrate adaptation conditions.

[a] Full description of protein functions are available in the genome annotation under accession CP159846.

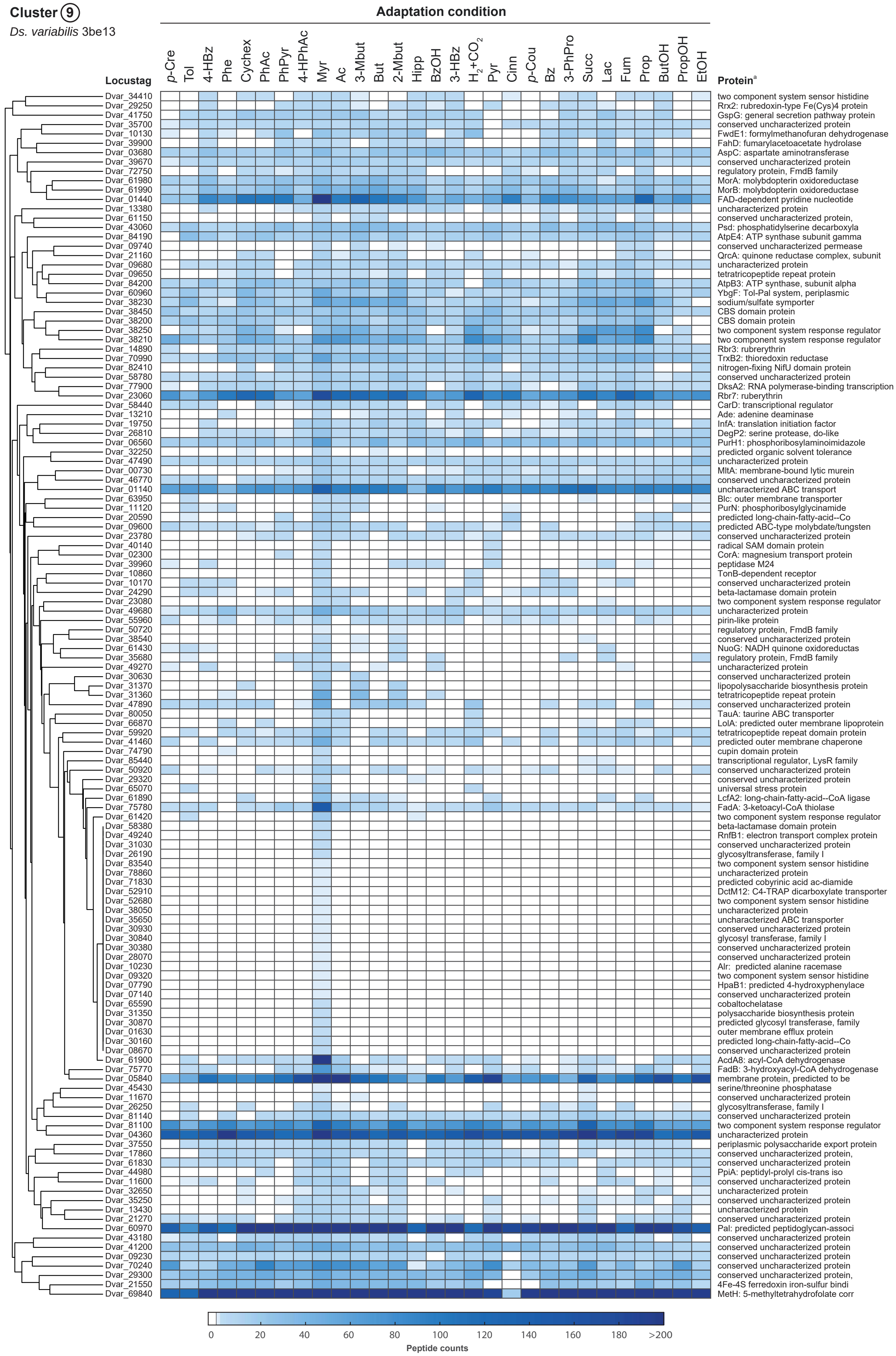

Fig. S34: Detailed view on abundances of proteins forming a sub-cluster in the global clustering depicted in Fig. S6. The global clustering is based on standardized protein abundances of all detected proteins of *Ds. variabilis* 3be13 across the 29 studied substrate adaptation conditions.
[a] Full description of protein functions are available in the genome annotation under accession CP159846.

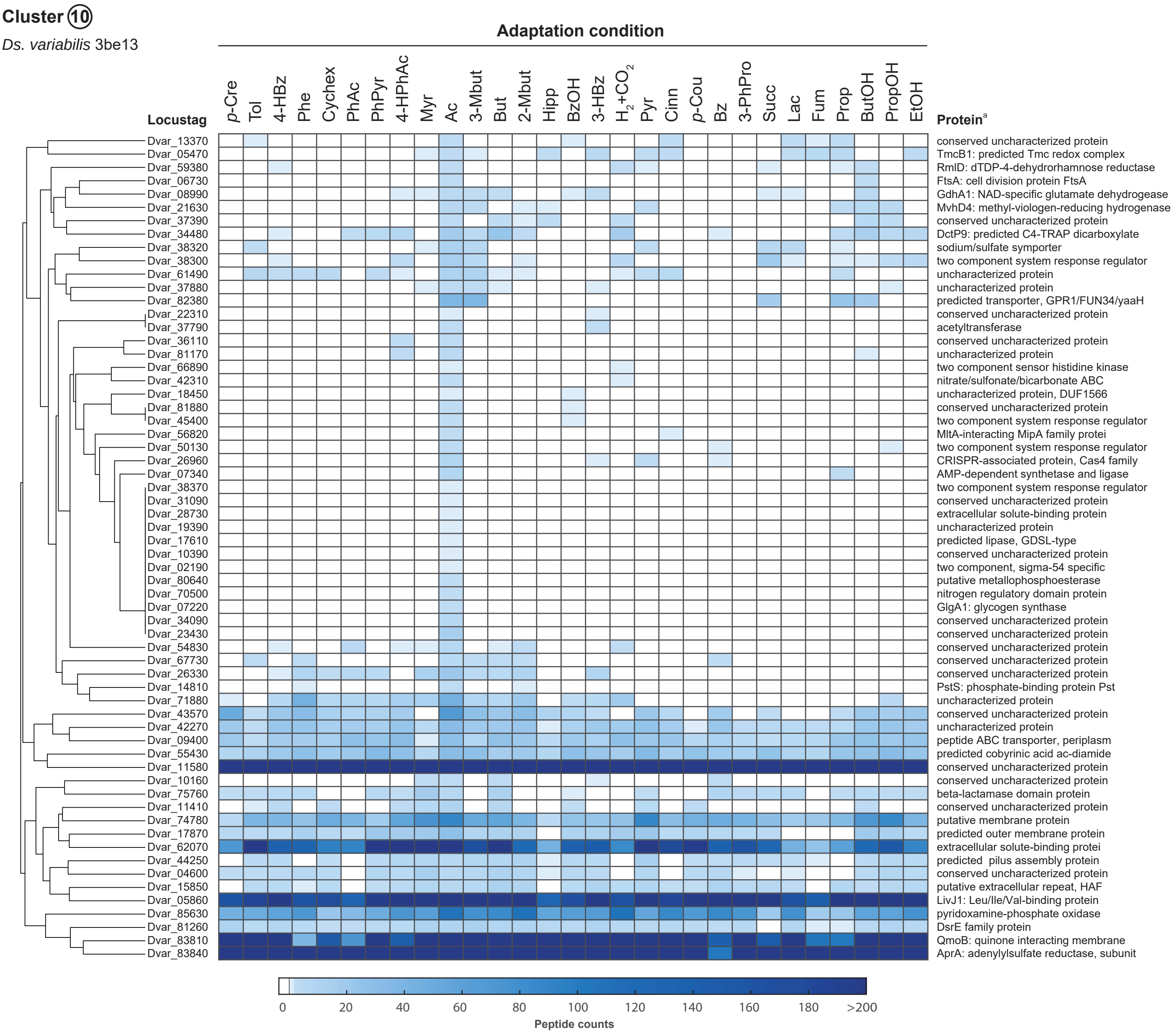

Fig. S35: Detailed view on abundances of proteins forming a sub-cluster in the global clustering depicted in Fig. S6. The global clustering is based on standardized protein abundances of all detected proteins of *Ds. variabilis* 3be13 across the 29 studied substrate adaptation conditions.

[a] Full description of protein functions are available in the genome annotation under accession CP159846.

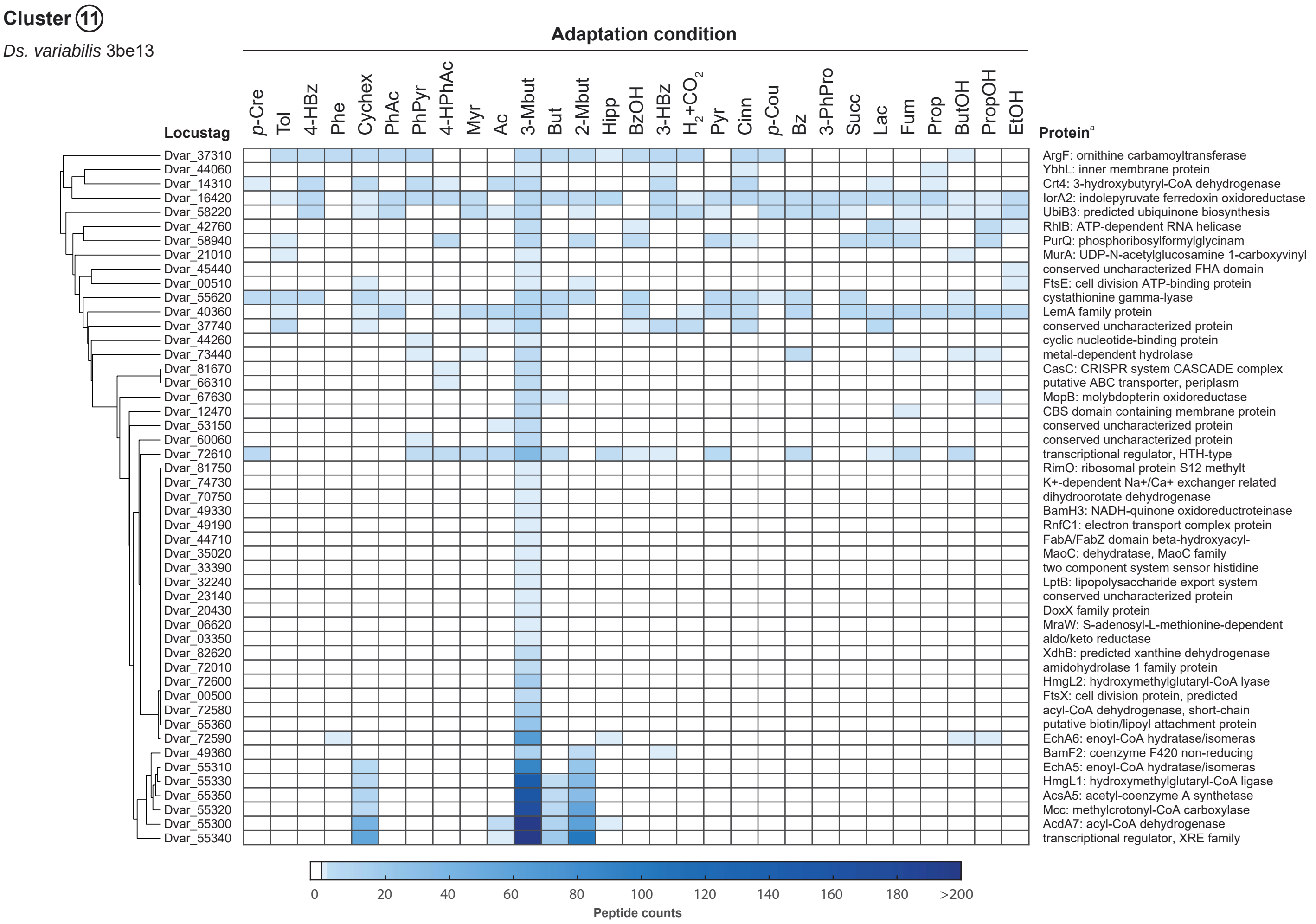

Fig. S36: Detailed view on abundances of proteins forming a sub-cluster in the global clustering depicted in Fig. S6. The global clustering is based on standardized protein abundances of all detected proteins of *Ds. variabilis* 3be13 across the 29 studied substrate adaptation conditions.

[a] Full description of protein functions are available in the genome annotation under accession CP159846.

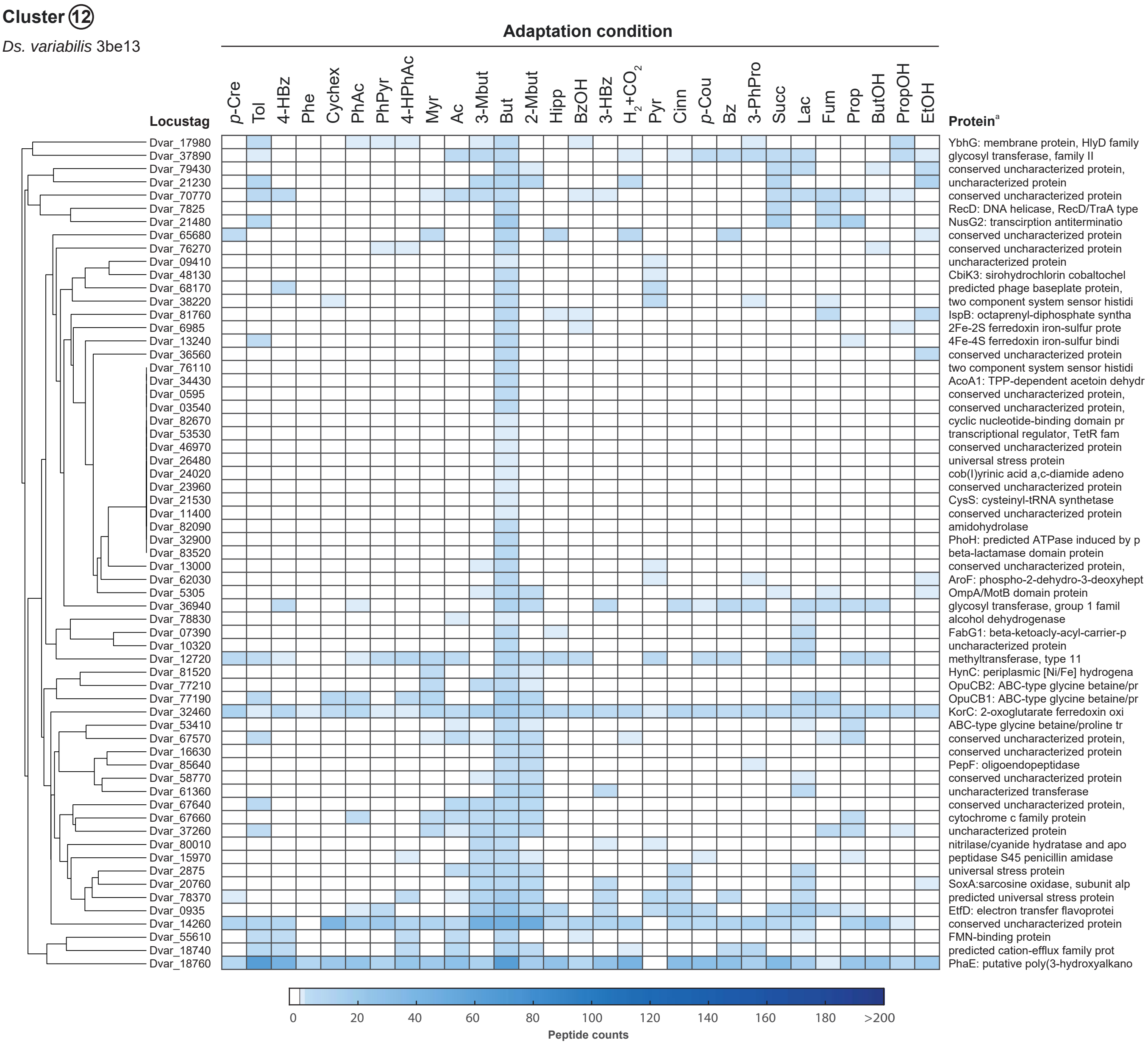

Fig. S37: Detailed view on abundances of proteins forming a sub-cluster in the global clustering depicted in Fig. S6. The global clustering is based on standardized protein abundances of all detected proteins of *Ds. variabilis* 3be13 across the 29 studied substrate adaptation conditions.

[a] Full description of protein functions are available in the genome annotation under accession CP159846.

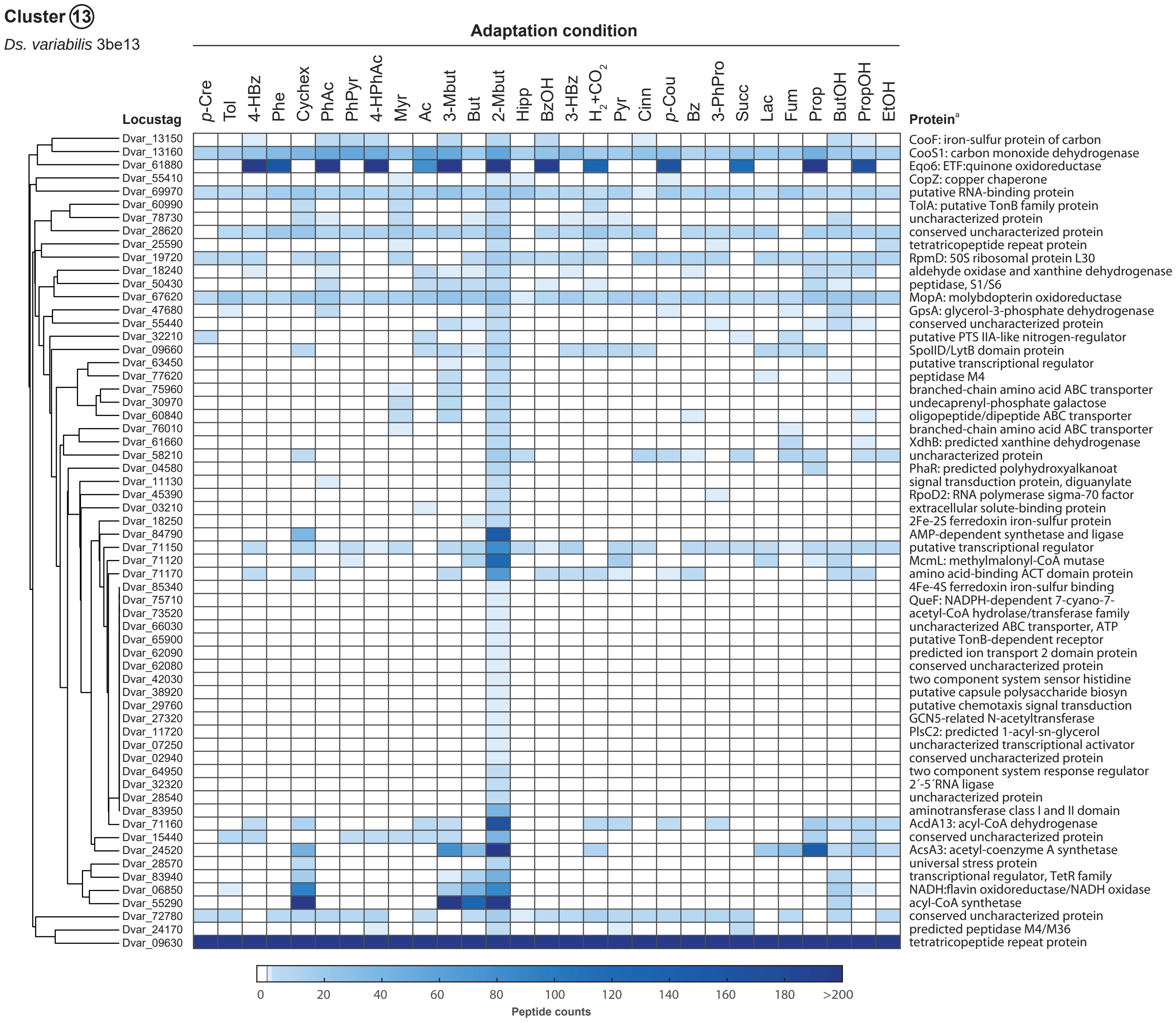

Fig. S38: Detailed view on abundances of proteins forming a sub-cluster in the global clustering depicted in Fig. S6. The global clustering is based on standardized protein abundances of all detected proteins of *Ds. variabilis* 3be13 across the 29 studied substrate adaptation conditions.
[a] Full description of protein functions are available in the genome annotation under accession CP159846.

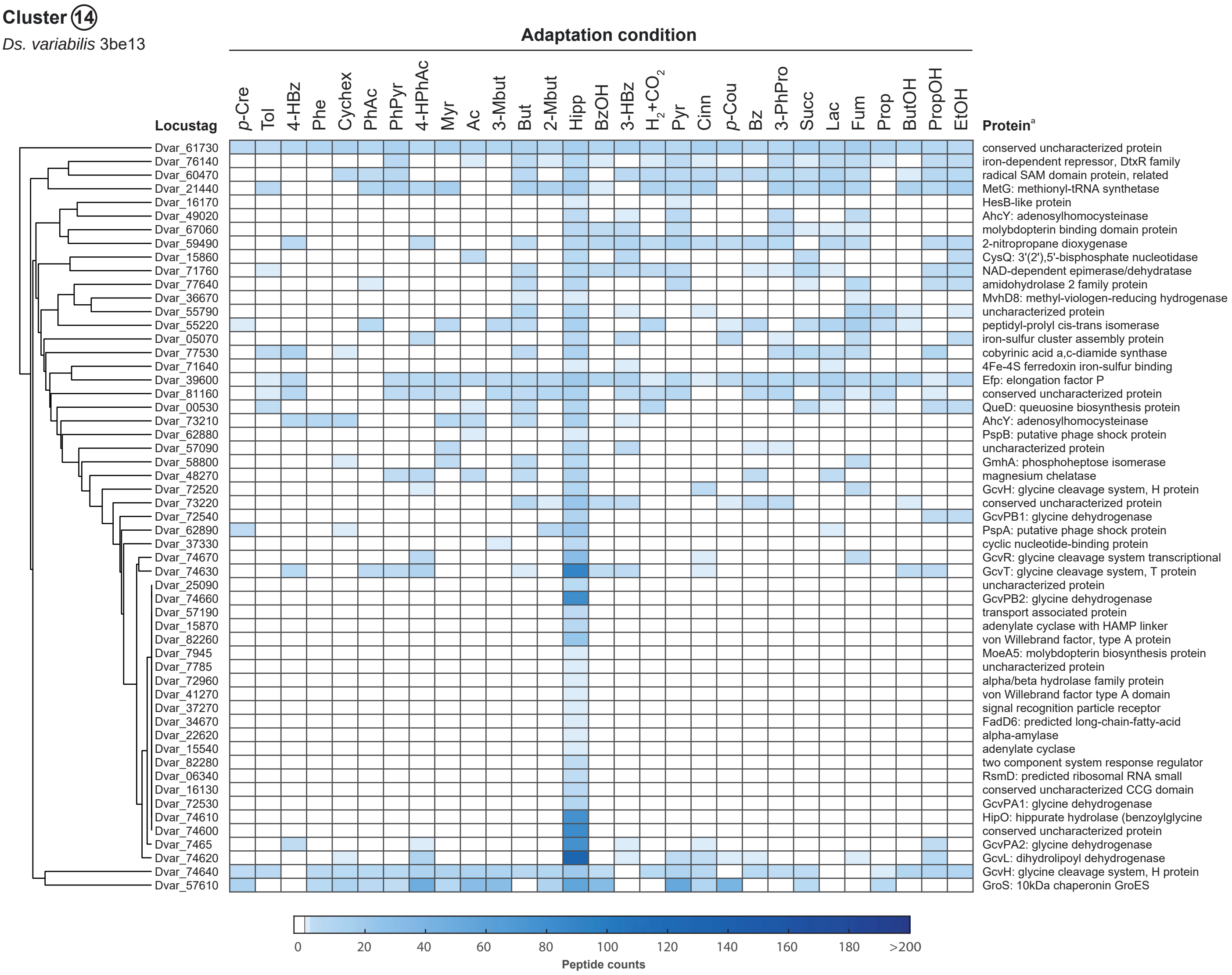

Fig. S39: Detailed view on abundances of proteins forming a sub-cluster in the global clustering depicted in Fig. S6. The global clustering is based on standardized protein abundances of all detected proteins of *Ds. variabilis* 3be13 across the 29 studied substrate adaptation conditions.

[a] Full description of protein functions are available in the genome annotation under accession CP159846.

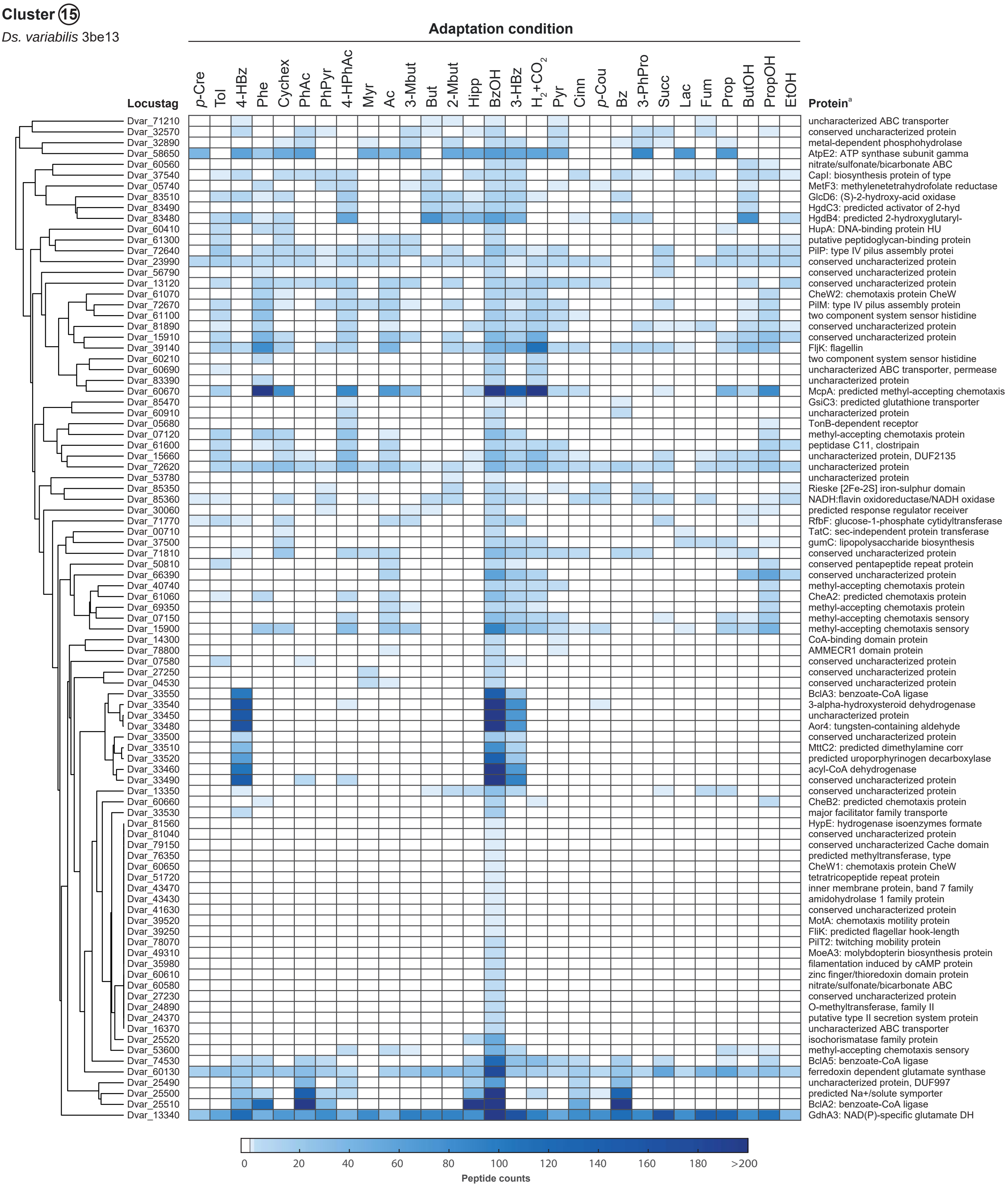

Fig. S40: Detailed view on abundances of proteins forming a sub-cluster in the global clustering depicted in Fig. S6. The global clustering is based on standardized protein abundances of all detected proteins of *Ds. variabilis* 3be13 across the 29 studied substrate adaptation conditions.

[a] Full description of protein functions are available in the genome annotation under accession CP159846.

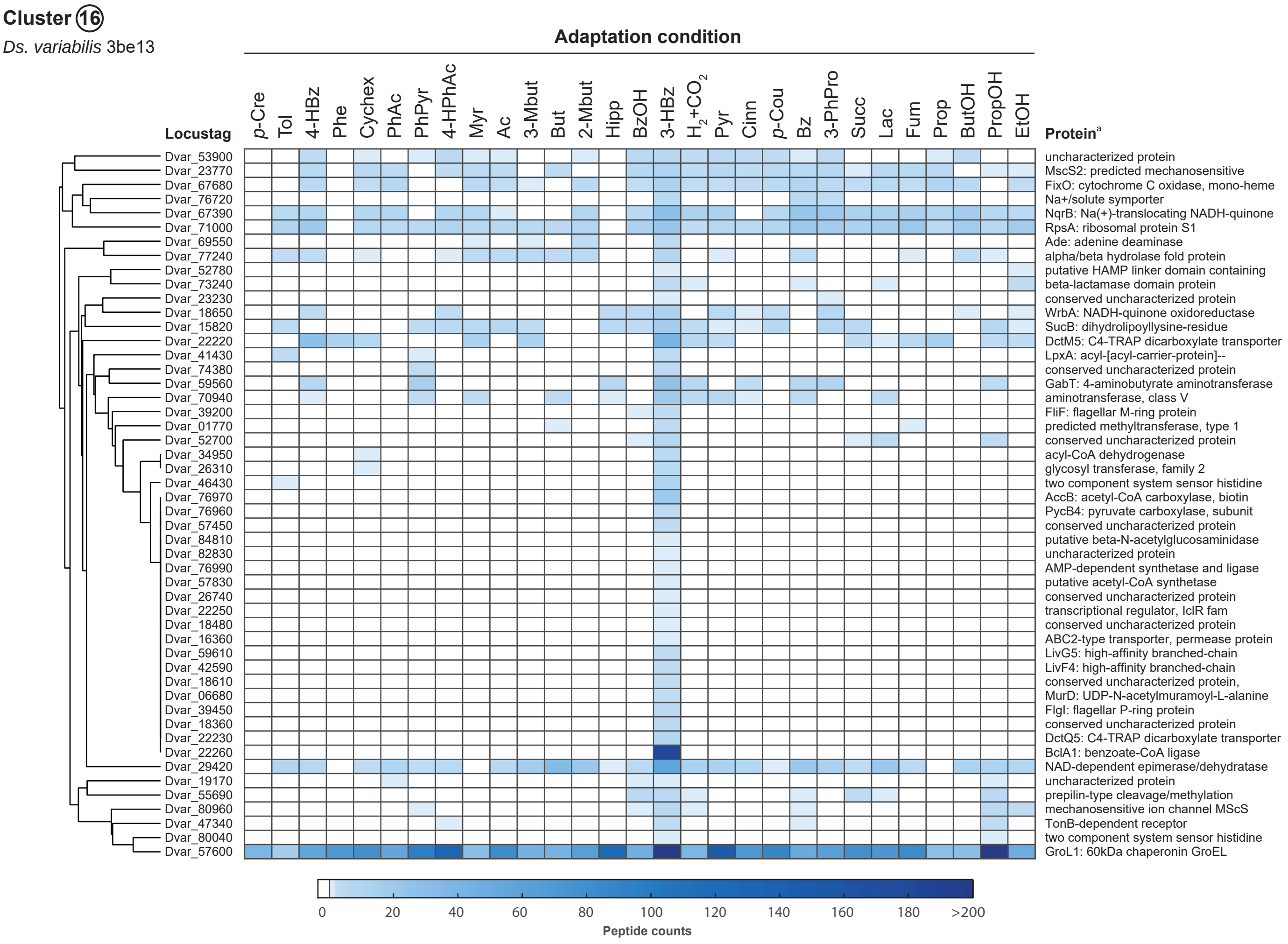

Fig. S41: Detailed view on abundances of proteins forming a sub-cluster in the global clustering depicted in Fig. S6. The global clustering is based on standardized protein abundances of all detected proteins of *Ds. variabilis* 3be13 across the 29 studied substrate adaptation conditions.

[a] Full description of protein functions are available in the genome annotation under accession CP159846.

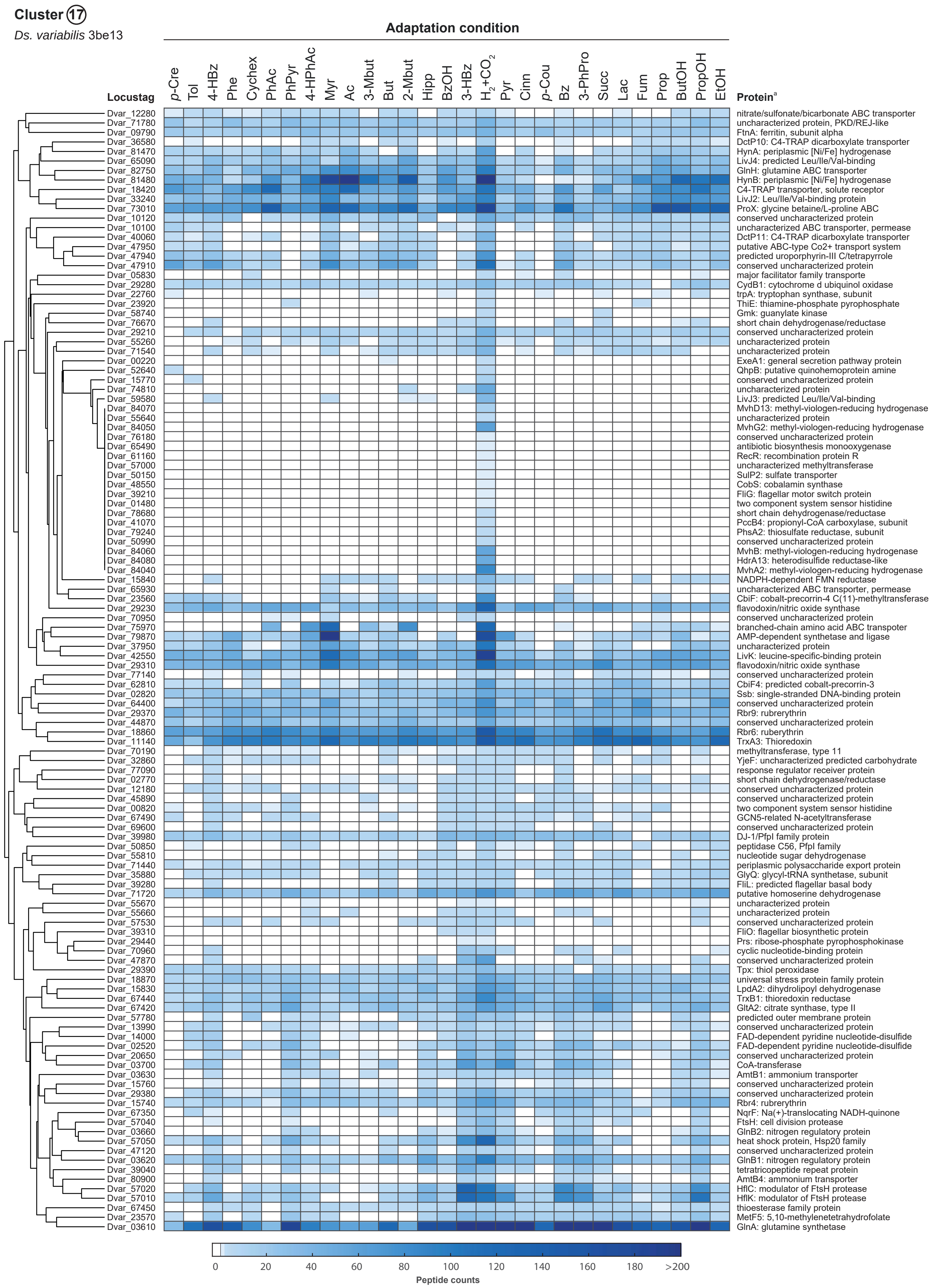

Fig. S42: Detailed view on abundances of proteins forming a sub-cluster in the global clustering depicted in Fig. S6. The global clustering is based on standardized protein abundances of all detected proteins of *Ds. variabilis* 3be13 across the 29 studied substrate adaptation conditions.

[a] Full description of protein functions are available in the genome annotation under accession CP159846.

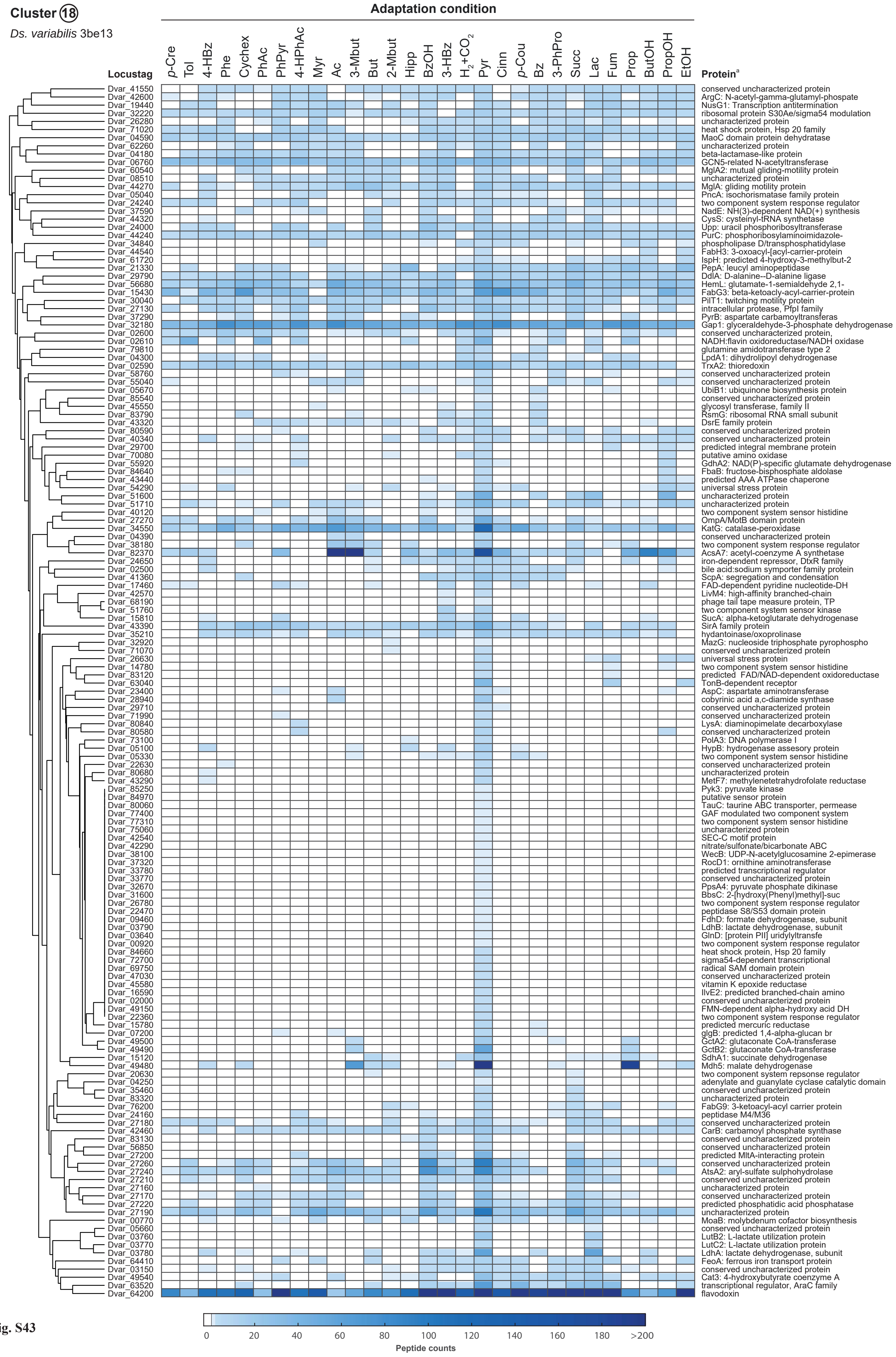

Cluster 18
Ds. variabilis 3be13
Adaptation condition
Locustag
p-Cre
Tol
4-HBz
Phe
Cychex
PhAc
PhPyr
4-HPhAc
Myr
Ac
3-Mbut
But
2-Mbut
Hipp
BzOH
3-HBz
H_2+CO_2
Pyr
Cinn
p-Cou
Bz
3-PhPro
Succ
Lac
Fum
Prop
ButOH
PropOH
EtOH
Protein[a]
Dvar_41550 conserved uncharacterized protein
Dvar_42600 ArgC: N-acetyl-gamma-glutamyl-phospate
Dvar_19440 NusG1: Transcription antitermination
Dvar_32220 ribosomal protein S30Ae/sigma54 modulation
Dvar_26280 uncharacterized protein
Dvar_71020 heat shock protein, Hsp 20 family
Dvar_04590 MaoC domain protein dehydratase
Dvar_62260 uncharacterized protein
Dvar_04180 beta-lactamase-like protein
Dvar_06760 GCN5-related N-acetyltransferase
Dvar_60540 MglA2: mutual gliding-motility protein
Dvar_08510 uncharacterized protein
Dvar_44270 MglA: gliding motility protein
Dvar_05040 PncA: isochorismatase family protein
Dvar_24240 two component system response regulator
Dvar_37590 NadE: NH(3)-dependent NAD(+) synthesis
Dvar_44320 CysS: cysteinyl-tRNA synthetase
Dvar_24000 Upp: uracil phosphoribosyltransferase
Dvar_44240 PurC: phosphoribosylaminoimidazole-
Dvar_34840 phospholipase D/transphosphatidylase
Dvar_44540 FabH3: 3-oxoacyl-[acyl-carrier-protein
Dvar_61720 IspH: predicted 4-hydroxy-3-methylbut-2
Dvar_21330 PepA: leucyl aminopeptidase
Dvar_29790 DdlA: D-alanine--D-alanine ligase
Dvar_56680 HemL: glutamate-1-semialdehyde 2,1-
Dvar_15430 FabG3: beta-ketoacly-acyl-carrier-protein
Dvar_30040 PilT1: twitching motility protein
Dvar_27130 intracellular protease, PfpI family
Dvar_37290 PyrB: aspartate carbamoyltransferas
Dvar_32180 Gap1: glyceraldehyde-3-phospate dehydrogenase
Dvar_02600 conserved uncharacterized protein,
Dvar_02610 NADH:flavin oxidoreductase/NADH oxidase
Dvar_79810 glutamine amidotransferase type 2
Dvar_04300 LpdA1: dihydrolipoyl dehydrogenase
Dvar_02590 TrxA2: thioredoxin
Dvar_58760 conserved uncharacterized protein
Dvar_55040 conserved uncharacterized protein
Dvar_05670 UbiB1: ubiquinone biosynthesis protein
Dvar_85540 conserved uncharacterized protein
Dvar_45550 glycosyl transferase, family II
Dvar_83790 RsmG: ribosomal RNA small subunit
Dvar_43320 DsrE family protein
Dvar_80590 conserved uncharacterized protein
Dvar_40340 conserved uncharacterized protein
Dvar_29700 predicted integral membrane protein
Dvar_70080 putative amino oxidase
Dvar_55920 GdhA2: NAD(P)-specific glutamate dehydrogenase
Dvar_84640 FbaB: fructose-bisphosphate aldolase
Dvar_43440 predicted AAA ATPase chaperone
Dvar_54290 universal stress protein
Dvar_51600 uncharacterized protein
Dvar_51710 uncharacterized protein
Dvar_40120 two component system sensor histidine
Dvar_27270 OmpA/MotB domain protein
Dvar_34550 KatG: catalase-peroxidase
Dvar_04390 conserved uncharacterized protein
Dvar_38180 two component system response regulator
Dvar_82370 AcsA7: acetyl-coenzyme A synthetase
Dvar_24650 iron-dependent repressor, DtxR family
Dvar_02500 bile acid:sodium symporter family protein
Dvar_41360 ScpA: segregation and condensation
Dvar_17460 FAD-dependent pyridine nucleotide-DH
Dvar_42570 LivM4: high-affinity branched-chain
Dvar_68190 phage tail tape measure protein, TP
Dvar_51760 two component system sensor kinase
Dvar_15810 SucA: alpha-ketoglutarate dehydrogenase
Dvar_43390 SirA family protein
Dvar_35210 hydantoinase/oxoprolinase
Dvar_32920 MazG: nucleoside triphosphate pyrophospho
Dvar_71070 conserved uncharacterized protein
Dvar_26630 universal stress protein
Dvar_14780 two component system sensor histidine
Dvar_83120 predicted FAD/NAD-dependent oxidoreductase
Dvar_63040 TonB-dependent receptor
Dvar_23400 AspC: aspartate aminotransferase
Dvar_28940 cobyrinic acid a,c-diamide synthase
Dvar_29710 conserved uncharacterized protein
Dvar_71990 conserved uncharacterized protein
Dvar_80840 LysA: diaminopimelate decarboxylase
Dvar_80580 conserved uncharacterized protein
Dvar_73100 PolA3: DNA polymerase I
Dvar_05100 HypB: hydrogenase assesory protein
Dvar_05330 two component system sensor histidine
Dvar_22630 conserved uncharacterized protein
Dvar_80680 uncharacterized protein
Dvar_43290 MetF7: methylenetetrahydrofolate reductase
Dvar_85250 Pyk3: pyruvate kinase
Dvar_84970 putative sensor protein
Dvar_80060 TauC: taurine ABC transporter, permease
Dvar_77400 GAF modulated two component system
Dvar_77310 two component system sensor histidine
Dvar_75060 uncharacterized protein
Dvar_42540 SEC-C motif protein
Dvar_42290 nitrate/sulfonate/bicarbonate ABC
Dvar_38100 WecB: UDP-N-acetylglucosamine 2-epimerase
Dvar_37320 RocD1: ornithine aminotransferase
Dvar_33780 predicted transcriptional regulator
Dvar_33770 conserved uncharacterized protein
Dvar_32670 PpsA4: pyruvate phosphate dikinase
Dvar_31600 BbsC: 2-[hydroxy(Phenyl)methyl]-suc
Dvar_26780 two component system response regulator
Dvar_22470 peptidase S8/S53 domain protein
Dvar_09460 FdhD: formate dehydrogenase, subunit
Dvar_03790 LdhB: lactate dehydrogenase, subunit
Dvar_03640 GlnD: [protein PII] uridylyltransfe
Dvar_00920 two component system response regulator
Dvar_84660 heat shock protein, Hsp 20 family
Dvar_72700 sigma54-dependent transcriptional
Dvar_69750 radical SAM domain protein
Dvar_47030 conserved uncharacterized protein
Dvar_45580 vitamin K epoxide reductase
Dvar_16590 IlvE2: predicted branched-chain amino
Dvar_02000 conserved uncharacterized protein
Dvar_49150 FMN-dependent alpha-hydroxy acid DH
Dvar_22360 two component system response regulator
Dvar_15780 predicted mercuric reductase
Dvar_07200 glgB: predicted 1,4-alpha-glucan br
Dvar_49500 GctA2: glutaconate CoA-transferase
Dvar_49490 GctB2: glutaconate CoA-transferase
Dvar_15120 SdhA1: succinate dehydrogenase
Dvar_49480 Mdh5: malate dehydrogenase
Dvar_20630 two component system repsonse regulator
Dvar_04250 adenylate and guanylate cyclase catalytic domain
Dvar_35460 conserved uncharacterized protein
Dvar_83320 uncharacterized protein
Dvar_76200 FabG9: 3-ketoacyl-acyl carrier protein
Dvar_24160 peptidase M4/M36
Dvar_27180 conserved uncharacterized protein
Dvar_42460 CarB: carbamoyl phosphate synthase
Dvar_83130 conserved uncharacterized protein
Dvar_56850 conserved uncharacterized protein
Dvar_27200 predicted MltA-interacting protein
Dvar_27260 conserved uncharacterized protein
Dvar_27240 AtsA2: aryl-sulfate sulphohydrolase
Dvar_27210 conserved uncharacterized protein
Dvar_27160 uncharacterized protein
Dvar_27170 conserved uncharacterized protein
Dvar_27220 predicted phosphatidic acid phosphatase
Dvar_27190 uncharacterized protein
Dvar_00770 MoaB: molybdenum cofactor biosynthesis
Dvar_05660 conserved uncharacterized protein
Dvar_03760 LutB2: L-lactate utilization protein
Dvar_03770 LutC2: L-lactate utilization protein
Dvar_03780 LdhA: lactate dehydrogenase, subunit
Dvar_64410 FeoA: ferrous iron transport protein
Dvar_03150 conserved uncharacterized protein
Dvar_49540 Cat3: 4-hydroxybutyrate coenzyme A
Dvar_63520 transcriptional regulator, AraC family
Dvar_64200 flavodoxin
0
20
40
60
80
100
120
140
160
180
>200
Peptide counts

Fig. S43

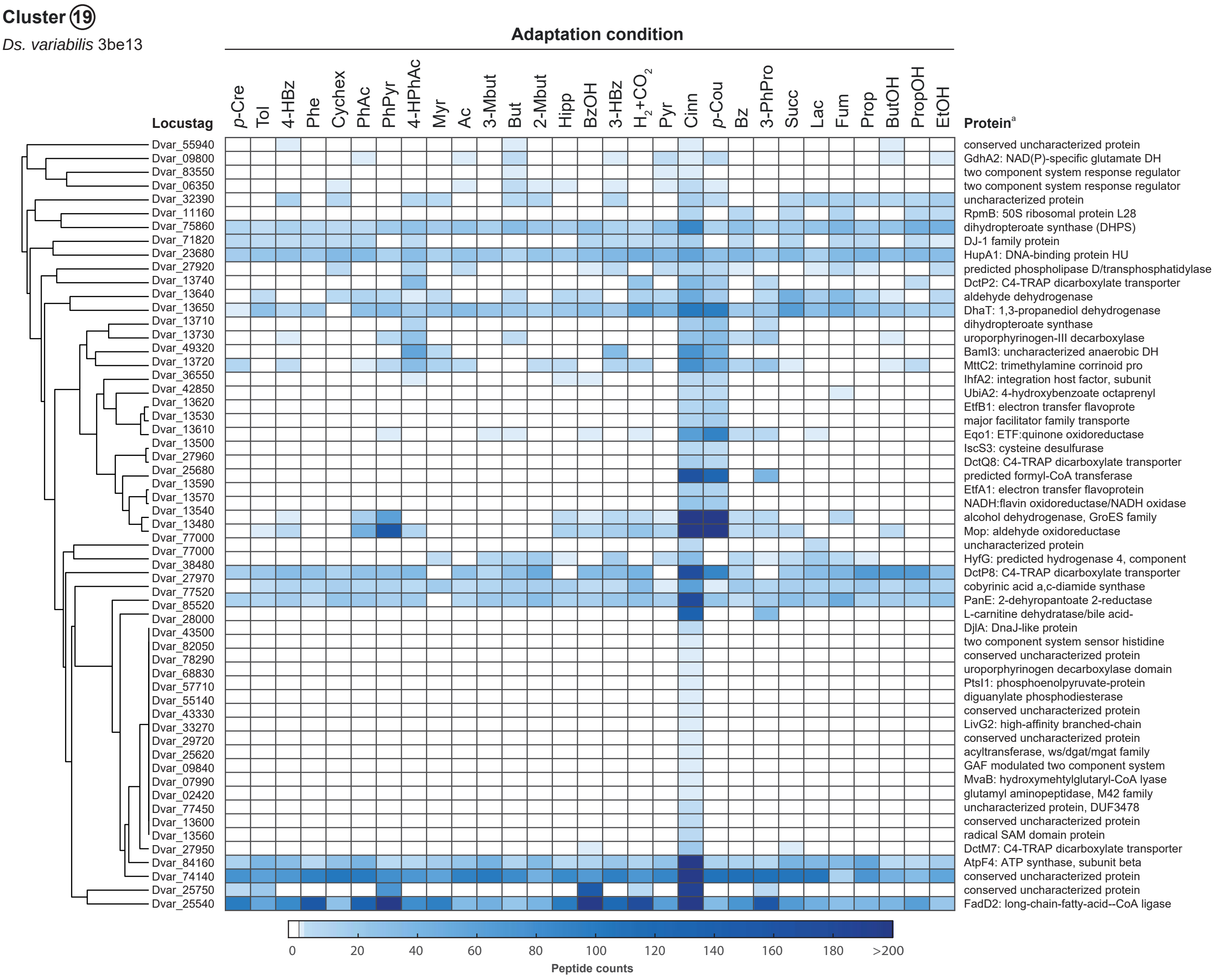

Fig. S44: Detailed view on abundances of proteins forming a sub-cluster in the global clustering depicted in Fig. S6. The global clustering is based on standardized protein abundances of all detected proteins of *Ds. variabilis* 3be13 across the 29 studied substrate adaptation conditions.

[a] Full description of protein functions are available in the genome annotation under accession CP159846.

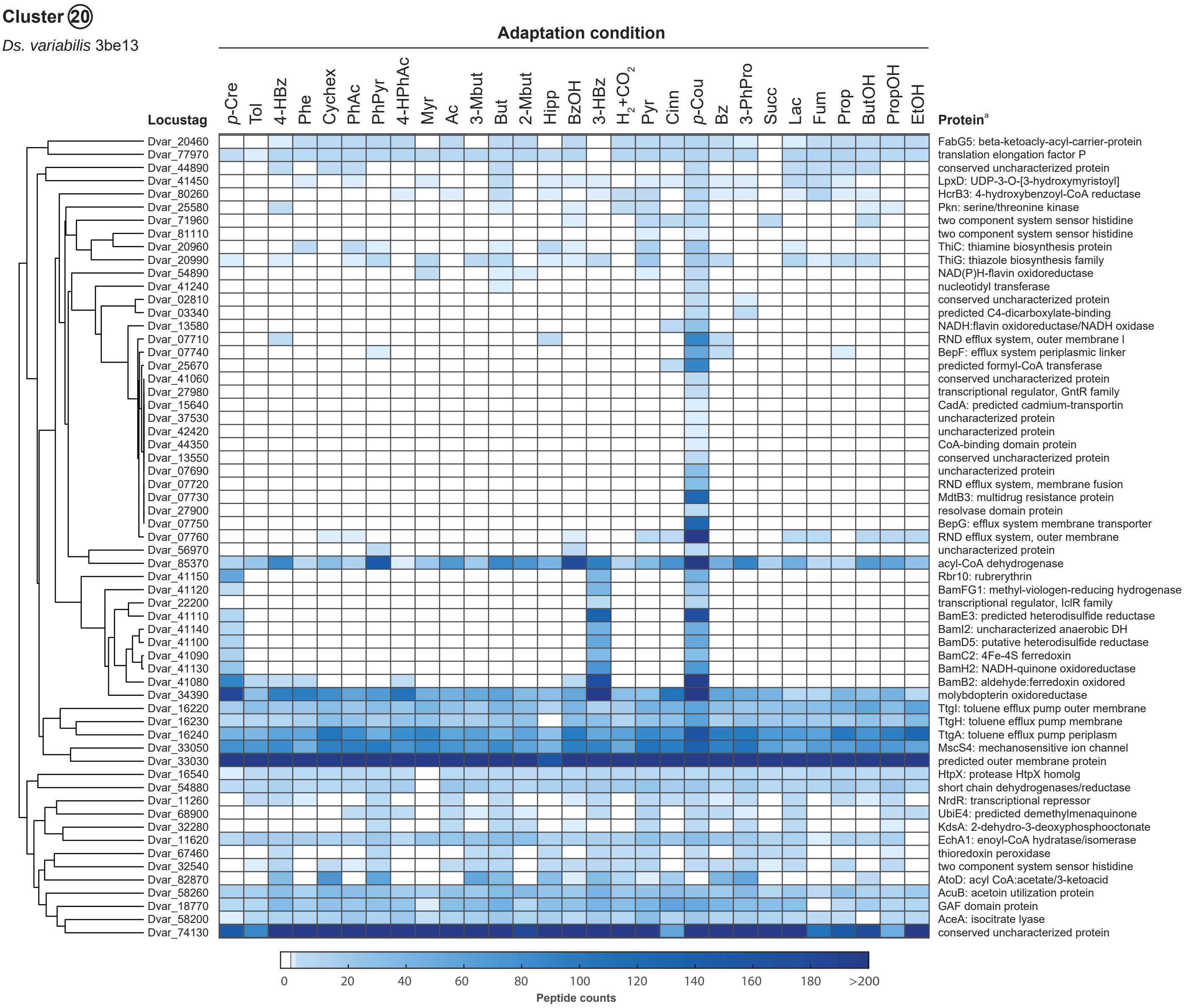

Fig. S45: Detailed view on abundances of proteins forming a sub-cluster in the global clustering depicted in Fig. S6. The global clustering is based on standardized protein abundances of all detected proteins of *Ds. variabilis* 3be13 across the 29 studied substrate adaptation conditions.

[a] Full description of protein functions are available in the genome annotation under accession CP159846.

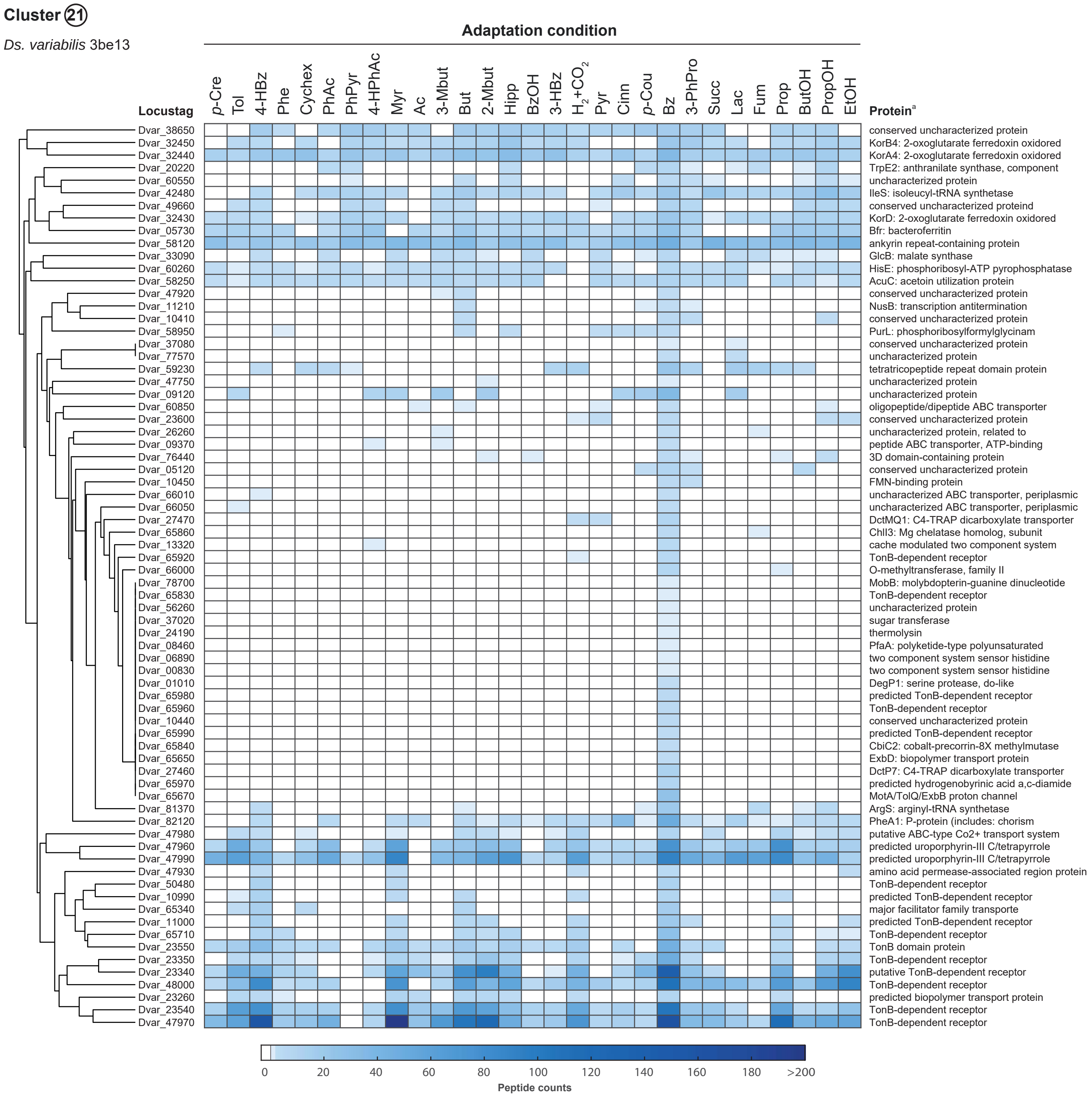

Fig. S46: Detailed view on abundances of proteins forming a sub-cluster in the global clustering depicted in Fig. S6. The global clustering is based on standardized protein abundances of all detected proteins of *Ds. variabilis* 3be13 across the 29 studied substrate adaptation conditions.

[a] Full description of protein functions are available in the genome annotation under accession CP159846.

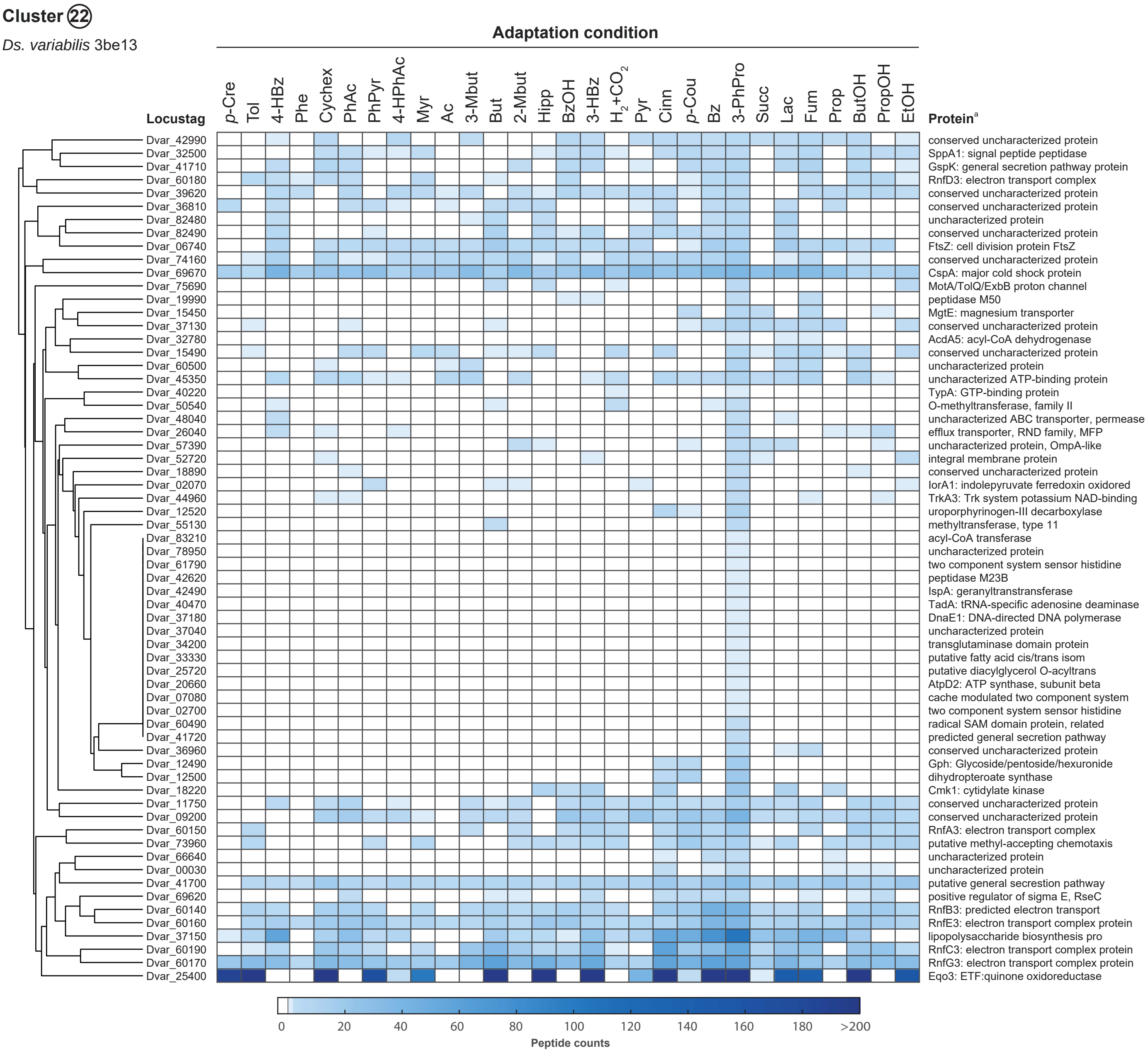

Fig. S47: Detailed view on abundances of proteins forming a sub-cluster in the global clustering depicted in Fig. S6. The global clustering is based on standardized protein abundances of all detected proteins of *Ds. variabilis* 3be13 across the 29 studied substrate adaptation conditions.
[a] Full description of protein functions are available in the genome annotation under accession CP159846.

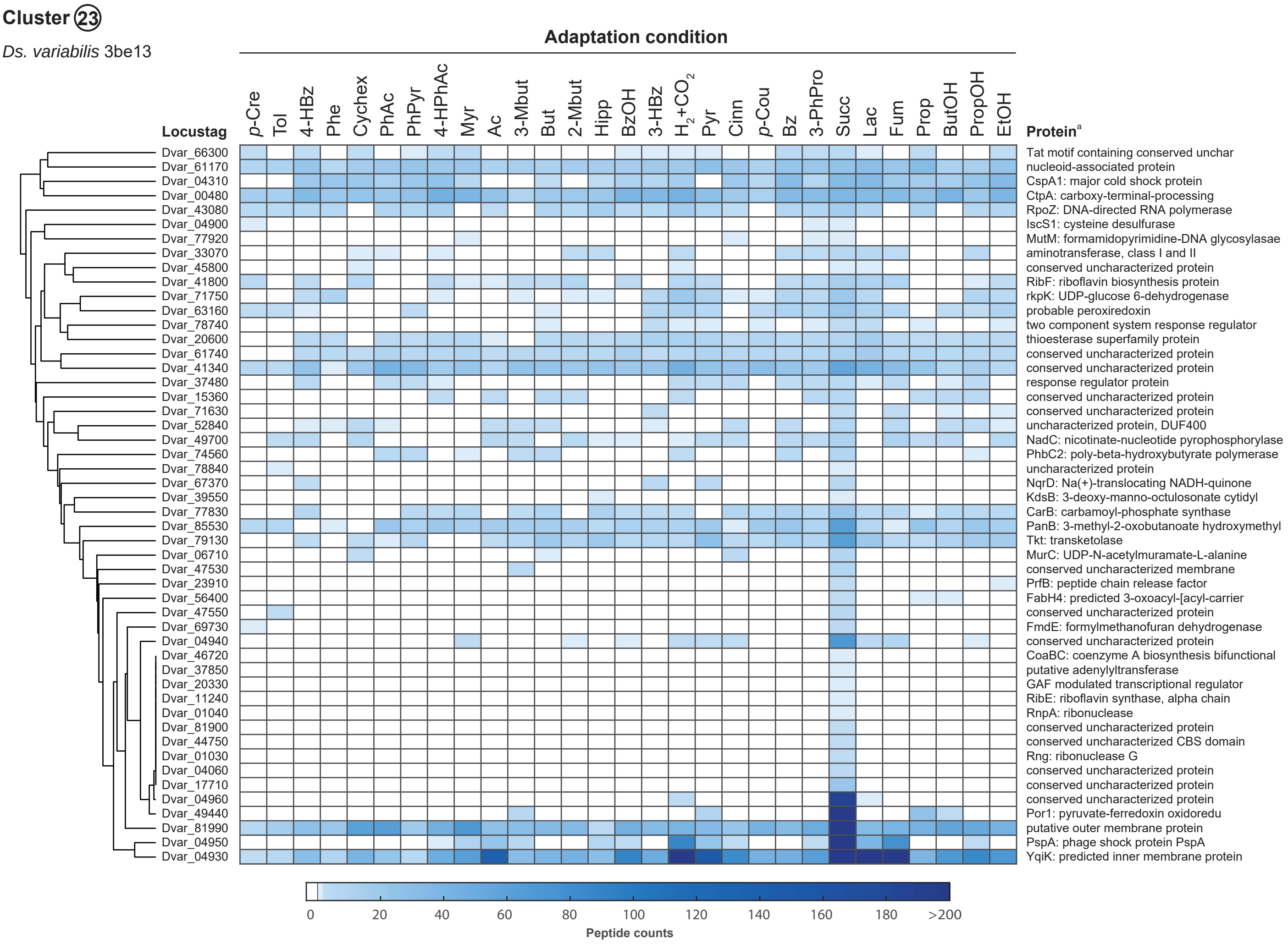

Fig. S48: Detailed view on abundances of proteins forming a sub-cluster in the global clustering depicted in Fig. S6. The global clustering is based on standardized protein abundances of all detected proteins of *Ds. variabilis* 3be13 across the 29 studied substrate adaptation conditions.
[a] Full description of protein functions are available in the genome annotation under accession CP159846.

Cluster 24

Ds. variabilis 3be13

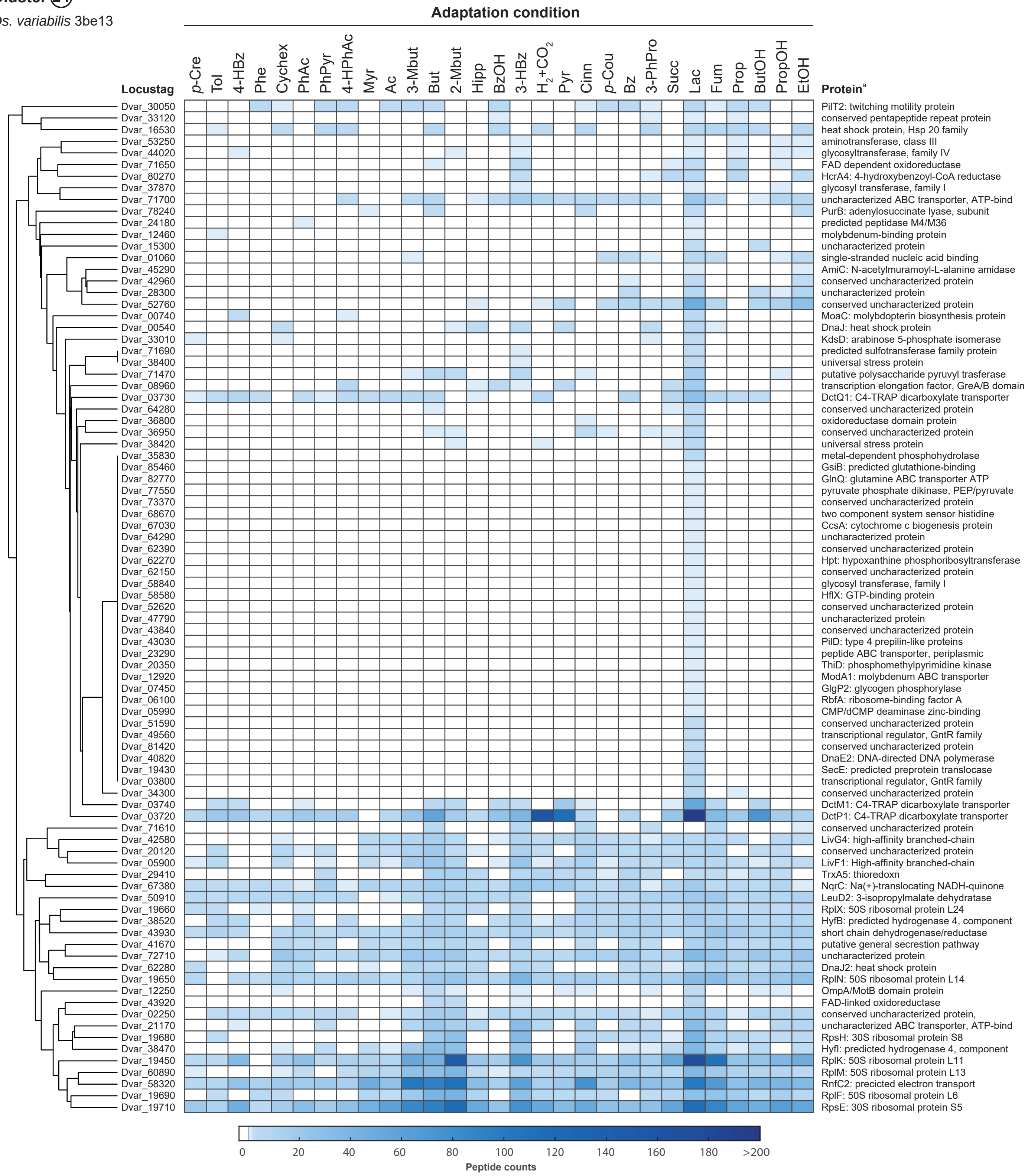

Fig. S49: Detailed view on abundances of proteins forming a sub-cluster in the global clustering depicted in Fig. S6. The global clustering is based on standardized protein abundances of all detected proteins of *Ds. variabilis* 3be13 across the 29 studied substrate adaptation conditions.

[a] Full description of protein functions are available in the genome annotation under accession CP159846.

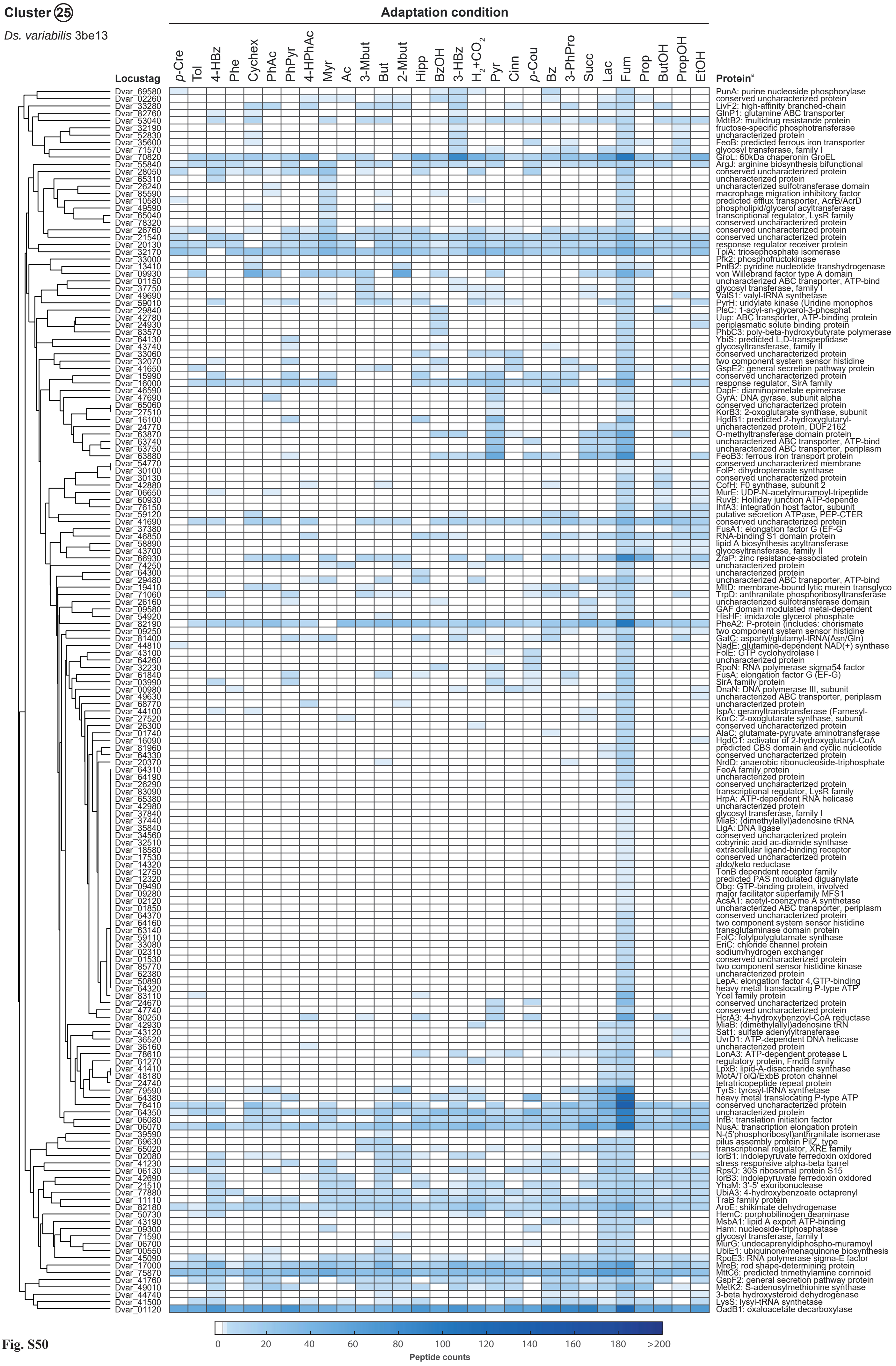
Cluster 25
Ds. variabilis 3be13
Adaptation condition
Locustag
p-Cre
Tol
4-HBz
Phe
Cychex
PhAc
PhPyr
4-HPhAc
Myr
Ac
3-Mbut
But
2-Mbut
Hipp
BzOH
3-HBz
H2+CO2
Pyr
Cinn
p-Cou
Bz
3-PhPro
Succ
Lac
Fum
Prop
ButOH
PropOH
EtOH
Protein[a]
Dvar_69580 PunA: purine nucleoside phosphorylase
Dvar_02260 conserved uncharacterized protein
Dvar_33280 LivF2: high-affinity branched-chain
Dvar_82760 GlnP1: glutamine ABC transporter
Dvar_53040 MdtB2: multidrug resistande protein
Dvar_32190 fructose-specific phosphotransferase
Dvar_52830 uncharacterized protein
Dvar_35600 FeoB: predicted ferrous iron transporter
Dvar_71570 glycosyl transferase, family I
Dvar_70820 GroL: 60kDa chaperonin GroEL
Dvar_55840 ArgJ: arginine biosynthesis bifunctional
Dvar_28050 conserved uncharacterized protein
Dvar_65310 uncharacterized protein
Dvar_26240 uncharacterized sulfotransferase domain
Dvar_85590 macrophage migration inhibitory factor
Dvar_10580 predicted efflux transporter, AcrB/AcrD
Dvar_49590 phospholipid/glycerol acyltransferase
Dvar_65040 transcriptional regulator, LysR family
Dvar_78320 conserved uncharacterized protein
Dvar_26760 conserved uncharacterized protein
Dvar_21540 conserved uncharacterized protein
Dvar_20130 response regulator receiver protein
Dvar_32170 TpiA: triosephosphate isomerase
Dvar_33000 Pfk2: phosphofructokinase
Dvar_13410 PntB2: pyridine nucleotide transhydrogenase
Dvar_09930 von Willebrand factor type A domain
Dvar_01150 uncharacterized ABC transporter, ATP-bind
Dvar_37750 glycosyl transferase, family I
Dvar_49690 ValS1: valyl-tRNA synthetase
Dvar_59010 PyrH: uridylate kinase (Uridine monophos
Dvar_29840 PlsC: 1-acyl-sn-glycerol-3-phosphat
Dvar_42780 Uup: ABC transporter, ATP-binding protein
Dvar_24930 periplasmatic solute binding protein
Dvar_83570 PhbC3: poly-beta-hydroxybutyrate polymerase
Dvar_64130 YbiS: predicted L,D-transpeptidase
Dvar_43740 glycosyltransferase, family II
Dvar_33060 conserved uncharacterized protein
Dvar_32070 two component system sensor histidine
Dvar_41650 GspE2: general secretion pathway protein
Dvar_15990 conserved uncharacterized protein
Dvar_16000 response regulator, SirA family
Dvar_46590 DapF: diaminopimelate epimerase
Dvar_47690 GyrA: DNA gyrase, subunit alpha
Dvar_65060 conserved uncharacterized protein
Dvar_27510 KorB3: 2-oxoglutarate synthase, subunit
Dvar_16100 HgdB1: predicted 2-hydroxyglutaryl-
Dvar_24770 uncharacterized protein, DUF2162
Dvar_63870 O-methyltransferase domain protein
Dvar_63740 uncharacterized ABC transporter, ATP-bind
Dvar_63750 uncharacterized ABC transporter, periplasm
Dvar_63880 FeoB3: ferrous iron transport protein
Dvar_54770 conserved uncharacterized membrane
Dvar_30100 FolP: dihydropteroate synthase
Dvar_30130 conserved uncharacterized protein
Dvar_42880 CofH: F0 synthase, subunit 2
Dvar_06650 MurE: UDP-N-acetylmuramoyl-tripeptide
Dvar_60930 RuvB: Holliday junction ATP-depende
Dvar_76150 IhfA3: integration host factor, subunit
Dvar_59120 putative secretion ATPase, PEP-CTER
Dvar_41690 conserved uncharacterized protein
Dvar_37380 FusA1: elongation factor G (EF-G
Dvar_46850 RNA-binding S1 domain protein
Dvar_58890 lipid A biosynthesis acyltransferase
Dvar_43700 glycosyltransferase, family II
Dvar_66930 ZraP: zinc resistance-associated protein
Dvar_74250 uncharacterized protein
Dvar_64300 uncharacterized protein
Dvar_29480 uncharacterized ABC transporter, ATP-bind
Dvar_19410 MltD: membrane-bound lytic murein transglyco
Dvar_71060 TrpD: anthranilate phosphoribosyltransferase
Dvar_26160 uncharacterized sulfotransferase domain
Dvar_09580 GAF domain modulated metal-dependent
Dvar_54920 HisHF: imidazole glycerol phosphate
Dvar_82190 PheA2: P-protein (includes: chorismate
Dvar_09250 two component system sensor histidine
Dvar_81400 GatC: aspartyl/glutamyl-tRNA(Asn/Gln)
Dvar_44810 NadE: glutamine-dependent NAD(+) synthase
Dvar_43100 FolE: GTP cyclohydrolase I
Dvar_64260 uncharacterized protein
Dvar_32230 RpoN: RNA polymerase sigma54 factor
Dvar_61840 FusA: elongation factor G (EF-G)
Dvar_03990 SirA family protein
Dvar_00980 DnaN: DNA polymerase III, subunit
Dvar_49630 uncharacterized ABC transporter, periplasm
Dvar_68770 uncharacterized protein
Dvar_44100 IspA: geranyltranstransferase (Farnesyl-
Dvar_27520 KorC: 2-oxoglutarate synthase, subunit
Dvar_26300 conserved uncharacterized protein
Dvar_01740 AlaC: glutamate-pyruvate aminotransferase
Dvar_16090 HgdC1: activator of 2-hydroxyglutaryl-CoA
Dvar_81960 predicted CBS domain and cyclic nucleotide
Dvar_64330 conserved uncharacterized protein
Dvar_20370 NrdD: anaerobic ribonucleoside-triphosphate
Dvar_64310 FeoA family protein
Dvar_64190 uncharacterized protein
Dvar_26290 conserved uncharacterized protein
Dvar_83090 transcriptional regulator, LysR family
Dvar_65380 HrpA: ATP-dependent RNA helicase
Dvar_42980 uncharacterized protein
Dvar_37840 glycosyl transferase, family I
Dvar_37440 MiaB: (dimethylallyl)adenosine tRNA
Dvar_35840 LigA: DNA ligase
Dvar_34560 conserved uncharacterized protein
Dvar_32510 cobyrinic acid ac-diamide synthase
Dvar_18580 extracellular ligand-binding receptor
Dvar_17530 conserved uncharacterized protein
Dvar_14320 aldo/keto reductase
Dvar_12750 TonB dependent receptor family
Dvar_12320 predicted PAS modulated diguanylate
Dvar_09490 Obg: GTP-binding protein, involved
Dvar_09280 major facilitator superfamily MFS1
Dvar_02120 AcsA1: acetyl-coenzyme A synthetase
Dvar_01850 uncharacterized ABC transporter, periplasm
Dvar_64370 conserved uncharacterized protein
Dvar_64160 two component system sensor histidine
Dvar_63140 transglutaminase domain protein
Dvar_59110 FolC: folylpolyglutamate synthase
Dvar_33080 EriC: chloride channel protein
Dvar_02310 sodium/hydrogen exchanger
Dvar_01530 conserved uncharacterized protein
Dvar_85770 two component sensor histidine kinase
Dvar_62380 uncharacterized protein
Dvar_50890 LepA: elongation factor 4,GTP-binding
Dvar_64320 heavy metal translocating P-type ATP
Dvar_83110 YceI family protein
Dvar_24670 conserved uncharacterized protein
Dvar_47740 conserved uncharacterized protein
Dvar_80250 HcrA3: 4-hydroxybenzoyl-CoA reductase
Dvar_42930 MiaB: (dimethylallyl)adenosine tRN
Dvar_43120 Sat1: sulfate adenylyltransferase
Dvar_36520 UvrD1: ATP-dependent DNA helicase
Dvar_36160 uncharacterized protein
Dvar_78610 LonA3: ATP-dependent protease L
Dvar_61270 regulatory protein, FmdB family
Dvar_41410 LpxB: lipid-A-disaccharide synthase
Dvar_48180 MotA/TolQ/ExbB proton channel
Dvar_24740 tetratricopeptide repeat protein
Dvar_79590 TyrS: tyrosyl-tRNA synthetase
Dvar_64380 heavy metal translocating P-type ATP
Dvar_76410 conserved uncharacterized protein
Dvar_64350 uncharacterized protein
Dvar_06080 InfB: translation initiation factor
Dvar_06070 NusA: transcription elongation protein
Dvar_39590 N-(5'phosphoribosyl)anthranilate isomerase
Dvar_69630 pilus assembly protein PilZ, type
Dvar_65020 transcriptional regulator, XRE family
Dvar_02080 IorB1: indolepyruvate ferredoxin oxidored
Dvar_41230 stress responsive alpha-beta barrel
Dvar_06130 RpsO: 30S ribosomal protein S15
Dvar_42690 IorB3: indolepyruvate ferredoxin oxidored
Dvar_21510 YhaM: 3'-5' exoribonuclease
Dvar_77880 UbiA3: 4-hydroxybenzoate octaprenyl
Dvar_11110 TraB family protein
Dvar_82180 AroE: shikimate dehydrogenase
Dvar_50730 HemC: porphobilinogen deaminase
Dvar_43190 MsbA1: lipid A export ATP-binding
Dvar_09300 Ham: nucleoside-triphosphatase
Dvar_71590 glycosyl transferase, family I
Dvar_06700 MurG: undecaprenyldiphospho-muramoyl
Dvar_00550 UbiE1: ubiquinone/menaquinone biosynthesis
Dvar_45090 RpoE3: RNA polymerase sigma-E factor
Dvar_17000 MreB: rod shape-determining protein
Dvar_75870 MttC6: predicted trimethylamine corrinoid
Dvar_41760 GspF2: general secretion pathway protein
Dvar_49010 MetK2: S-adenosylmethionine synthase
Dvar_44740 3-beta hydroxysteroid dehydrogenase
Dvar_41500 LysS: lysyl-tRNA synthetase
Dvar_01120 OadB1: oxaloacetate decarboxylase
0
20
40
60
80
100
120
140
160
180
>200
Peptide counts

Fig. S50

Cluster 26

Ds. variabilis 3be13

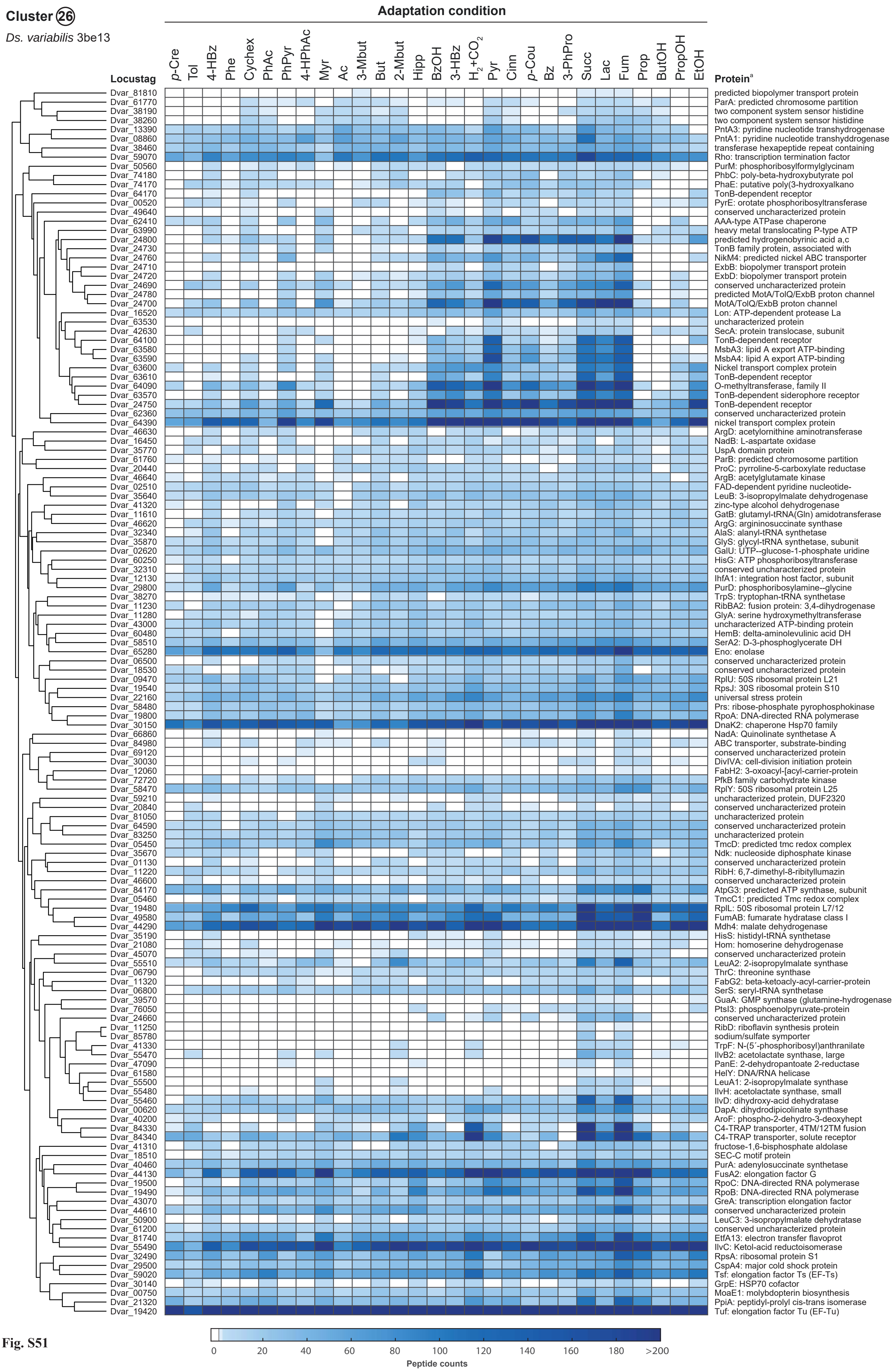

Fig. S51

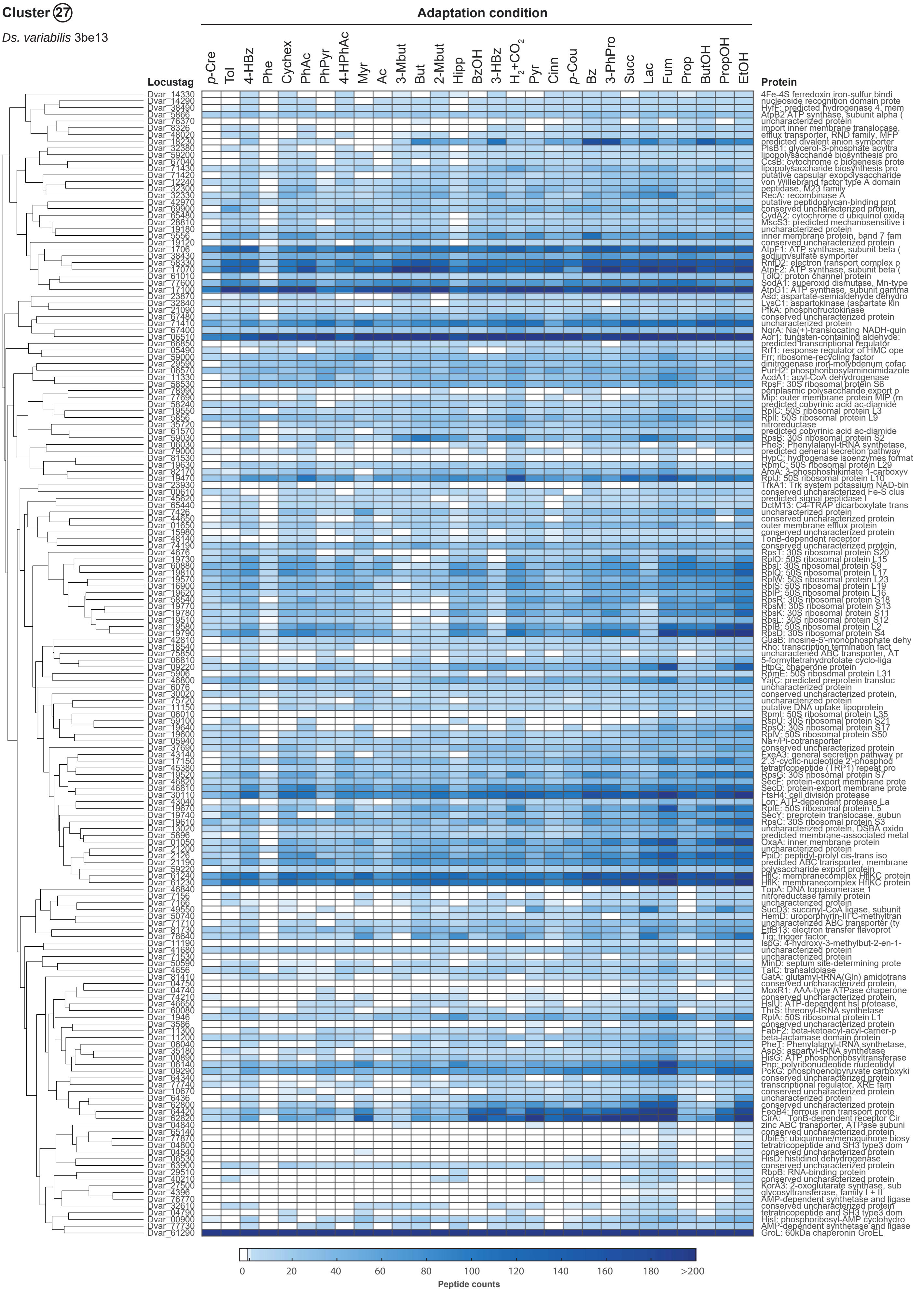

Fig. S52: Detailed view on abundances of proteins forming a sub-cluster in the global clustering depicted in Fig. S6. The global clustering is based on standardized protein abundances of all detected proteins of *Ds. variabilis* 3be13 across the 29 studied substrate adaptation conditions.

[a] Full description of protein functions are available in the genome annotation under accession CP159846.

Cluster 28

Ds. variabilis 3be13

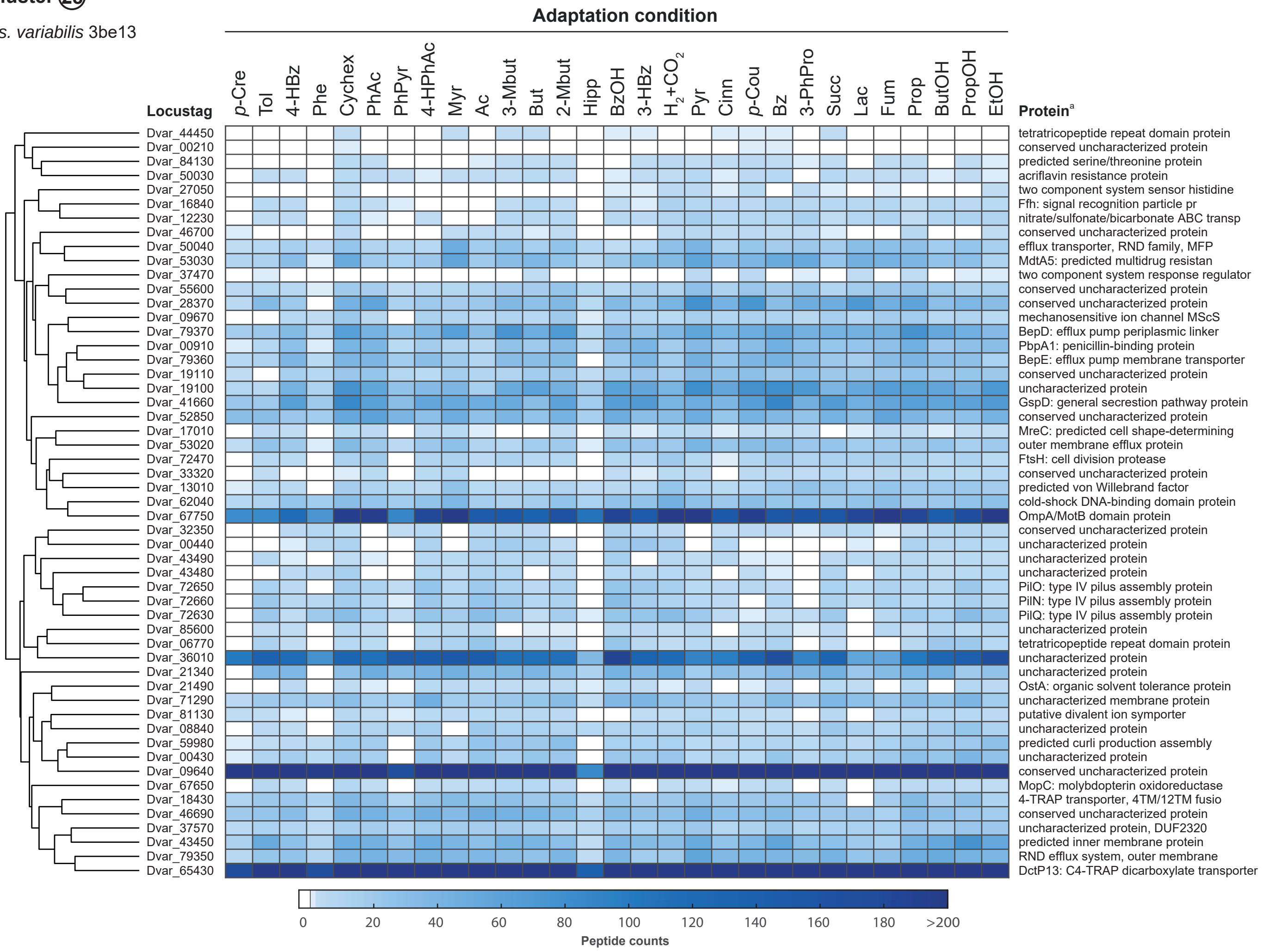

Fig. S53: Detailed view on abundances of proteins forming a sub-cluster in the global clustering depicted in Fig. S6. The global clustering is based on standardized protein abundances of all detected proteins of *Ds. variabilis* 3be13 across the 29 studied substrate adaptation conditions.

[a] Full description of protein functions are available in the genome annotation under accession CP159846.

Cluster 29

Ds. variabilis 3be13

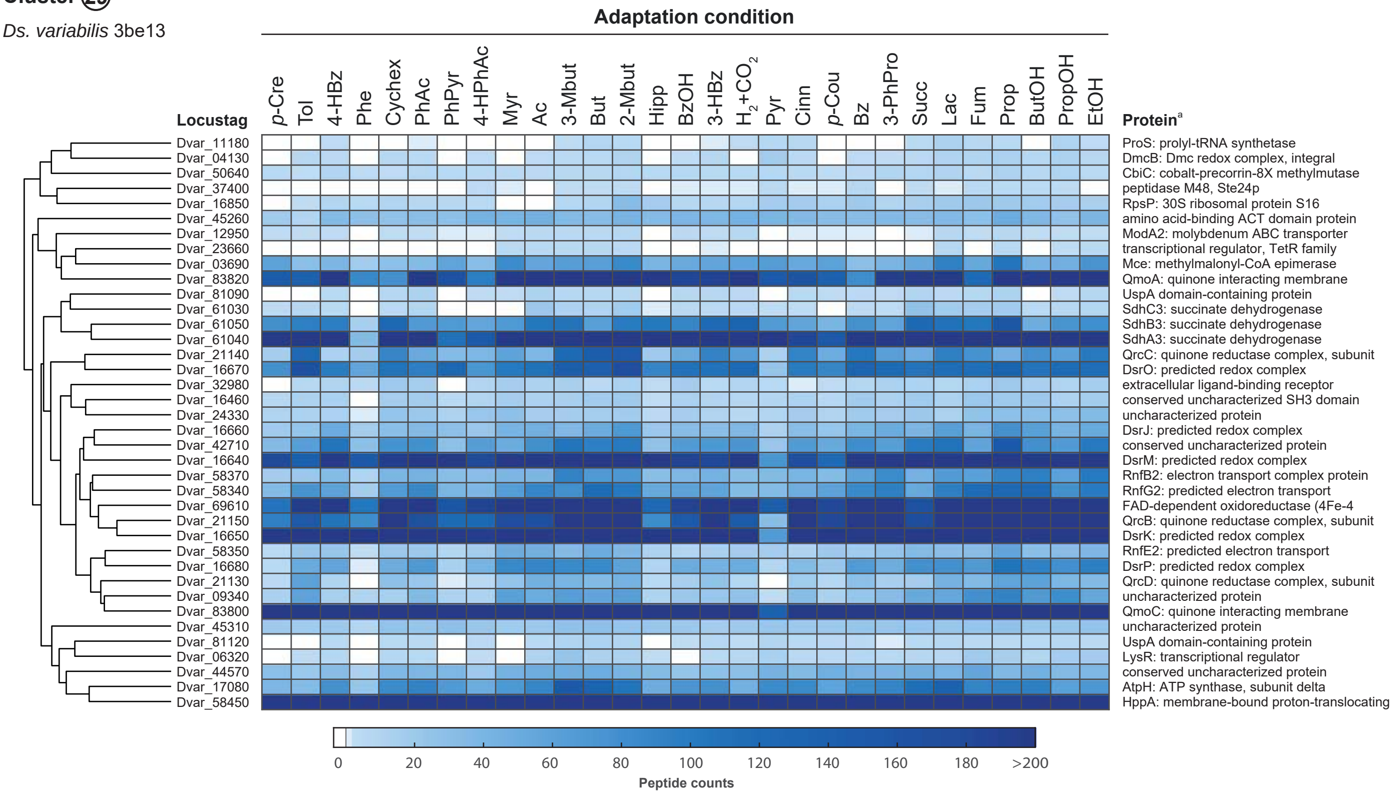

Fig. S54: Detailed view on abundances of proteins forming a sub-cluster in the global clustering depicted in Fig. S6. The global clustering is based on standardized protein abundances of all detected proteins of *Ds. variabilis* 3be13 across the 29 studied substrate adaptation conditions.

[a] Full description of protein functions are available in the genome annotation under accession CP159846.

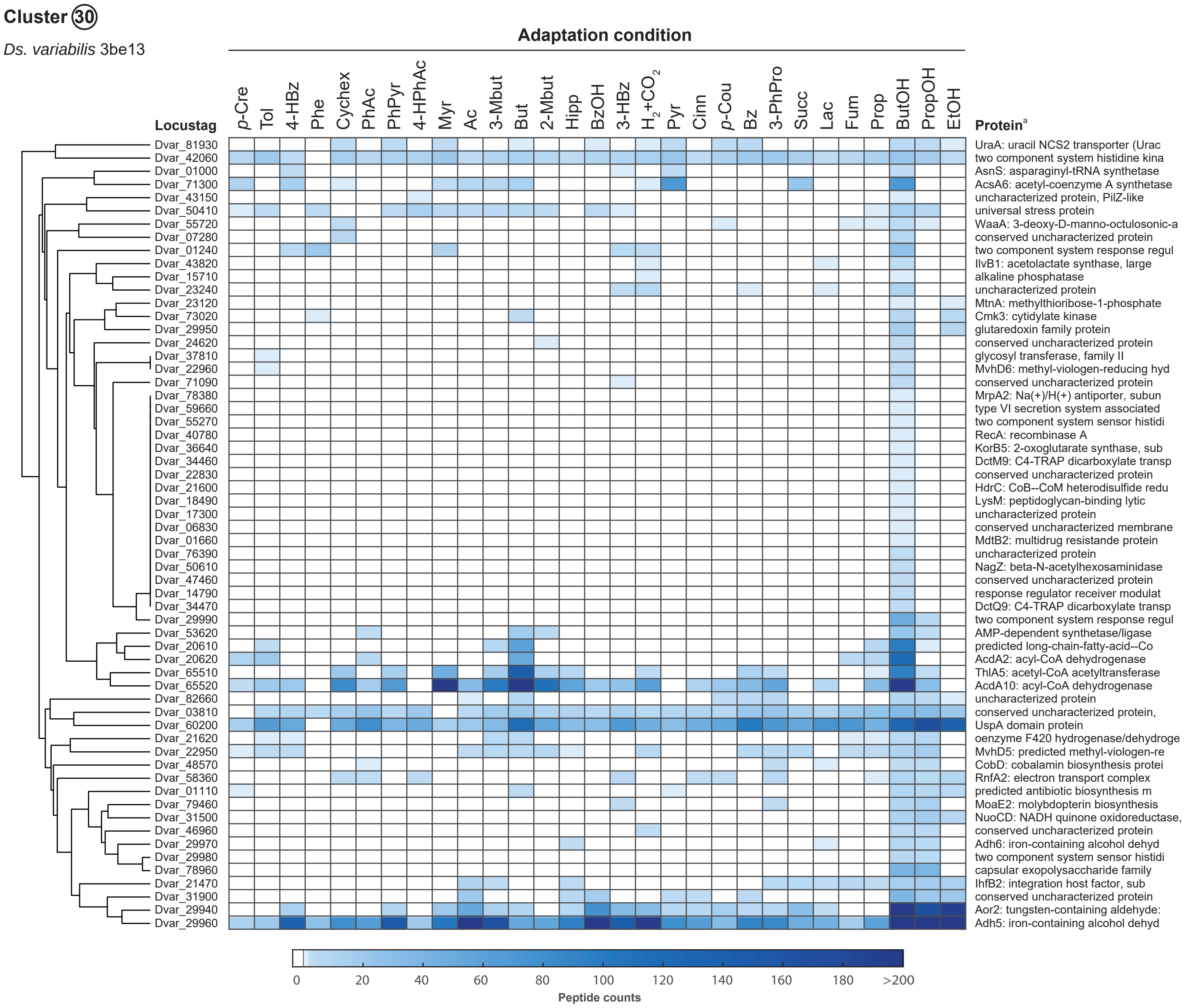

Fig. S55: Detailed view on abundances of proteins forming a sub-cluster in the global clustering depicted in Fig. S6. The global clustering is based on standardized protein abundances of all detected proteins of *Ds. variabilis* 3be13 across the 29 studied substrate adaptation conditions.

[a] Full description of protein functions are available in the genome annotation under accession CP159846.

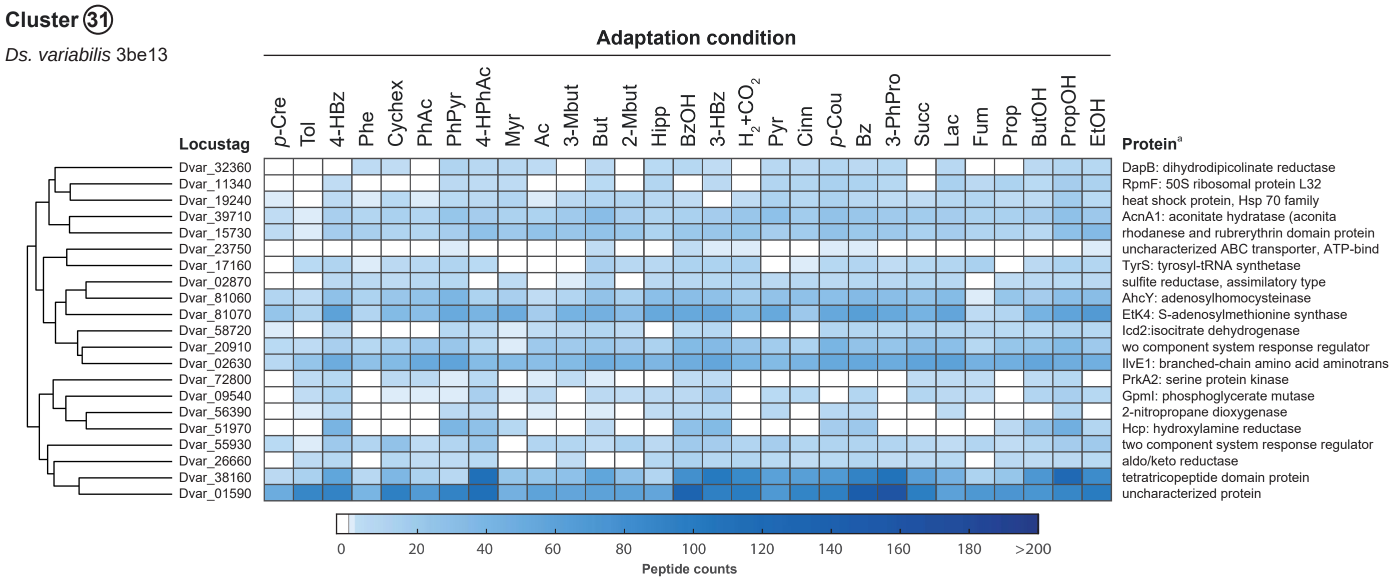

Fig. S56: Detailed view on abundances of proteins forming a sub-cluster in the global clustering depicted in Fig. S6. The global clustering is based on standardized protein abundances of all detected proteins of *Ds. variabilis* 3be13 across the 29 studied substrate adaptation conditions.
[a] Full description of protein functions are available in the genome annotation under accession CP159846.

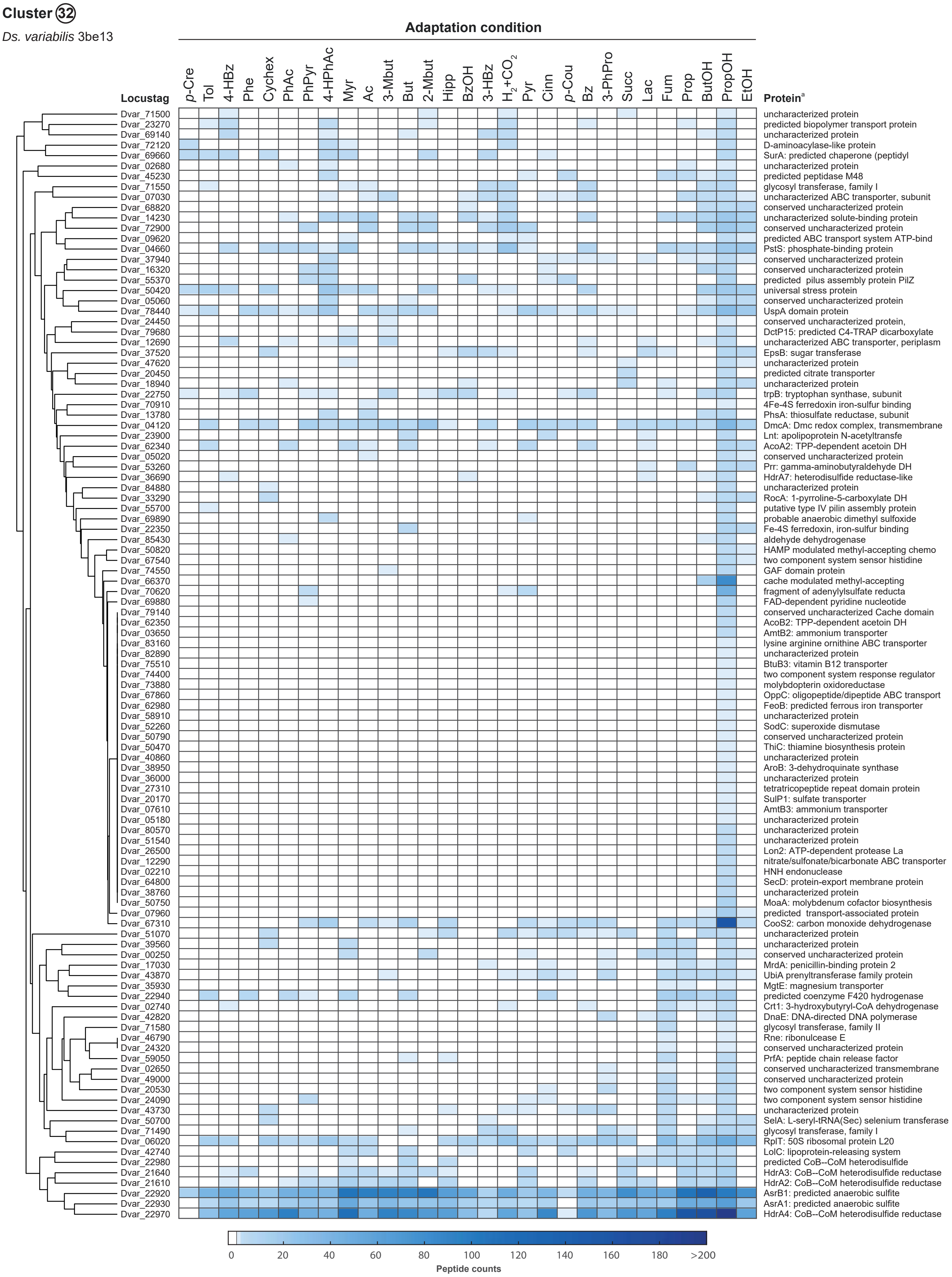

Fig. S57: Detailed view on abundances of proteins forming a sub-cluster in the global clustering depicted in Fig. S6. The global clustering is based on standardized protein abundances of all detected proteins of *Ds. variabilis* 3be13 across the 29 studied substrate adaptation conditions.

[a] Full description of protein functions are available in the genome annotation under accession CP159846.

Cluster 33

Ds. variabilis 3be13

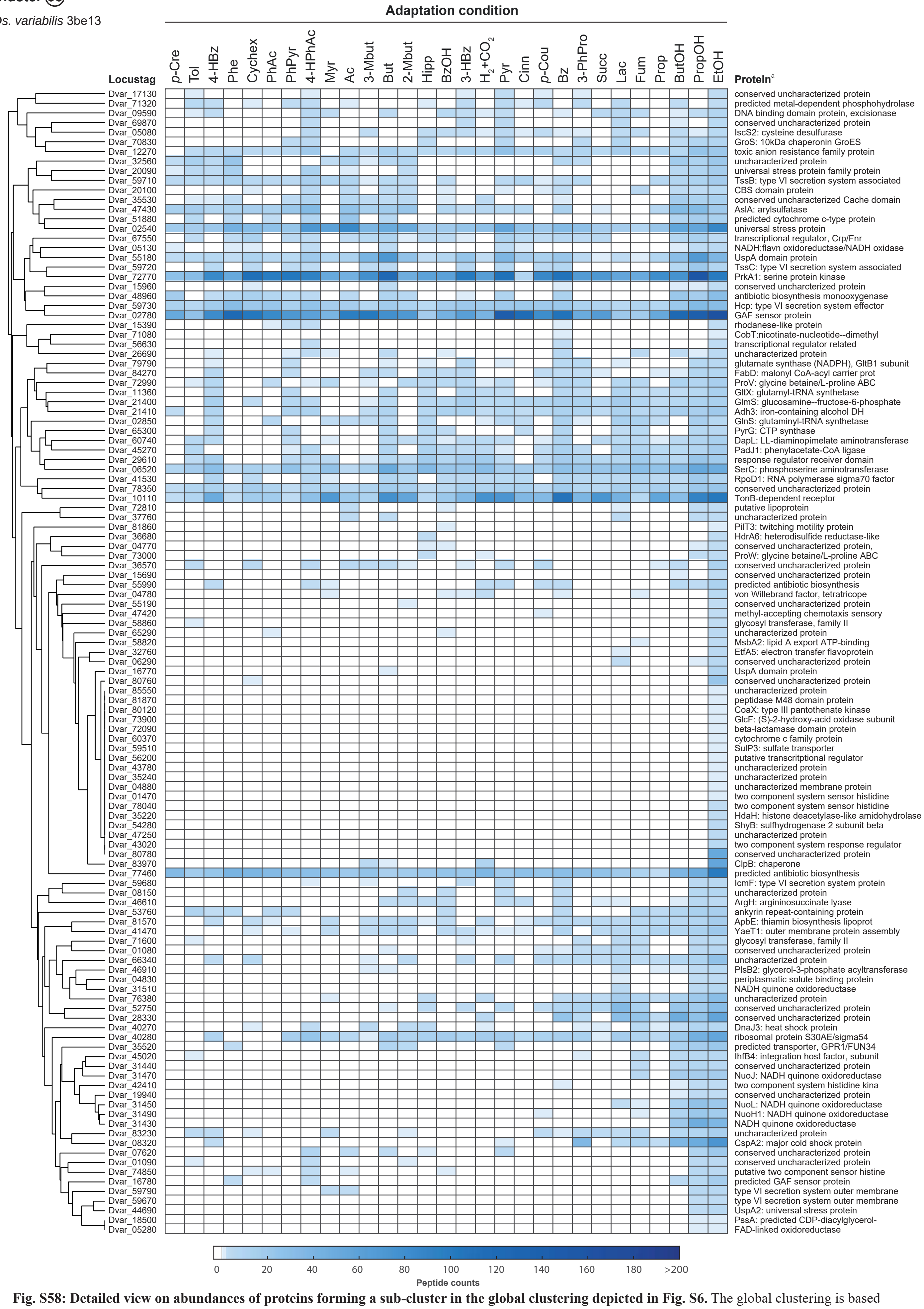

Fig. S58: Detailed view on abundances of proteins forming a sub-cluster in the global clustering depicted in Fig. S6. The global clustering is based on standardized protein abundances of all detected proteins of *Ds. variabilis* 3be13 across the 29 studied substrate adaptation conditions.

[a] Full description of protein functions are available in the genome annotation under accession CP159846.

Cluster 1

Dn. magnum

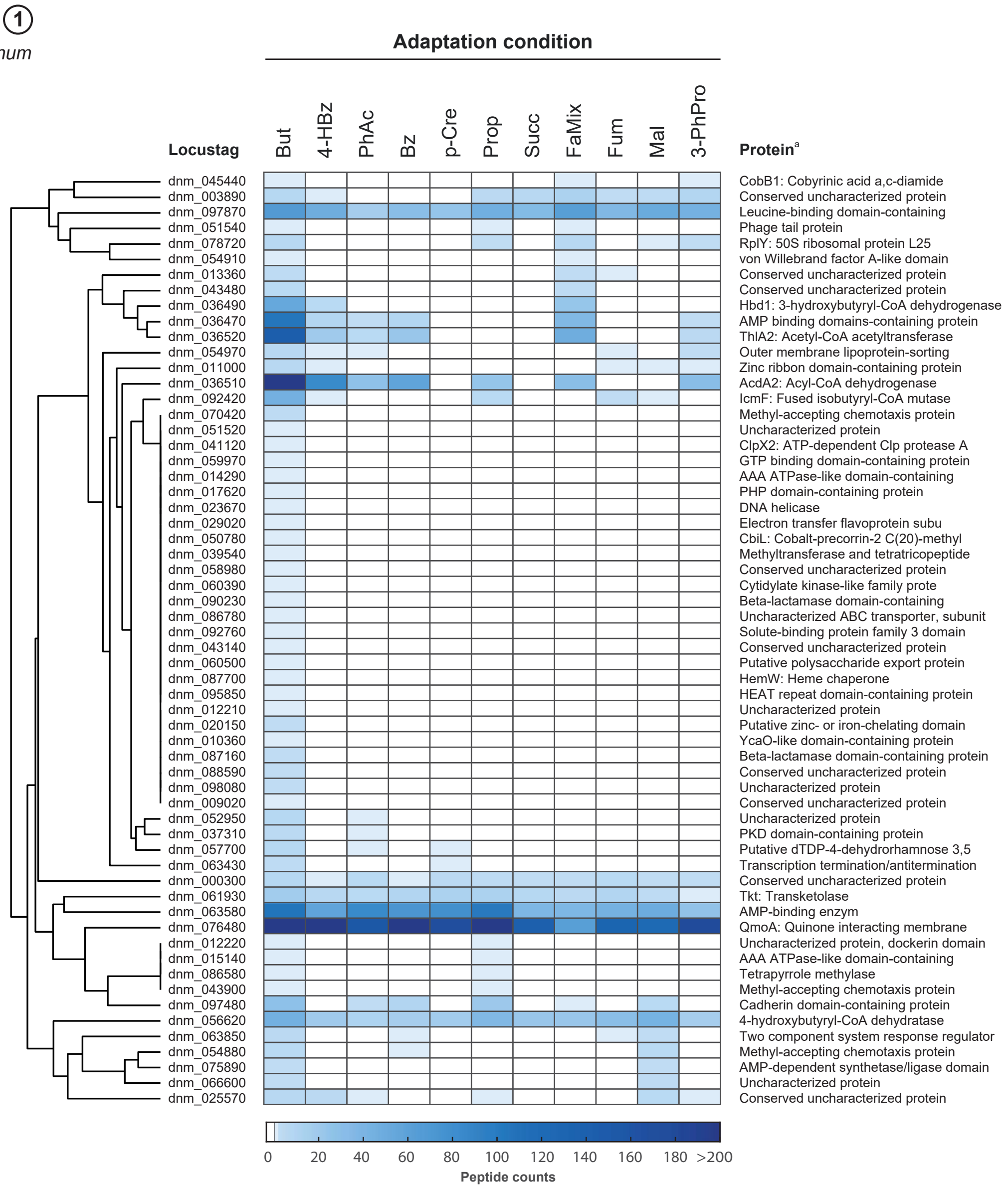

Fig. S59: Detailed view on abundances of proteins forming a sub-cluster in the global clustering depicted in Fig. S7. The global clustering is based on standardized protein abundances of all detected proteins of *Dn. magnum* across the 11 studied substrate adaptation conditions.

[a] Full description of protein functions are available in the genome annotation under accession CP061800.

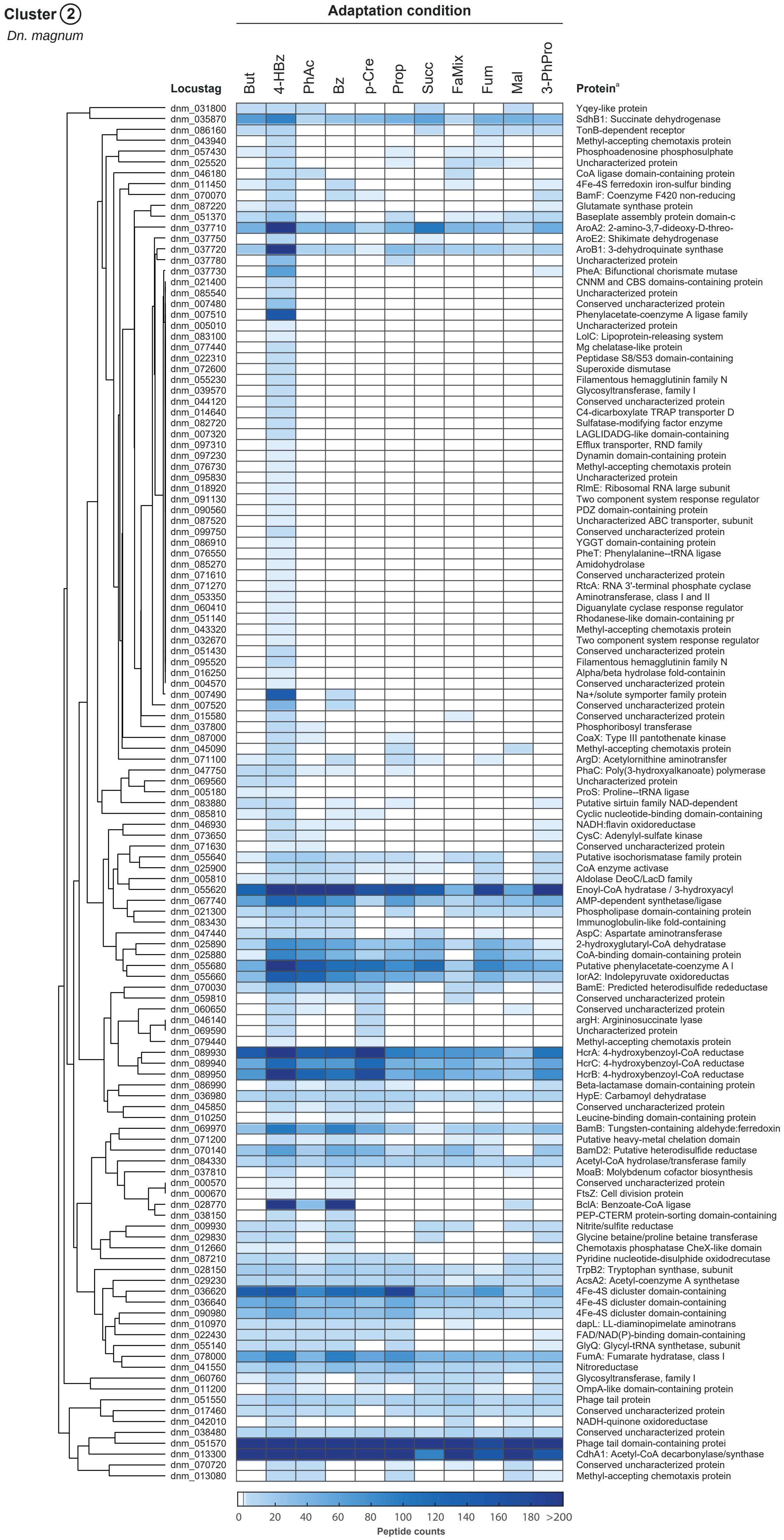

Fig. S60: Detailed view on abundances of proteins forming a sub-cluster in the global clustering depicted in Fig. S7. The global clustering is based on standardized protein abundances of all detected proteins of *Dn. magnum* across the 11 studied substrate adaptation conditions.

[a] Full description of protein functions are available in the genome annotation under accession CP061800.

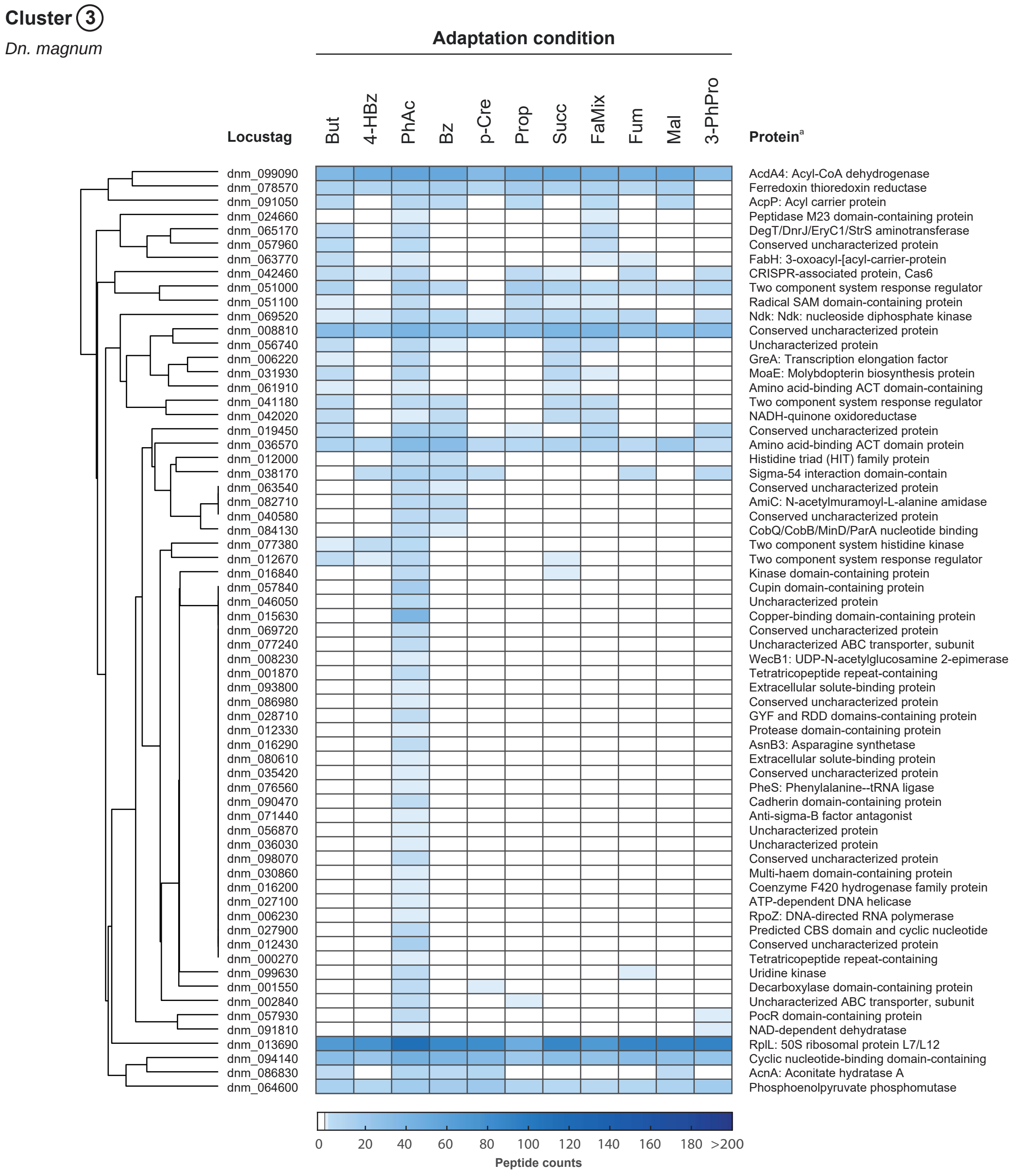

Fig. S61: Detailed view on abundances of proteins forming a sub-cluster in the global clustering depicted in Fig. S7. The global clustering is based on standardized protein abundances of all detected proteins of *Dn. magnum* across the 11 studied substrate adaptation conditions.

[a] Full description of protein functions are available in the genome annotation under accession CP061800.

Cluster 4
Dn. magnum

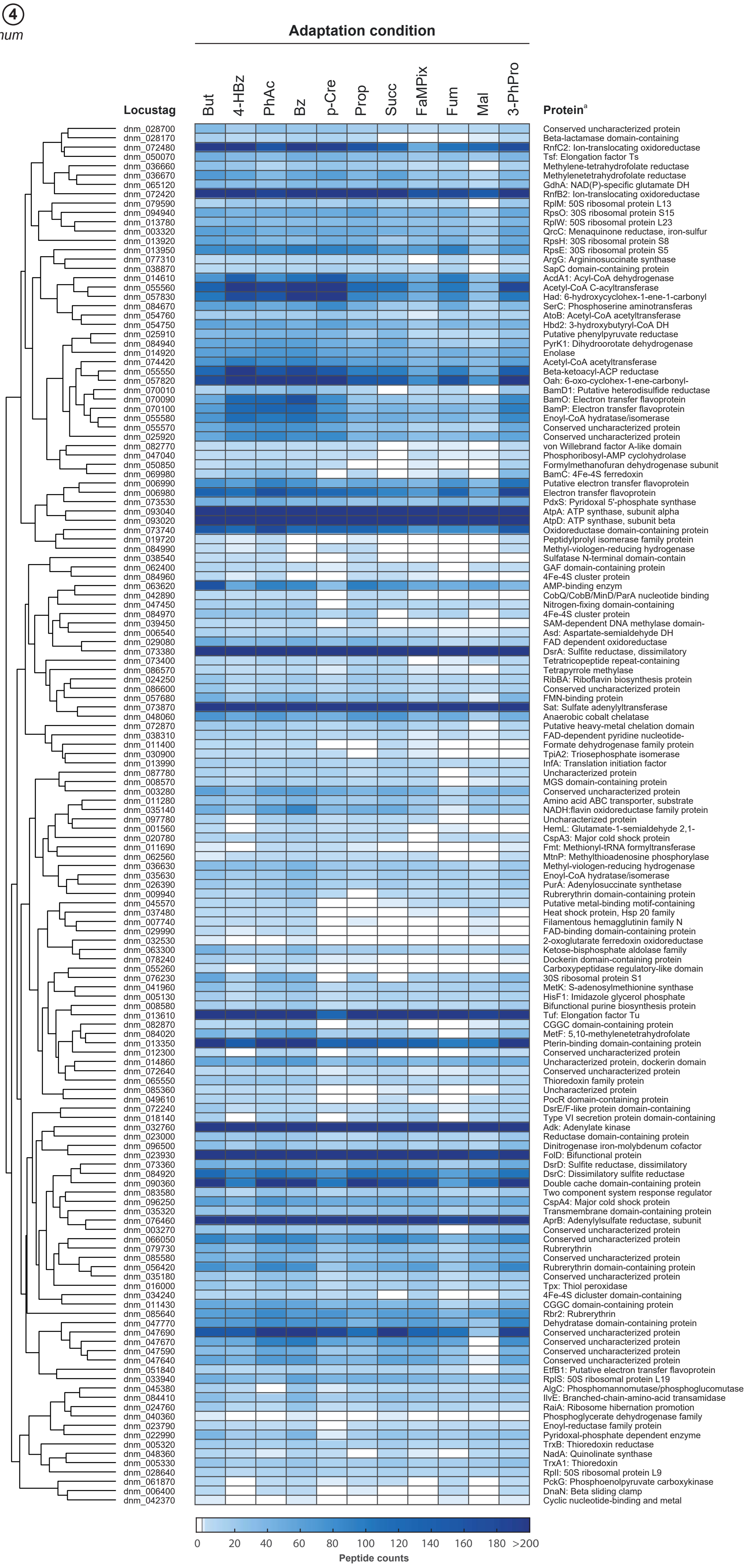

Fig. S62: Detailed view on abundances of proteins forming a sub-cluster in the global clustering depicted in Fig. S7. The global clustering is based on standardized protein abundances of all detected proteins of *Dn. magnum* across the 11 studied substrate adaptation conditions.

[a] Full description of protein functions are available in the genome annotation under accession CP061800.

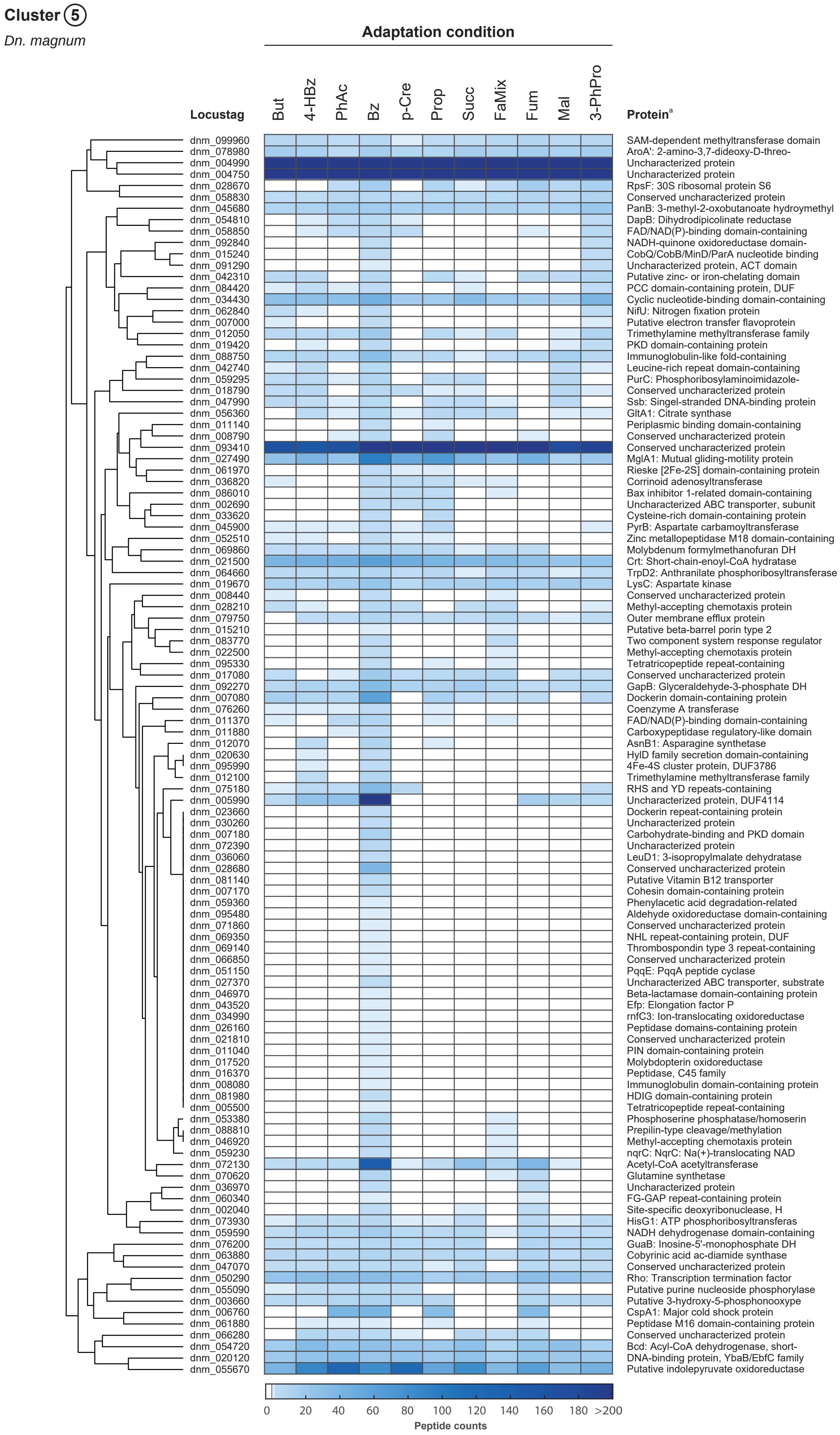

Fig. S63: Detailed view on abundances of proteins forming a sub-cluster in the global clustering depicted in Fig. S7. The global clustering is based on standardized protein abundances of all detected proteins of *Dn. magnum* across the 11 studied substrate adaptation conditions.

[a] Full description of protein functions are available in the genome annotation under accession CP061800.

Cluster 6

Dn. magnum

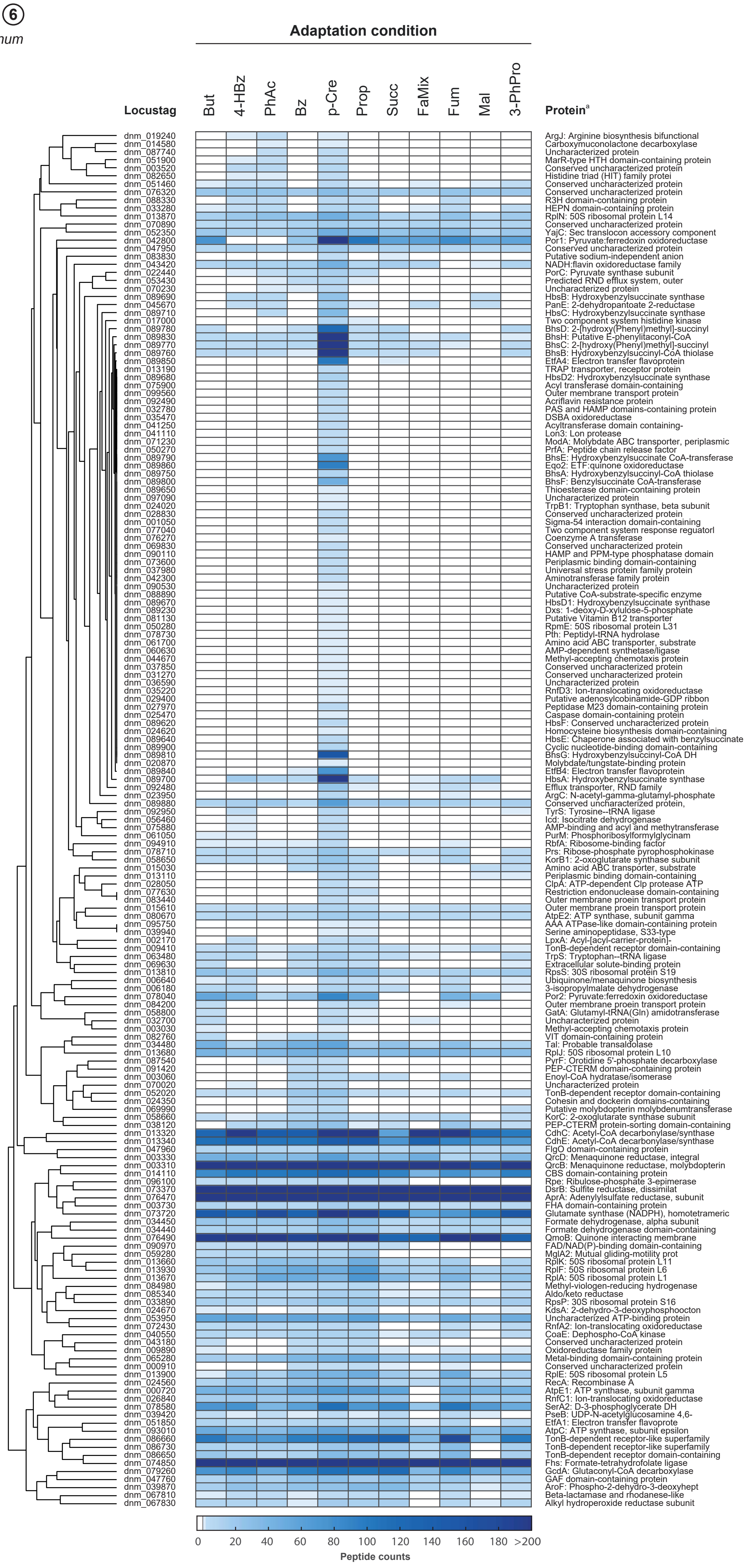

Fig. S64: Detailed view on abundances of proteins forming a sub-cluster in the global clustering depicted in Fig. S7. The global clustering is based on standardized protein abundances of all detected proteins of *Dn. magnum* across the 11 studied substrate adaptation conditions.

[a] Full description of protein functions are available in the genome annotation under accession CP061800.

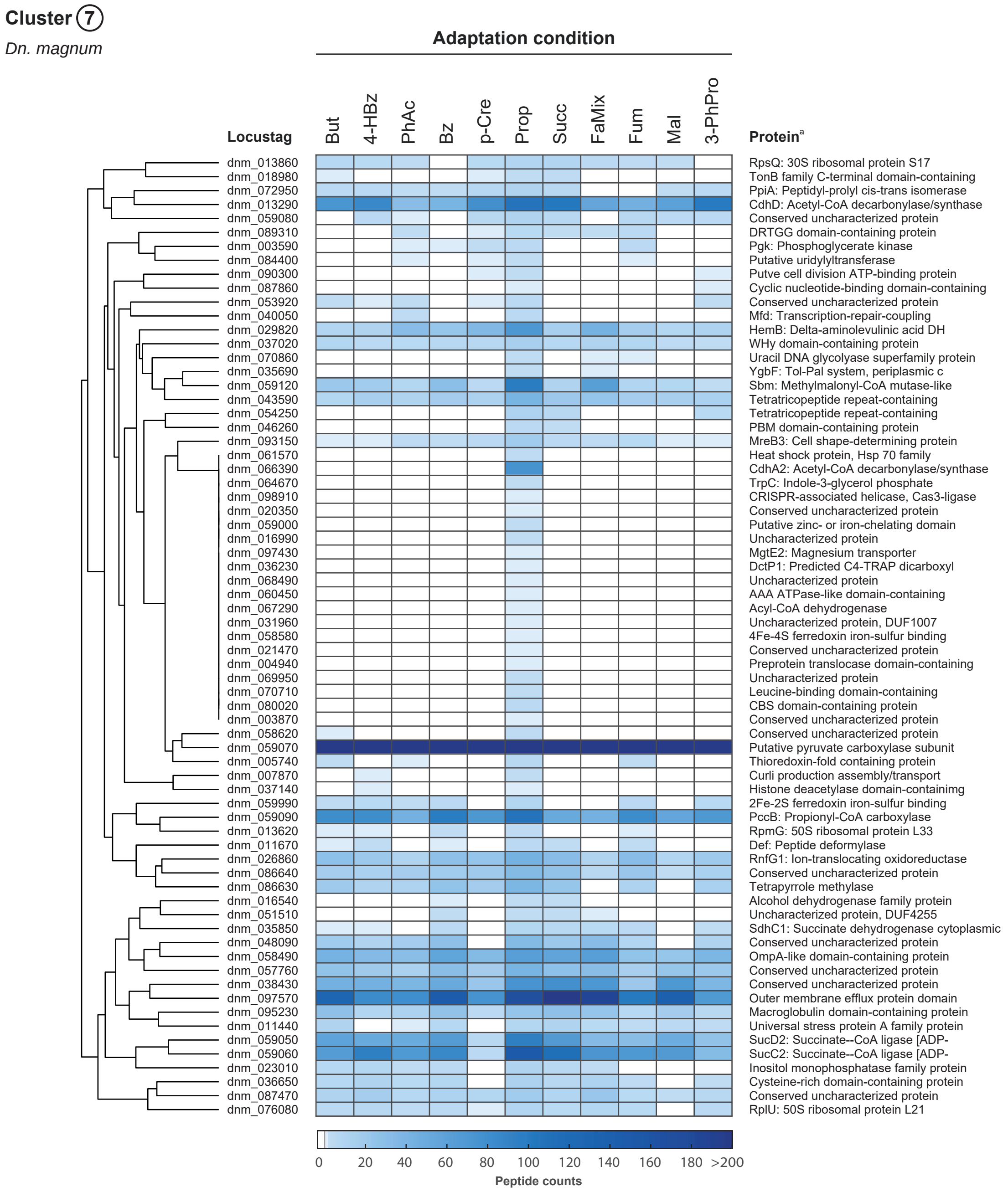

Fig. S65: Detailed view on abundances of proteins forming a sub-cluster in the global clustering depicted in Fig. S7. The global clustering is based on standardized protein abundances of all detected proteins of *Dn. magnum* across the 11 studied substrate adaptation conditions.

[a] Full description of protein functions are available in the genome annotation under accession CP061800.

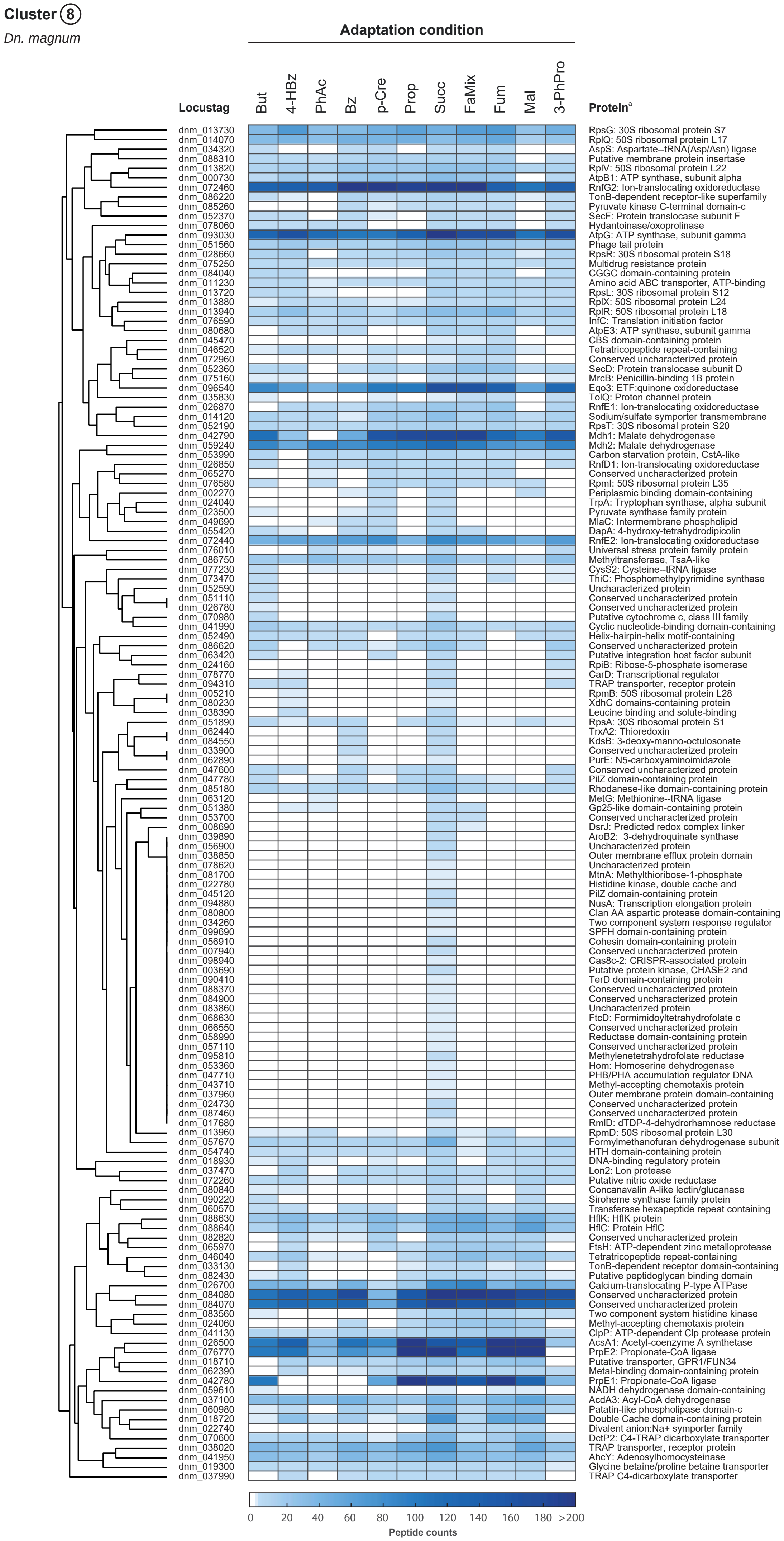

Fig. S66: Detailed view on abundances of proteins forming a sub-cluster in the global clustering depicted in Fig. S7. The global clustering is based on standardized protein abundances of all detected proteins of *Dn. magnum* across the 11 studied substrate adaptation conditions.

[a] Full description of protein functions are available in the genome annotation under accession CP061800.

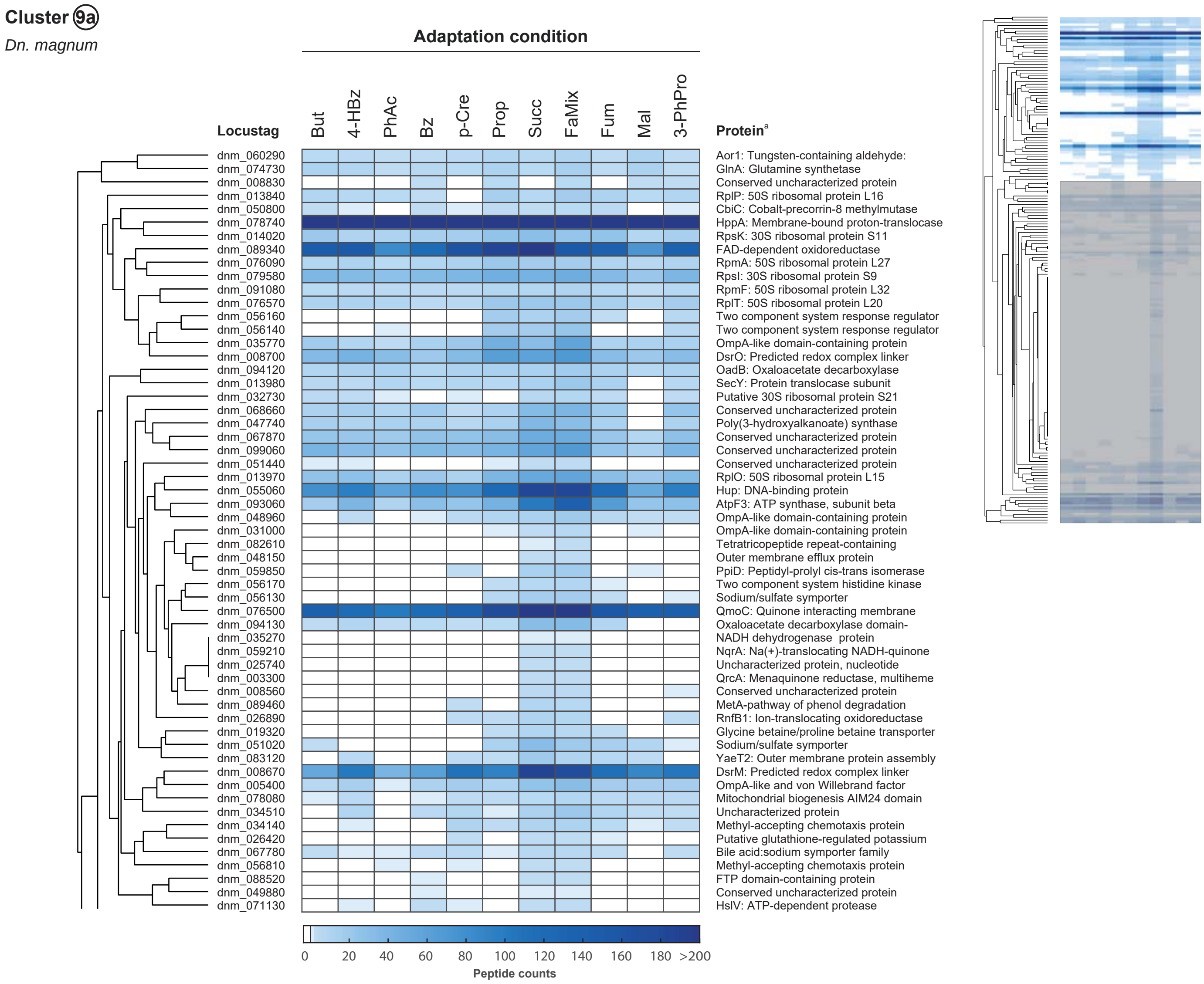

Fig. S67: Detailed view on abundances of proteins forming a sub-cluster in the global clustering depicted in Fig. S7. The global clustering is based on standardized protein abundances of all detected proteins of *Dn. magnum* across the 11 studied substrate adaptation conditions.

[a] Full description of protein functions are available in the genome annotation under accession CP061800.

Cluster 9b
Dn. magnum

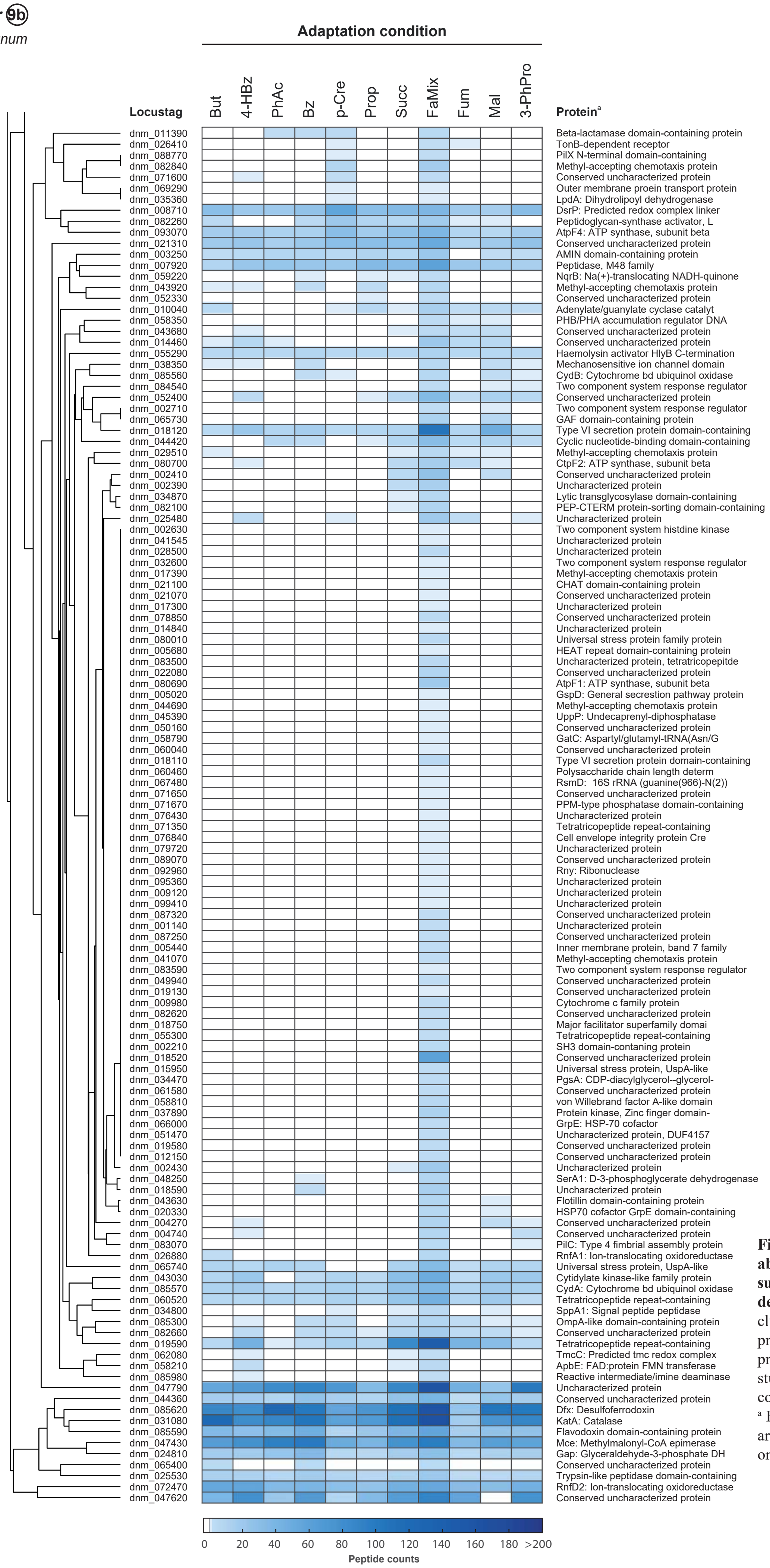

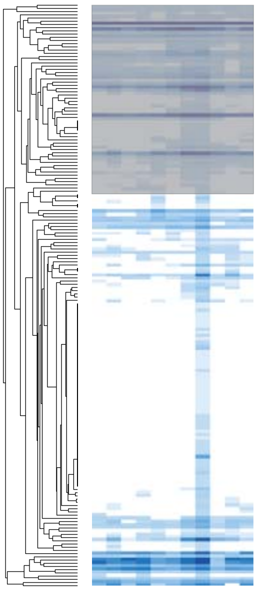

Fig. S68: Detailed view on abundances of proteins forming a sub-cluster in the global clustering depicted in Fig. S7. The global clustering is based on standardized protein abundances of all detected proteins of *Dn. magnum* across the 11 studied substrate adaptation conditions.

[a] Full description of protein functions are available in the genome annotation under accession CP061800.

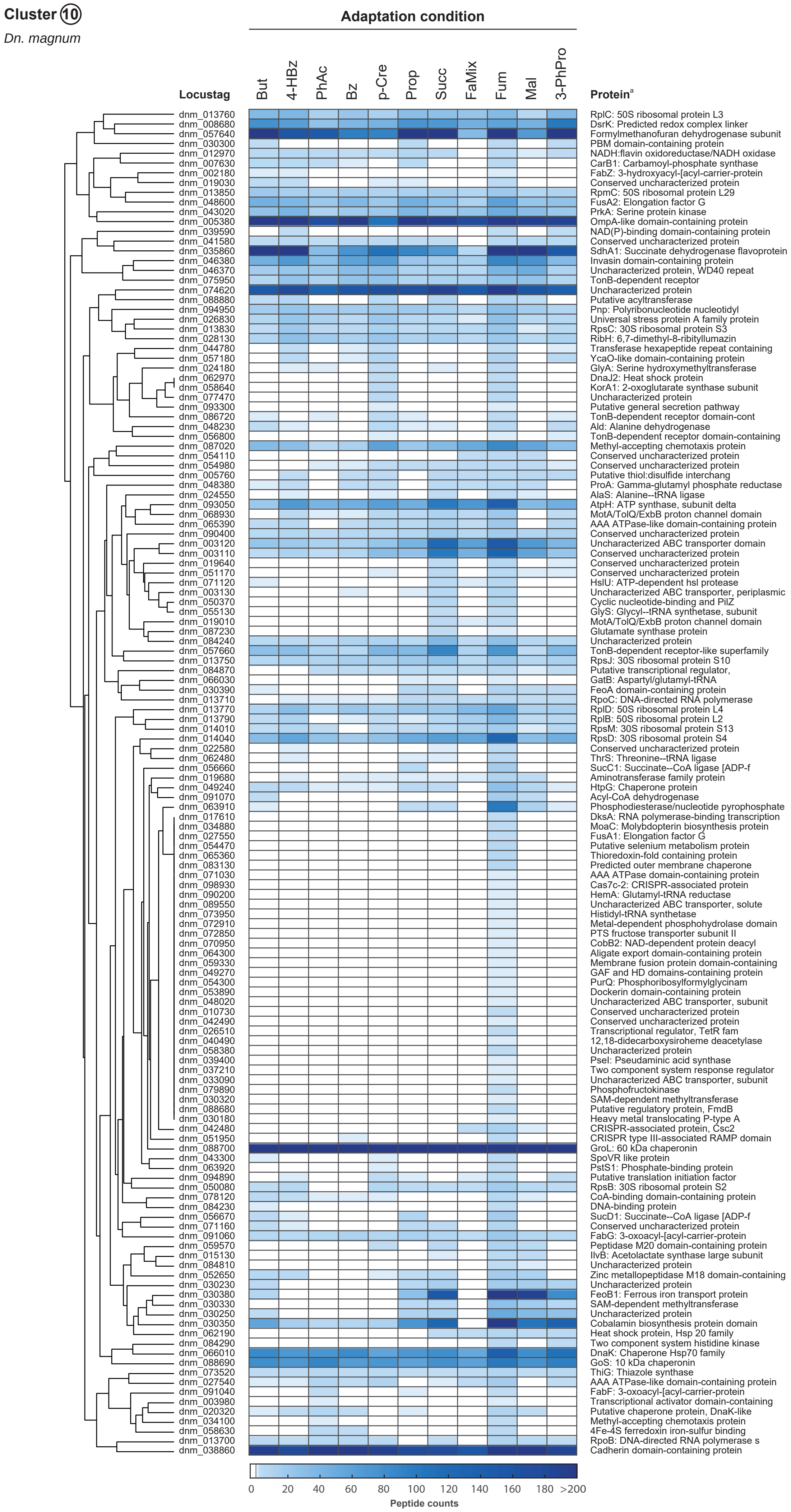

Fig. S69: Detailed view on abundances of proteins forming a sub-cluster in the global clustering depicted in Fig. S7. The global clustering is based on standardized protein abundances of all detected proteins of *Dn. magnum* across the 11 studied substrate adaptation conditions.

[a] Full description of protein functions are available in the genome annotation under accession CP061800.

Cluster (11)

Dn. magnum

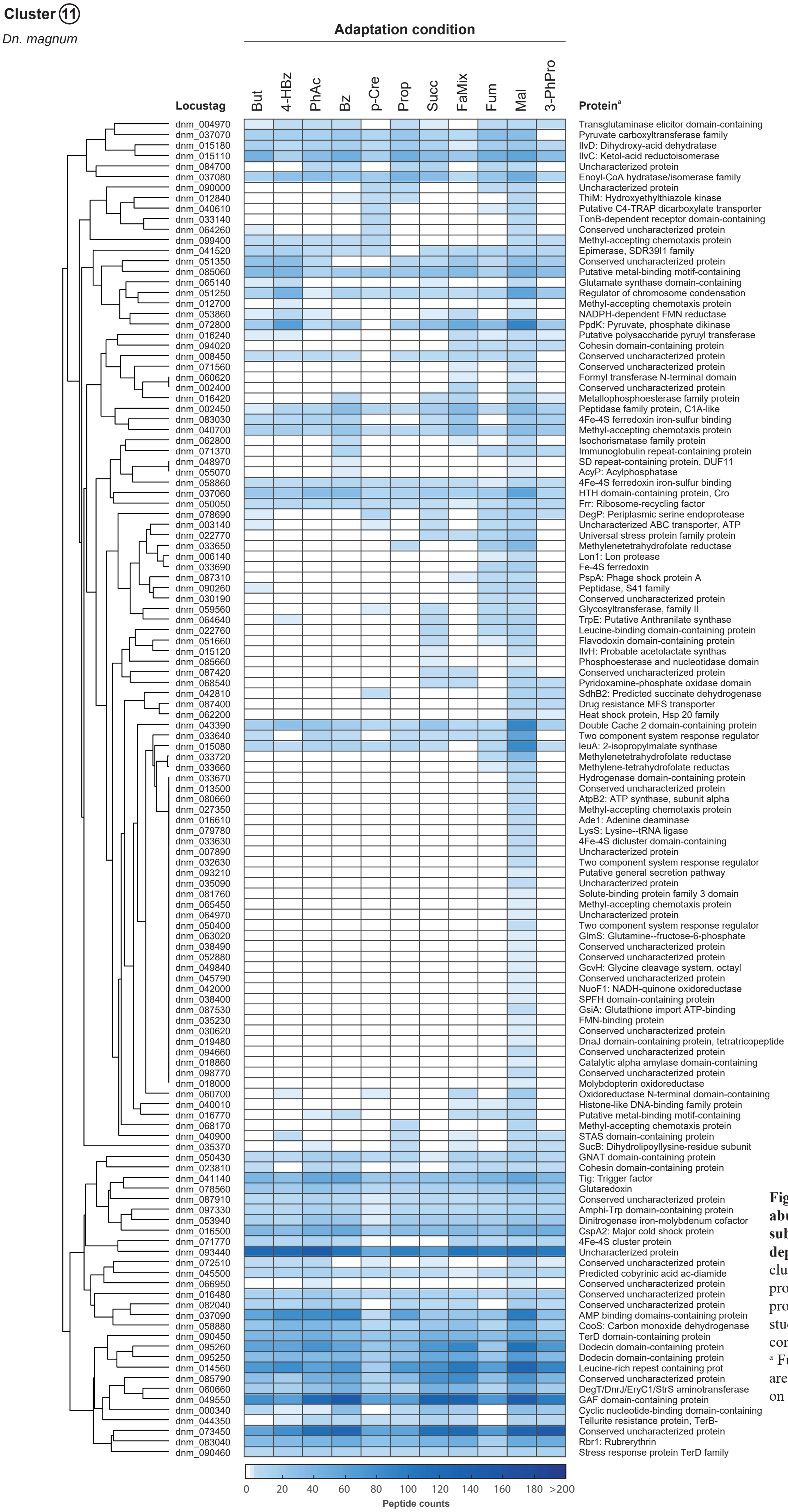

Fig. S70: Detailed view on abundances of proteins forming a sub-cluster in the global clustering depicted in Fig. S7. The global clustering is based on standardized protein abundances of all detected proteins of *Dn. magnum* across the 11 studied substrate adaptation conditions.

[a] Full description of protein functions are available in the genome annotation under accession CP061800.

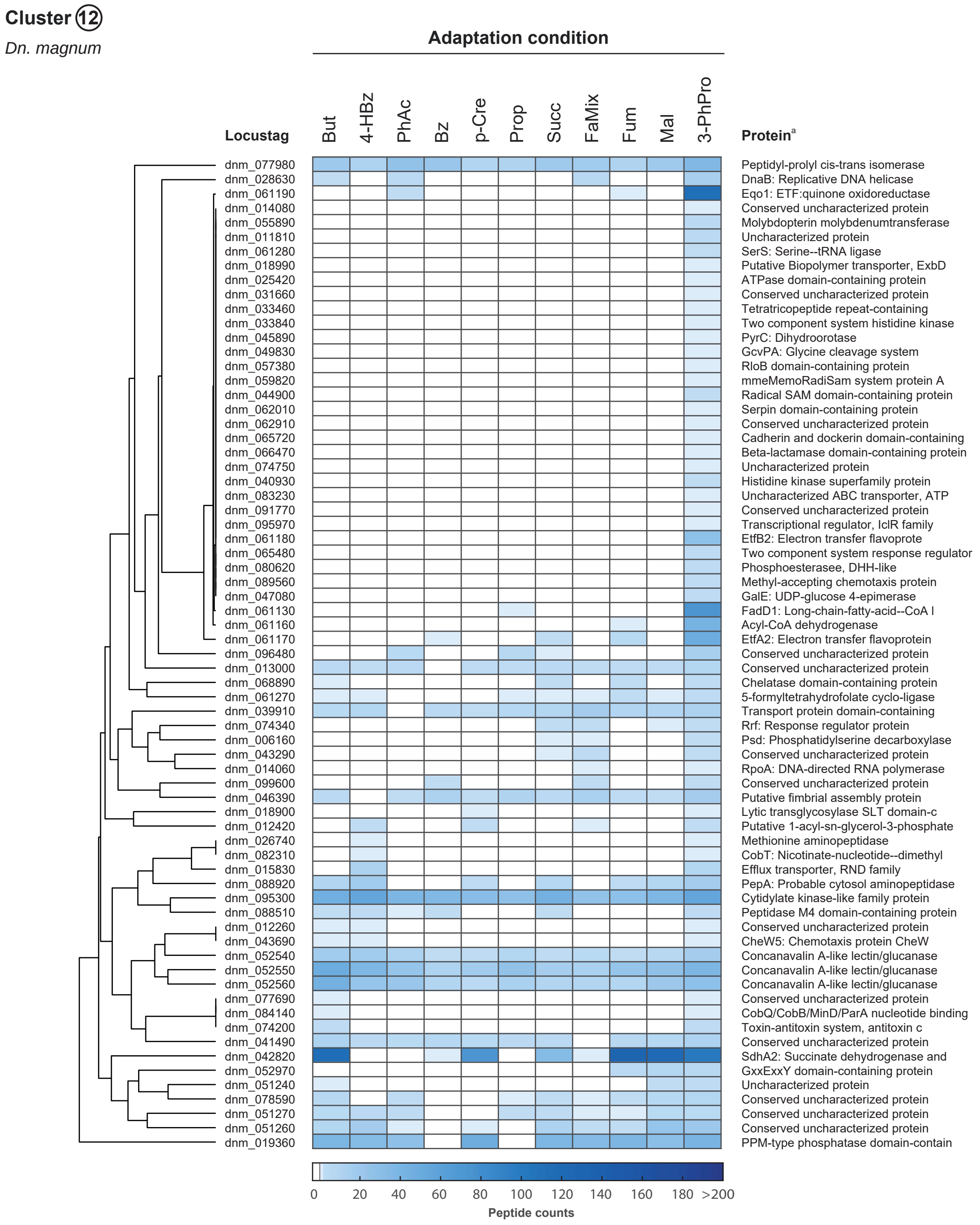

Fig. S71: Detailed view on abundances of proteins forming a sub-cluster in the global clustering depicted in Fig. S7. The global clustering is based on standardized protein abundances of all detected proteins of *Dn. magnum* across the 11 studied substrate adaptation conditions.

[a] Full description of protein functions are available in the genome annotation under accession CP061800.

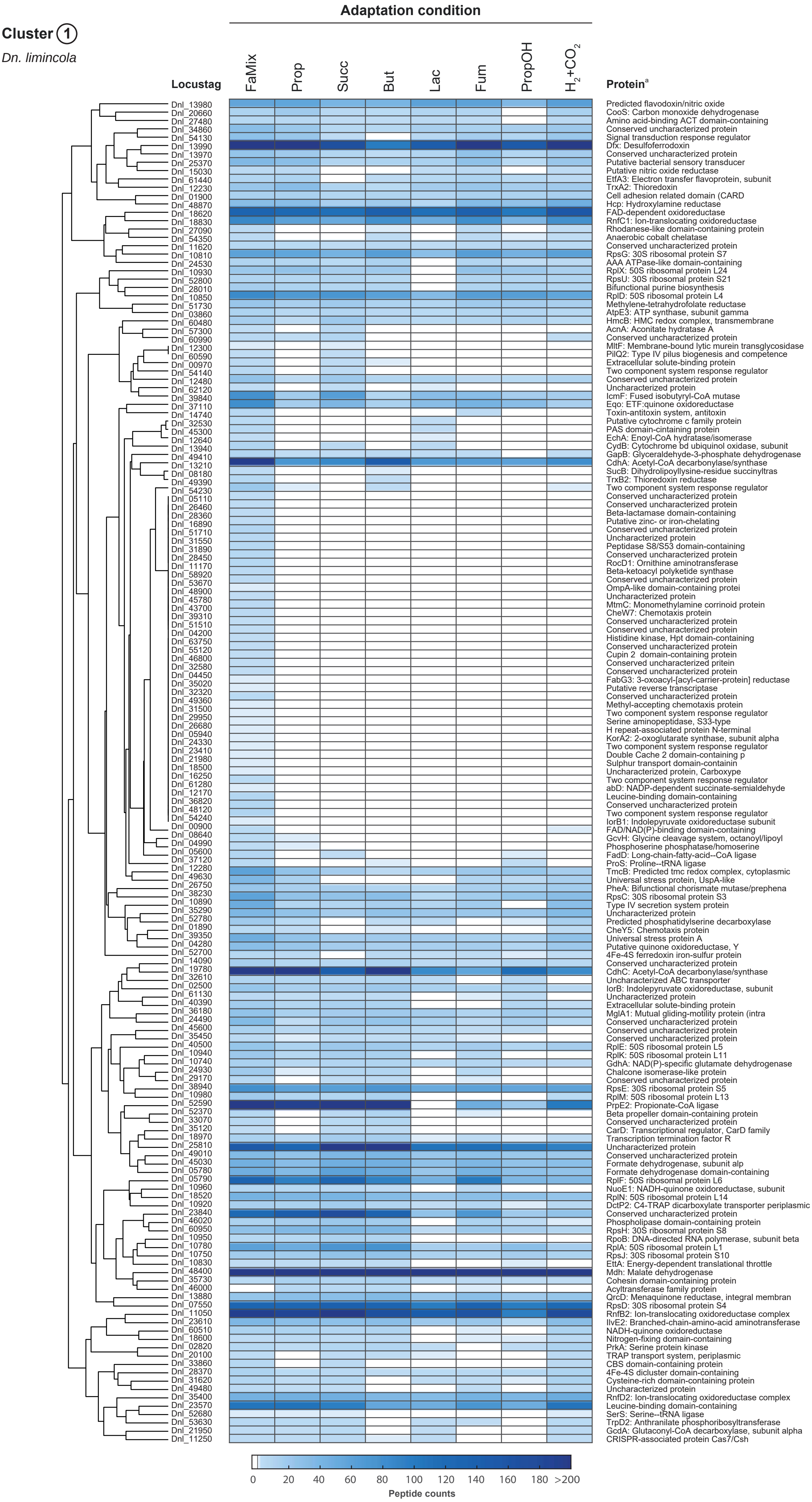

Fig. S72: Detailed view on abundances of proteins forming a sub-cluster in the global clustering depicted in Fig. S8. The global clustering is based on standardized protein abundances of all detected proteins of *Dn. limicola* across the 8 studied substrate adaptation conditions.

[a] Full description of protein functions are available in the genome annotation under accession CP061799.

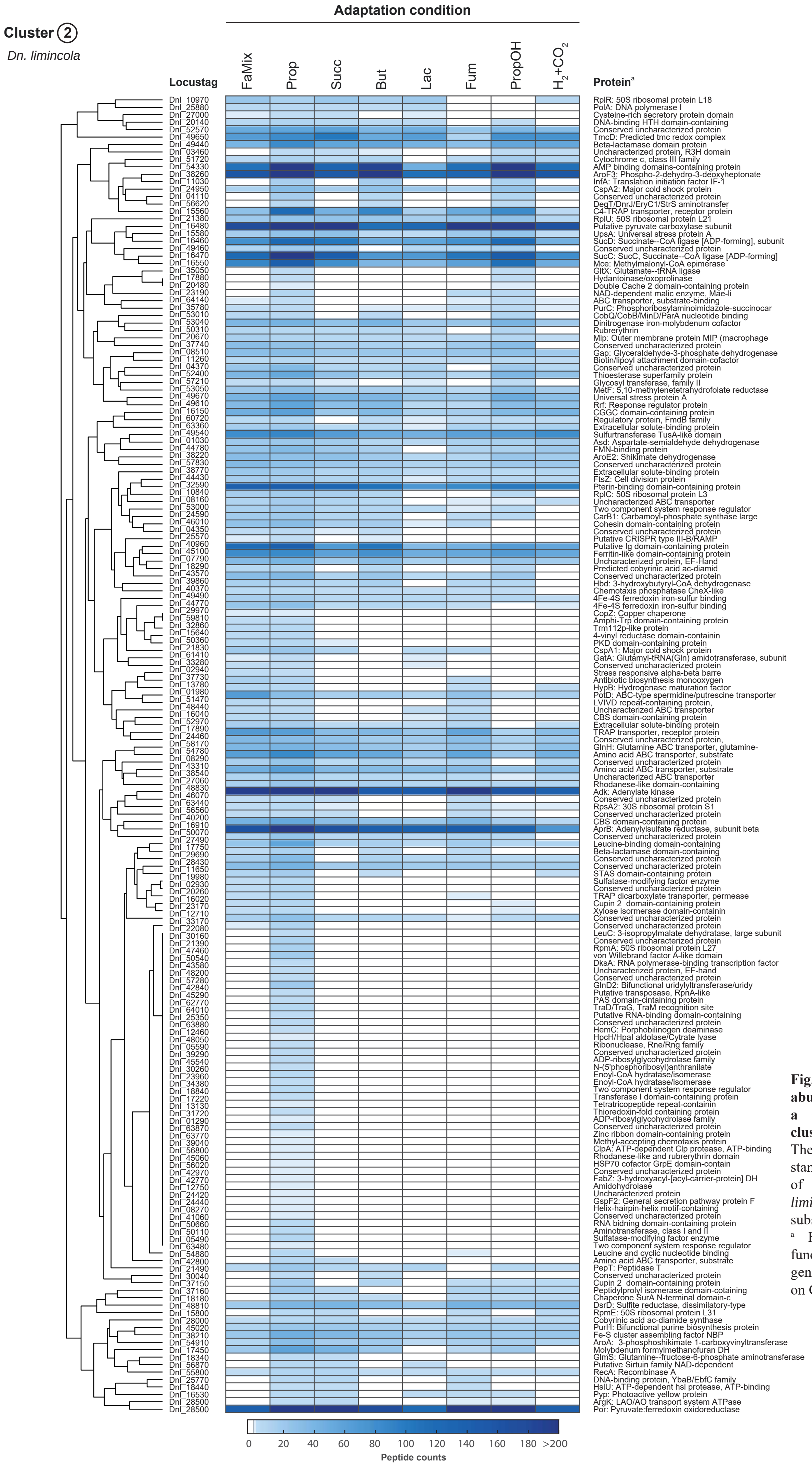

Fig. S73: Detailed view on abundances of proteins forming a sub-cluster in the global clustering depicted in Fig. S8. The global clustering is based on standardized protein abundances of all detected proteins of *Dn. limicola* across the 8 studied substrate adaptation conditions.

[a] Full description of protein functions are available in the genome annotation under accession CP061799.

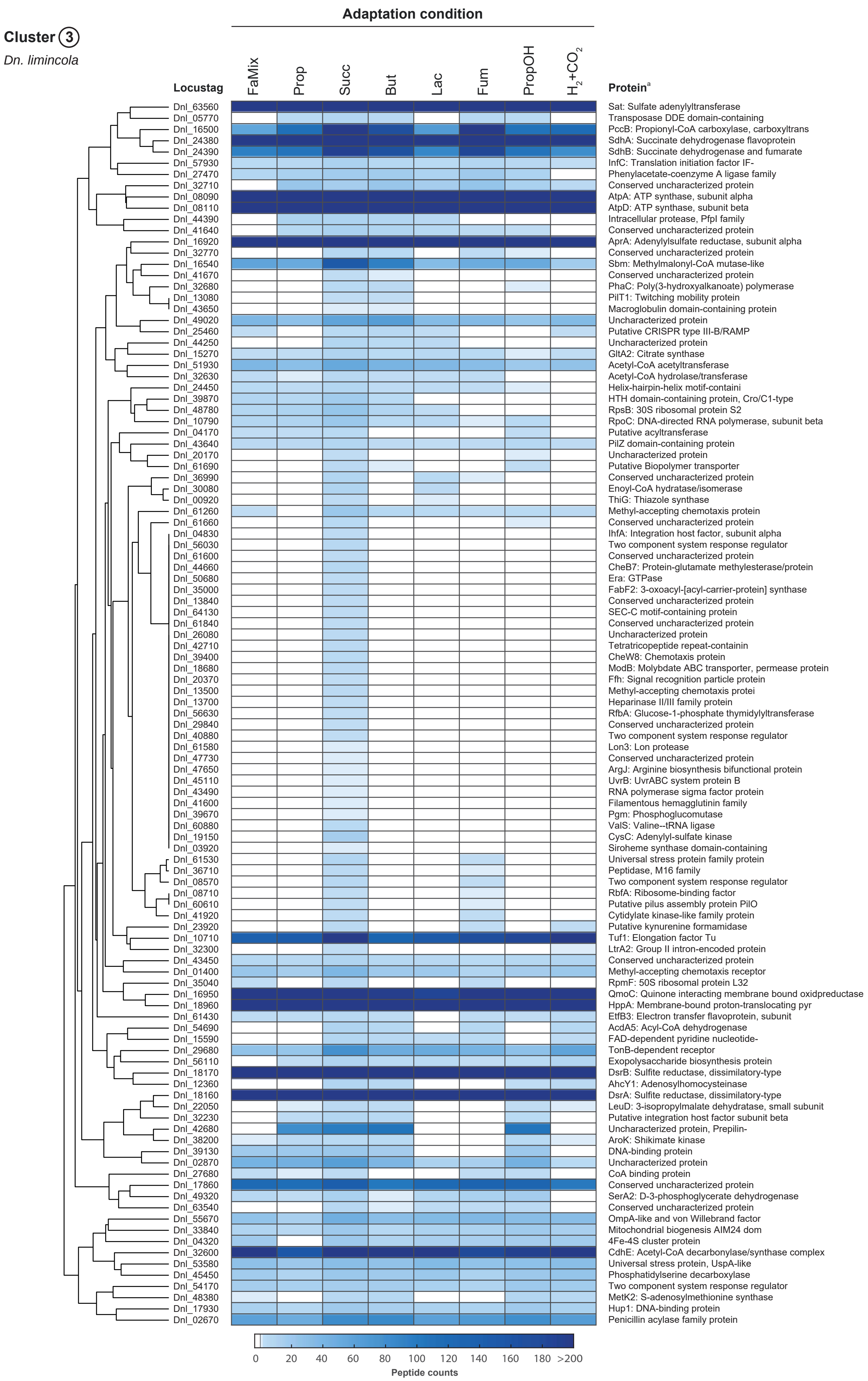

Fig. S74: Detailed view on abundances of proteins forming a sub-cluster in the global clustering depicted in Fig. S8. The global clustering is based on standardized protein abundances of all detected proteins of *Dn. limicola* across the 8 studied substrate adaptation conditions.

[a] Full description of protein functions are available in the genome annotation under accession CP061799.

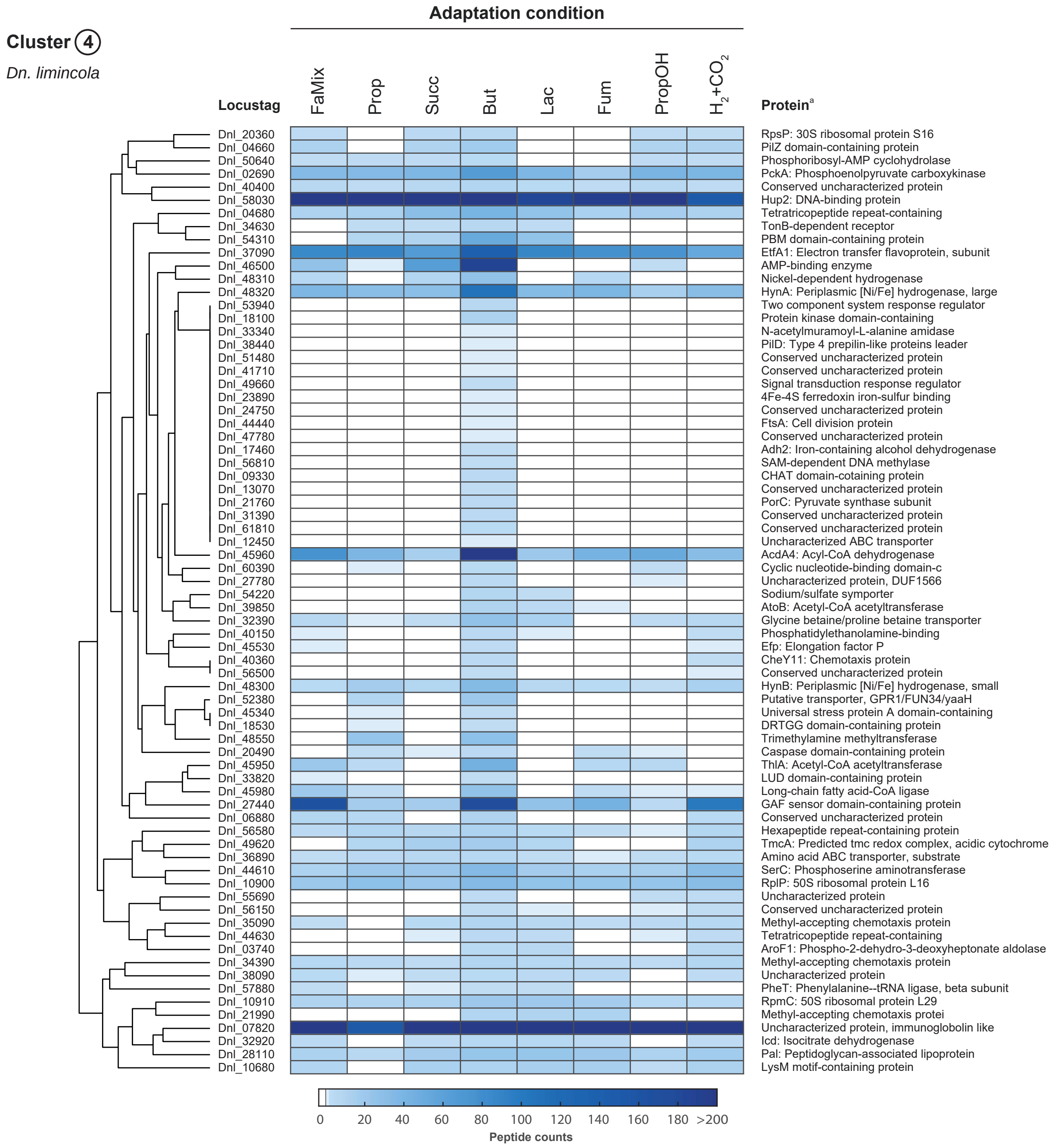

Fig. S75: Detailed view on abundances of proteins forming a sub-cluster in the global clustering depicted in Fig. S8. The global clustering is based on standardized protein abundances of all detected proteins of *Dn. limicola* across the 8 studied substrate adaptation conditions.

[a] Full description of protein functions are available in the genome annotation under accession CP061799.

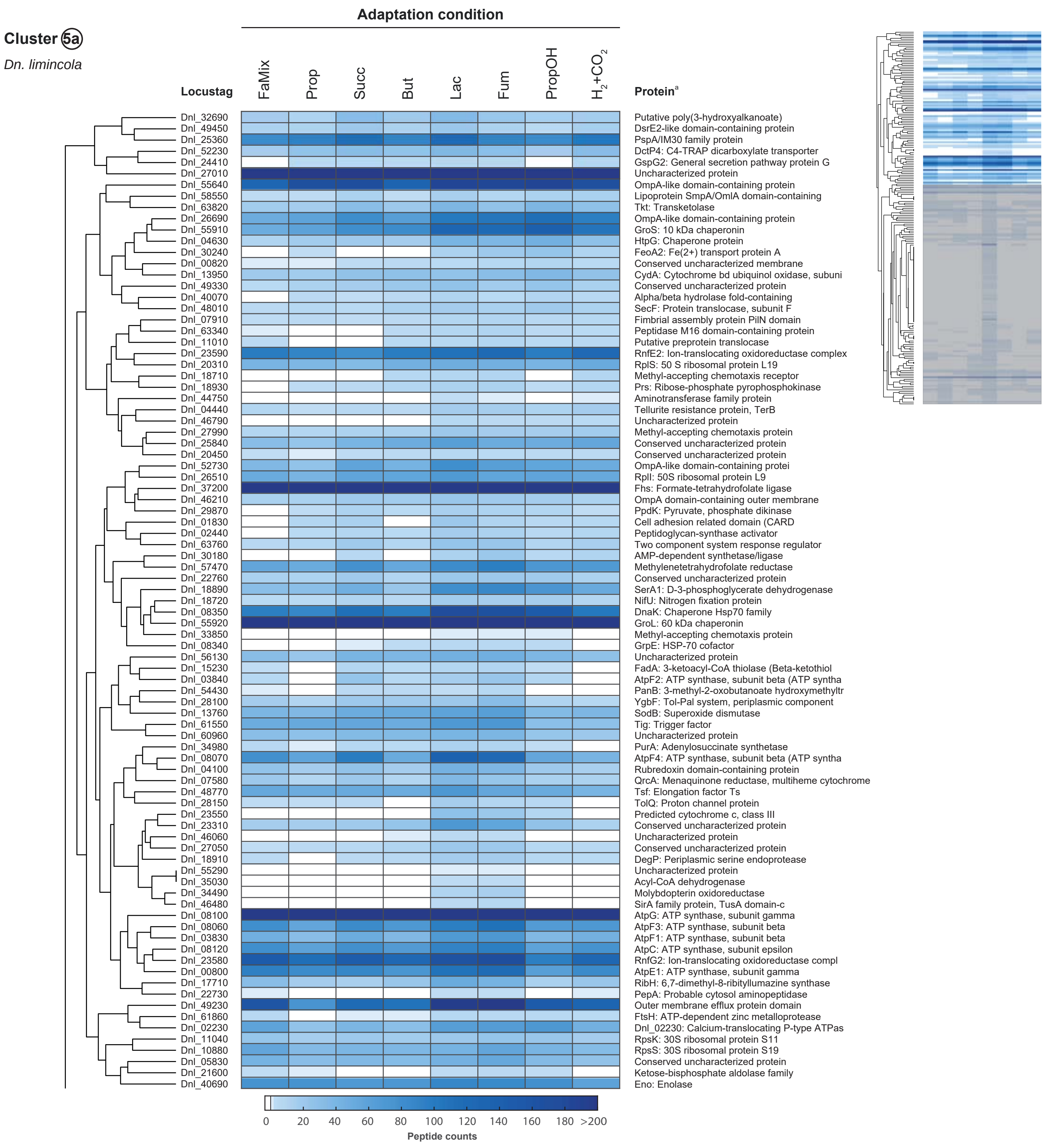

Fig. S76: Detailed view on abundances of proteins forming a sub-cluster in the global clustering depicted in Fig. S8. The global clustering is based on standardized protein abundances of all detected proteins of *Dn. limicola* across the 8 studied substrate adaptation conditions.

[a] Full description of protein functions are available in the genome annotation under accession CP061799.

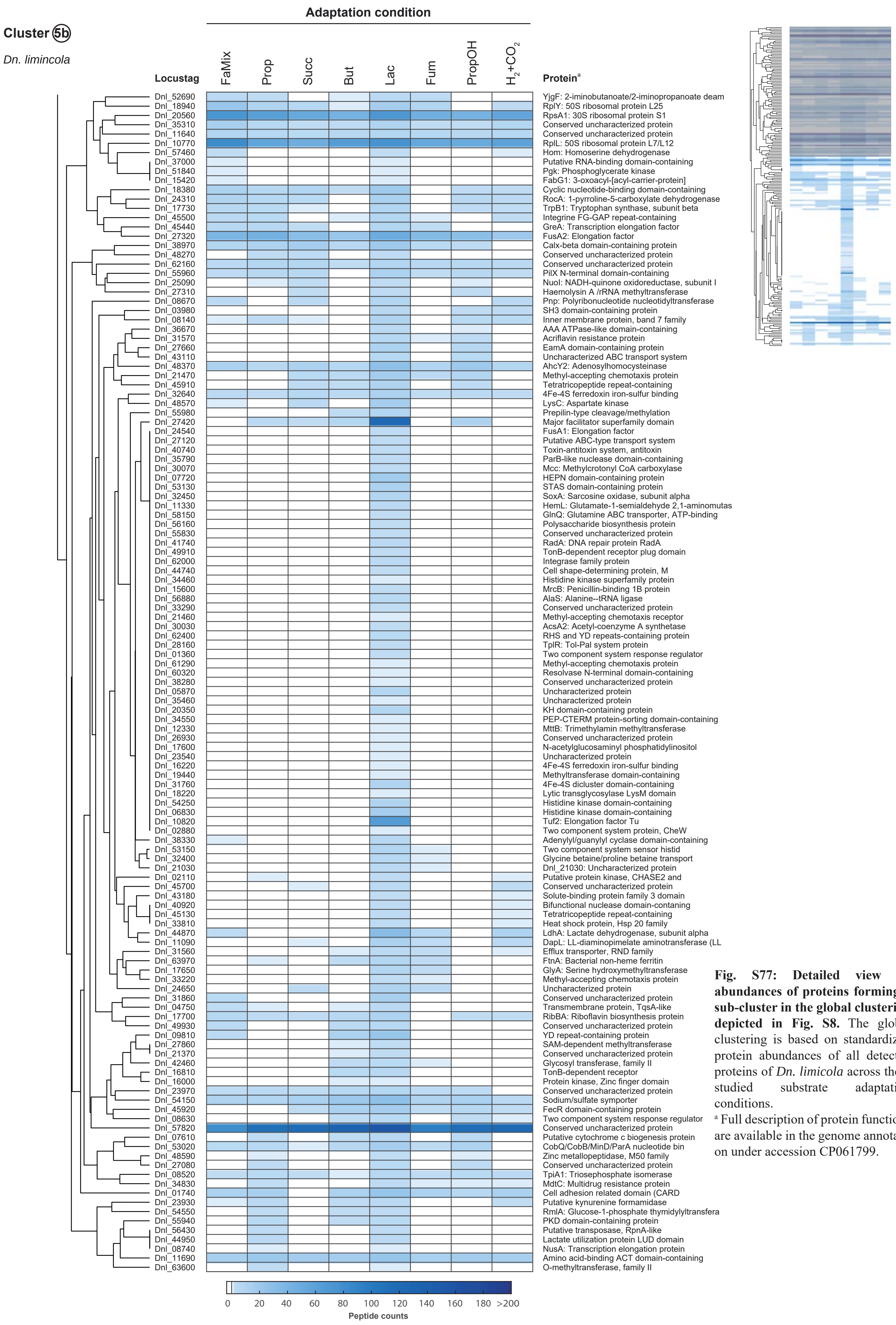

Fig. S77: Detailed view on abundances of proteins forming a sub-cluster in the global clustering depicted in Fig. S8. The global clustering is based on standardized protein abundances of all detected proteins of *Dn. limicola* across the 8 studied substrate adaptation conditions.

[a] Full description of protein functions are available in the genome annotation under accession CP061799.

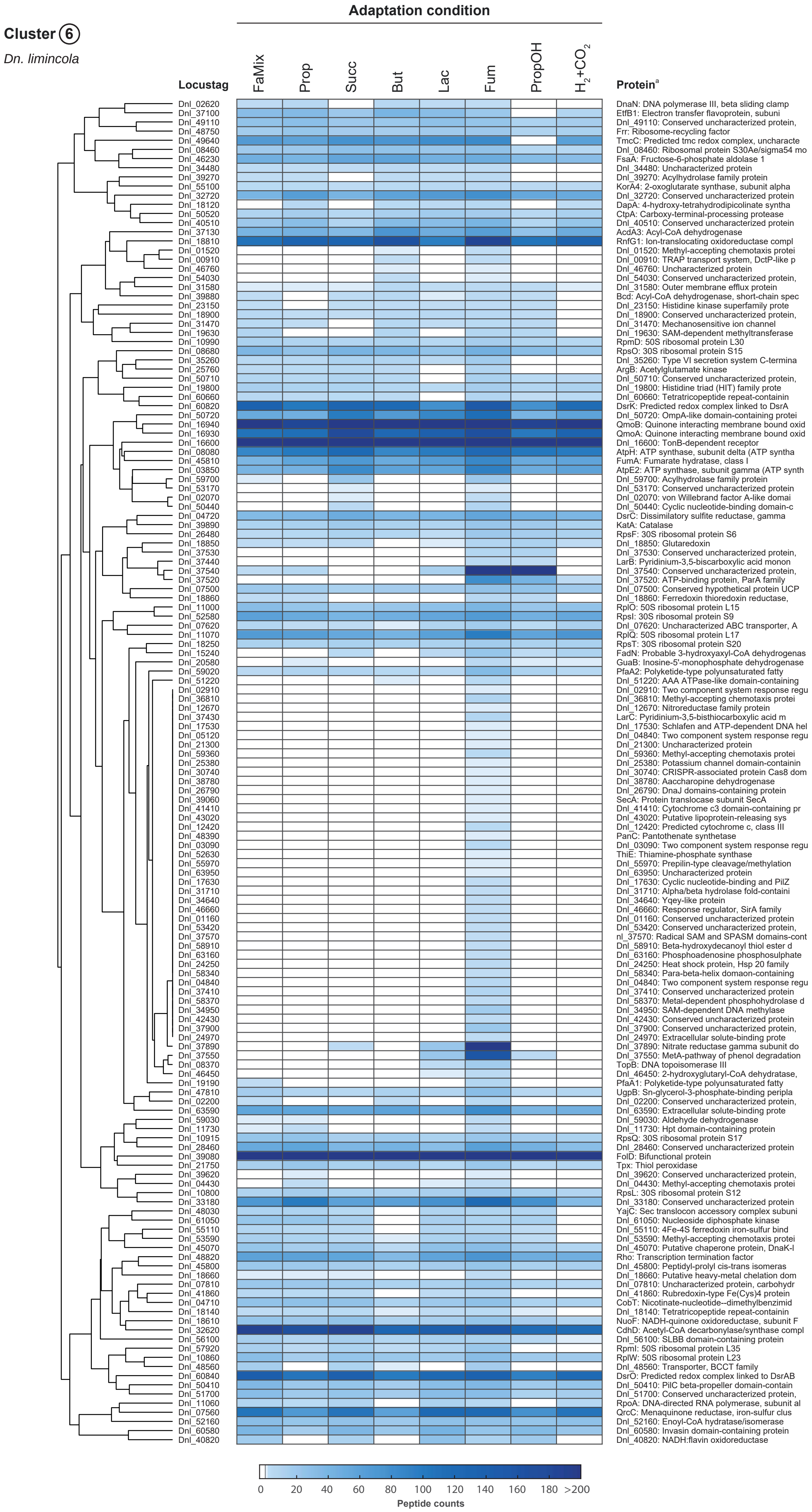

Fig. S78: Detailed view on abundances of proteins forming a sub-cluster in the global clustering depicted in Fig. S8. The global clustering is based on standardized protein abundances of all detected proteins of *Dn. limicola* across the 8 studied substrate adaptation conditions.

[a] Full description of protein functions are available in the genome annotation under accession CP061799.

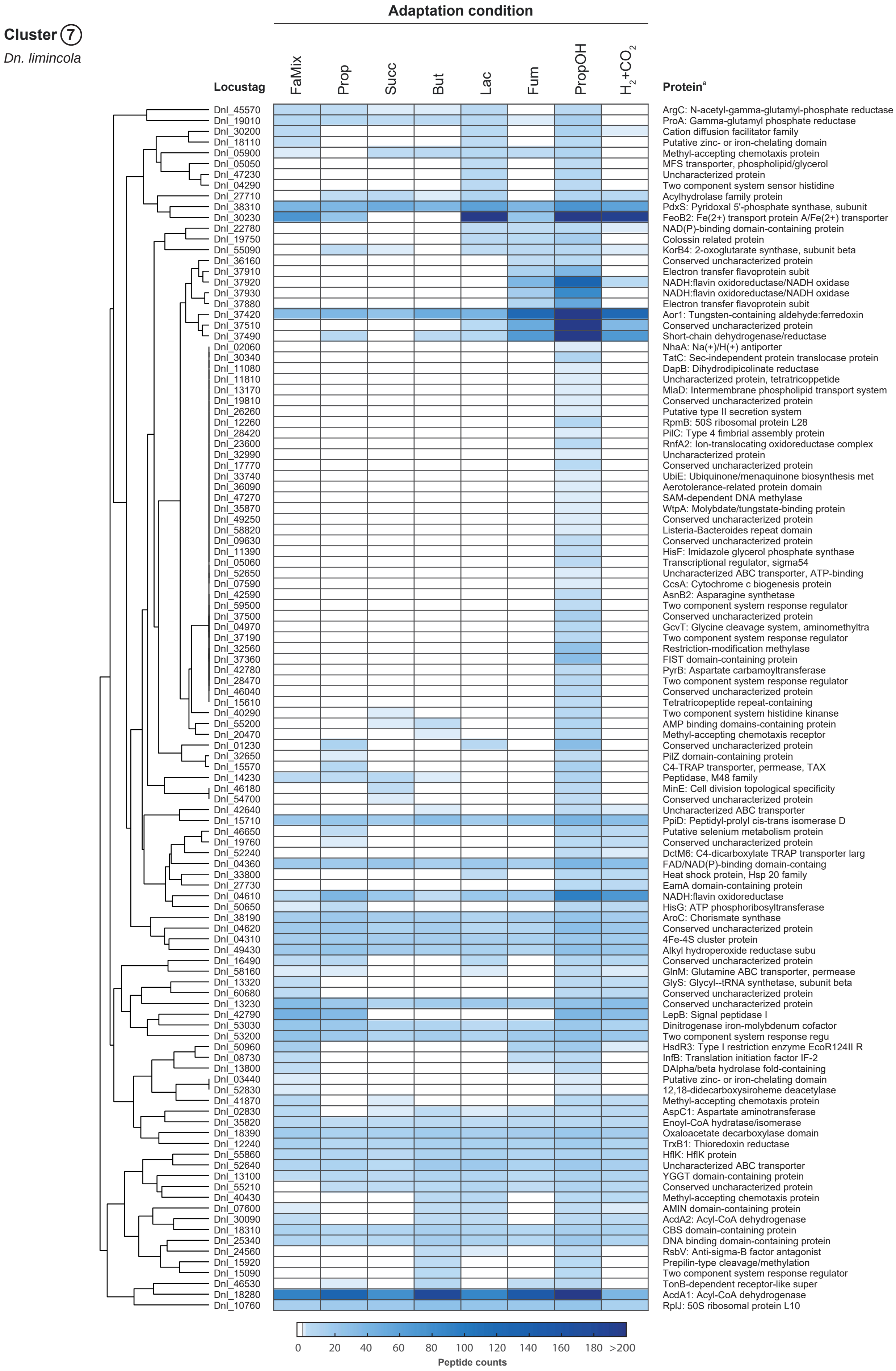

Fig. S79: Detailed view on abundances of proteins forming a sub-cluster in the global clustering depicted in Fig. S8. The global clustering is based on standardized protein abundances of all detected proteins of *Dn. limicola* across the 8 studied substrate adaptation conditions.

[a] Full description of protein functions are available in the genome annotation under accession CP061799.

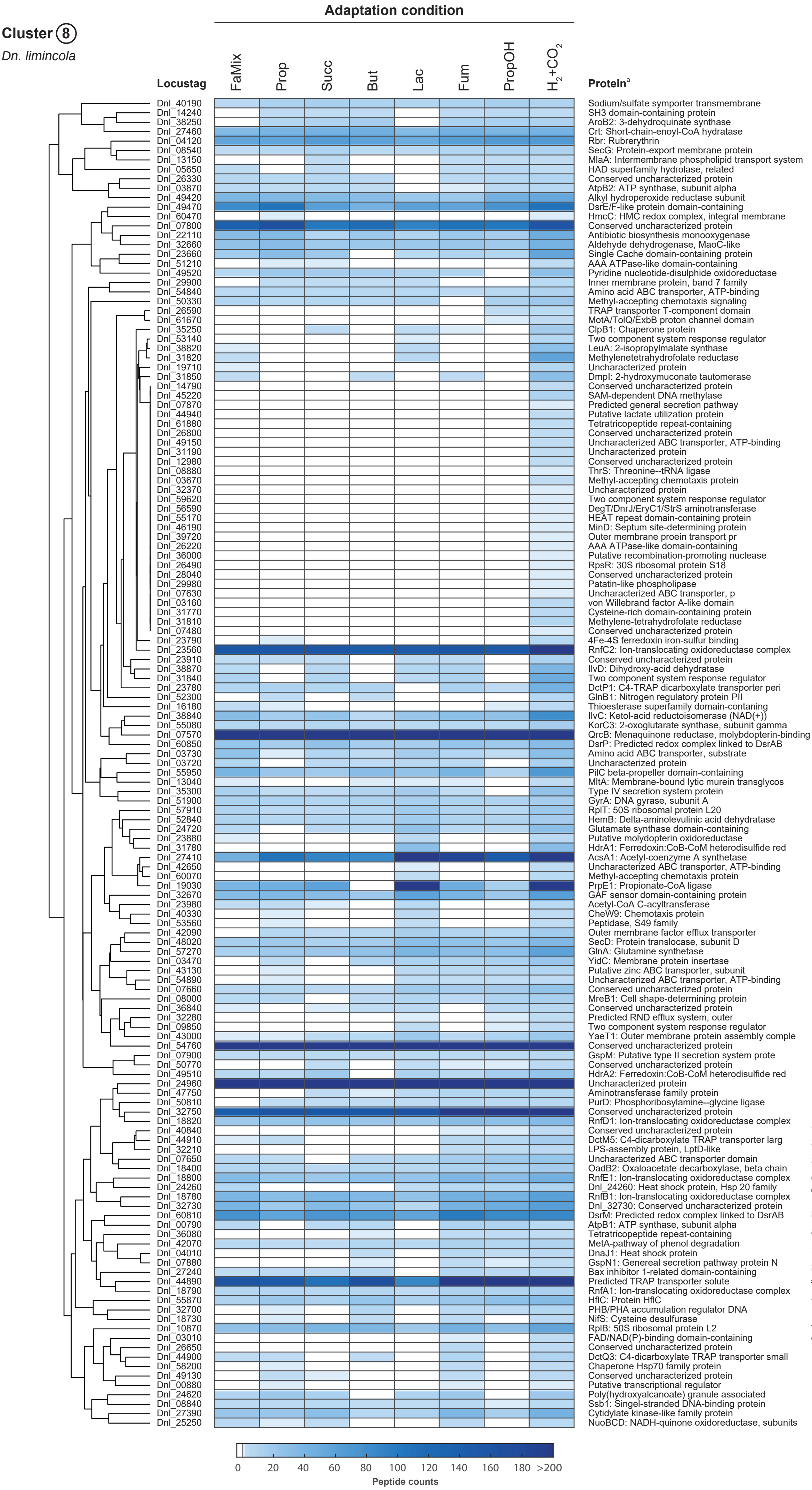

Fig. S80: Detailed view on abundances of proteins forming a sub-cluster in the global clustering depicted in Fig. S8. The global clustering is based on standardized protein abundances of all detected proteins of *Dn. limicola* across the 8 studied substrate adaptation conditions.

[a] Full description of protein functions are available in the genome annotation under accession CP061799.

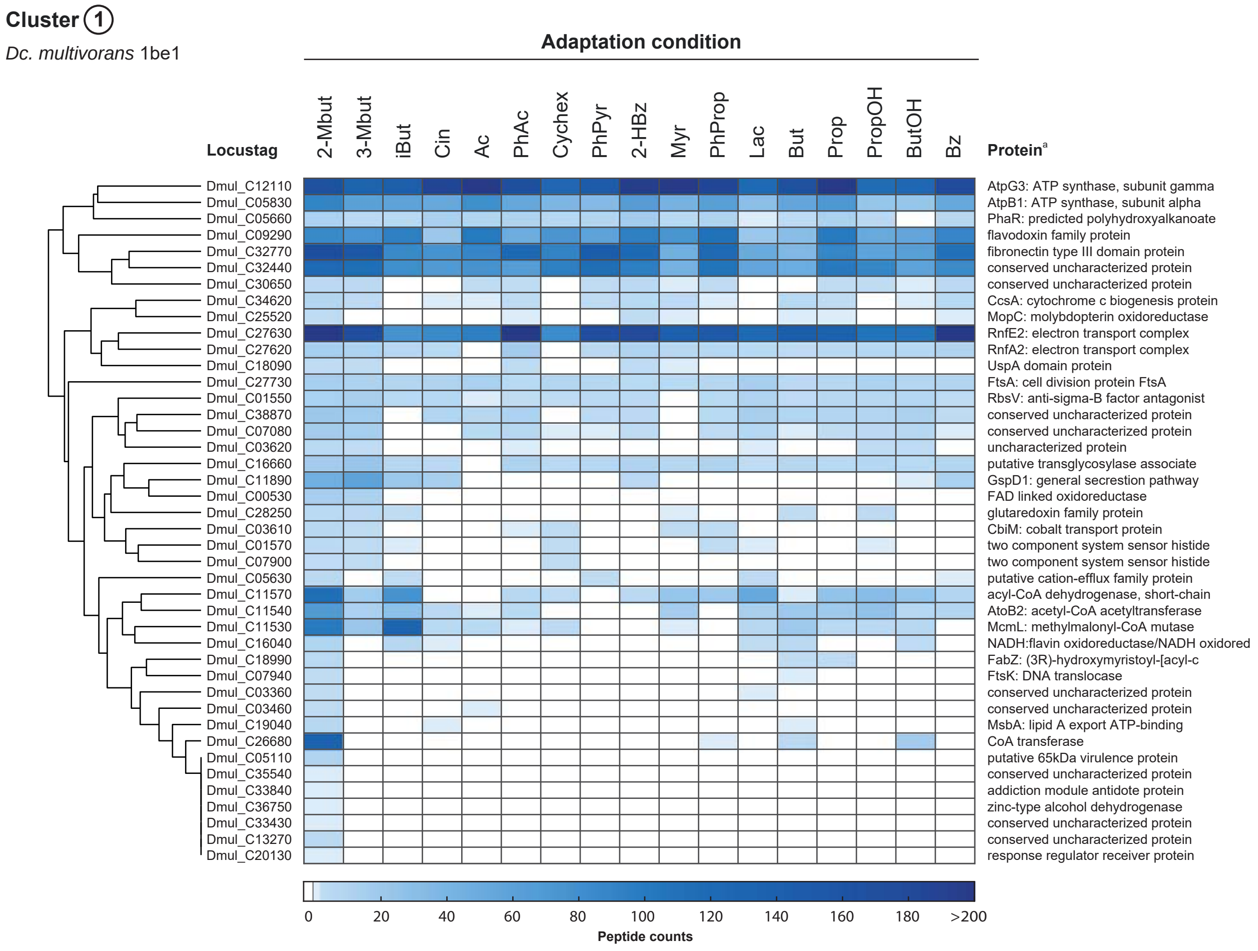

Fig. S81: Detailed view on abundances of proteins forming a sub-cluster in the global clustering depicted in Fig. S9. The global clustering is based on standardized protein abundances of all detected proteins of *Dc. multivorans* 1be1 across the 17 studied substrate adaptation conditions.

[a] Full description of protein functions are available in the genome annotation under accession CP015381.

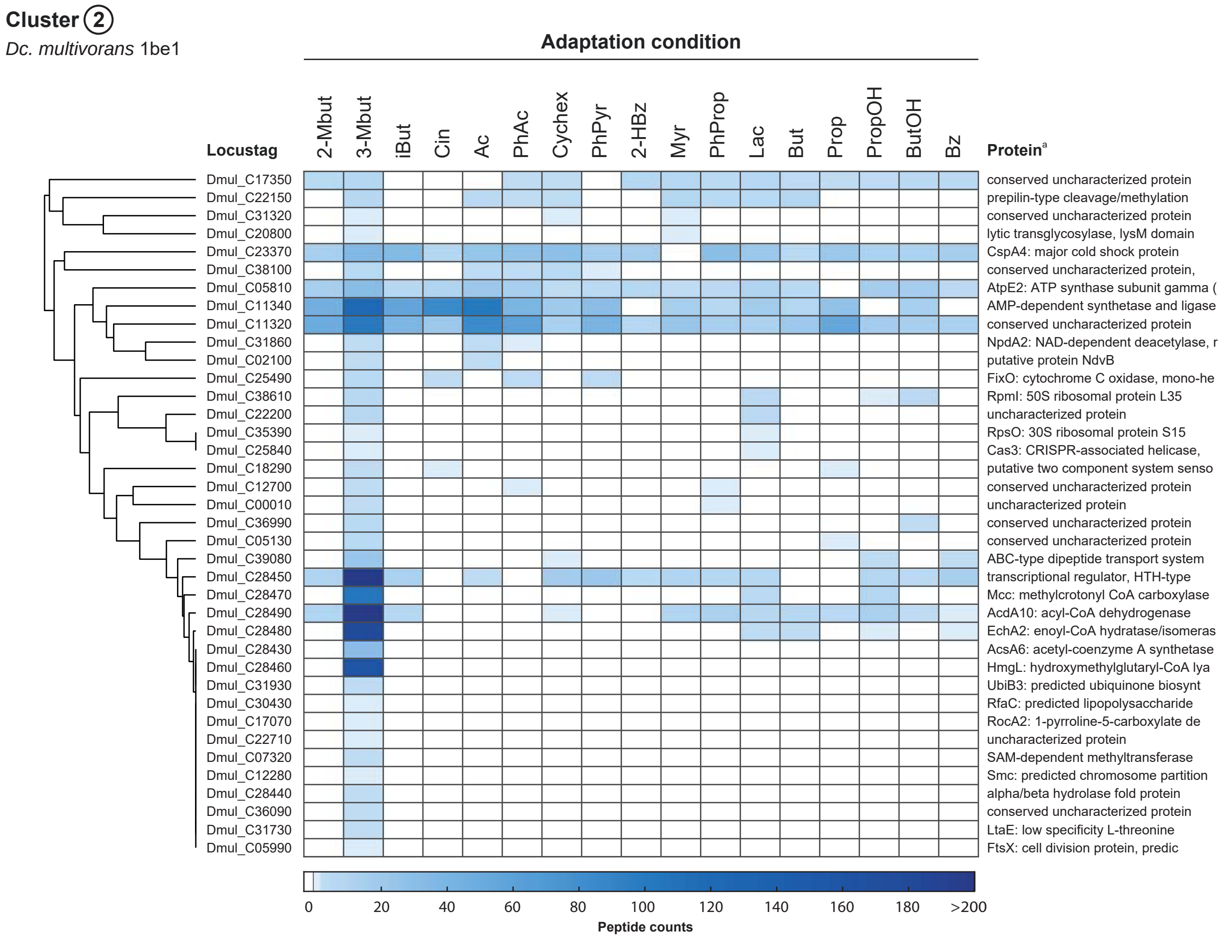

Fig. S82: Detailed view on abundances of proteins forming a sub-cluster in the global clustering depicted in Fig. S9. The global clustering is based on standardized protein abundances of all detected proteins of *Dc. multivorans* 1be1 across the 17 studied substrate adaptation conditions.

[a] Full description of protein functions are available in the genome annotation under accession CP015381.

Cluster 3

Dc. multivorans 1be1

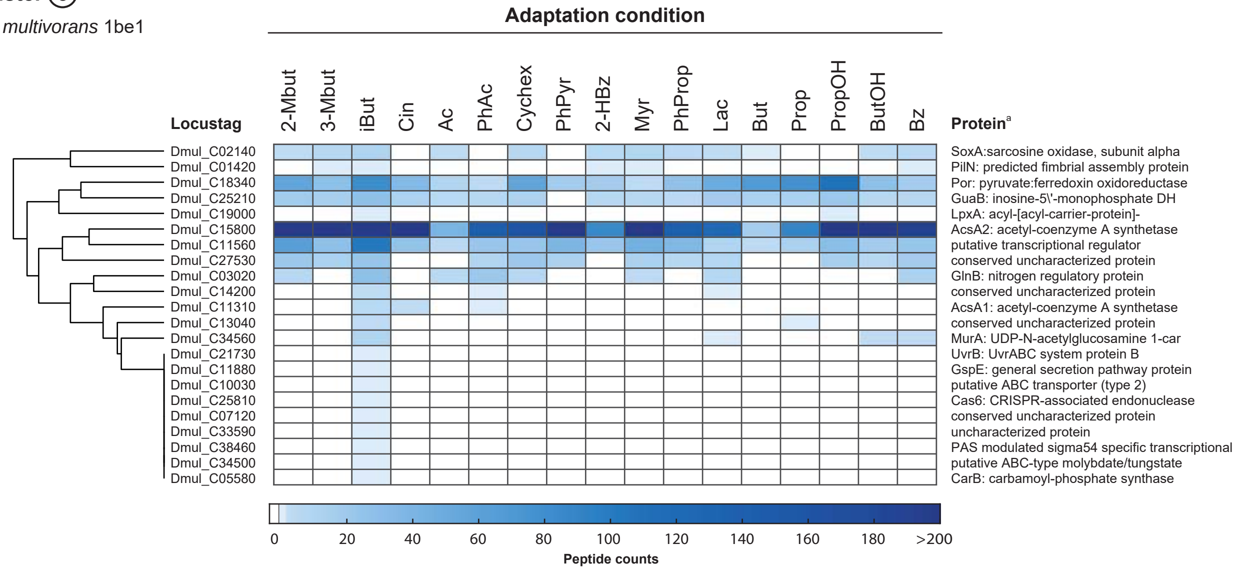

Fig. S83: Detailed view on abundances of proteins forming a sub-cluster in the global clustering depicted in Fig. S9. The global clustering is based on standardized protein abundances of all detected proteins of *Dc. multivorans* 1be1 across the 17 studied substrate adaptation conditions.

[a] Full description of protein functions are available in the genome annotation under accession CP015381.

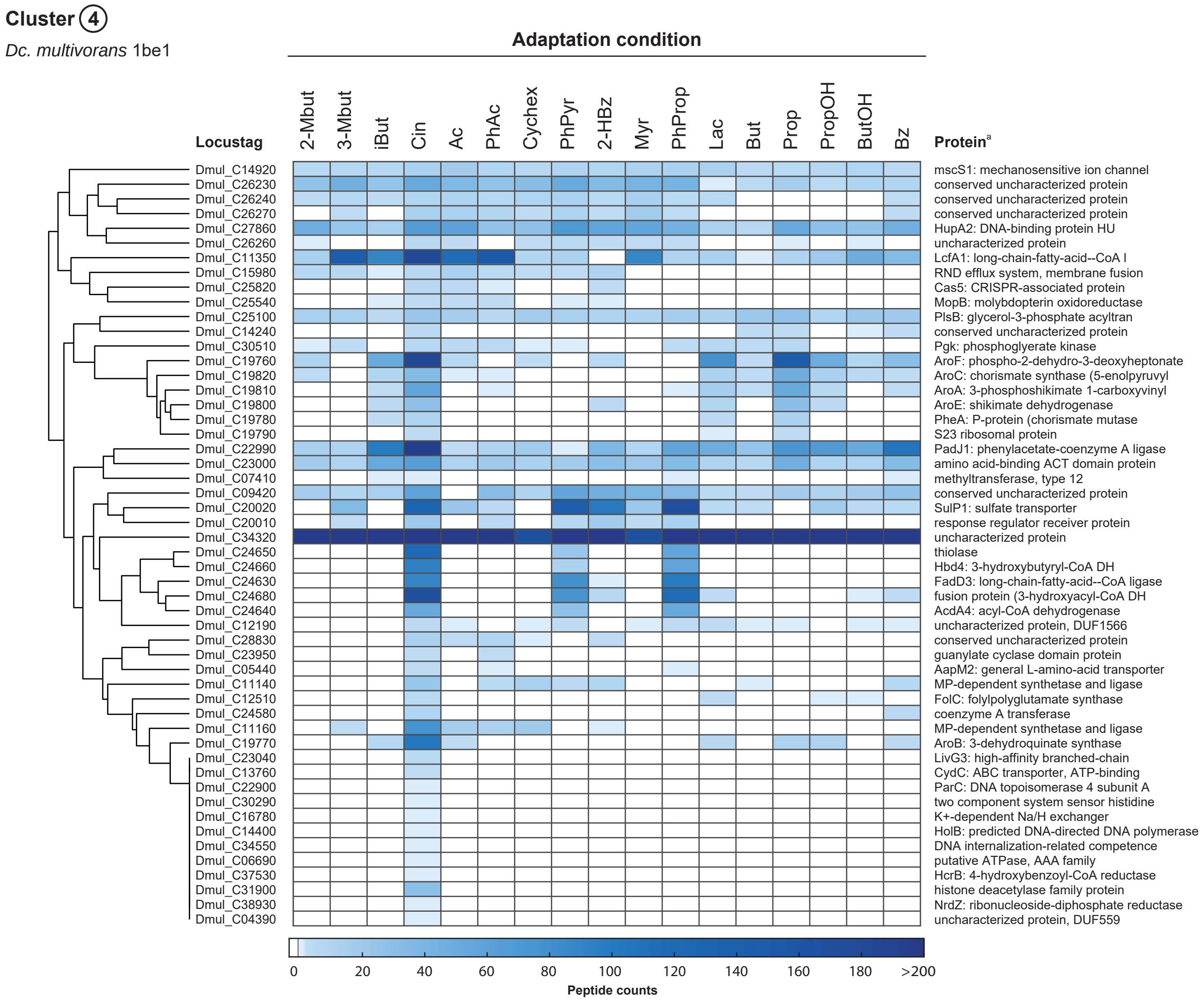

Fig. S84: Detailed view on abundances of proteins forming a sub-cluster in the global clustering depicted in Fig. S9. The global clustering is based on standardized protein abundances of all detected proteins of *Dc. multivorans* 1be1 across the 17 studied substrate adaptation conditions.
[a] Full description of protein functions are available in the genome annotation under accession CP015381.

Cluster 5a

Dc. multivorans 1be1

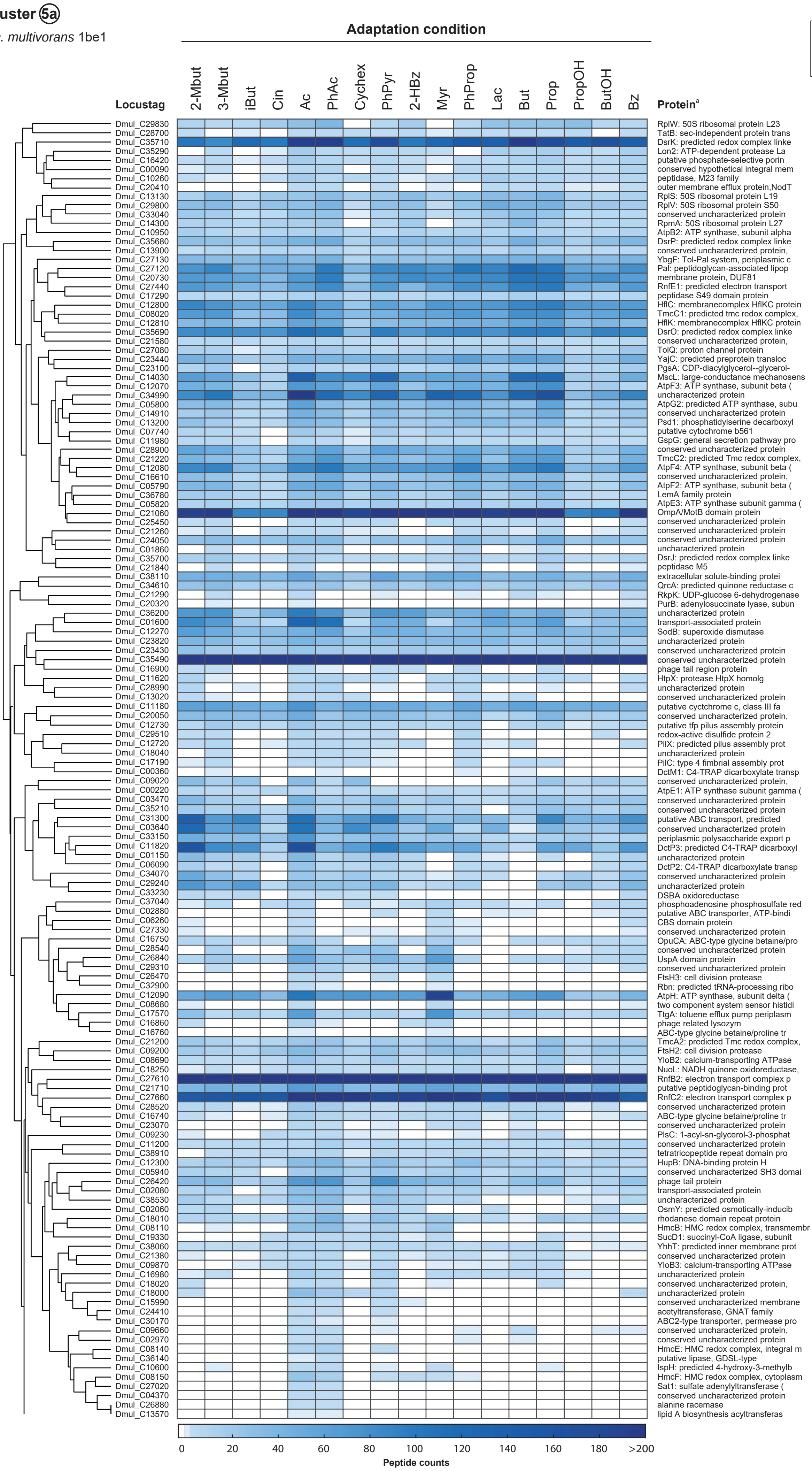

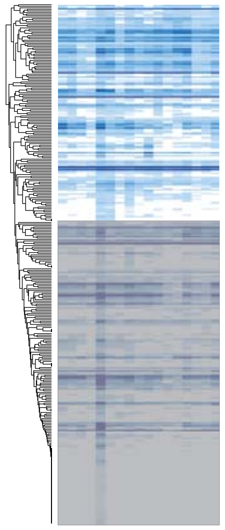

Fig. S85: Detailed view on abundances of proteins forming a sub-cluster in the global clustering depicted in Fig. S9. The global clustering is based on standardized protein abundances of all detected proteins of *Dc. multivorans* 1be1 across the 17 studied substrate adaptation conditions.

[a] Full description of protein functions are available in the genome annotation under accession CP015381.

Cluster 5b

Dc. multivorans 1be1

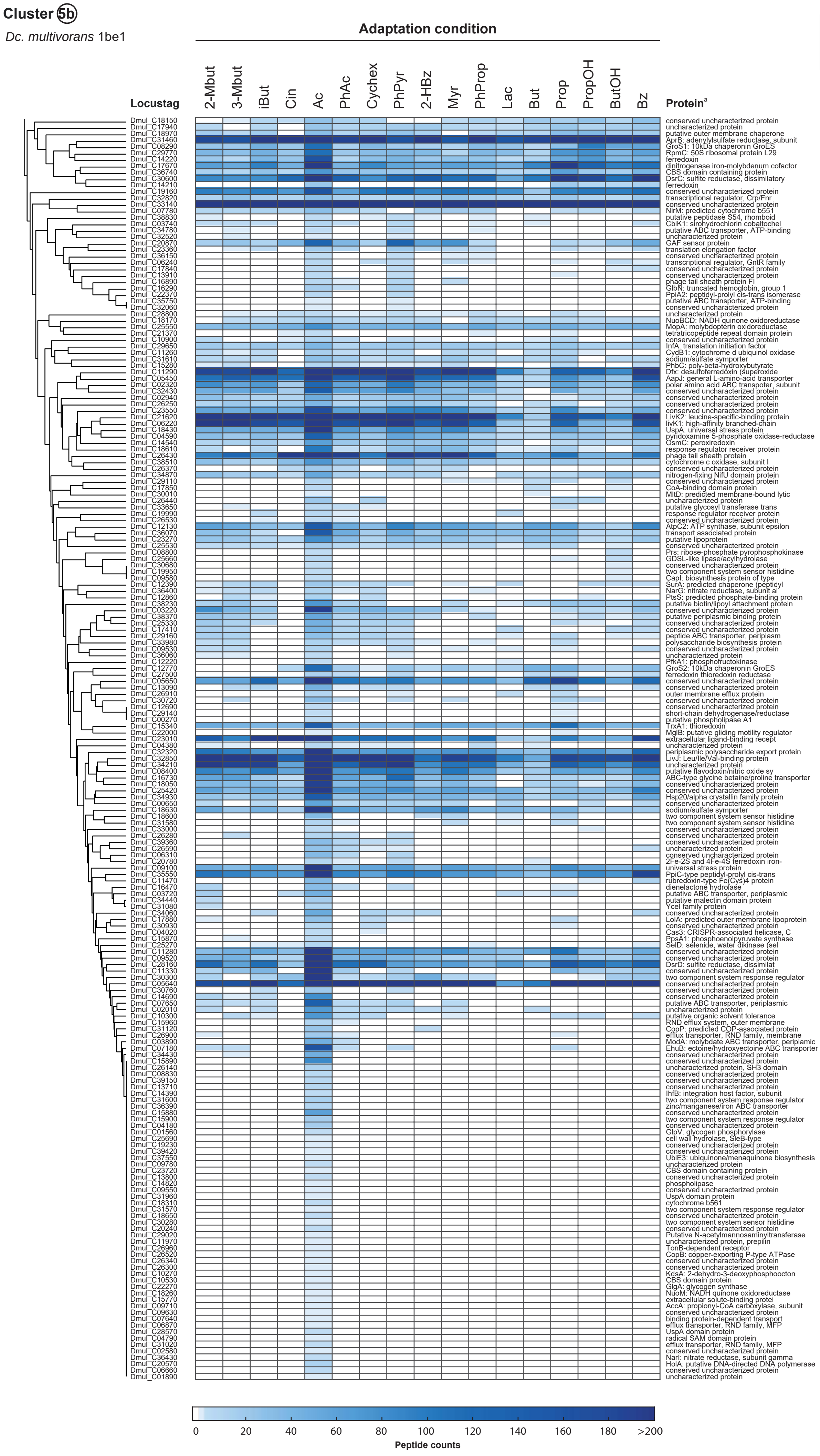

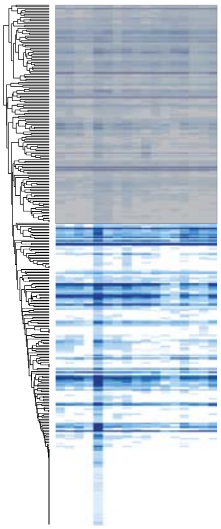

Fig. S86: Detailed view on abundances of proteins forming a sub-cluster in the global clustering depicted in Fig. S9. The global clustering is based on standardized protein abundances of all detected proteins of *Dc. multivorans* 1be1 across the 17 studied substrate adaptation conditions.

[a] Full description of protein functions are available in the genome annotation under accession CP015381.

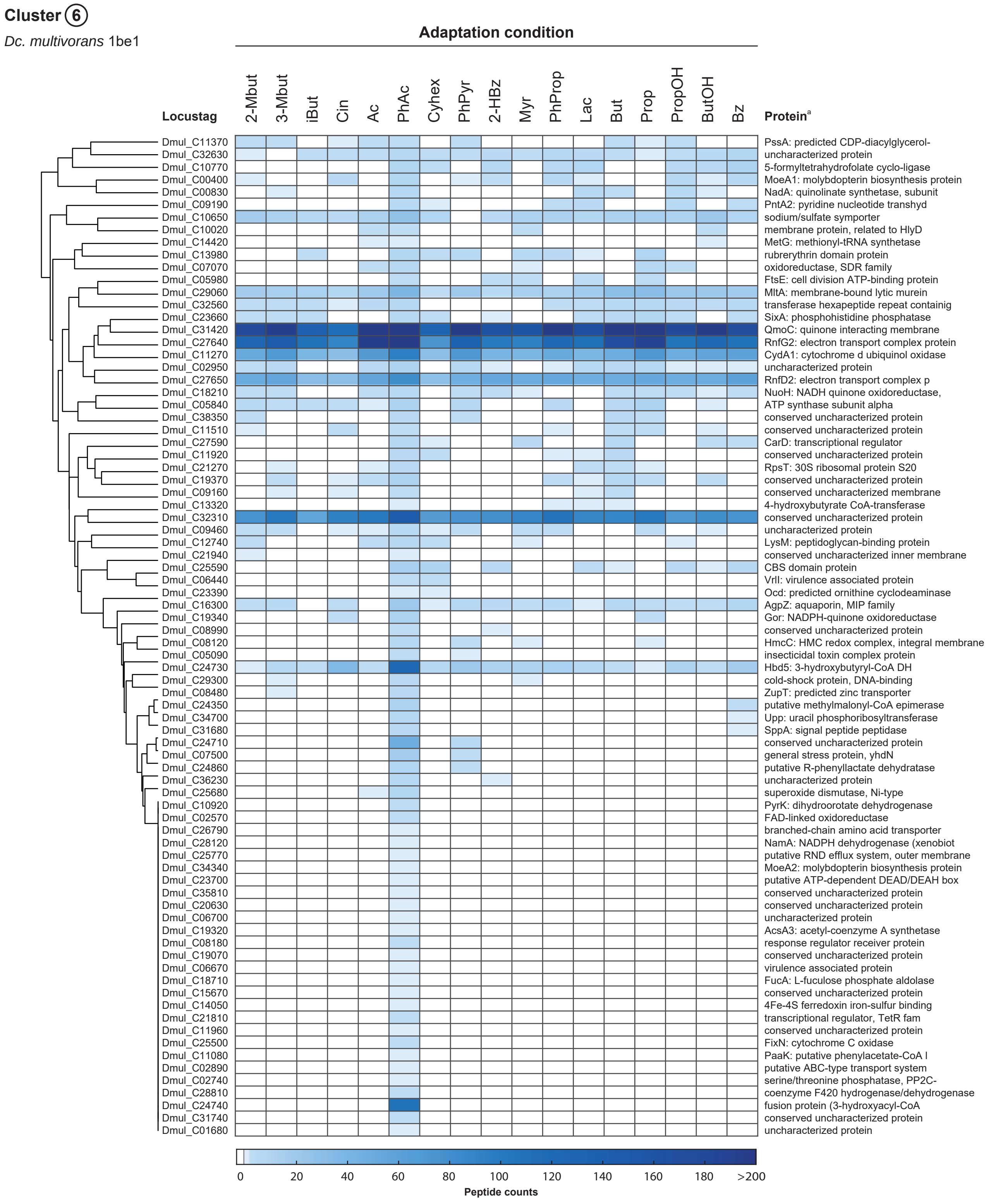

Fig. S87: Detailed view on abundances of proteins forming a sub-cluster in the global clustering depicted in Fig. S9. The global clustering is based on standardized protein abundances of all detected proteins of *Dc. multivorans* 1be1 across the 17 studied substrate adaptation conditions.
[a] Full description of protein functions are available in the genome annotation under accession CP015381.

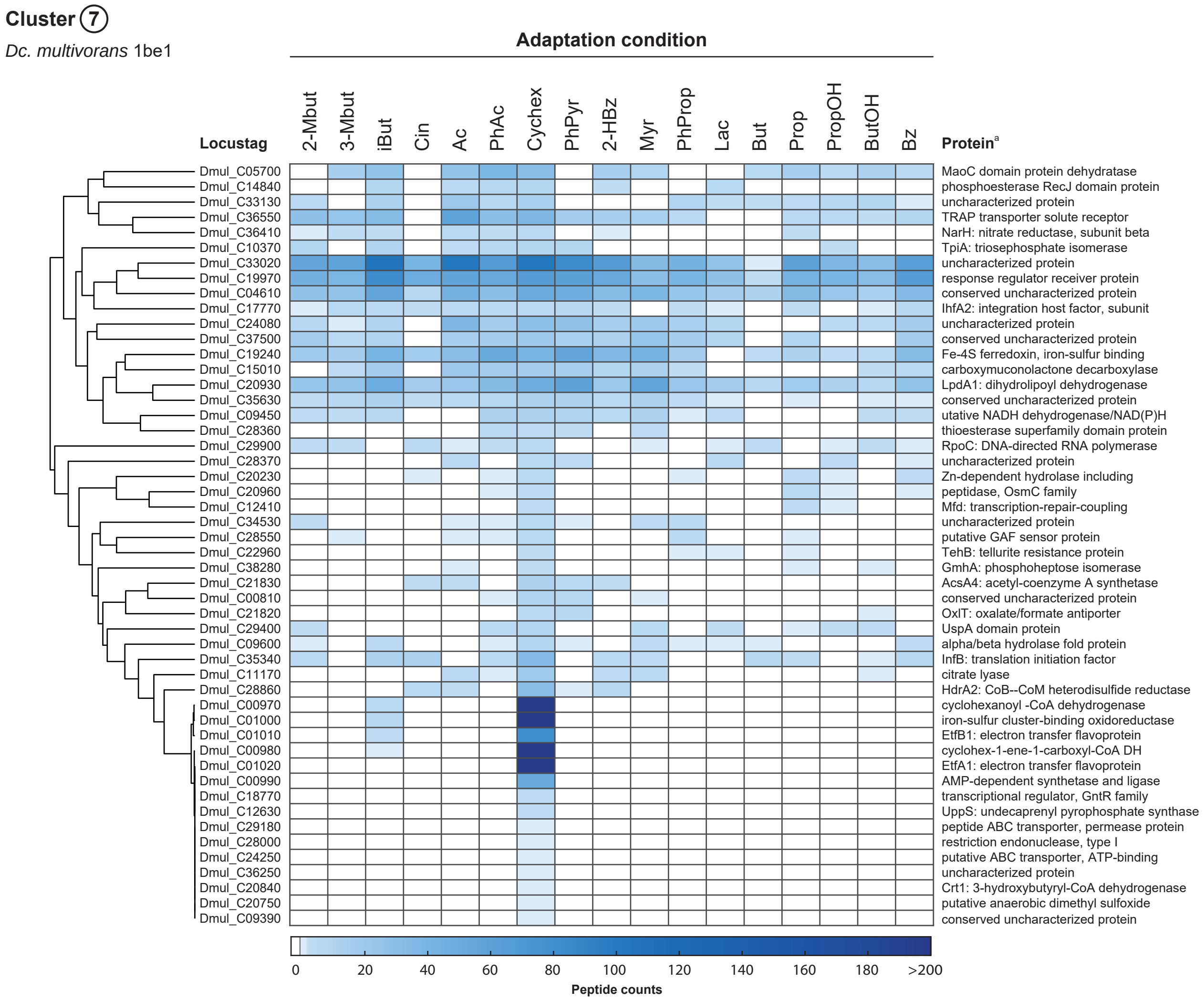

Fig. S88: Detailed view on abundances of proteins forming a sub-cluster in the global clustering depicted in Fig. S9. The global clustering is based on standardized protein abundances of all detected proteins of *Dc. multivorans* 1be1 across the 17 studied substrate adaptation conditions.

[a] Full description of protein functions are available in the genome annotation under accession CP015381.

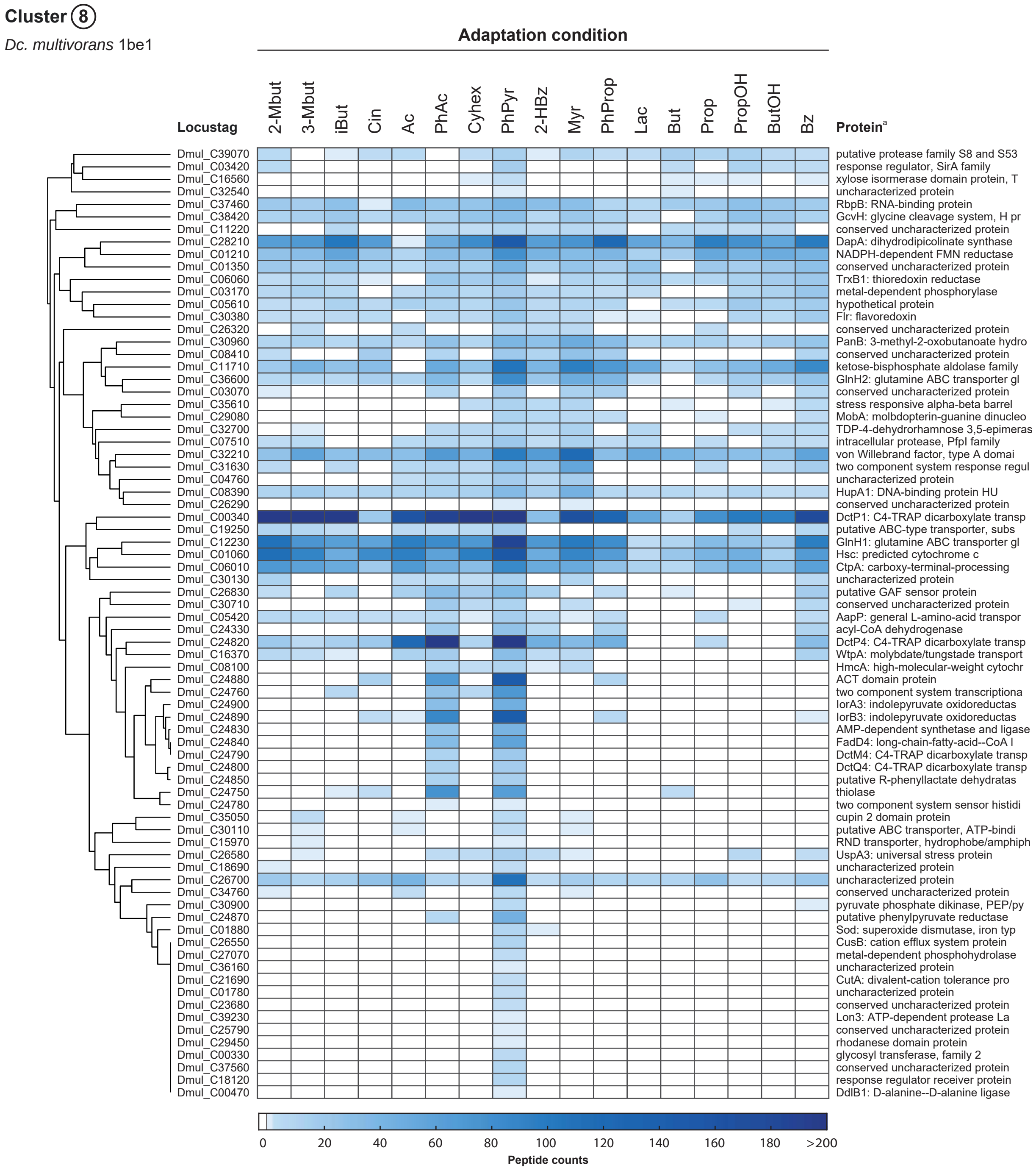

Fig. S89: Detailed view on abundances of proteins forming a sub-cluster in the global clustering depicted in Fig. S9. The global clustering is based on standardized protein abundances of all detected proteins of *Dc. multivorans* 1be1 across the 17 studied substrate adaptation conditions.

[a] Full description of protein functions are available in the genome annotation under accession CP015381.

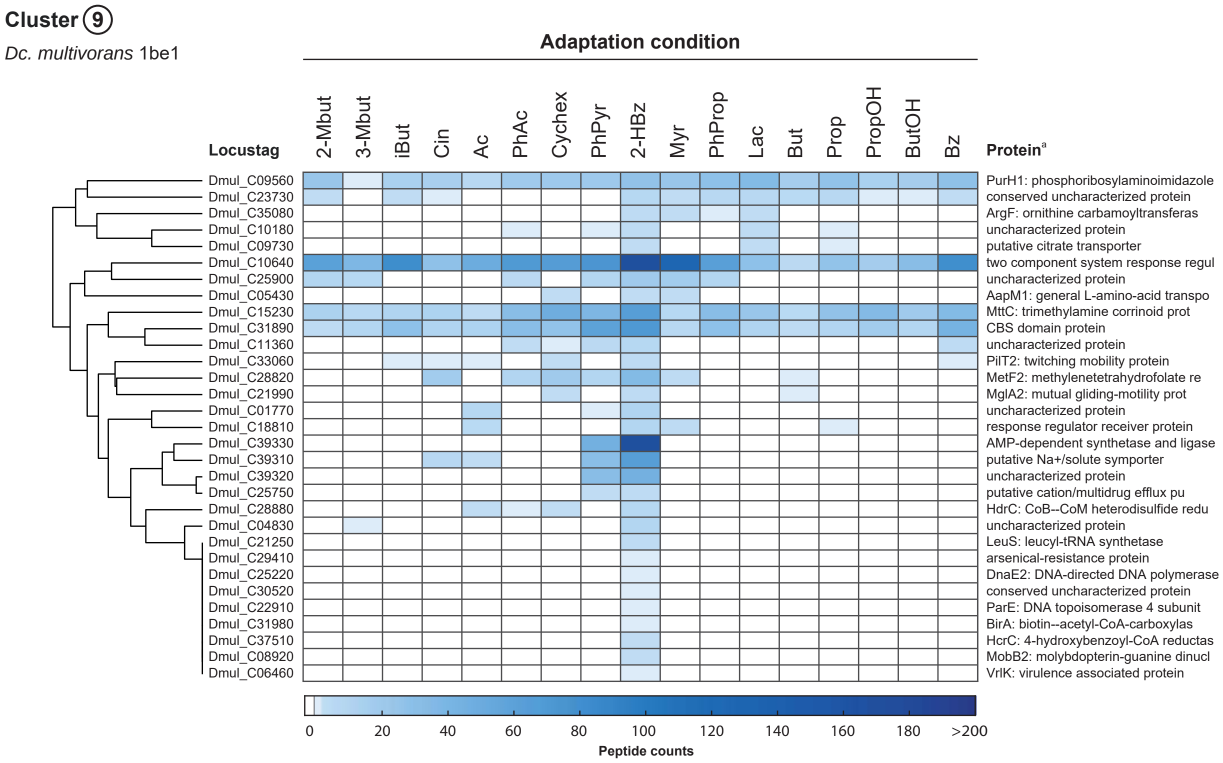

Fig. S90: Detailed view on abundances of proteins forming a sub-cluster in the global clustering depicted in Fig. S9. The global clustering is based on standardized protein abundances of all detected proteins of *Dc. multivorans* 1be1 across the 17 studied substrate adaptation conditions.

[a] Full description of protein functions are available in the genome annotation under accession CP015381.

Cluster (10)

Dc. multivorans 1be1

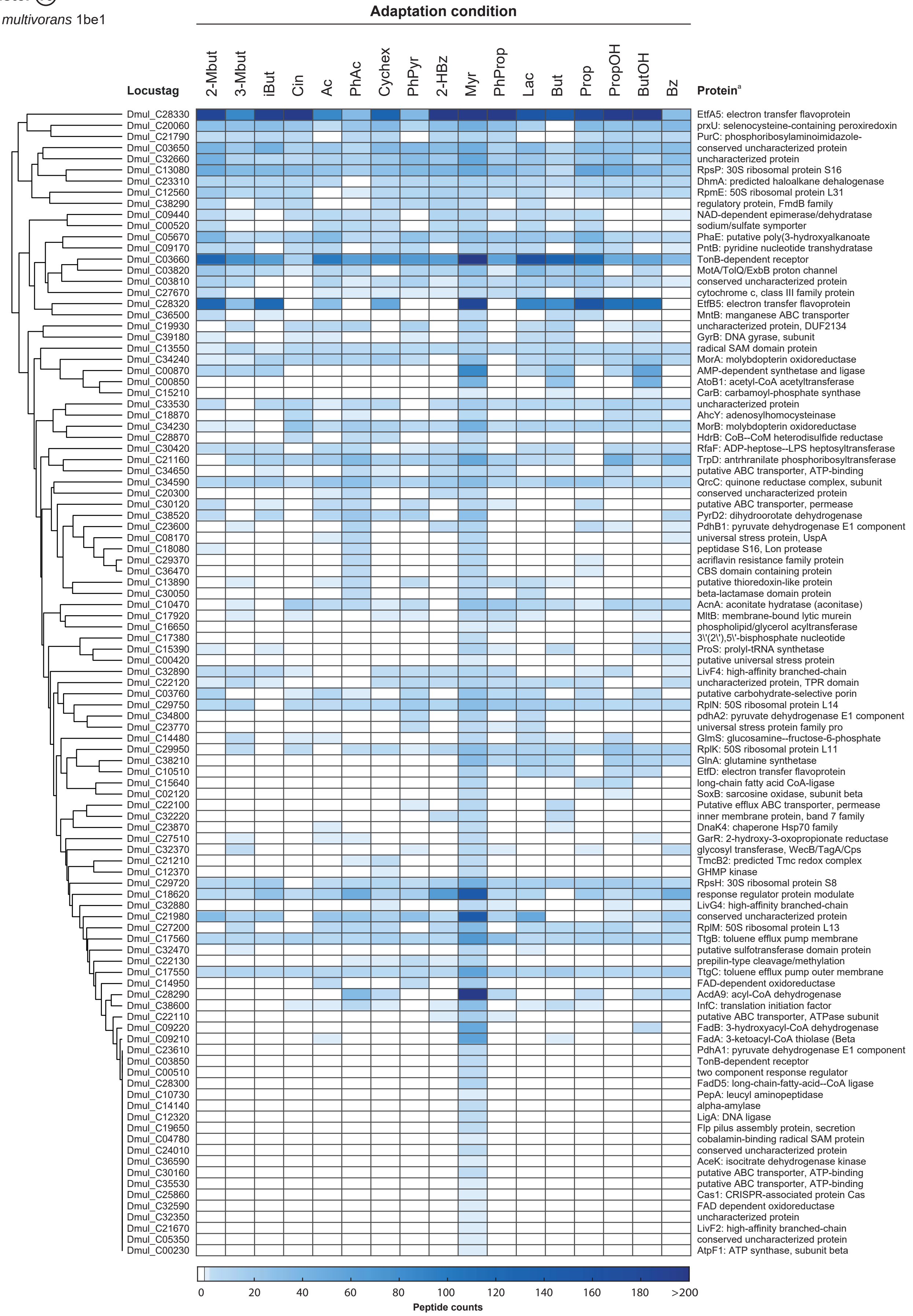

Fig. S91: Detailed view on abundances of proteins forming a sub-cluster in the global clustering depicted in Fig. S9. The global clustering is based on standardized protein abundances of all detected proteins of *Dc. multivorans* 1be1 across the 17 studied substrate adaptation conditions.

[a] Full description of protein functions are available in the genome annotation under accession CP015381.

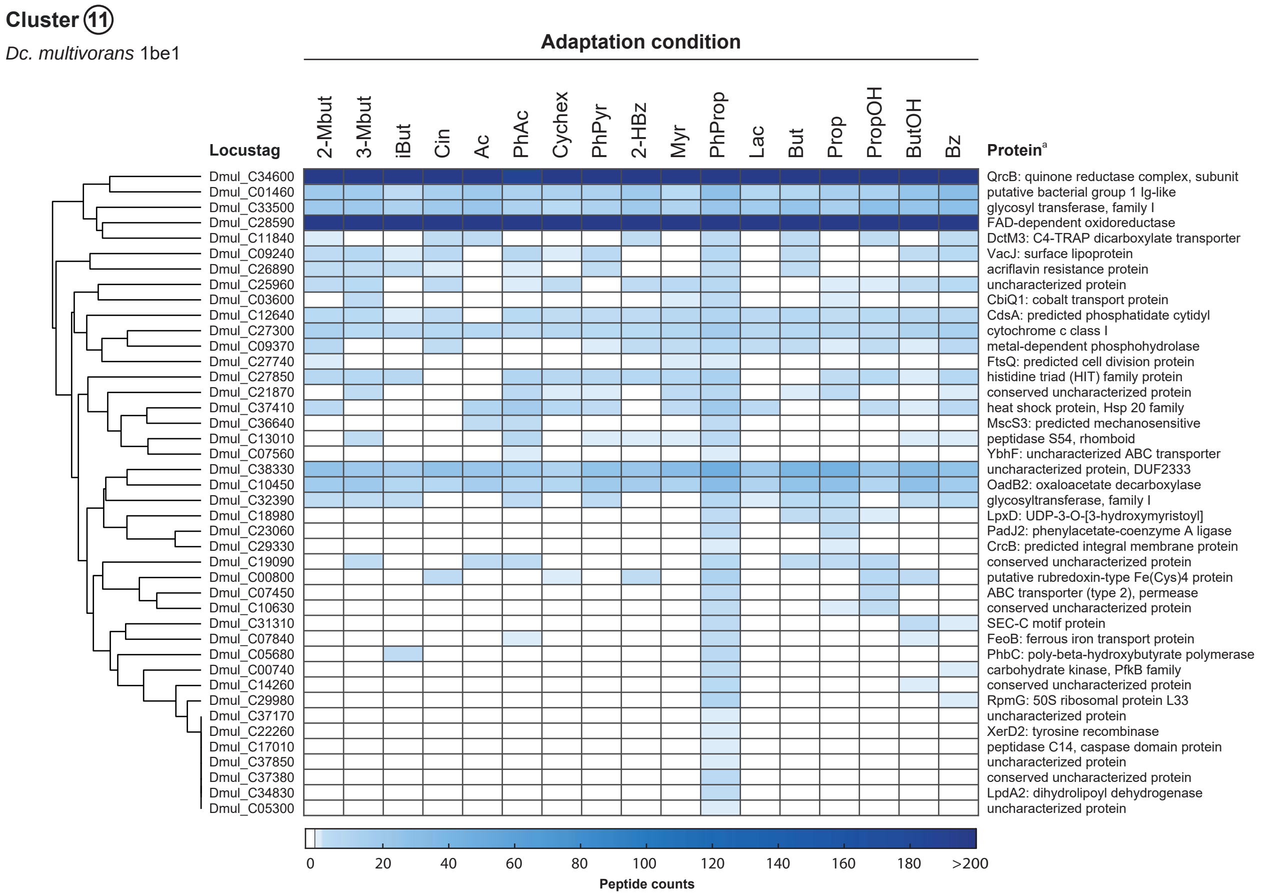

Fig. S92: Detailed view on abundances of proteins forming a sub-cluster in the global clustering depicted in Fig. S9. The global clustering is based on standardized protein abundances of all detected proteins of *Dc. multivorans* 1be1 across the 17 studied substrate adaptation conditions.

[a] Full description of protein functions are available in the genome annotation under accession CP015381.

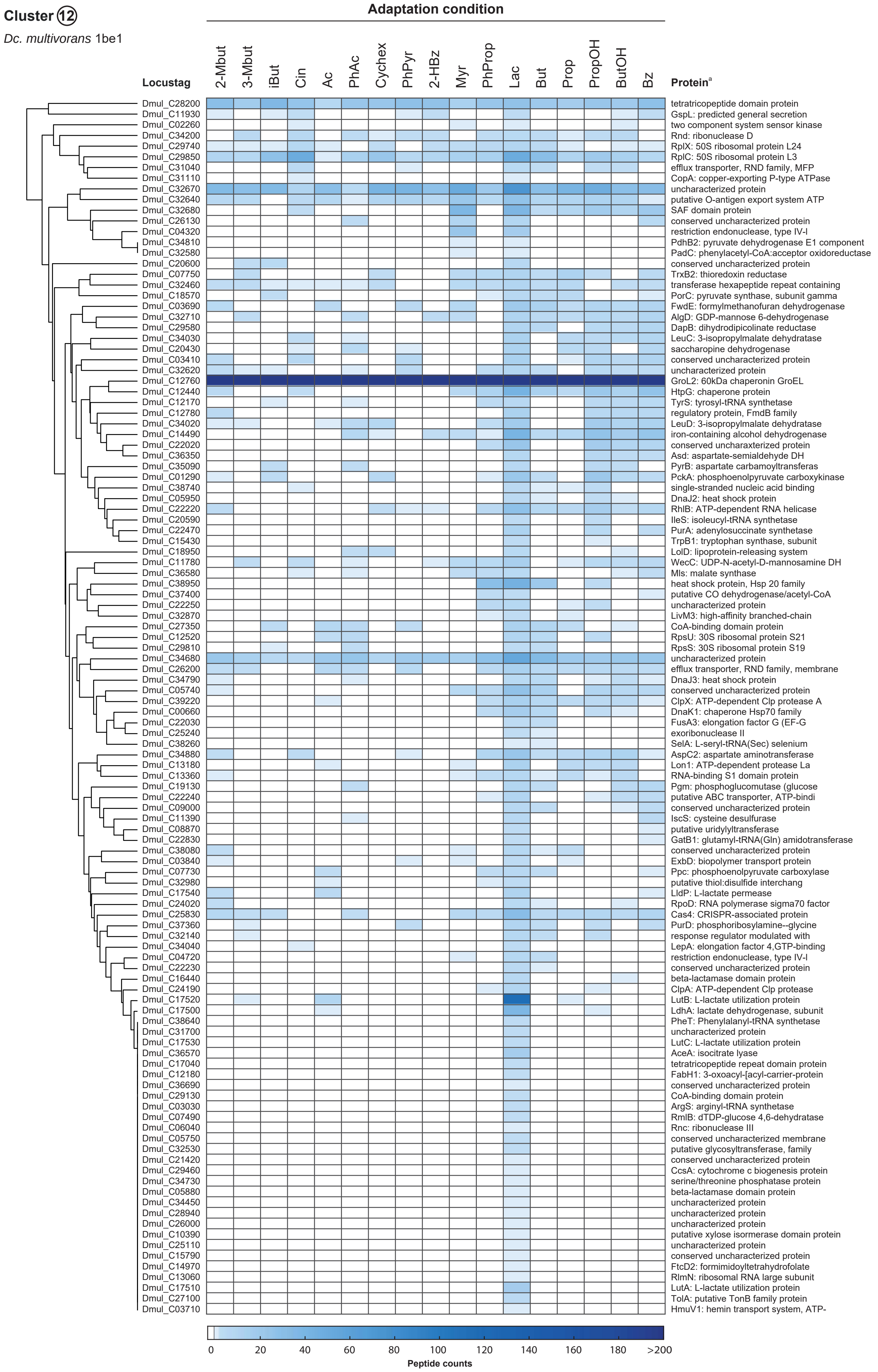

Fig. S93: Detailed view on abundances of proteins forming a sub-cluster in the global clustering depicted in Fig. S9. The global clustering is based on standardized protein abundances of all detected proteins of *Dc. multivorans* 1be1 across the 17 studied substrate adaptation conditions.

[a] Full description of protein functions are available in the genome annotation under accession CP015381.

Cluster 13

Dc. multivorans 1be1

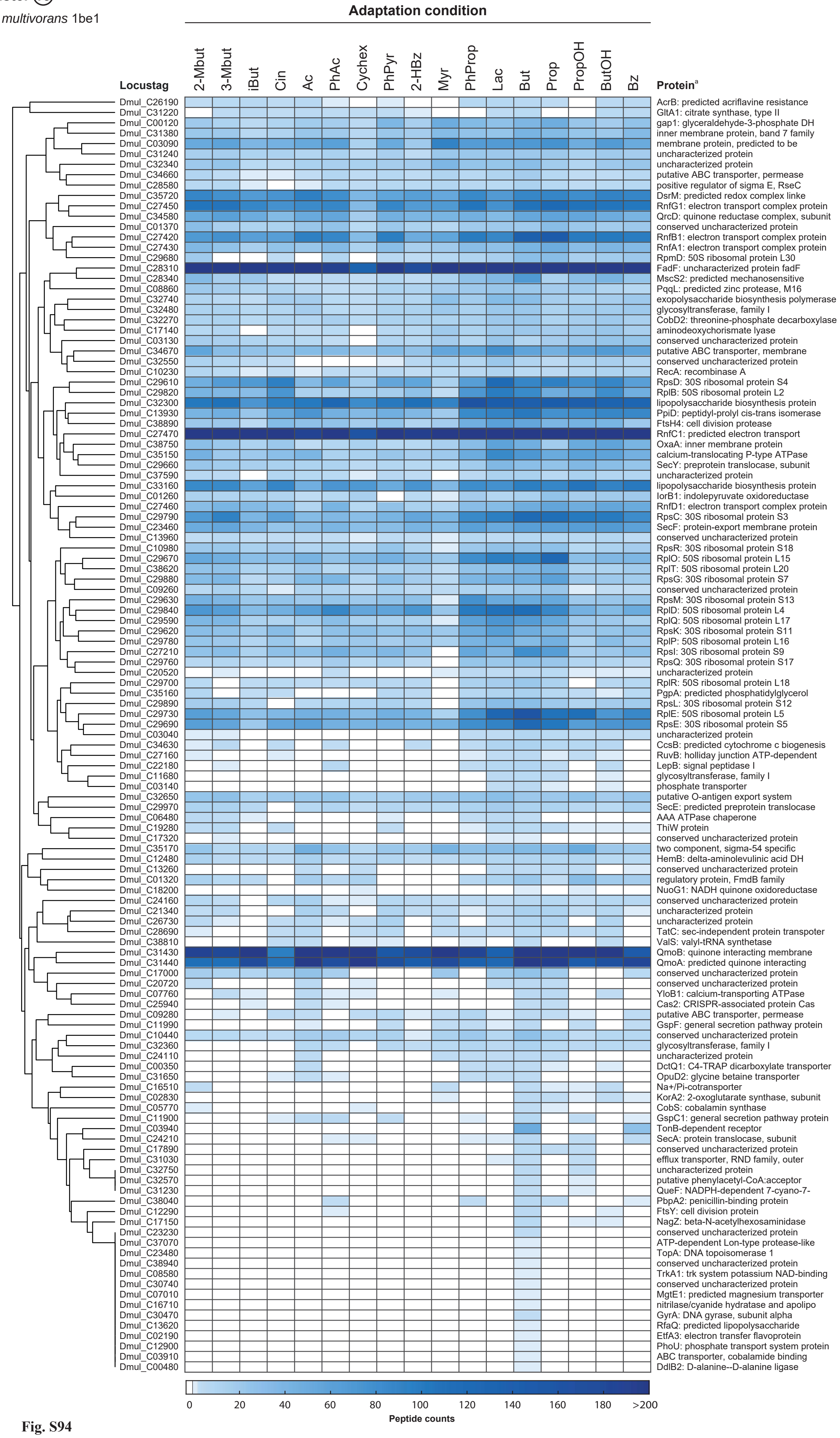

Fig. S94

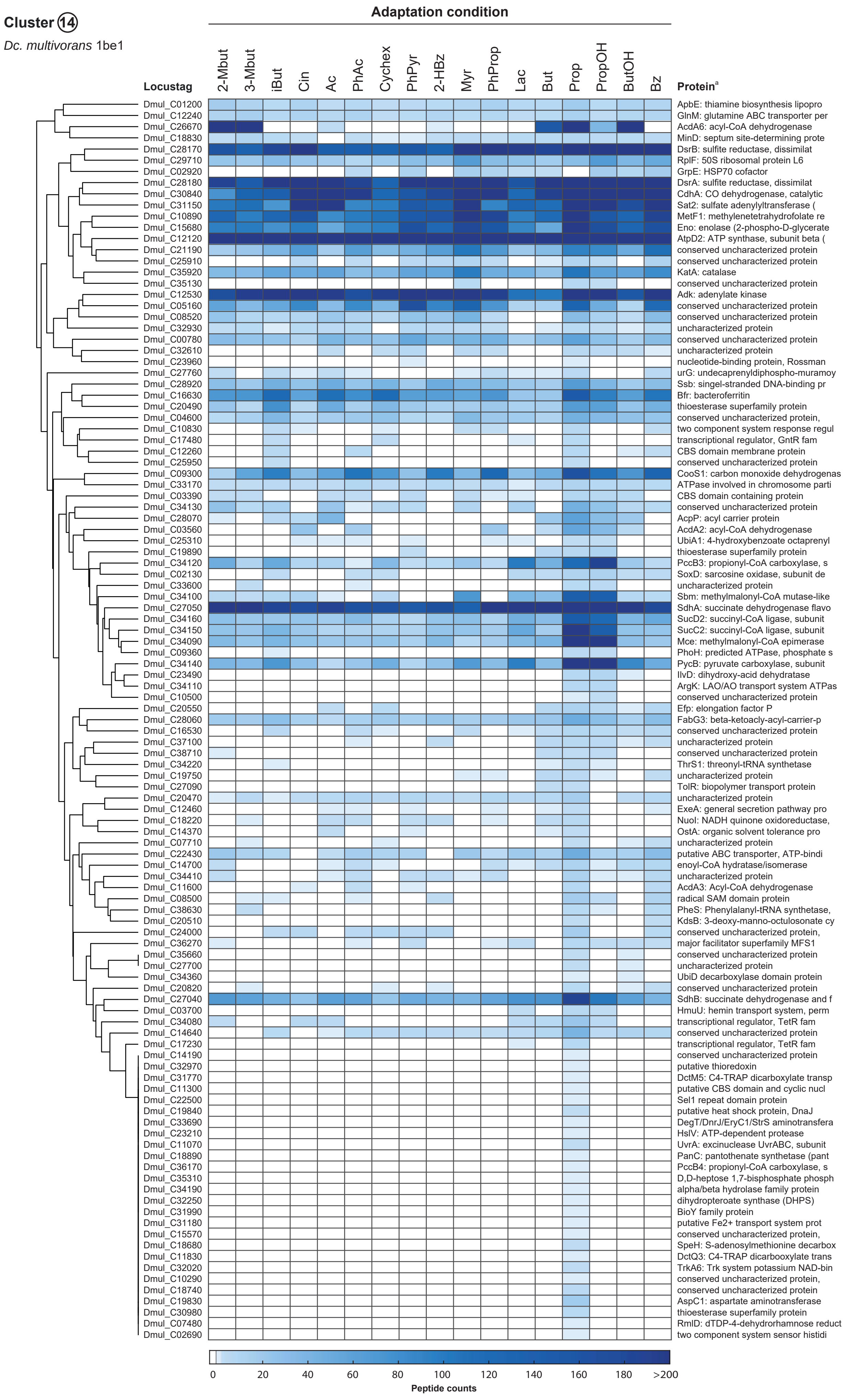

Fig. S95: Detailed view on abundances of proteins forming a sub-cluster in the global clustering depicted in Fig. S9. The global clustering is based on standardized protein abundances of all detected proteins of *Dc. multivorans* 1be1 across the 17 studied substrate adaptation conditions.

[a] Full description of protein functions are available in the genome annotation under accession CP015381.

Cluster 15

Dc. multivorans 1be1

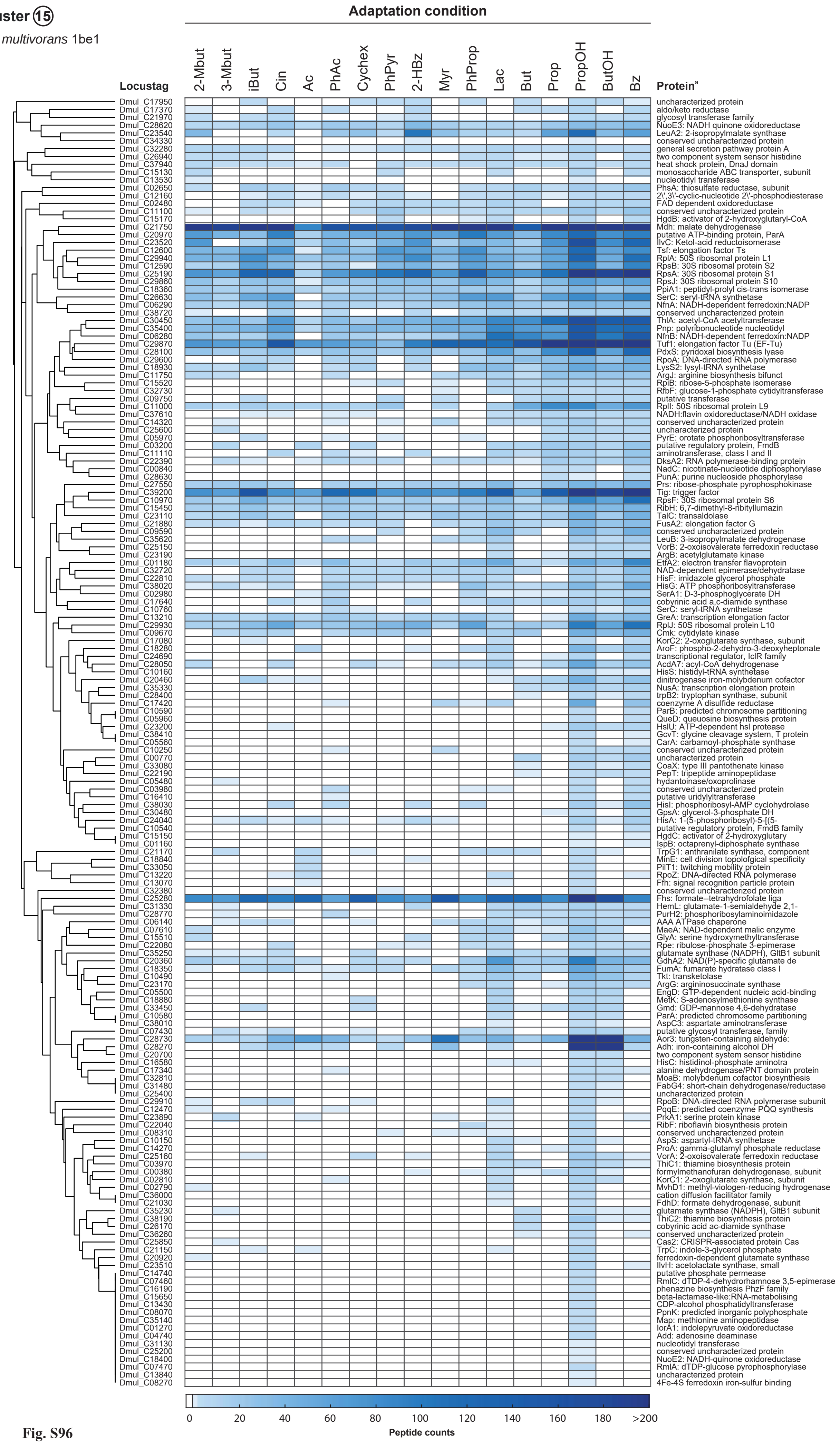

Fig. S96

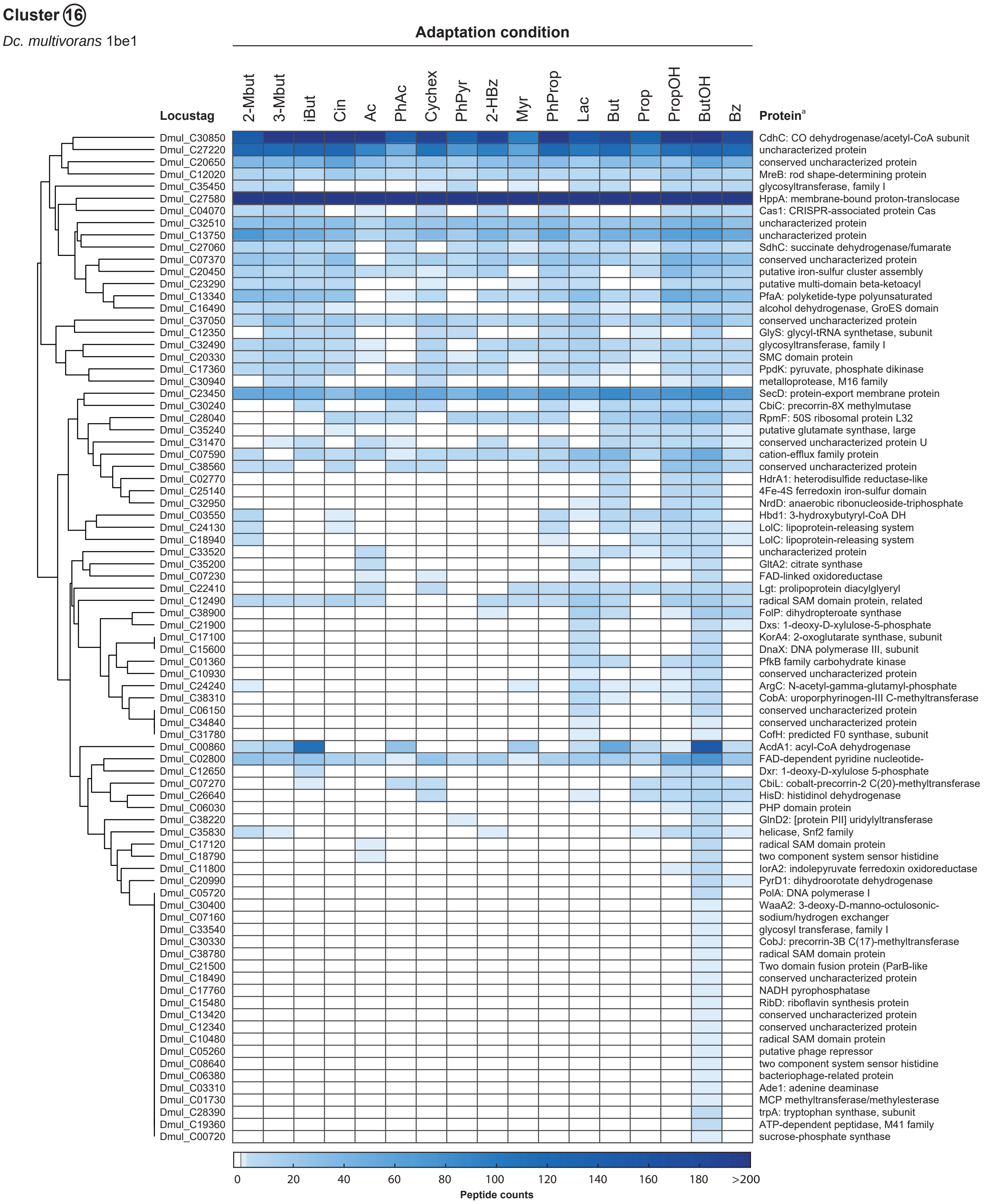

Fig. S97: Detailed view on abundances of proteins forming a sub-cluster in the global clustering depicted in Fig. S9. The global clustering is based on standardized protein abundances of all detected proteins of *Dc. multivorans* 1be1 across the 17 studied substrate adaptation conditions.

[a] Full description of protein functions are available in the genome annotation under accession CP015381.

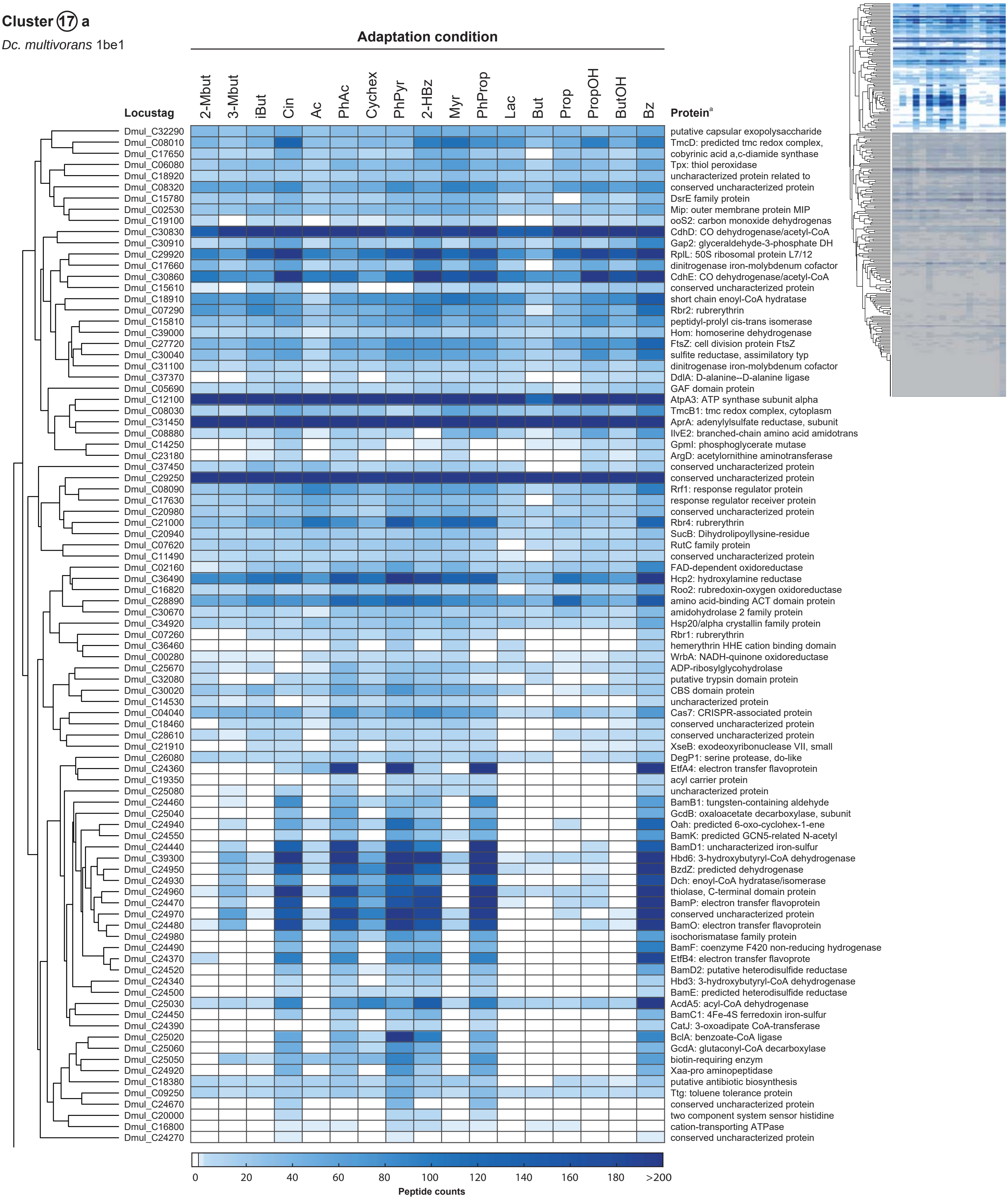

Fig. S98: Detailed view on abundances of proteins forming a sub-cluster in the global clustering depicted in Fig. S9. The global clustering is based on standardized protein abundances of all detected proteins of *Dc. multivorans* 1be1 across the 17 studied substrate adaptation conditions.

[a] Full description of protein functions are available in the genome annotation under accession CP015381.

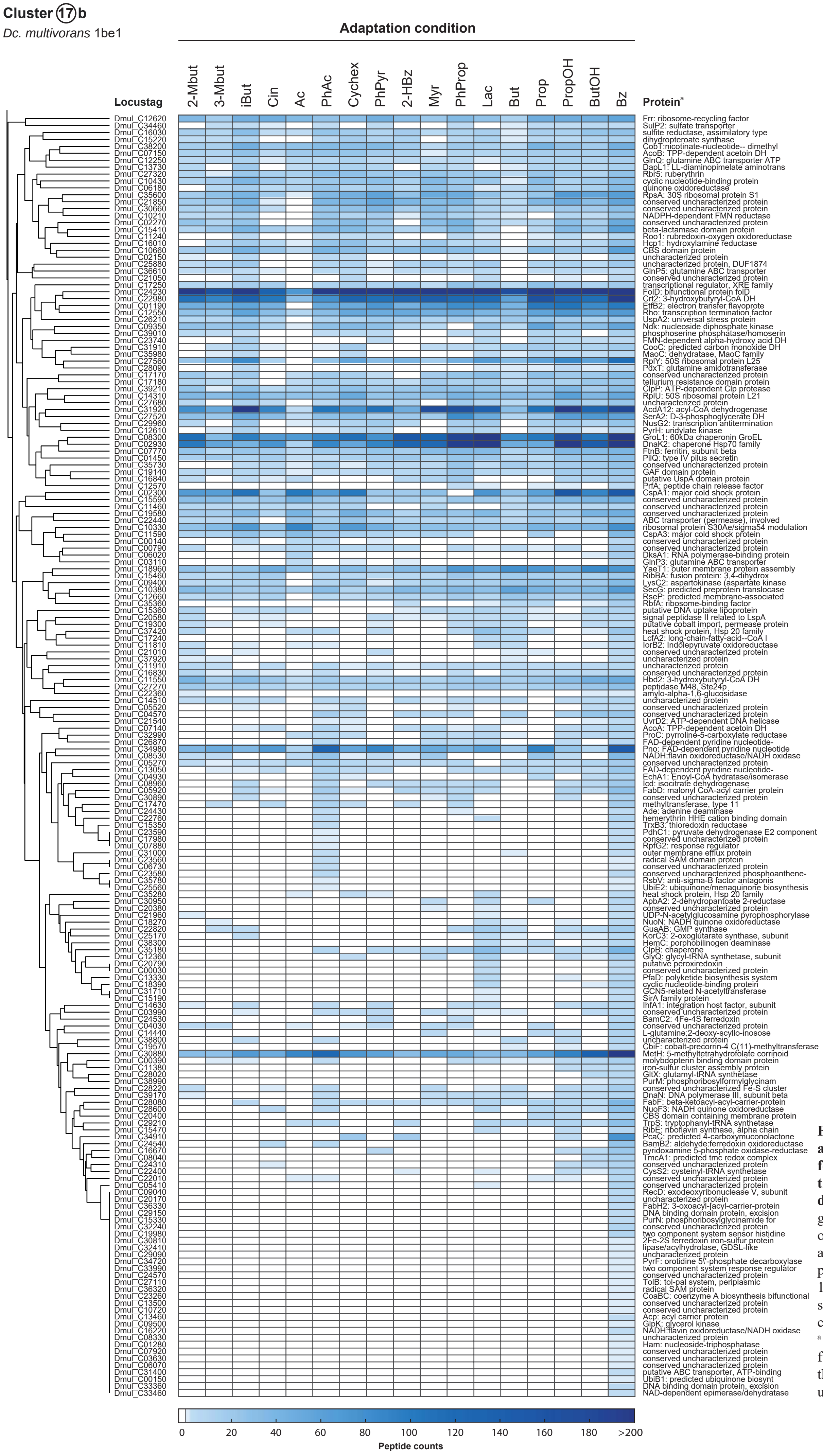

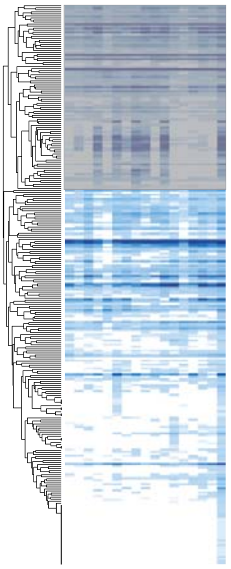

Fig. S99: Detailed view on abundances of proteins forming a sub-cluster in the global clustering depicted in Fig. S9. The global clustering is based on standardized protein abundances of all detected proteins of *Dc. multivorans* 1be1 across the 17 studied substrate adaptation conditions.

[a] Full description of protein functions are available in the genome annotation under accession CP015381.

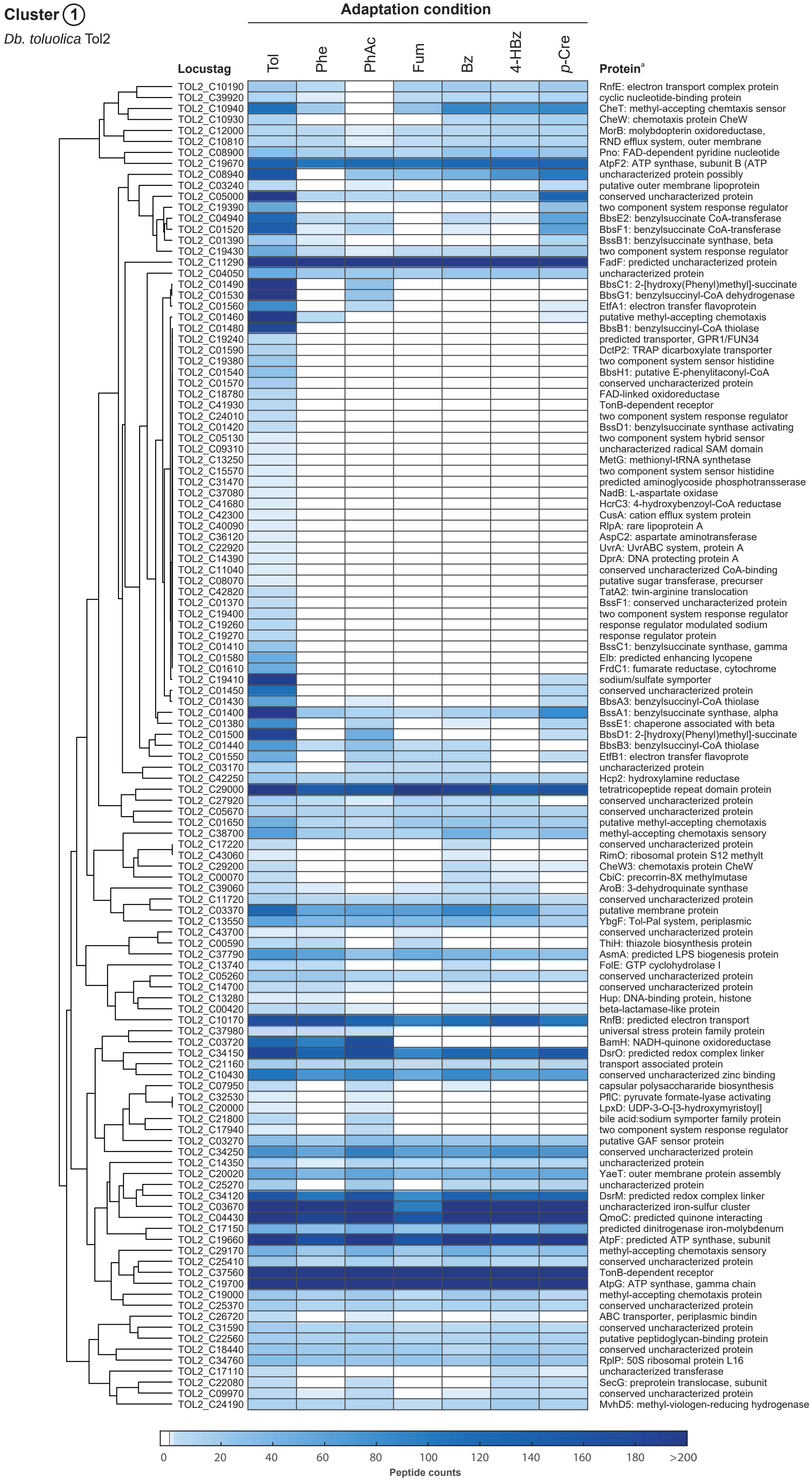

Fig. S100: Detailed view on abundances of proteins forming a sub-cluster in the global clustering depicted in Fig. S10. The global clustering is based on standardized protein abundances of all detected proteins of *Db. toluolica* Tol2 across the 7 studied substrate adaptation conditions.

[a] Full description of protein functions are available in the genome annotation under accession FO203503.

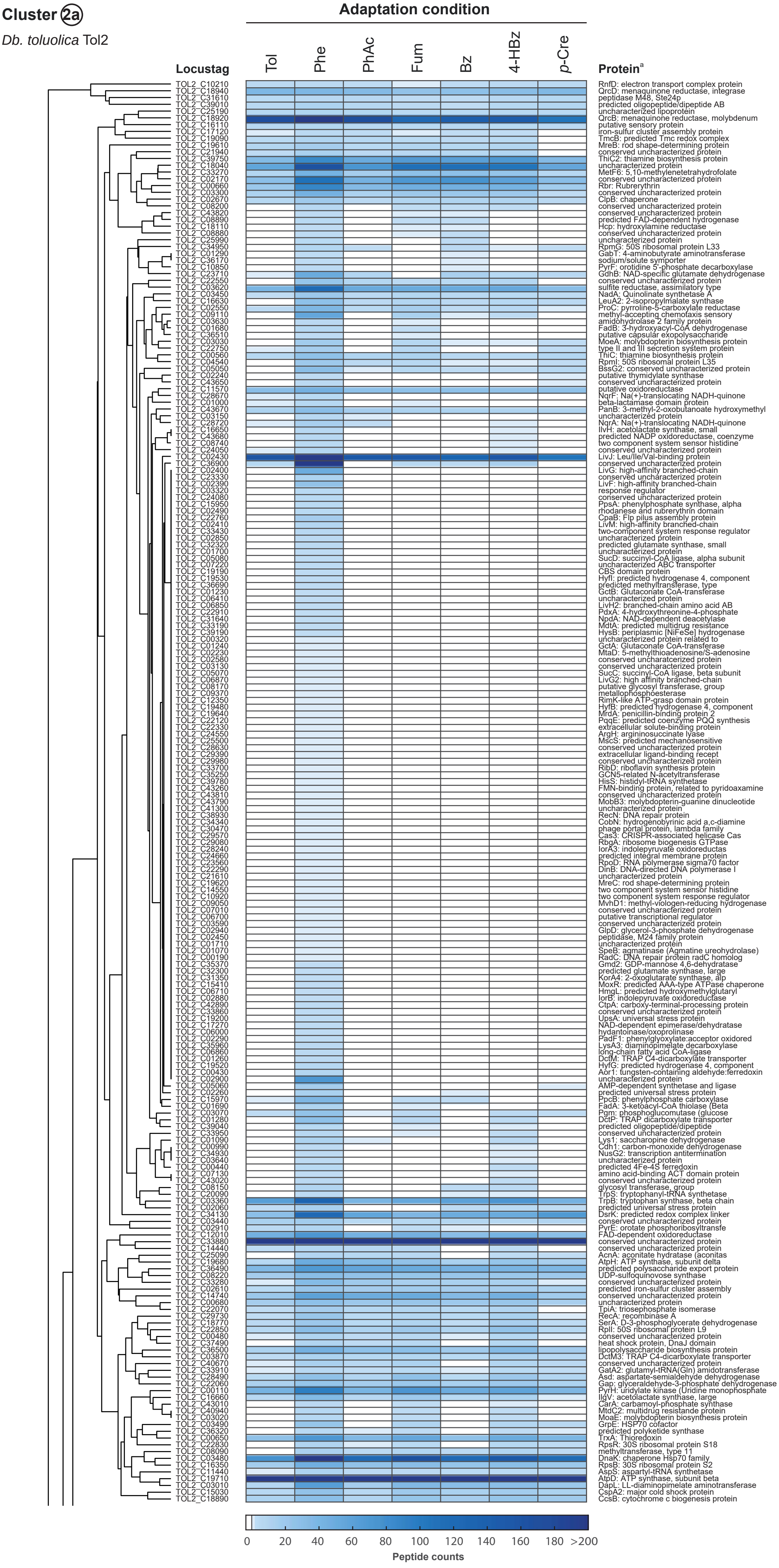

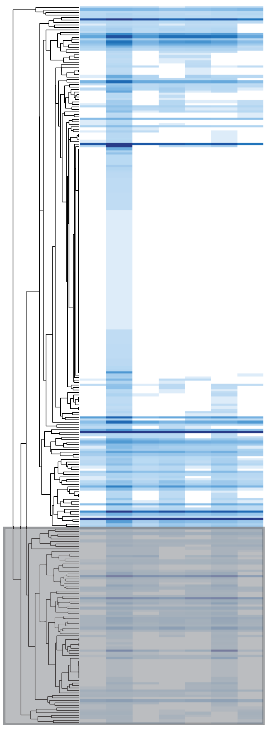

Fig. S101: Detailed view on abundances of proteins forming a sub-cluster in the global clustering depicted in Fig. S10. The global clustering is based on standardized protein abundances of all detected proteins of *Db. toluolica* Tol2 across the 7 studied substrate adaptation conditions.

[a] Full description of protein functions are available in the genome annotation under accession FO203503.

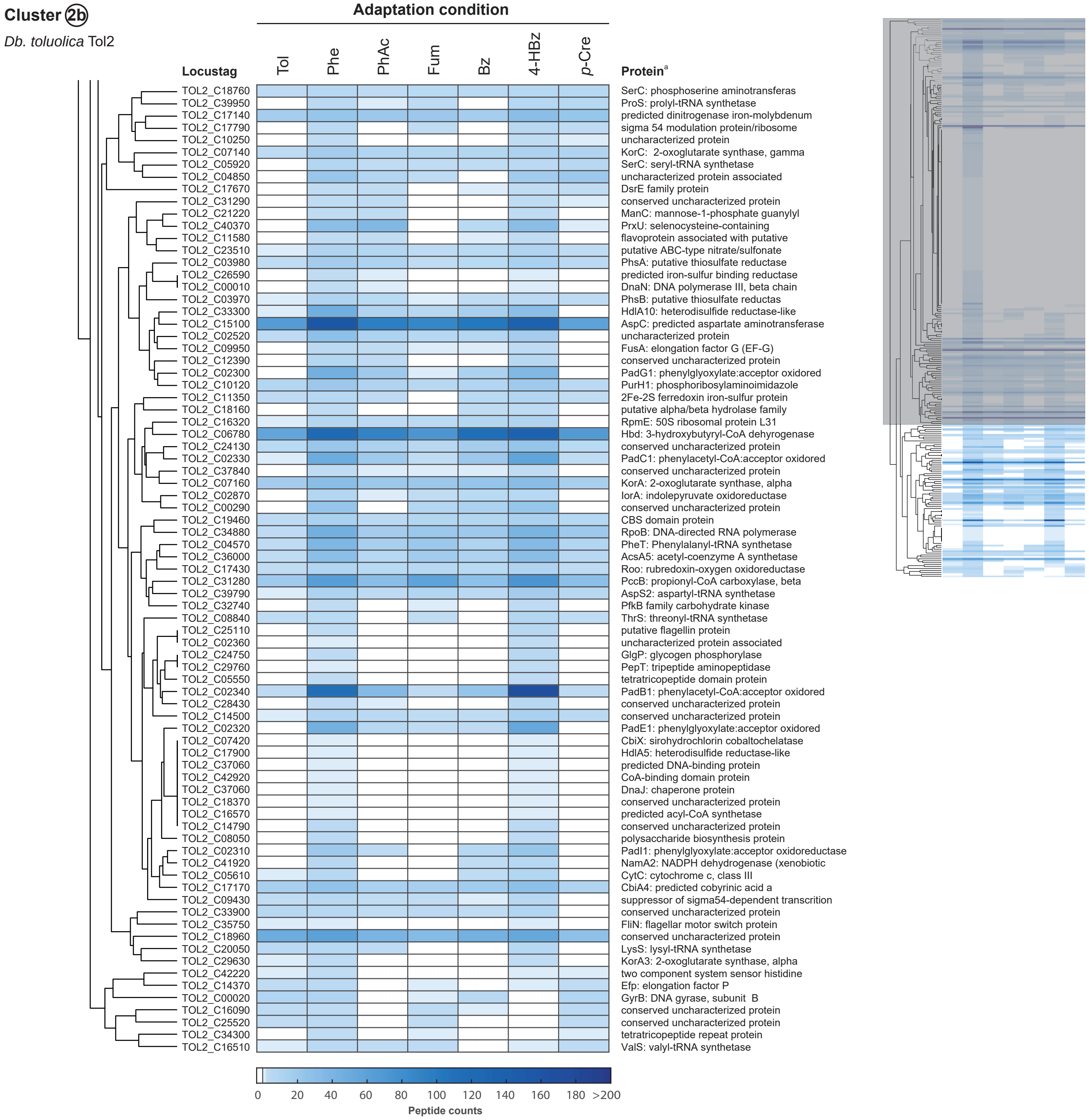

Fig. S102: Detailed view on abundances of proteins forming a sub-cluster in the global clustering depicted in Fig. S10. The global clustering is based on standardized protein abundances of all detected proteins of *Db. toluolica* Tol2 across the 7 studied substrate adaptation conditions.

[a] Full description of protein functions are available in the genome annotation under accession FO203503.

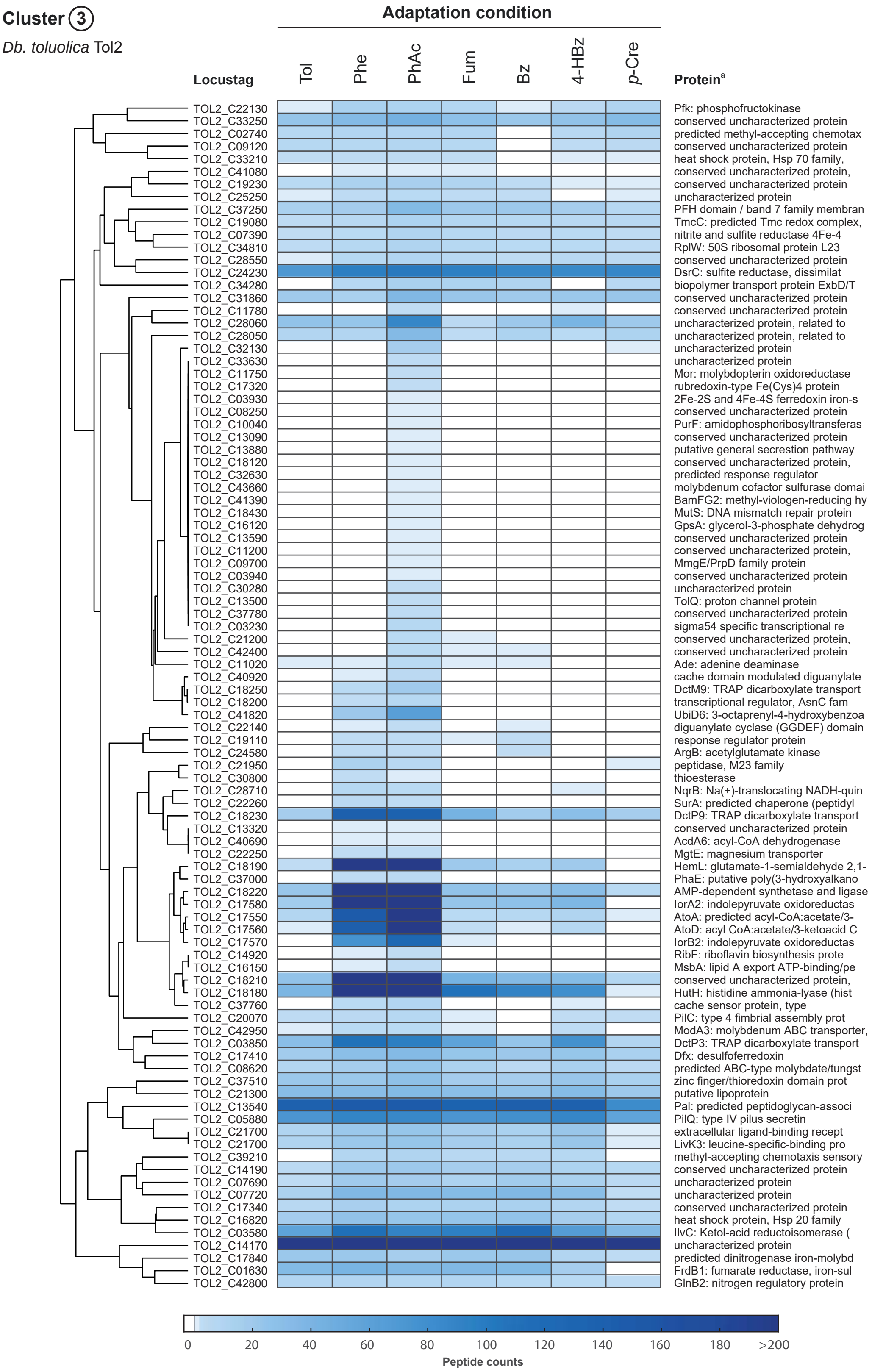

Fig. S103: Detailed view on abundances of proteins forming a sub-cluster in the global clustering depicted in Fig. S10. The global clustering is based on standardized protein abundances of all detected proteins of *Db. toluolica* Tol2 across the 7 studied substrate adaptation conditions.

[a] Full description of protein functions are available in the genome annotation under accession FO203503.

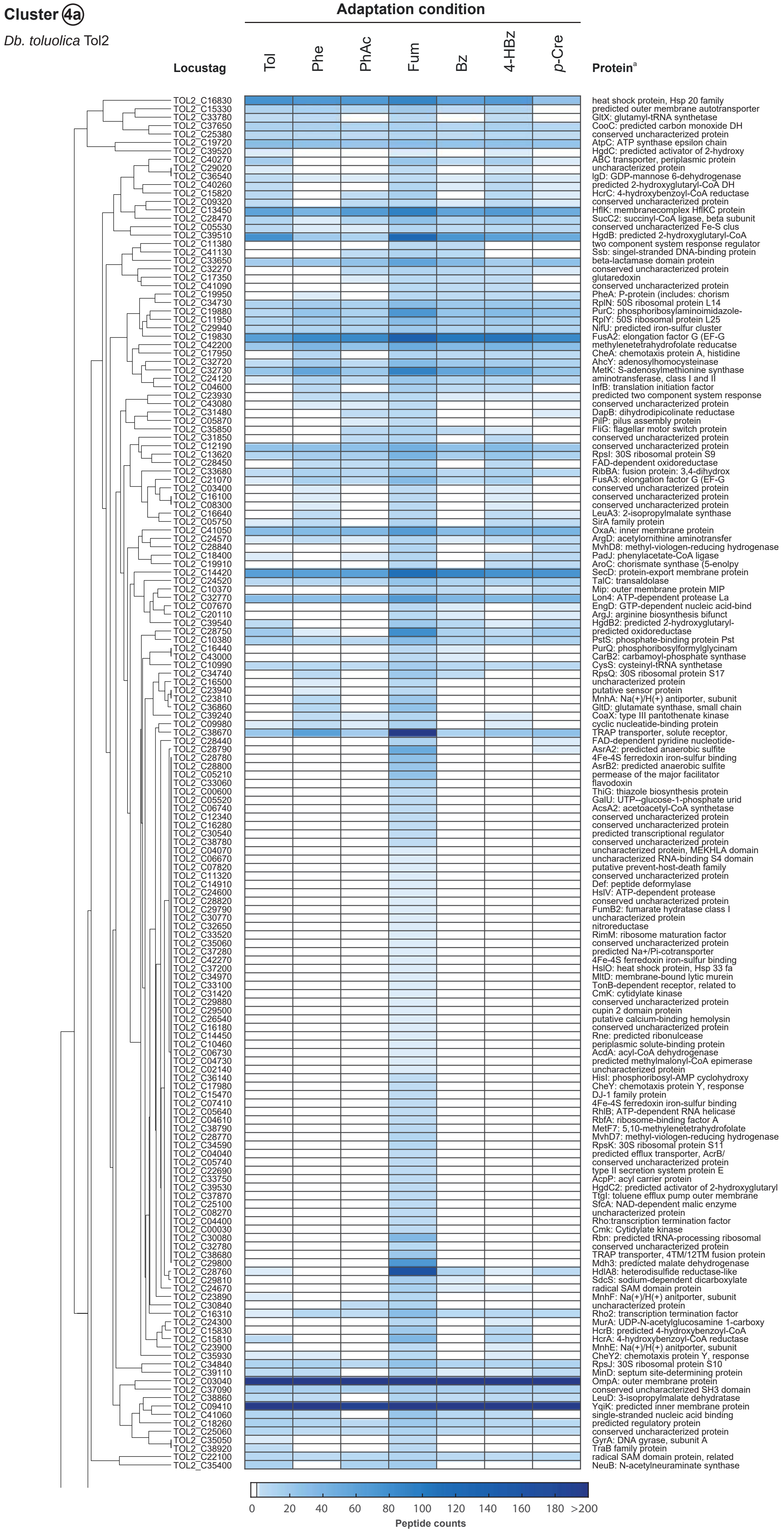

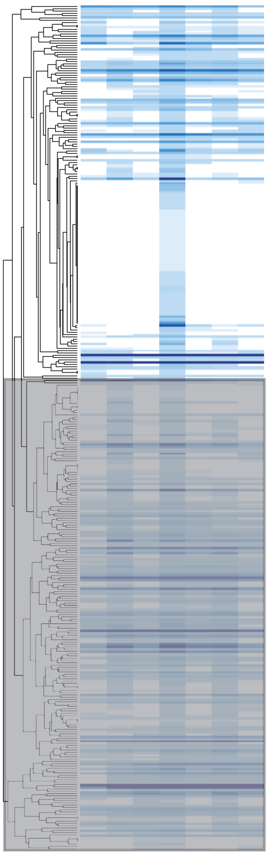

Fig. S104: Detailed view on abundances of proteins forming a sub-cluster in the global clustering depicted in Fig. S10. The global clustering is based on standardized protein abundances of all detected proteins of *Db. toluolica* Tol2 across the 7 studied substrate adaptation conditions.

[a] Full description of protein functions are available in the genome annotation under accession FO203503.

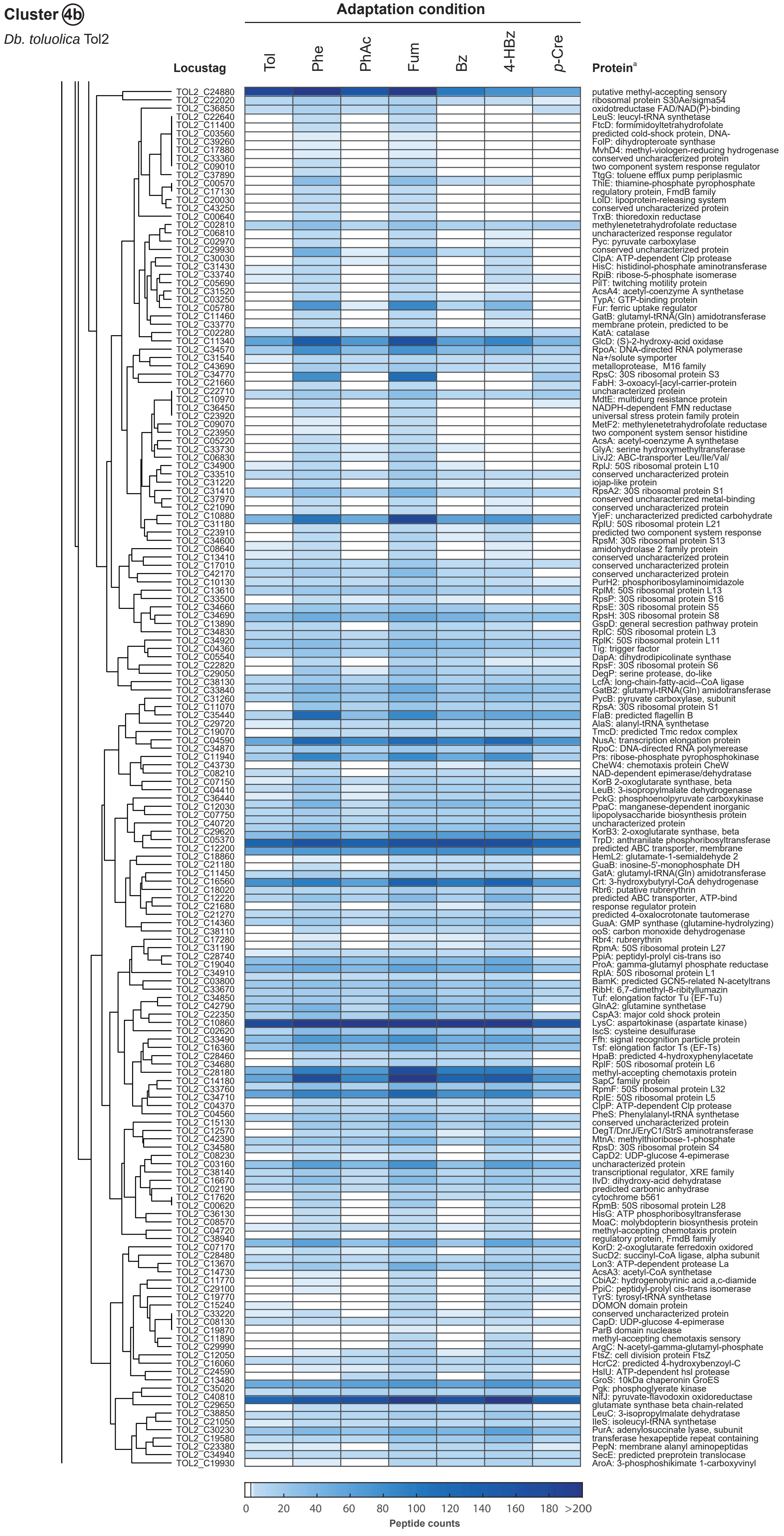

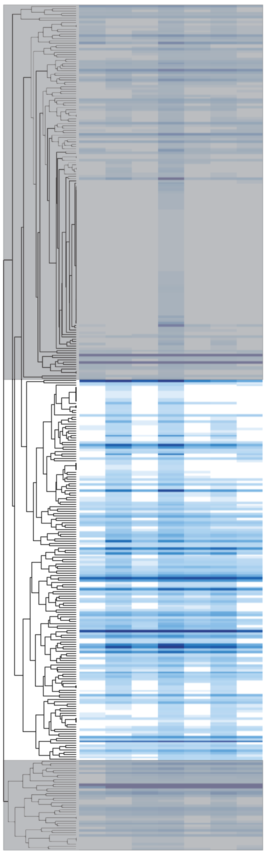

Fig. S105: Detailed view on abundances of proteins forming a sub-cluster in the global clustering depicted in Fig. S10. The global clustering is based on standardized protein abundances of all detected proteins of *Db. toluolica* Tol2 across the 7 studied substrate adaptation conditions.

[a] Full description of protein functions are available in the genome annotation under accession FO203503.

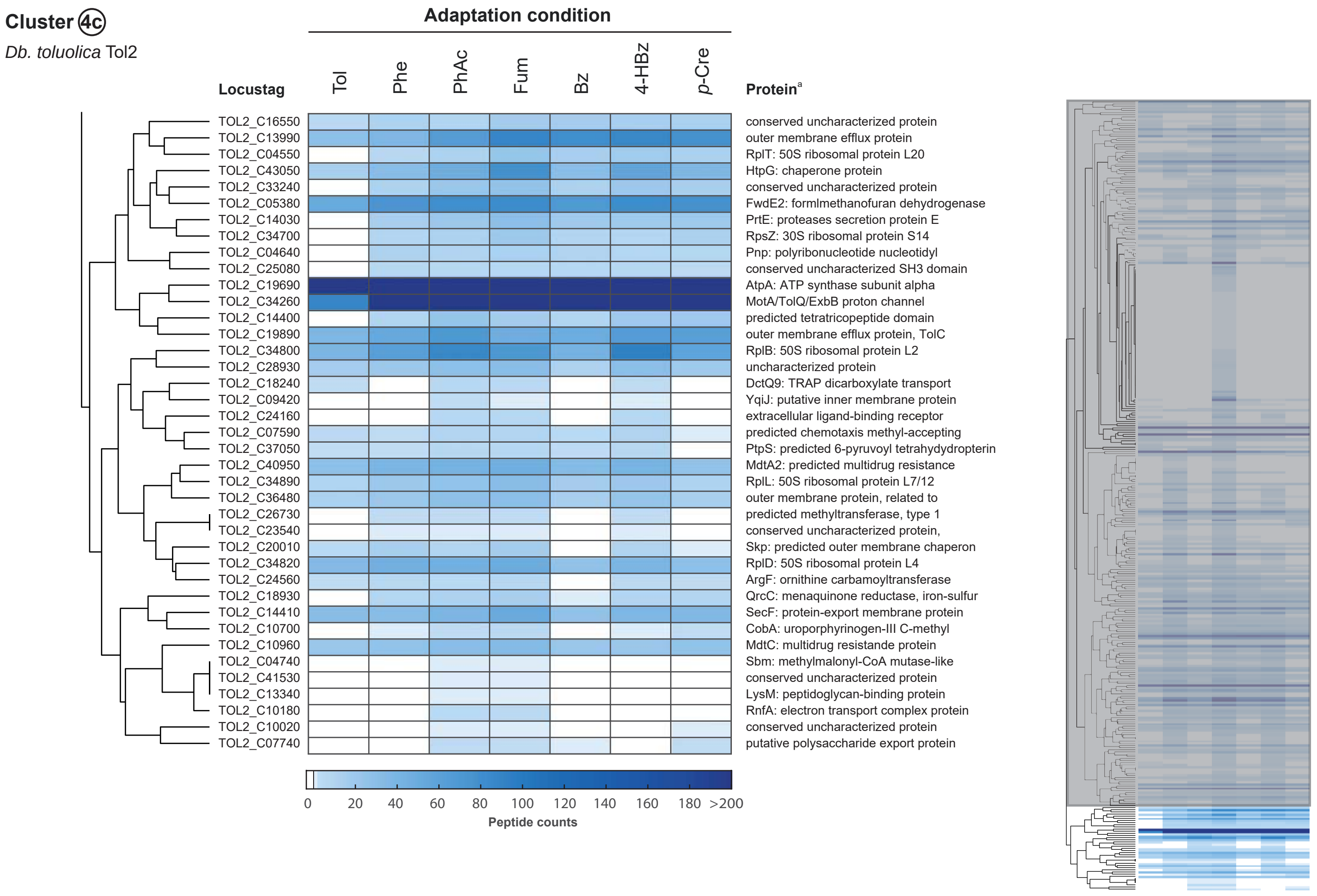

Fig. S106: Detailed view on abundances of proteins forming a sub-cluster in the global clustering depicted in Fig. S10. The global clustering is based on standardized protein abundances of all detected proteins of *Db. toluolica* Tol2 across the 7 studied substrate adaptation conditions.

[a] Full description of protein functions are available in the genome annotation under accession FO203503.

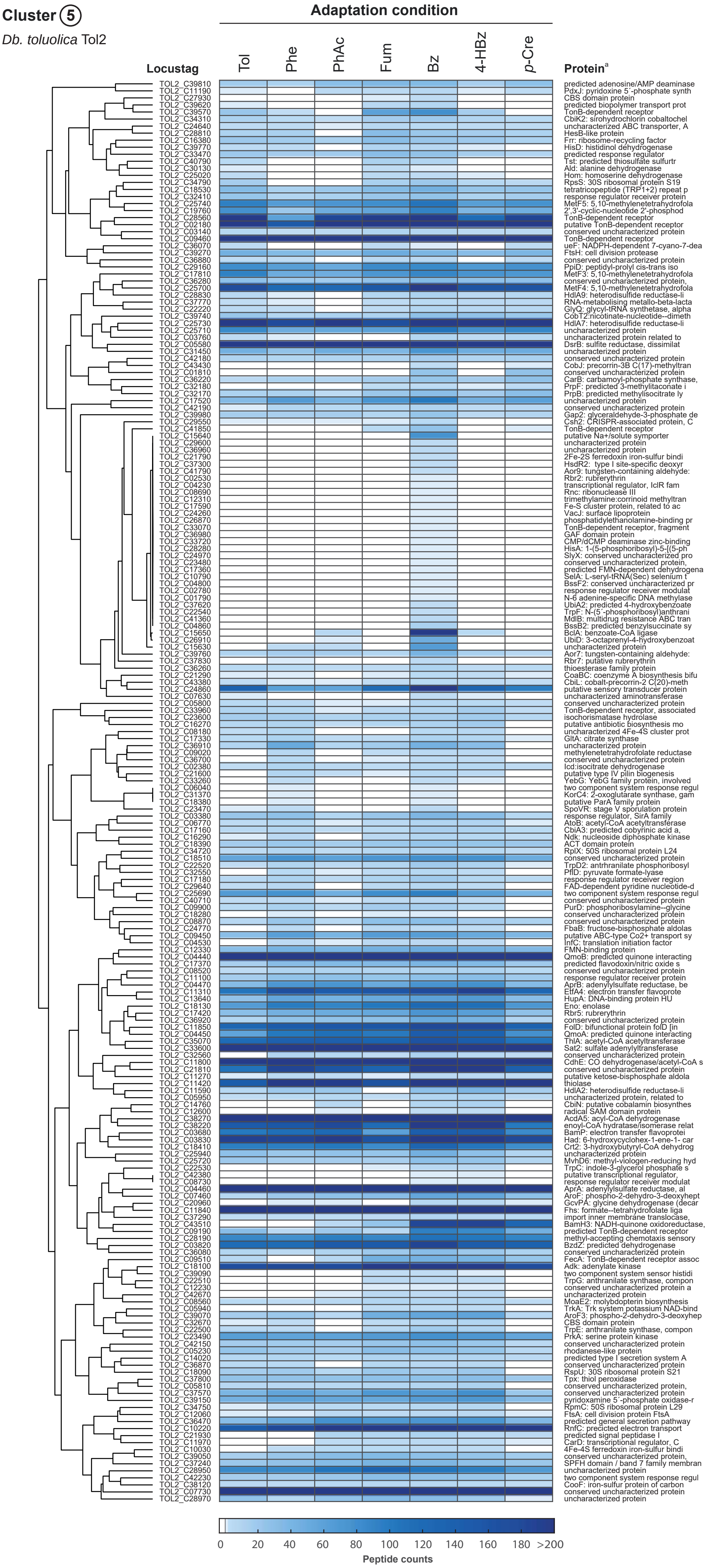

Fig. S107: Detailed view on abundances of proteins forming a sub-cluster in the global clustering depicted in Fig. S10. The global clustering is based on standardized protein abundances of all detected proteins of *Db. toluolica* Tol2 across the 7 studied substrate adaptation conditions.

[a] Full description of protein functions are available in the genome annotation under accession FO203503.

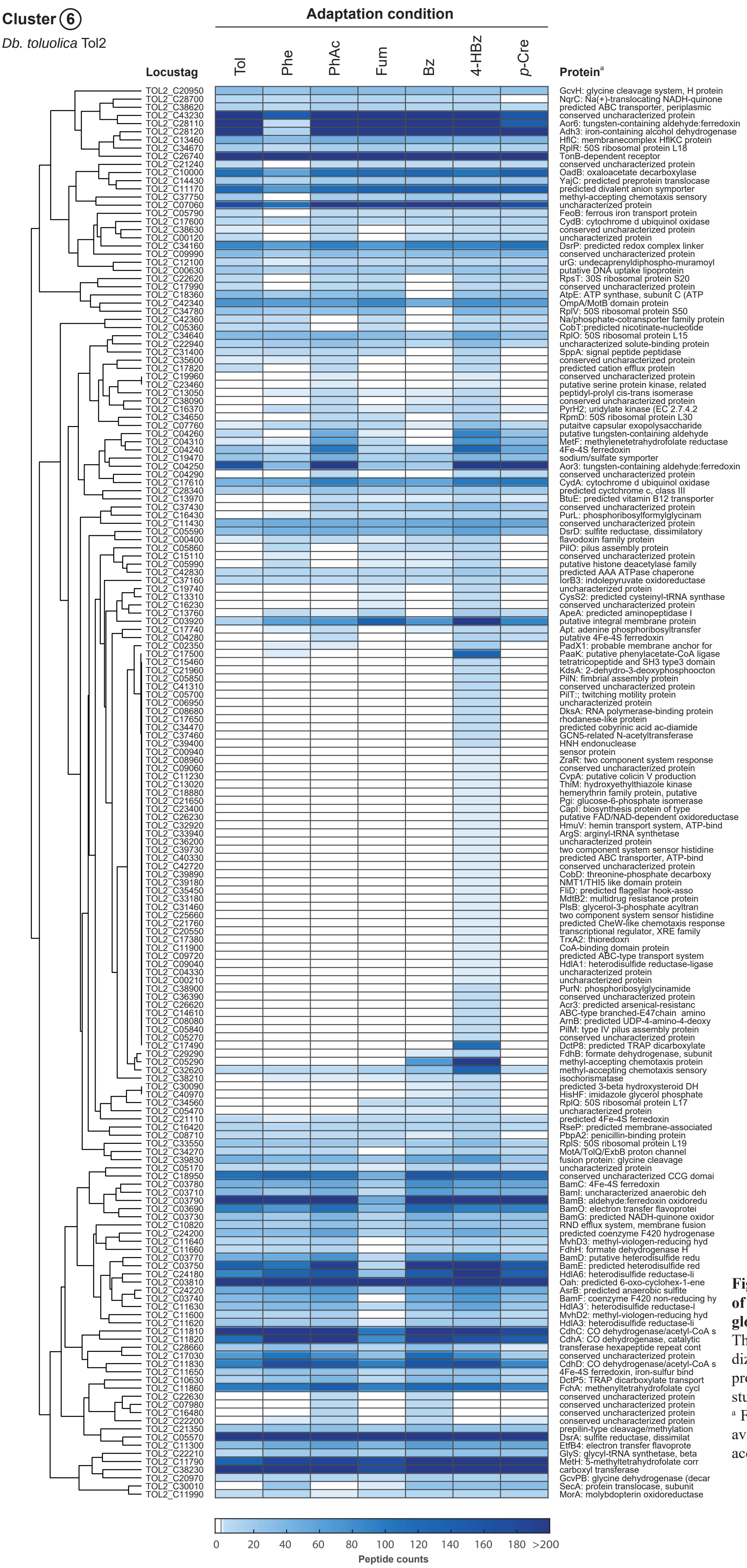

Fig. S108: Detailed view on abundances of proteins forming a sub-cluster in the global clustering depicted in Fig. S10. The global clustering is based on standardized protein abundances of all detected proteins of *Db. toluolica* Tol2 across the 7 studied substrate adaptation conditions.

[a] Full description of protein functions are available in the genome annotation under accession FO203503.

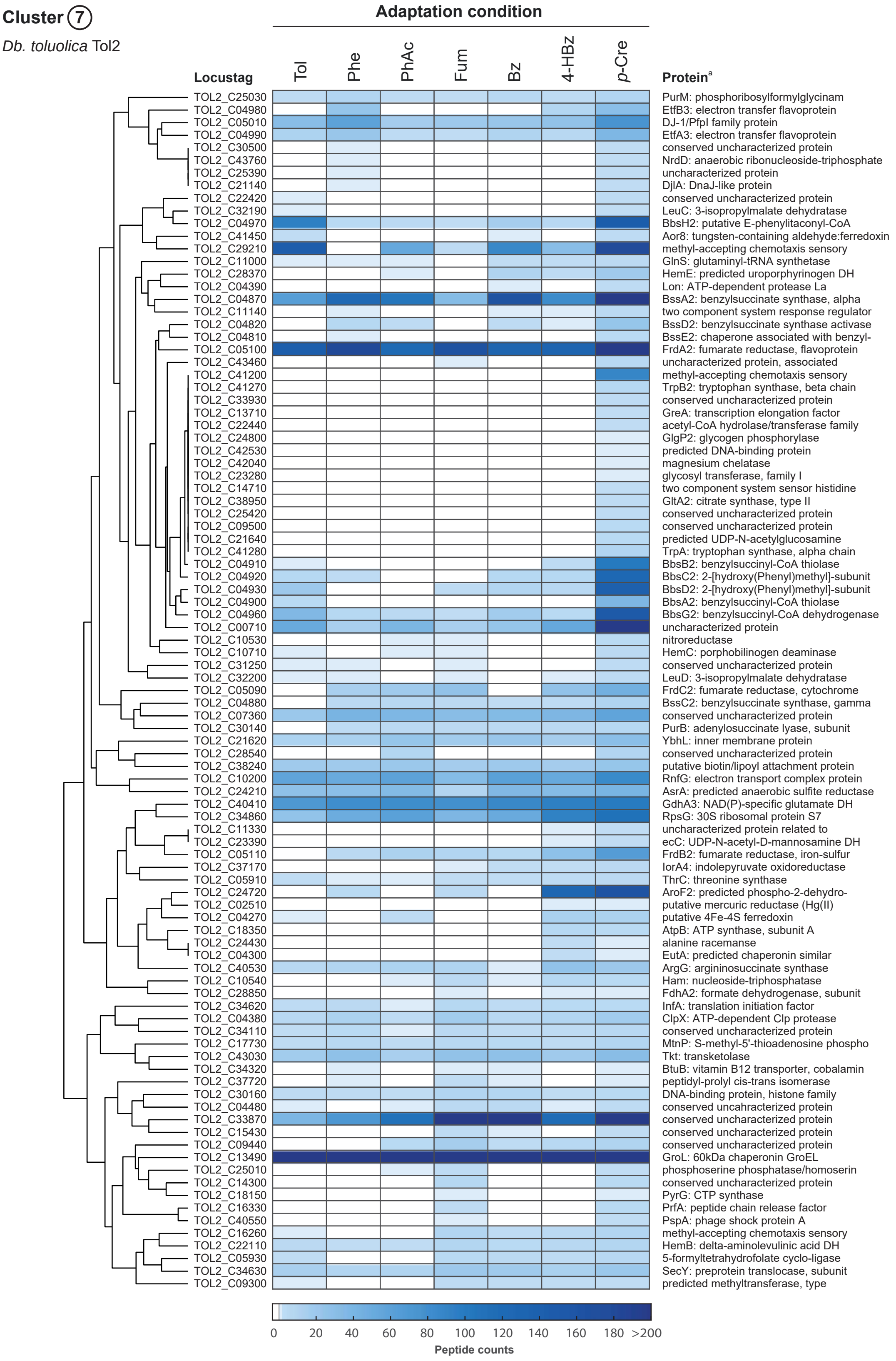

Fig. S109: Detailed view on abundances of proteins forming a sub-cluster in the global clustering depicted in Fig. S10. The global clustering is based on standardized protein abundances of all detected proteins of *Db. toluolica* Tol2 across the 7 studied substrate adaptation conditions.

[a] Full description of protein functions are available in the genome annotation under accession FO203503.

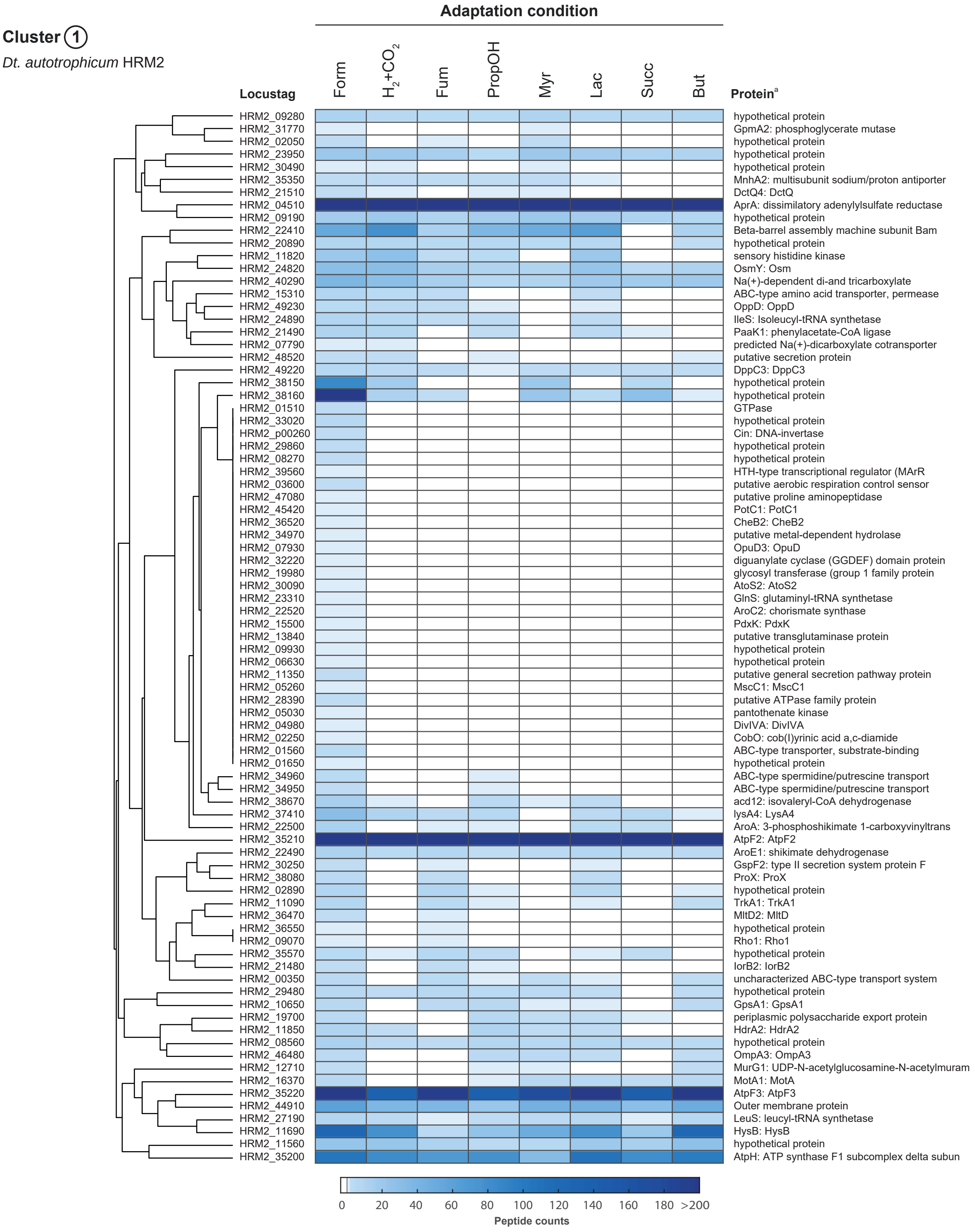

Fig. S110: Detailed view on abundances of proteins forming a sub-cluster in the global clustering depicted in Fig. S11. The global clustering is based on standardized protein abundances of all detected proteins of *Dt. autotrophicum* HRM2 across the 8 studied substrate adaptation conditions.

[a] Full description of protein functions are available in the genome annotation under accession CP001087.

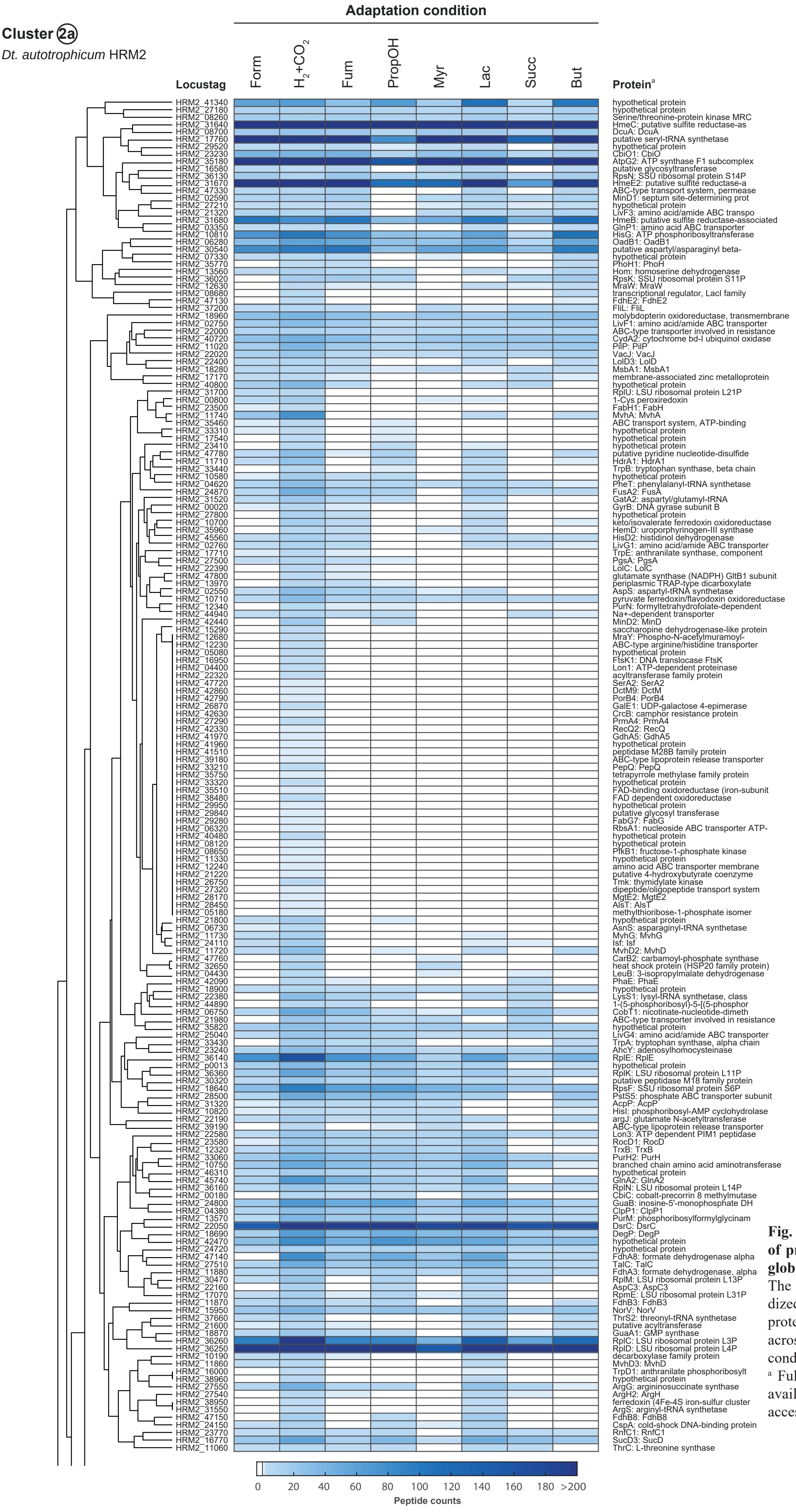

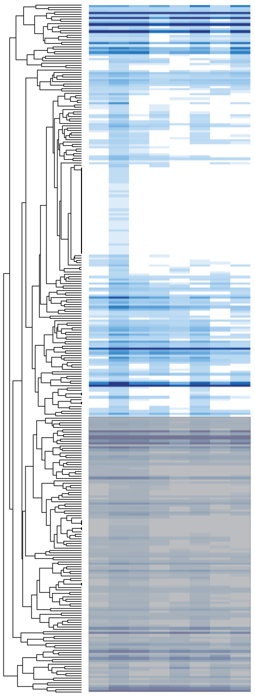

Fig. S111: Detailed view on abundances of proteins forming a sub-cluster in the global clustering depicted in Fig. S11. The global clustering is based on standardized protein abundances of all detected proteins of *Dt. autotrophicum* HRM2 across the 8 studied substrate adaptation conditions.

[a] Full description of protein functions are available in the genome annotation under accession CP001087.

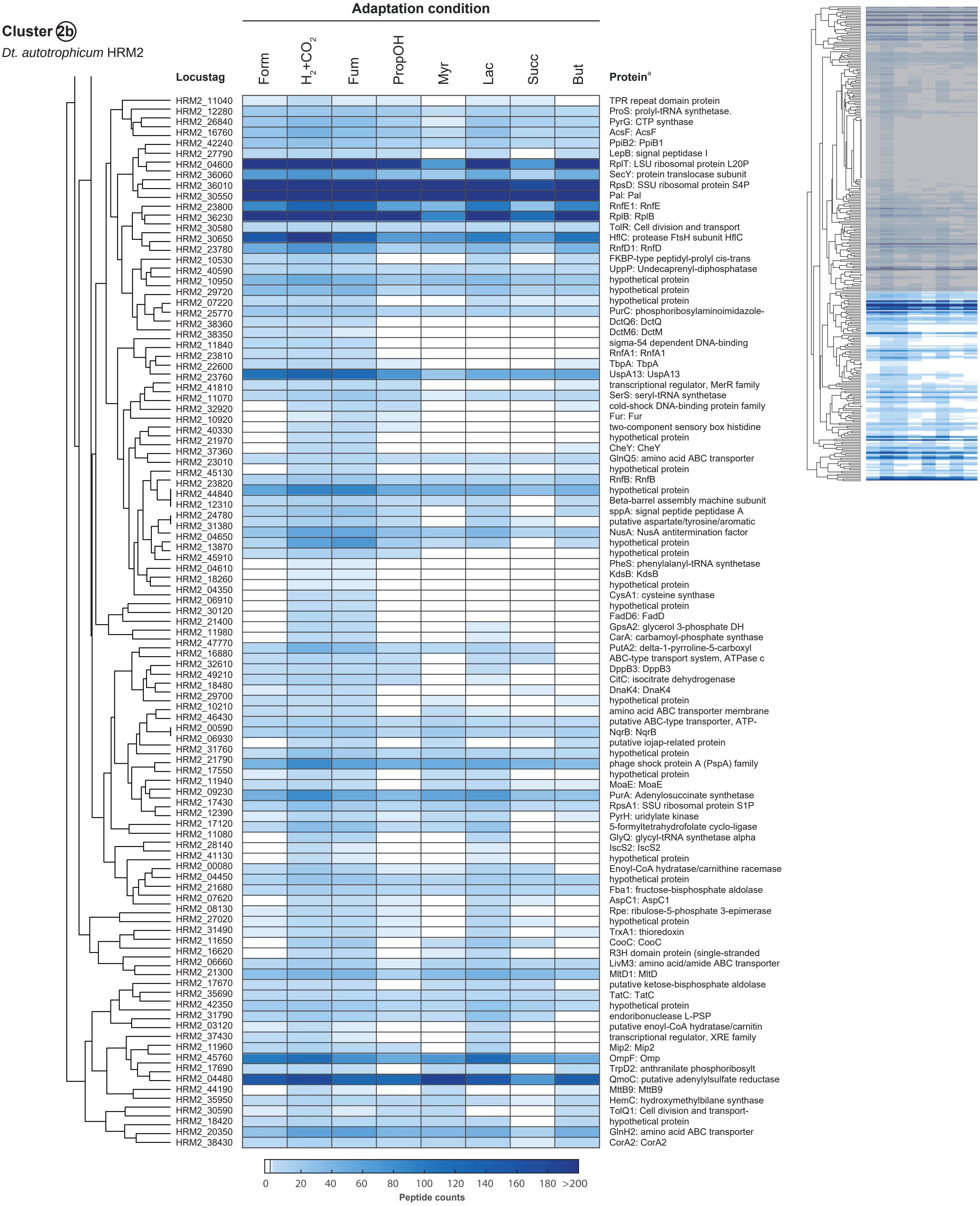

Fig. S112: Detailed view on abundances of proteins forming a sub-cluster in the global clustering depicted in Fig. S11. The global clustering is based on standardized protein abundances of all detected proteins of *Dt. autotrophicum* HRM2 across the 8 studied substrate adaptation conditions.
[a] Full description of protein functions are available in the genome annotation under accession CP001087.

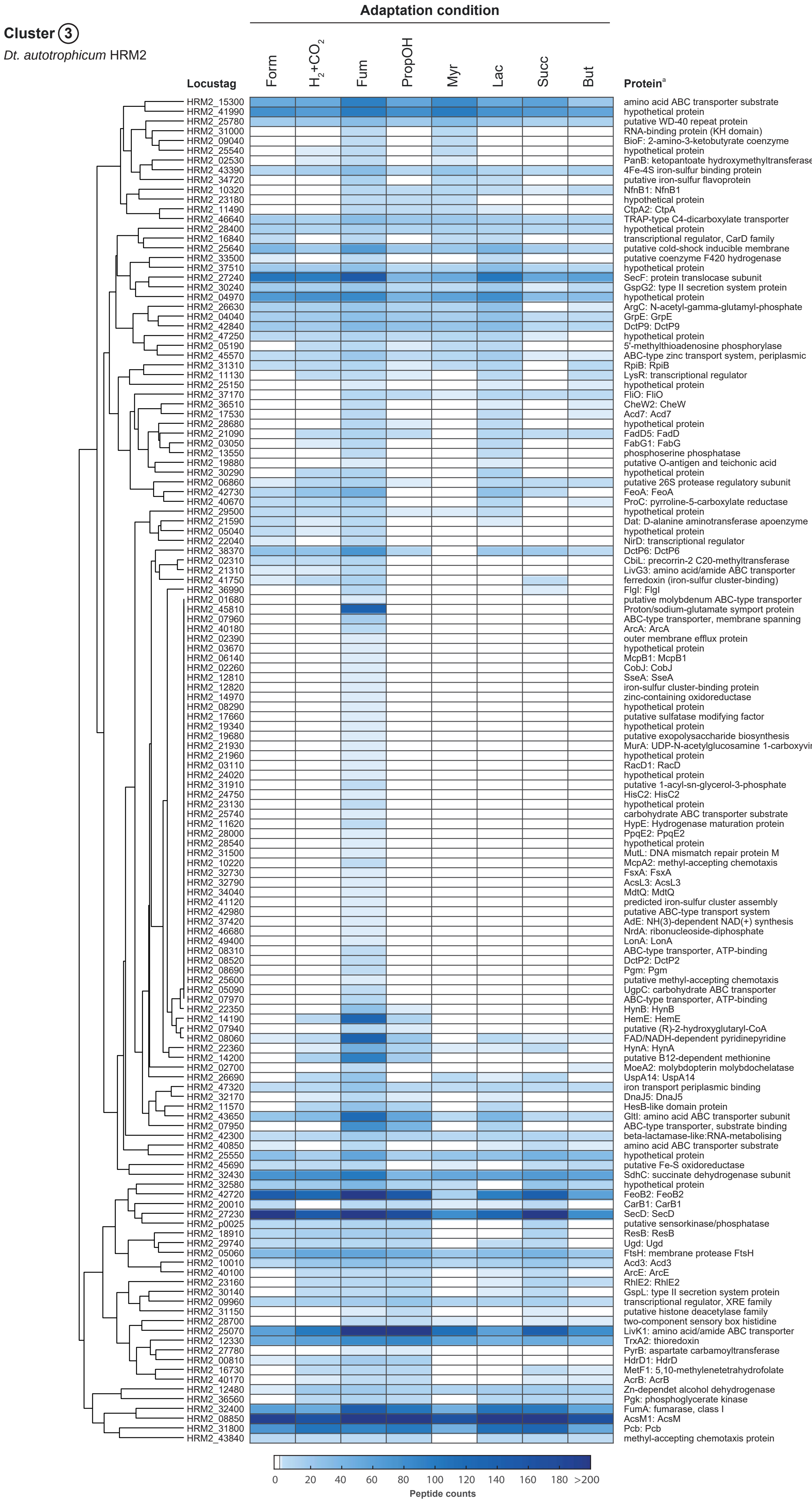

Fig. S113: Detailed view on abundances of proteins forming a sub-cluster in the global clustering depicted in Fig. S11. The global clustering is based on standardized protein abundances of all detected proteins of *Dt. autotrophicum* HRM2 across the 8 studied substrate adaptation conditions.

[a] Full description of protein functions are available in the genome annotation under accession on CP001087.

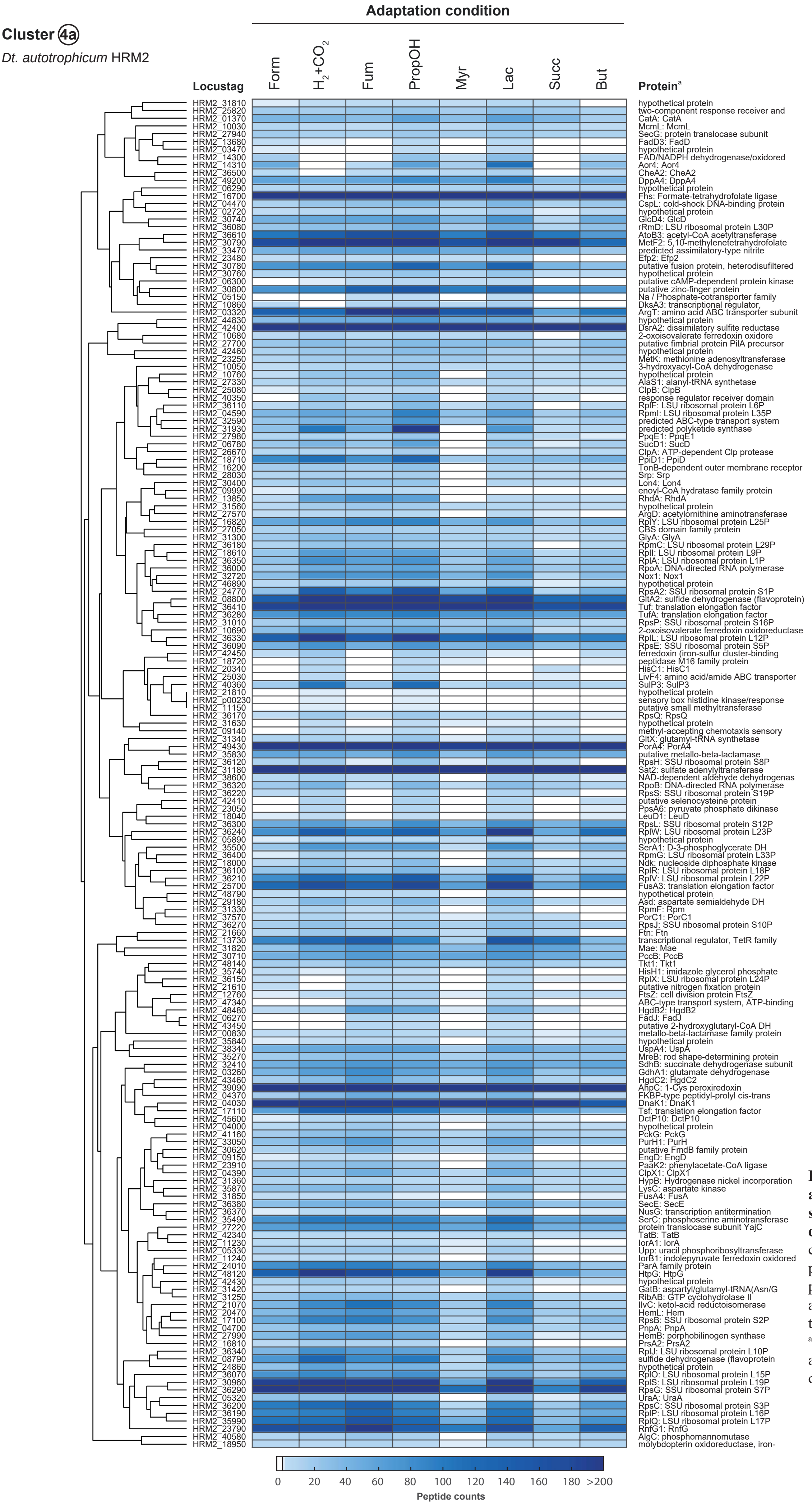

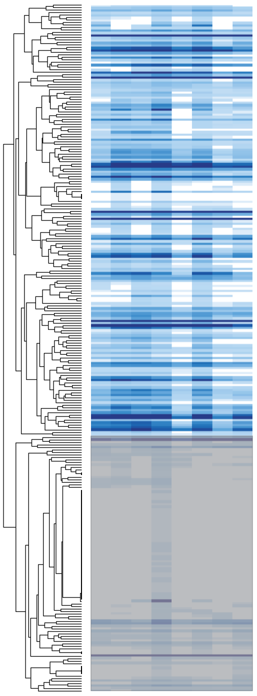

Fig. S114: Detailed view on abundances of proteins forming a sub-cluster in the global clustering depicted in Fig. S11. The global clustering is based on standardized protein abundances of all detected proteins of *Dt. autotrophicum* HRM2 across the 8 studied substrate adaptation conditions.

[a] Full description of protein functions are available in the genome annotation under accession CP001087.

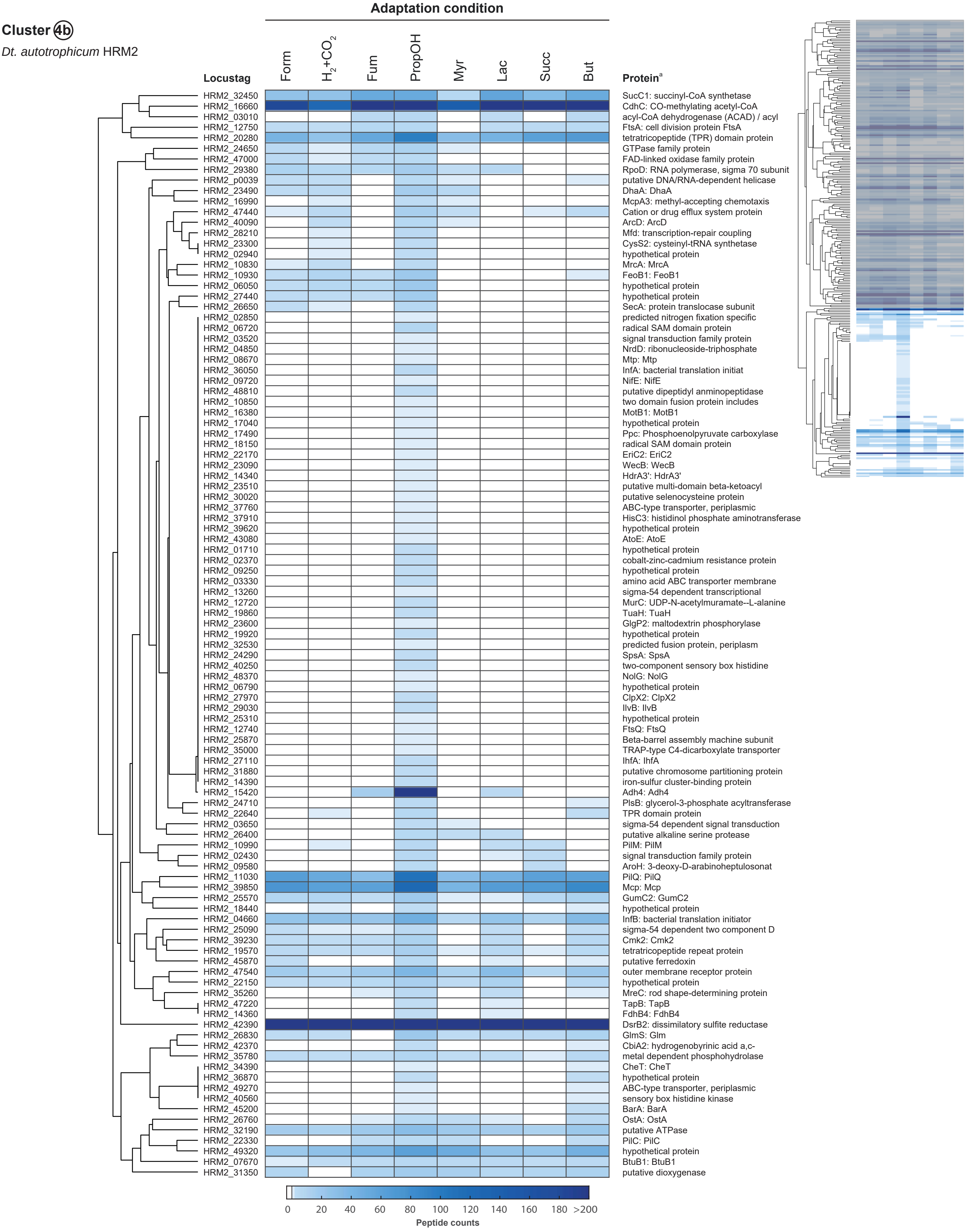

Fig. S115: Detailed view on abundances of proteins forming a sub-cluster in the global clustering depicted in Fig. S11. The global clustering is based on standardized protein abundances of all detected proteins of *Dt. autotrophicum* HRM2 across the 8 studied substrate adaptation conditions.

[a] Full description of protein functions are available in the genome annotation under accession CP001087.

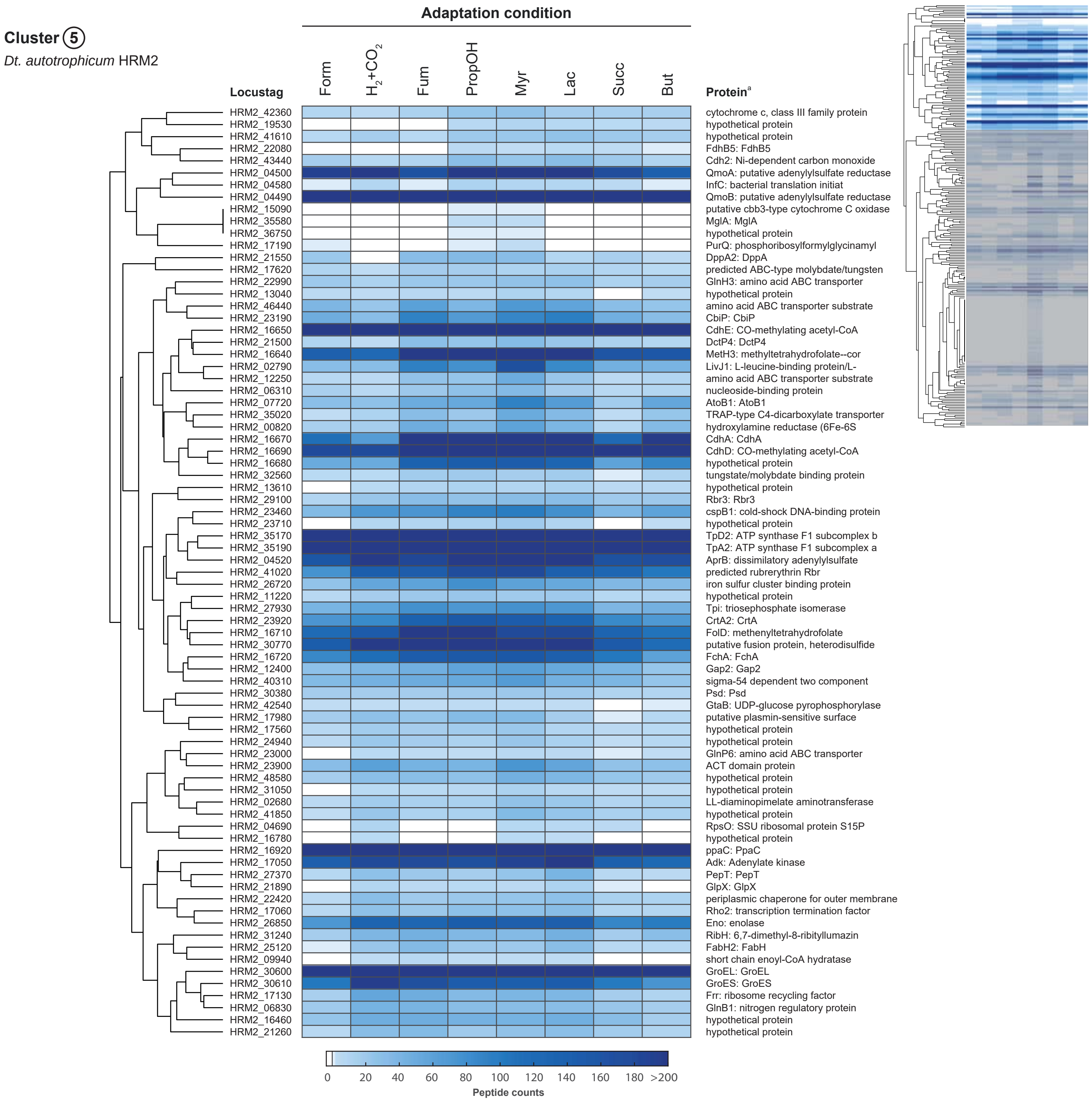

Fig. S116: Detailed view on abundances of proteins forming a sub-cluster in the global clustering depicted in Fig. S11. The global clustering is based on standardized protein abundances of all detected proteins of *Dt. autotrophicum* HRM2 across the 8 studied substrate adaptation conditions.

[a] Full description of protein functions are available in the genome annotation under accession CP001087.

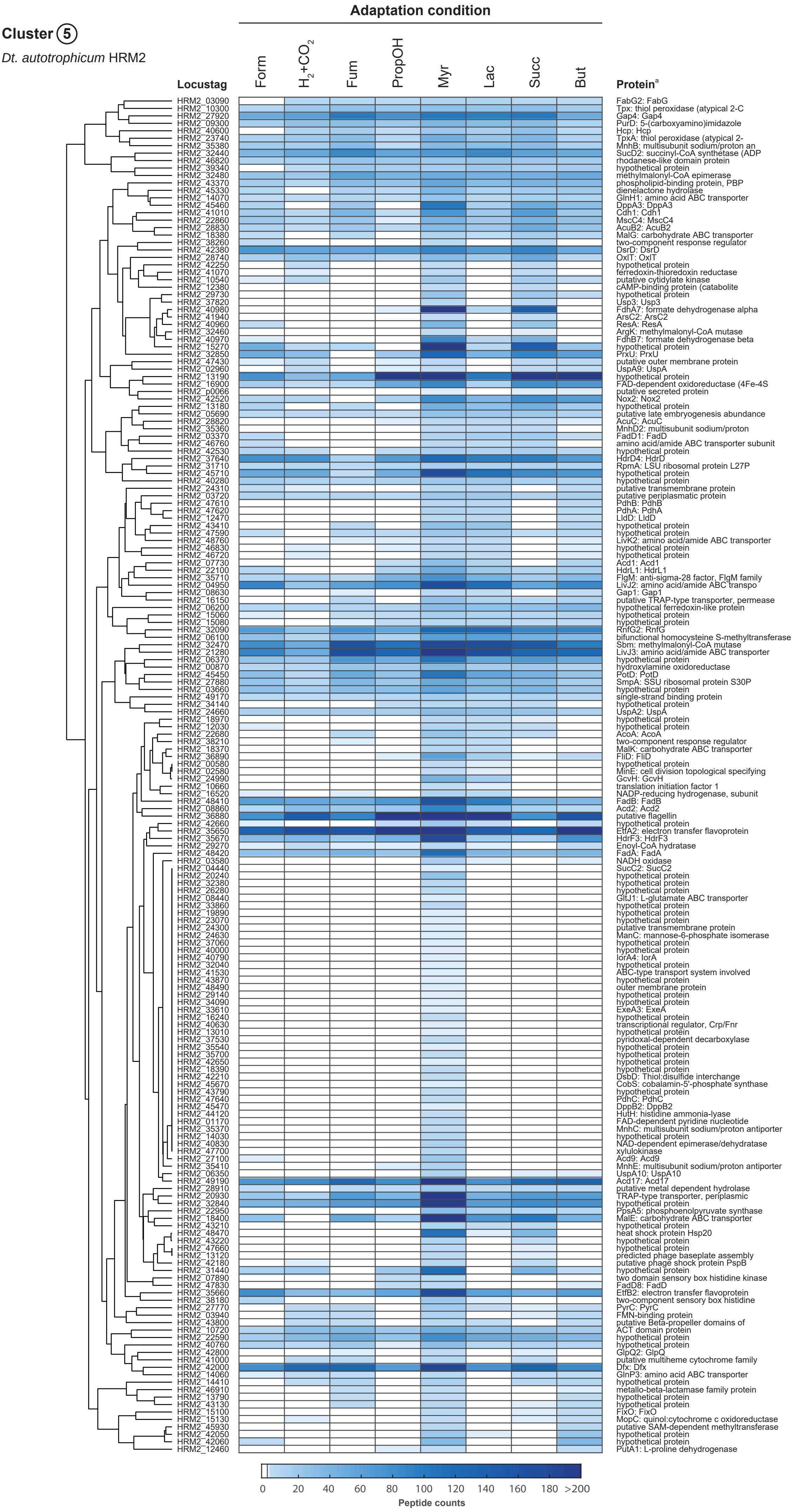

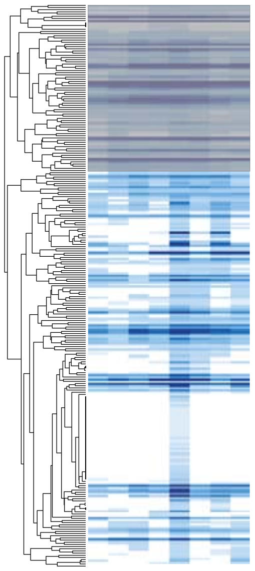

Fig. S117: Detailed view on abundances of proteins forming a sub-cluster in the global clustering depicted in Fig. S11. The global clustering is based on standardized protein abundances of all detected proteins of *Dt. autotrophicum* HRM2 across the 8 studied substrate adaptation conditions.

[a] Full description of protein functions are available in the genome annotation under accession CP001087..

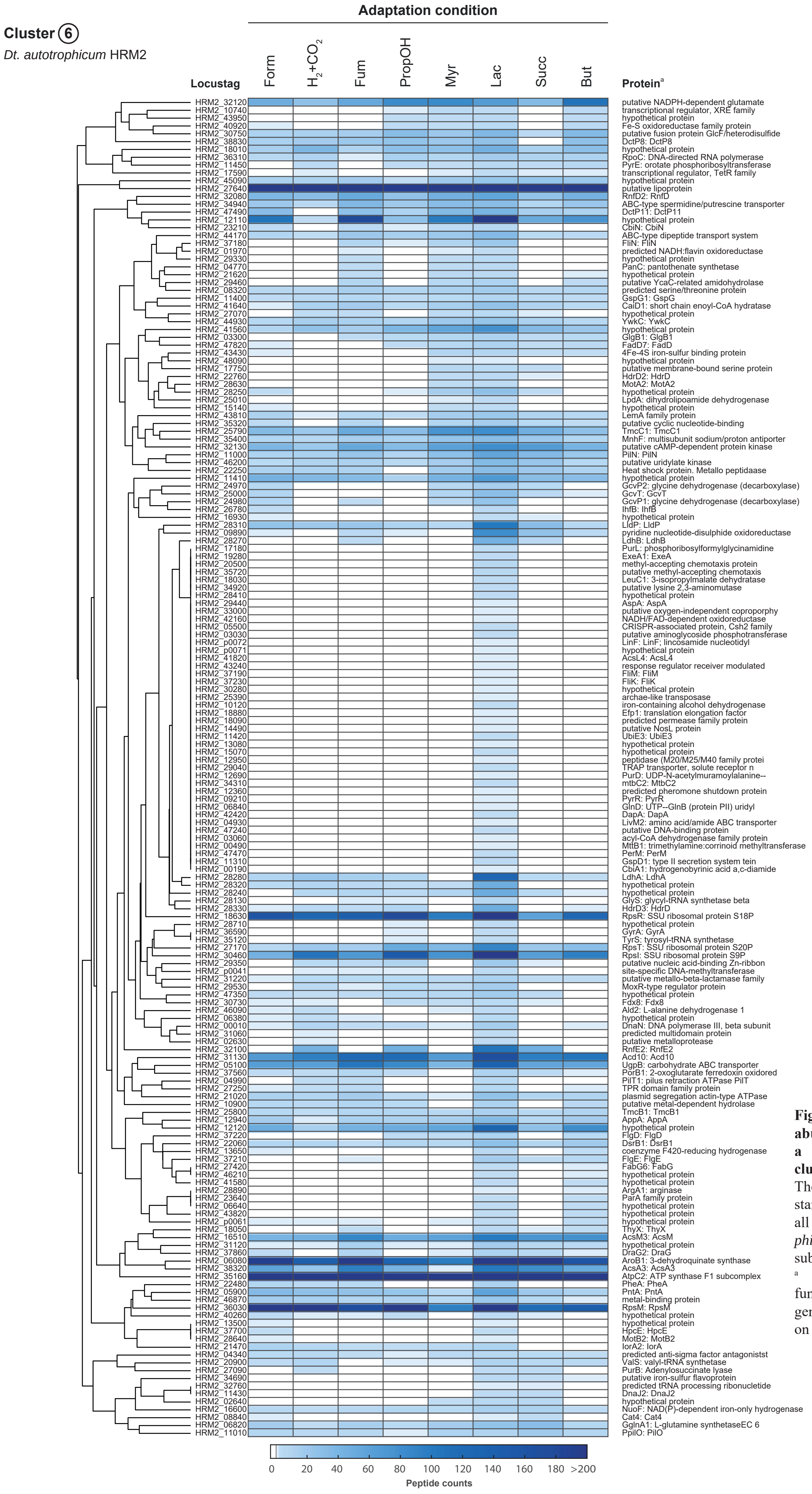

Fig. S118: Detailed view on abundances of proteins forming a sub-cluster in the global clustering depicted in Fig. S11. The global clustering is based on standardized protein abundances of all detected proteins of *Dt. autotrophicum* HRM2 across the 8 studied substrate adaptation conditions.

[a] Full description of protein functions are available in the genome annotation under accession CP001087.

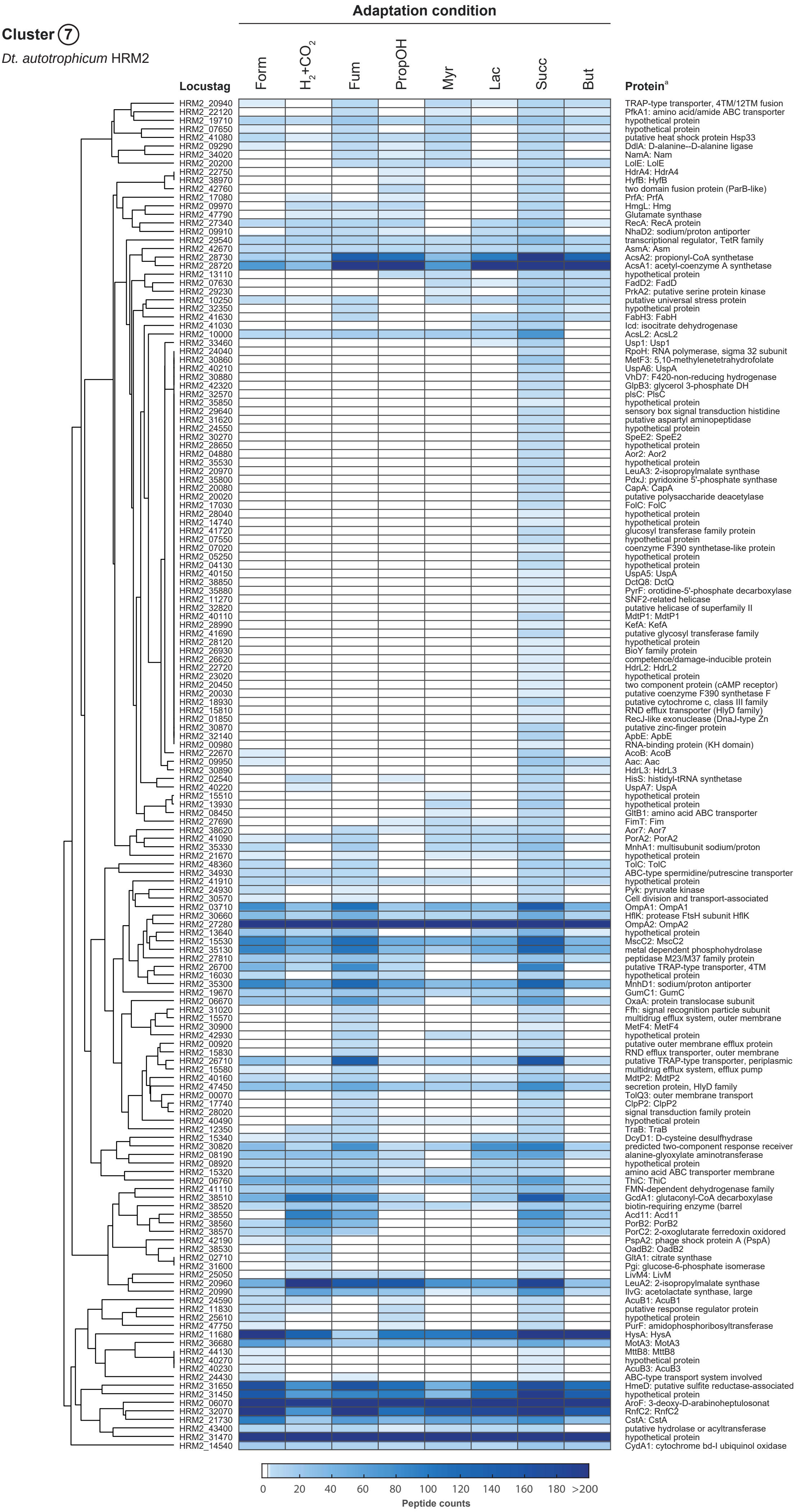

Fig. S119: Detailed view on abundances of proteins forming a sub-cluster in the global clustering depicted in Fig. S11. The global clustering is based on standardized protein abundances of all detected proteins of *Dt. autotrophicum* HRM2 across the 8 studied substrate adaptation conditions.

[a] Full description of protein functions are available in the genome annotation under accession CP001087.

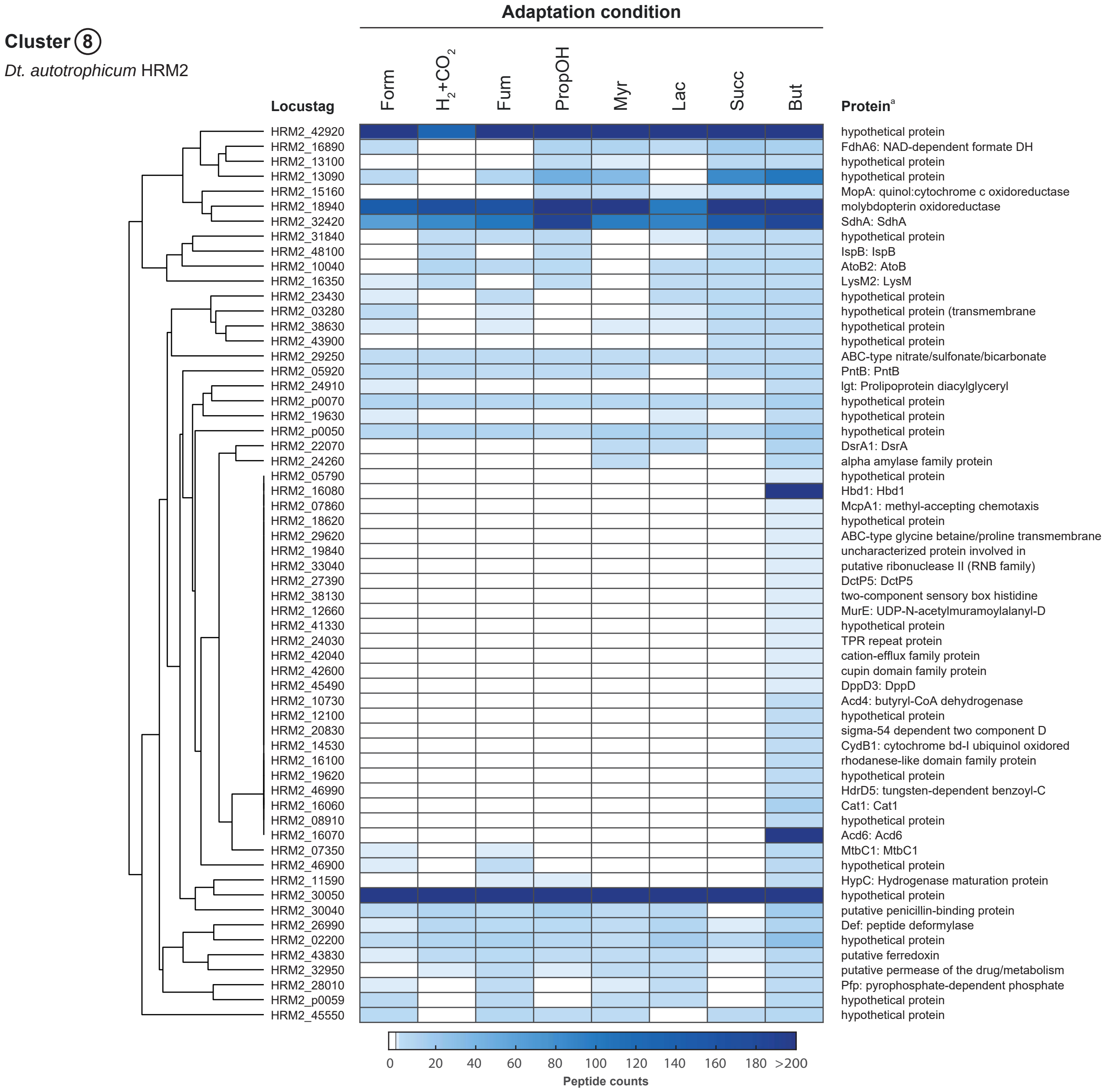

Fig. S120: Detailed view on abundances of proteins forming a sub-cluster in the global clustering depicted in Fig. S11. The global clustering is based on standardized protein abundances of all detected proteins of *Dt. autotrophicum* HRM2 across the 8 studied substrate adaptation conditions.

[a] Full description of protein functions are available in the genome annotation under accession CP001087.

Supplementary Tables

Table S1 Biogeography, habitats, and diversity of isolated strains belonging to *Desulfosarcina, Desulfococcus*, *Desulfobacula*, *Desulfobacterium* and *Desulfonema*.

**Strain**	**Sample**			**Genome**	
	**Type**	**Location**	**Reference**	**Status**	**Reference**
***Desulfosarcina* (9 isolated strains, 5 Species, 5 Genomes)**					
Ds. widdelii					
PP31	Petroleum contaminated Marine shelf sediment	Shuaiba, Kuwait	(*112*)	complete	(*113*)
Ds. ovata subs. Sediminis					
28bB2T	Marine shelf sediment	Tokyo Bay, Japan	(*114*)	complete	(*113*)
oXyS1	Waterphase of an Oiltank	Wilhelmshaven, Germany	(*115*)	complete	(*113*)
Desulfosarcina sp.					
SD1	Mangrove sediment	Mtoni mangrove forest at Mzinga creek, Dar-es-Salaam, Tanzania	(*116*)		
BuS5	Marine hydrocarbon seep	Guaymas Basin, Gulf of California	(*117*)	complete	(*118*)
CME1	Estuarine sediment	Kysing Fjord, Norsminde, Denmark	(*119*)		
Ds. alkanivorans					
PL12	Petroleum contaminated Marine shelf sediment	Shuaiba, Kuwait	(*120*)	complete	(*113*)
Ds. variabilis					
3be13	Marine shelf sediment	Montpellier, France	(*68*)		
Ds. cetonica					
480	Oil recovery water	Aspheron Peninsula, Aserbaidschan	(*121*)		
***Desulfococcus* (4 isolated strains, 2 Species, 1 Genome)**					
Ds. multivorans					
1be1	Marine shelf sediment	Montpellier, France	(*68*)	complete	(*28*)
Ds. biacutus					
KMRActS	Anaerobic digestor sludge	Marburg, Germany	(*122*)		
Desulfococcus sp.					
Hy5	Marine sediment	Rio marin, venice, Italy	(*123*)		
WHC	Intertidal sediment	Wilhelmshaven, Germany	(*124*)		
Desulfobacula (2 isolated strains, 2 Species, 2 Genomes)					
Db. tuluolica					
Tol2	Marine shelf sediment	Eel Pond, Falmouth, USA	(*125*)	complete	(*27*)
Db. phenolica					
Ph01	Marine sediment	Rio marin, venice, Italy	(*126*)	draft	(*127*)

Table S1 continued

Strain	Sample			Genome	
	Type	Location	Reference	Status	Reference
Desulfobacterium (13 isolated strains, 4 Species, 1 Genome)					
Dt. autotrophicum					
HRM2	Marine sediment	Rio marin, Venice, Italy	(*128*)	complete	(*129*)
Dt. catecholicum					
NZva20	Marine shelf sediment	Delaware Bay, Nelson, New Zealand	(*130*)		
Dt. indolicum					
InO4	Marine sediment	Rio marin, Venice, Italy	(*131*)		
Dt. niacini					
NAV-1	Marine sediment	Rio marin, Venice, Italy	(*132*)		
Dt. sp.					
AK1	Estuarine sediment	New York-New Jersey Harbor estuary, USA	(*133*)		
LSv25	Marine shelf sediment	Hornsund, Svalbard, Norway	(*134*)		
BSv41	Marine shelf sediment	Van Mijenfjord, Svalbard, Norway	(*135*)		
mAB1	Saltmarsh/Marine sediment	Étang du Prévost	(*135*)		
W3A	Marine shelf sediment	Chesapeake Bay, Virginia, USA	(*136*)		
X2	Marine shelf sediment	Chesapeake Bay, Virginia, USA	(*136*)		
Cat2	Marine shelf sediment	North Sea coast, Germany	(*123*)		
HRM4	Marine sediment	Rio marin, Venice, Italy	(*128*)		
HRM6	Marine sediment	Rio marin, Venice, Italy	(*128*)		
Desulfonema (4 isolated strains, 3 Species, 1 Genome)					
Dn. ishimotonii					
Tokyo 01	Marine shelf sediment	Tokyo Bay, Japan	(*137*)	complete	(*138*)
Jade 02	Intertidal sediment	Jadebusen, Germany	(*137*)		
Dn. limicola					
5ac10	Intertidal sediment	Jadebusen, Germany	(*139, 140*)		(*29*)
Dn. magnum					
4be13	Marine shelf sediment	Étang du Prévost	(*139, 140*)		(*29*)

Table S2 Biogeography and habitats of phylotypes affiliating with the *Desulfococcus/Desulfosarcina* cluster.

Sample type	Method	Sample location	Reference
Estuarine sediment	16S-rRNA and *dsr*B sequencing	Yangtze Estuary, China	(*141*)
Marine shelf sediment	16S-rRNA gene amplicon sequencing	Cape Lookout Bight, North Carolina, USA	(*142*)
Settling ponds and wetland sediment	16S-rRNA sequencing	Pennsylvania, USA	(*143*)
Seeding sludge	16S-rRNA sequencing	Hongkong, China	(*144*)
Marine shelf sediment	16S-rRNA sequencing	Jansand, Germany; Courseulles-sur-Mer, Mont St. Michel, France	(*145*)
Estuarine to marin bay waters	16S-rRNA and *dsr*AB sequencing	Adour estuary, France	(*146*)
Saltmarsh sediment	16S-rRNA and *dsr*B sequencing	Yancheng Nature Reserve, China	(*147*)
Wetland	16S-rRNA sequencing	North Dakota, USA	(*148*)
Marine shelf sediment	16S-rRNA sequencing	Ría de Vigo, Spain	(*149*)
Saline lake reservoir	16S-rRNA, FISH, PLFA	Lake Grevelingen, The Netherlands	(*150*)
Hypersaline lake/marine shelf sediment	16S-rRNA sequencing	Great salt lake, Utah, USA	(*151*)
Coid grain surfaces of marine and Terrestrial spots	16S-rRNA sequencing	Pigeon Cay, The Bahamas	(*152*)
Sublake/ lake/ freshwater reservoir	16S-rRNA sequencing, EL-FAME	Maojiabu and Xilihu, Hangzhou, China	(*153*)
Uranium contaminated aquifer	16S-rRNA and *dsr*A sequencing	Oak ridge, Tennessee, USA	(*154*)
Marine shelf sediment	AAMG	Rabigh coast, Saudi-Arabia	(*155*)
Meromictic lake	16S-rRNA sequencing	Lake Cadagno, Swiss Alps	(*156*)
Marine shelf sediment	16S-cDNA sequencing	Mira channel, Portugal	(*157*)
Rhizospheres from seagrasses	16S-rRNA gene amplicon sequencing	Culatra Island, Faro, Portugal	(*158*)
Methane seep/deep sea sediment	16S-rRNA and *apr*A sequencing, FISH-NanoSIMS	Southern ridge, California, USA; Mound 12, Costa rica	(*159*)
Methane seep/deep sea sediment	16S-rRNA, FISH/CARD-FISH, BONCAT	Santa monica Basin, California, USA; Hydrate ridge, Gulf of Mexico	(*160*)
Saline lake reservoir	16S-rRNA and *apr*A sequencing	Lake Grevelingen, The Netherlands	(*161*)
Subtropical freshwater reservoirs	16S-rRNA pyro-sequencing	Queensland, Australia	(*162*)
Deep sea sediment; shelf sediment	16S-rRNA pyro-sequencing	Skagerrak; Bothnian bay, Baltic/north sea	(*163*)
Marsh/estuarine sediment	*dsr*AB, RFLP	Shanyutan, China	(*164*)
Mangrove wetland	16S-rRNA sequencing	Jimei district, Xiamen City, China	(*165*)
Deep sea sediment	16S-rRNA sequencing	Gulf of Mexico	(*166*)
Hydrothermal sediment	16S-rRNA, CARD-FISH	Guaymas Basin, Gulf of California, Mexico	(*167*)
Oil contaminated desert soils	16S-rRNA, ARISA	north of Oman	(*168*)
Crude oil contaminated marsh	Metagenomic analysis	Bay Jimmy, Louisiana, USA	(*169*)
Mangrove soil	*dsr*B, DGGE	Red sea coast, Jeddah, Saudi-Arabia	(*170*)
Deep sea sediment/cold seep	16S-rRNA, ARISA	Sonora Margin, Guaymas Basin, Gulf of California, Mexico	(*171*)
Landfill	16S-rRNA sequencing	Jinqianpu landfill, China	(*172*)
Salt marh sediment	16S-rRNA sequencing	Bayou La Batre, Alabama, USA	(*173*)
Marine watercolumn	16S-rRNA, *dsr*B, DGGE	Gdansk Bay, Baltic Sea	(*174*)
Intertidal mudflat	16S rRNA gene-Illumina Miseq sequencing	Sansha bay, Fuan, China	(*175*)
Marine sheld sediment	16S-rRNA, *dsr*B sequencing, SIP	Aarhus Bugt, Denmark	(*176*)
Hydrothermal vent system	16S-rRNA, *dsr*B, FISH, CSLM	Central Bay, Prony, New Caledonia	(*177*)
Coastal seawater	16S-rRNA sequencing	Key West, Florida, USA	(*178*)
Intertidal mangrove zone	16S-rRNA sequencing	Lim Chu Kang, Singapore	(*179*)
Landfill	16S-rRNA gene pyrosequencing	Laohukeng, ShenZhen, China	(*180*)
Marine shelf sediment	16S-rRNA gene pyrosequencing	Busan New Port, North Port, South Korea	(*181*)
Rice paddy soils	16S-rRNA sequencing	Guiyang city, China	(*182*)
Oil Sands Composite Tailings Deposit	16S-rRNA sequencing, PFLA	Kingfisher CT deposit, Canada	(*183*)
Coastal microbial mat	16S-rRNA sequencing	Shark bay, Australia	(*184*)
Petroleum-hydrocarbon-contaminated Marine sediment	16S-rRNA sequencing	Hong-Kong, China	(*185*)
Petroleum reservoir	16S-rRNA, *dsr*B, FLASH	Xinjiang Luliang Oil Field, China	(*186*)
Hydrothermal, metalliferous Marine sediments	masD/assA amino sequences	Chowder Hill, Juan de Fuca Ridge	(*187*)
Salt marsh sediment	16S-rRNA, T-RFLP, SIP	Talbert salt marsh, California, USA	(*188*)
Sea cucumber gut	16S-rRNA gene pyrosequencing	Quingdao, China	(*189*)
Coastal wetland	16S-rRNA sequencing	Magnolia/Talbert marsh, California, USA	(*190*)
Eutrophic, meromictic lake	16S-rRNA/*apr*A, CARD-FISH	Lake Harutori, Hokkaido, Japan	(*191*)
Deep sea sediment	16S-rRNA, TEFAP	Eastern Mediterranean Levantine Basin	(*192*)
Boreal acid sulphate soil	CARD-FISH, EL/NEL-PLFA	AS soil near Helsinki, Finland	(*193*)

Table S2 continued

Sample type	Method	Sample location	Reference
Cold seep/deep sea sediment	16S-rDNA, FISH	Sonora Margin, Guaymas Basin, Gulf of California, Mexico	(*171*)
Saline solis from barren land	16S-rRNA, *aps*A sequencing	Arabian Sea coast, Gujarat, India	(*194*)
Marine shelf sediment/deepsurface fluid	SSU rRNA sequencing	Juan de Fuca Ridge	(*195*)
Seep sediment/deep sea sediment	FISH-nanoSIMS	Mound 12, Costa Rica	(*196*)
Seep sediment/deep sea sediment	CARD-FISH, SIP	Amon mud volcano, Nile deep-sea in the mediterranean sea	(*197*)
Pond in a pyrite mine	16S-rRNA sequencing	Rudawy Janowickie Mountains, Poland	(*198*)
Marine shelf sediment	SSU-rRNA sequencing and lipid sequence via HPLC, FLEC	Highborne Cay, The Bahamas	(*199*)
Marine shelf sediment	16S-rRNA pyrosequencing	Port of Aveiro, Atlantic coast, Portugal	(*200*)
Seep sediment/deep sea sediment	16S-rRNA, nanoSIMS	Hydrate ridge, Gulf of Mexico	(*201*)
Estuarine sediment	16S-rRNA, DGGE	Colne estuary, Essex, UK	(*202*)
Natural corrosion deposits from marine Steel structures	16S-rRNA, *dsr*B, DGGE	carbon steel sheet piles of a European harbor	(*203*)
Marine oil fields	*dsr*B, DHPLC, DGGE	MOB11, Brazil; MOB12A/B, USA	(*204*)
Deep-sea methane seeps	16S-rRNA, ARISA, CARD-FISH	Wairarapa Takahae, New Zealand	(*205*)
Mesotrophic and oligotrophic lake	*apr*A sequencing	Lake Biwa; Lake Okotanpe, Japan	(*206*)
Hydrothermal sediment	16S-rRNA, CARD-FISH	Yonaguni Knoll IV, southern Okinawa Trough	(*24*)
Seep sediment/deep sea sediment	16S-rRNA, GC-MS	Cinarcik Basin, Marmara Sea	(*207*)
Estuarine microbial mat	SSU-rRNA sequencing	Elkhorn Slough estuary, California, USA	(*208*)
Tidal flat area	16S-rRNA, DGGE	Spiekeroog Island, Germany	(*209*)
Hydrocarbon seep/deep sea sediment	16S-rRNA, CARD-FISH	Gulf of mexico	(*210*)
River water/sediment	16S-rRNA sequencing	Taif River, Taif, Saudi Arabia	(*211*)
Deep sea mud volcano	CARD-FISH	Isis mud volcano, Mediterranean Sea	(*212*)
Paddy field	16S-rRNA sequencing	Fukutsu, Japan	(*213*)
Intertidal mudflat, mangrove sediment	16S-rRNA sequencing	Mai Po Ramsar wetland, Hong-Kong	(*214*)
Hydrothermal deep sea sediment	16S-rRNA, FISH	Chowder Hill hydrothermal vent field, Juan de Fuca ridge	(*215*)
Hg enriched river sediment	16S-rRNA sequencing	South River, Virginia, USA	(*216*)
Marine shelf sediment	16S-rRNA, DGGE	Porto Marghera, Venice, Italy	(*217*)
River/estuarine sediment	*dsr*B, DGGE	Danshuei River estuary, Taiwan	(*218*)
Cold mineral spring	16S-rRNA, *dsr*B, oligonucleotide hybridization	Ust'Kachka resort, Russia	(*219*)
Polluted microbial mat	16S-rRNA, slot-blot hybridization	Wadi Gaza, Gaza Strip, Palestine	(*220*)
Marine microbial mat	16S-rRNA sequencing	Island Schiermonnikoog, The Netherlands	(*221*)
Submarine spring	16S-rRNA sequencing	Jewfish Sink, Gulf of Mexico	(*222*)
Seep sediment/deep sea sediment	16S-rRNA, CARD-FISH	Nile Deep Sea Fan, Mediterranean Sea	(*223*)
Coal oil point seep	*dsr*AB sequencing	Tonya Seep, California, USA	(*224*)
Deep sea sediment	16S-rRNA, DGGE	Nyegga G11 pockmark, Offcoast Norway	(*225*)
Haloalkaline lake	16S-rRNA, ARDRA	Lake Elmenteita, Kenya	(*226*)
Hydrothermal deep sea sediment	16S-rRNA, CARD-FISH	Logatchev hydrothermal field, north Atlantic Ocean	(*227*)
Meromictic lake	16S-rRNA, CARD-FISH	Lake Cadagno, Alps, Switzerland	(*228*)
Methane seep/deep sea sediment	16S-rRNA, DGGE, CARD-FISH	Basin off Sumatra, Indian Ocean	(*229*)
Deep sea sediment	*dsr*A, *apr*A sequencing	Black Sea	(*230*)
Marine oxygen-minimum-zone	16S-rRNA, *dsr*, *apr* sequencing	Off the Chilean Coast	(*5*)
Shallow marine hydrothermal sediment	16S-rRNA, *dsr*AB, RFLP	Nea Kameni Island, Greece	(*231*)
Spring water	16S-rRNA sequencing	Zodletone Spring, Oklahoma, USA	(*232*)
Deep sea/methane seep sediment	16S-rRNA, FISH/CARD-FISH	Hydrate ridge, Gulf of Mexico	(*233*)
Marine shelf sediment	16S-rDNA, DGGE	Emerald Basin, Halifax Harbour, Canada	(*234*)
Intertidal sediment	16S-rRNA, *dsr*AB, *apr*A, FISH	Wadden Sea, Germany	(*235*)
Marine shelf sediment	16S-rRNA, FISH	Cabreara national Park, Balearic Islands	(*236*)
Deep sea sediment	16S-rRNA, *dsr*AB, FISH	Santa barbara Basin, California, USA	(*237*)
Hydrocarbon polluted estuarine sediment	16s-rRNA, dsrAB sequencing, PLFA	Pearl river Estuary, China	(*238*)
Hypersaline brine pool/deep sea	16S-rRNA, *dsr*AB, sequencing	Gulf of Mexico	(*239*)
Cold seep/deep sea sediment	16S-rRNA sequencing	Larsen B area, Weddell, Antarktika	(*240*)
Cave sediment	16S-rRNA, DGGE	Altamira cave, Spain	(*241*)
Anoxic tidal fjord	SSU-rRNA, RFLP	Nitinat Lake, British Columbia, Canada	(*242*)
Estuarine intertidal flat	16S-rRNA, Mag-SIP	Oosterschelde Estuary, The Netherlands	(*243*)
Deep sea sediment	16S-rRNA, *dsr*AB, DGGE	Green Canyon Region, Gulf of Mexico	(*244*)

Table S2 continued

Sample type	Method	Sample location	Reference
Petroleum polluted marine sediment	16S-rRNA, *dsr*AB sequencing	Boston harbor, Massachusetts, USA	(*245*)
Deep sea sediment	16S-rRNA sequencing	Mid-Chilean Margin, Chile	(*246*)
Riversediment	16S-rRNA, T-RFLP	River Lahn, Marburg, Germany	(*247*)
Marine shelf sediment	16S-rRNA, probe hybridization	Étang de Berre, France	(*248*)
Estuarine sediment	16S-rRNA, T-RFLP	Hayle estuary, Fal estuary, Kingsbridge estuary, Cornwall and Devon, South West England, UK	(*249*)
Deep sea microbial mat/sediment	16S-rRNA, FISH/CARD-FISH	Nile Deep Sea fan, Mediterranean Sea	(*250*)
Shelf sediment/microbial mat	16S-rRNA, CARD-FISH, FAME	Gullfaks, Tommeliten, North Sea	(*251*)
Deep sea microbial mat/sediment	16S-rRNA, CARD-FISH	GHOSTDABS Field, Black Sea	(*252*)
Riversediment	16S-rRNA, *dsr*B, DGGE, PFLA	Meuse, Rhine, Overijsselse Vech, The Netherland	(*253*)
Cold seep/deep sea sediment	16S-rRNA, CARD-FISH	Gulf of Mexico	(*254*)
Marine oligochaetes	16S-rRNA, *dsr*AB, *apr*A, FISH	Island of Elba, Mediterranean Sea, Italy	(*255*)
Lakesediment	16S-rRNA, FISH	Lake Aha, China	(*256*)
Hypersaline pond	16S-rRNA, nanoSIMS, CARD-FISH	Exportadora De Sal, Baja California Sur, Mexico	(*257*)
Marine shelf sediment	16S-rRNA, DGGE	Paletta Creek, California, USA	(*258*)
Cold seep/deep sea sediment	16S-rRNA sequencing	Kazan mud volcano, Mediterranean Sea	(*259*)
Extremely hypersaline lake sediment	16S-rRNA, *dsr*AB, RFLP	Great salt lake, Utah, USA	(*260*)
Cold seep/deep sea sediment	16S-rRNA, FISH	Mosby Mud Volcano, Norwegian-Barents-Spitzbergen continental margin	(*261*)
Estuarine/riversediment	*dsr*A sequencing	River Colne Estuary, Essex, UK	(*262*)
Microbial mat, calcified deep sea reef	16S-rRNA, CARD-FISH	Crimean Peninsula, Black Sea	(*263*)
Deep sea sediment	16S-rRNA, T-RFLP, DGGE, PLFA	Skagerrak, Denmark	(*264*)
Deep sea sediment	16S-rRNA, DGGE	Benguela Upwelling System, Namibia	(*265*)
Saltmarsh	16S-rRNA, FISH	Little Sippewissett salt marsh, Massachusetts, USA	(*266*)
Hydrocarbon polluted shelf sediment	16S-rRNA, RISA	Canal viell cove, France	(*267*)
Anoxic watercolumn	16S-rRNA, *dsr*A sequencing	Western central Basin, Black Sea	(*268*)
Intertidal soil and intertidal mudflat	16S-rRNA, dsrAB, DGGE	River Rhine, Rozenburg, The Netherlands	(*269*)
Meromictic lake sediment/water	*dsr*A sequencing	Lake Suigetsu, Fukui, Japan	(*270*)
Mud volcano, deep sea sediment	16S-rRNA, RFLP	Northern GoM Slope, Gulf of Mexico	(*271*)
Intertidal sediment	16S-rRNA, FISH, ARDRA	Wadden Sea, south of Sylt, North Sea	(*272*)
Riverwater/sediment	FISH	Dresden-Ubigau, Coswig, Hitzacker, River Elbe, Germany	(*273*)
Shelf sediment/deep sea sediment	16S-rRNA, FISH	Black Sea	(*274*)
Deepsea carbonate crust	16S-rRNA sequencing	Napoli mud Volcano, Mediterranean Sea	(*275*)
Meromictic lake	16S-rRNA, FISH	Gek-Gel Lake, Azerbaijan	(*276*)
Meromictic lake	*dsr*A sequencing	Partly saline Lake Suigetsu, Fukui, Japan	(*277*)
Deep sea sediment/microbial mat	16S-rRNA, T-RFLP, HPLC-APCI-MS	Sagami Trough, Japan	(*278*)
Deep sea sediment/microbial mat	16S-rRNA, *dsr*AB sequencing	Green Canyon, Gulf of Mexico	(*279*)
Deep sea sediment and carbonate crust	16S-rDNA, GC-IRMS	Mud Vulcanos, Gulf of Cadiz	(*280*)
Lake sediment	16S-rRNA, *dsr*AB, DGGE	brakish lake Ogawara, Japan	(*281*)
Deep sea sediment/microbial mat	16S-rRNA sequencing, T-RFLP	Offshore Hokkaido Island, Japan Sea	(*282*)
Deep sea sediment and carbonate crust	16S-rRNA, FISH	Dniepr Canyon, Black Sea	(*283*)
Meromictic lake	16S-rRNA, *dsr*AB, *aps*A, RFLP	Hypersaline Mono Lake, California, USA	(*284*)
Intertidal sediment	FISH, FAME	Spiekeroog Island, North Sea, Germany	(*285*)
Gutless oligochaete O. crassitunicatus	16S-rRNA, FISH	Pacific Ocean, Peru Margin	(*286*)
Intertidal sediment	16S-rRNA, FISH, CARD-FISH	Dangast/Jadebay, Wadden Sea, Germany	(*16*)
Deep sea microbial mat	16S-rRNA sequencing	Milano mud Volcano, Mediterranean Sea	(*287*)
Deep sea microbial reef structure	16S-rRNA, FISH, slot-blot hybridization	Black Sea	(*288*)
Marine shelf sediment	FISH	Eckernförde Bay, Baltic Sea, Germany	(*289*)
HM polluted marine shelf sediment	16S-rRNA, DGGE, FISH	Sørfjord, Norway	(*290*)
Saltmarsh	*dsr*AB, DGGE	Plum Island, Massachusetts, USA	(*291*)
Eutrophic lake	FISH, CARD-FISH	Lake Plußsee, Rathjensdorf, Germany	(*292*)
Mesopelagic oxygen minimum zone	16S-rRNA, CARD-FISH	Arabian Sea, 400 km western of India	(*293*)
Eutrophic lake	16S-rRNA sequencing	Lake Kasumigaura, Japan	(*294*)
Floodplain lake	16S-rRNA, dot-blot hybridization	La Granja, Beni River Subbasin, Bolivia	(*295*)
Cold seep/deep sea sediment	16S-rRNA, CARD-FISH	Gulf of Mexico	(*296*)

Table S2 continued

Sample type	Method	Sample location	Reference
Deep sea sediment/microbial mat	16S-rRNA, FISH	Hydrate ridge, Oregon, USA; Cremean area, Black Sea	(*297*)
Seep sediment/deep sea sediment	SSU-rRNA sequencing	Monterey Bay, California, USA	(*298*)
Marine shelf sediment	CARD-FISH, GC-IRMS	Tommeliten Seepage Area, North SEa	(*299*)
deep sea microbial mat/carbonate crust	16S-rRNA, FISH	Lower Crimean Shelf, Black Sea	(*300*)
Methane seep/deep sea sediment	16S-rRNA sequencing	Ryukyu arc, off Ishigaki Island, Japan	(*301*)
Intertidal sediment	16S-rRNA, FISH, CARD-FISH	Janssand, Spiekeroog Island, Germany	(*22*)
Saltmarsh	16S-rRNA sequencing	Plum Island, Massachusetts, USA	(*302*)
Meromictic lake	16S-rDNA, DGGE	Lake Kaiike, Kamikoshiki Island, Japan	(*303*)
Deep sea black smoker chimney	*dsr*AB sequencing	Kairei Field, Indian Ocean	(*304*)
Estuarine intertidal mudflat	16S-rRNA, DNA-DNA-hybridization, DGGE	North Inlet Estuary, South Carolina, USA	(*305*)
Deep sea sediment	16S-rRNA, FISH, FISH-SIMS	Eel River Basin, California, USA	(*306*)
Shelf, deep sea sediment	16S-rRNA, DGGE, PLFA	George V Basin, Mertz Drift, North Mertz Drift, O'Briens Bay, Burton Lake, Vestfold Hills, Eastern Antarctica	(*307*)
Monomictic lake	SSU-rRNA, probe hybridization	Lake Biwa, Japan	(*308*)
Marine shelf sediment	16S-rRNA, DNA-DNA-hybridization	Signy Island, Antarctica	(*309*)
Deep sea sediment	16S-rDNA sequencing	George V Basin, Eastern Antarctica	(*310*)
Cold seep/deep sea sediment	16S-rRNA, T-RFLP	Japan Trench	(*311*)
Intertidal sediment	16S-rRNA, DGGE, PLFA, FISH	Dangast/Jadebay, Wadden Sea, Germany	(*15*)
Hydrothermal deep sea sediment	16S-rRNA sequencing	Guaymas Basin, Gulf of California, Mexico	(*312*)
Marine shelf sediment	16S-rRNA, DGGE	Tokyo Bay, Japan	(*313*)
Cyanobacterial mat, hypersaline lake	16S-rRNA, *dsr*AB, probe hybridization	Solar Lake, Sinai, Egypt	(*314*)
Deep sea sediment/microbial mat	16S-rDNA, FISH	Eel River Basin, Santa Babara Basin, USA	(*315*)
O. algarvensis, gutless oligochaete	16S-rRNA, FISH	Capo di San Andrea, Elba, Italy	(*61*)
Saltmarsh	16S-rRNA, probe hybridization	Savannah, Georgia, USA	(*316*)
Flooded rice roots/wetland sediment	SSU-rRNA, T-RFLP, dot-blot hybridization	Rice Research Institute, Vercelli, Italy	(*317*)
Seep sediment/deep sea sediment	FISH-SIMS	Eel River Basin, Santa Babara Basin, USA	(*318*)
Saltmarsh	16S-rRNA, probe hybridization	Savannah, Georgia, USA	(*319*)
Seep sediment/deep sea sediment	16S-rRNA, FISH	Hydrate ridge, Oregon, USA	(*320*)
Meromictic lakes	16S-rDNA, RFLP	Vestfold Hills, Eastern Antarctica	(*321*)
Marine shelf sediment	16S-rDNA sequencing	d'Arcachon, South-West France	(*322*)
Marine shelf sediment	16S-rRNA, FISH, slot-blot hybridization	Smeerenburgfjorden; Svalbard; Magdalenefjorden; Raudfjorden; Hornsund, Svalbard Arctic Ocean	(*323*)
Saltmarsh	16S-rRNA, slot-blot hybridization	Brunswick, Georgia, USA	(*324*)
Marine shelf sediment	16S-rDNA, dot-blot hybridization, ARDRA	Hornsund, Svalbard, Arctic Ocean	(*325*)
Marine shelf sediment	16S-rRNA, probe hybridization	Baltic Sea/North Sea, Aarhus, Denmark	(*326*)
Post-glacial freshwater sediments	16S-rRNA sequencing	North Basin of Windermere, England	(*327*)
Marine snow aggregates	16S-rRNA, RFLP	Gulf of Trieste, Aurisina, Italy	(*328*)
PCB polluted shelf sediment	16S-rDNA, RFLP	Baltimore Harbor, Maryland, USA	(*329*)
Marine shelf sediment	16S-rRNA, probe hybridization	Santa Rosa Sound, Florida, USA	(*330*)
Seep sediment/deep sea sediment	16S-rRNA, RFLP	Guaymas Basin, Gulf of California, Mexico	(*331*)
Saltmarsh	16S-rDNA, probe hybridization	Chapman's Marsh, New Hampshire, USA	(*332*)
Contaminated shelf sediment	16S-rDNA sequencing	Off Bainbridge Island, Puget Sound, USA	(*333*)
Marine shelf sediment	16S-rRNA, probe hybridization	Santa Rosa Sound, Florida, USA	(*334*)
Marine shelf sediment	16S-rRNA, probe hybridization	Santa Rosa Sound, Florida, USA	(*335*)
Hypersaline pond	16S-rRNA, probe hybridization	Exportadora De Sal, Baja California Sur, Mexico	(*336*)
Marine shelf sediment	SSU-rRNA sequencing	New England, USA	(*337*)
Marine deep sea/shelf sediment	16S-rRNA, *dsr*B sequencing	Skagerrak; Lillebælt, Baltic Sea	(*338*)
HM polluted shelf sediment	metagenomic shotgun sequencing	Centre Island; Che Lei Pai; Port Island; Tung Ping Chau, Tolo Harbour, China	(*339*)
Manrgove sediment	16S-rRNA, metagenomic sequencing	Mangroves at Yunxiao, China	(*340*)
Marine shelf sediment	16S-rRNA sequencing	Van Keulenfjorden, Svalbard	(*341*)
Marine lake sediment	16S-rRNA, FISH	Scharendijke Basin, marine Lake Grevelingen southwestern part of the Netherlands	(*342*)

Table S2 continued

Sample type	Method	Sample location	Reference
Intertidal flat	16S-rRNA, Illumina MiSeq	Eastern Beibu Gulf, China	(*343*)
Lake sediment	*dsr*AB, DGGE	Ghantasila Island, Chilika Lake, India	(*344*)
Hydrocarbon seep/deep sea sediment	metagenomic shotgun sequencing, 16S-rRNA, CARD-FISH	Green Canyon, Gulf of Mexico	(*345*)
Marine arctic deep sea sediment	16S-rRNA, FISH	Storfjordrenna, Svalbard	(*23*)
Deep sea sediment	16S-rRNA, metagenomic shotgun sequencing, CARD-FISH	Håkon Mosby mud Volcano, Norwegian-Barents-Spitzbergen Continental Margin	(*346*)
Marine shelf sediment	16S-rRNA, RFLP, Metagenomic shotgun sequencing	Northern Gulf of Mexico, near the Mississippi Delta	(*347*)
Deagras rhizosphere, shelf sediment	16S-rRNA sequencing	Off shore of Hobie Island, Florida, USA	(*348*)
Deep sea sediment	16S-rRNA sequencing	southwest of Oki Trough, Sea of Japan	(*349*)

Table S3 Biogeography and habitats of phylotypes affiliating with the genus *Desulfobacula.*

Sample type	Method	Sample location	Reference
Lake water/lake sediment	*hgc*A, 16S-rRNA, metagenomic analysis	lake manganika; lake McQuade, USA	(*350*)
Oily deep sea sediment	16S rRNA and metagenomic sequencing	Chapopote; Campeche Knolls in the Gulf of Mexico	(*351*)
Deep sea sediment	16S-rRNA sequencing	Kryos Basin, Eastern Mediterranean Sea	(*352*)
Deep sea wood fall	16S-rRNA, ARISA	Central Province, Eastern Mediterranean Sea	(*353*)
Intertidal sediment	16S-rRNA sequencing	Janssand, German Wadden Sea	(*354*)
Marine shelf sediment	16S-rRNA, MasD, CARD-FISH	Caspian Sea	(*355*)
Methane seep/deep sea sediment	16S-rRNA, FISH-nanoSIMS	Eel River Basin, southern Ridge	(*159*)
Marine shelf sediment	16S-rRNA sequencing	Thuwal Seeps, Saudi Arabia	(*356*)
Marine water column	*dsr*B, DGGE	Gdansk Bay,Baltic Sea	(*174*)
Marine shelf sediment	16S-rRNA, *ass*A, *bss*A sequencing	Chesapeake Bay, Virginia, USA	(*357*)
Deep sea sediment/microbial mat	IPL, 16S-rRNA sequencing	Japan Trench	(*358*)
Saltmarsh	16S-rRNA sequencing	Magnolia Marsh, Geogia, USA	(*190*)
Deep groundwater	16S-rDNA pyrosequencing	ONK-PVA6, ONKALO, Finland	(*359*)
Hydrocarbon seep/deep sea sediment	TEFAP 454 pyrosequencing	Acre location, Mediterranean Sea	(*192*)
Oil polluted marine shelf sediment	16S-rRNA, Genome sequencing	Taean, Republic of Korea	(*360*)
Intertidal sediment	*dsr*A, 16S-rRNA sequencing	Adour estuary, French Coast	(*361*)
Hydrocarbon seep/deep sea sediment	16S-rRNA, FISH-nanoSIMS	Hydrate Ridge, Gulf of Mexico	(*201*)
Deep sea sediment	16S-rRNA, DGGE, CARD-FISH	Continental Slope off Namibia	(*362*)
Estuarine sediment	16S-rRNA, *dsr*AB, DGGE	Colne Estuary, Essex, UK	(*202*)
Sulfidic spring	16S-rRNA , FISH, SR-FTIR	Mühlbacher Schwefelquelle, Germany	(*363*)
Cold seep/deep sea sediment	16S-rRNA, ARISA, CARD-FISH	Omakere Ridge; Wairarapa Takahae, Hikurangi Continental Margin, New Zealand	(*205*)
Marine shelf sediment	16S-rRNA, T-RFLP	Gulf of Finland, Baltic Sea	(*364*)
Contaminated marine shelf sediment	*bam*A, FISH	Sanban-ze, Tokyo Bay, Japan	(*114*)
Marine shelf sediment	16S-rRNA, T-RFLP	Western Gulf of Finland	(*365*)
Coal oil point seep	454 sequencing, *dsr*AB	Tonya Seep, California, USA	(*224*)
Aquifer	16S-rRNA, T-RFLP	Testfeld Süd, Germany	(*366*)
Aquifer	16S-rRNA, ARDRA, T-RFLP	Saxony-Anhalt, Germany	(*367*)
Cold seep/deep sea sediment	16S-rRNA, FISH	Håkon Mosby mud Volcano, Norwegian-Barents-Spitzbergen Continental Margin	(*261*)
Meromictic Lake	16S-rRNA, probe hybridization	Lake Cadagno, Switzerland	(*368*)
Deep sea sediment	16S-rDNA sequencing	Antarctic Continental shelf Area, Mertz Glacier Polynya	(*310*)
Deep sea sediment	16S-rRNA, *dsr*AB sequencing	Everest Mound, Guaymas basin, Gulf of California, Maxico	(*369*)
Hypersaline pond	16S-rDNA, RFLP, dot-blot-hybridization	Etang de Faraman, Salin-de-Giraud salterns, France	(*370*)
Marine shelf sediment	16S-rRNA, DNA-DNA-hybridization	Shallow Bay, Signy Island, Antarctica	(309)
Marine shelf sediment	16S-rRNA sequencing	Hornsund, Svalbard Arctic Ocean	(323)
Deep sea sediment	16S-rRNA sequencing	Guaymas Basin, Gulf of California, Mexico	(331)

Table S4 Biogeography and habitats of phylotypes affiliating with the genus *Desulfobacterium.*

Sample type	Method	Sample location	Reference
Mangrove sediment	16S-rRNA, metagenomic sequencing	Yunxiao, China	(*340*)
Perenially frozen lake	16S-rRNA sequncing	Tarn Flat Area, Northern Victoria Land	(*371*)
Marine shelf sediment	SSU-rRNA, RFLP	Northern Gulf of Mexico	(*347*)
Marine shelf sediment	16S-rRNA, dsrB, DGGE	Chersonesus (Blue) Bay, Black Sea	(*372*)
Hypersaline pond	Metagenomic sequencing	Lagunita, Churince lake, Mexico	(*373*)
Eutrophic lake	16S-rRNA, DGGE	Lake Vechten, The Netherlands	(*374*)
Deep sea sediment	16S-rRNA, CARD-FISH-raman	Smeerenburgfjorden, Svalbard	(*375*)
Marine shelf sediment	16S-rRNA sequencing	San Francisco bay, California, USA	(*376*)
Hydrothermal sediment	16S-rRNA, DGGE	Bahía de Banderas, Nayarit, Mexico	(*377*)
Oil field	16S-rRNA, *dsr*B, ARDRA	Xinjiang Oil Field, Dongying, China	(*378*)
Deep sea hydrothermal field	16S-rRNA, dsr (amino sequence) sequencing, metagenomic analysis	Pacmanus and Desmos hydrothermal fields, Manus Basin, Papua New Guinea	(*379*)
Organically/HM polluted shelf sediment	16S-rRNA sequencing	Sfax city, Tunisia	(*380*)
Saline enclosed reservoir	16S-rRNA sequencing, PLFA-SIP	Lake Grevelingen, The Netherlands	(*150*)
Methan seep/deep sea sediment	16S-rRNA, FISH-nanoSIMS	Eel River Basin, California, USA	(*159*)
Deep sea hydrothermal field	16S-rRNA pyrosequencing	Mat Mound, Guaymas Basin, Gulf of California	(*381*)
Deep sea sediment	16S-rRNA pyrosequencing	Skagerrak, Denmark	(*163*)
Deep sea hydrothermal field	16S-rRNA, CARD-FISH	Guaymas Basin, Gulf of California, Mexico	(*167*)
Deep sea hydrothermal field	16S-rRNA sequencing	Bastille Chimney, Juan de Fuca Ridge	(*382*)
Deep sea sediment	16S-rRNA, BONCAT, FISH, CARD-FISH	Santa Monica Basin, California; Hydrate Ridge, Oregon, USA	(*160*)
Methan seep/deep sea sediment	16S-rRNA, T-RFLP	Hydrate Ridge, Cascadia convergent Margin; Eel River Basin, California, USA	(*383*)
Intertidal sediment	*dsr*AB, T-RFLP	AberBenoît, Treglonoua Basin, France	(*384*)
Marine shelf sediment	16S-rRNA, DGGE	Eckernförde Bay, Germany	(*385*)
Deep sea sediment/microbial mat	IPL, 16S-rRNA sequencing	Japan Trench	(*358*)
Saltmarsh	16S-rRNA sequencing	Magnolia/Talbert marsh, California, USA	(*190*)
Deep sea sediment	16S-rRNA, SIP, CARD-FISH	Amon Mud Volcano, Nile deep Sea Fan	(*197*)
Paddy soil	*dsr*AB, T-RFLP	Wanshan Hg mining Area, China	(*386*)
Deep groundwater	16S-rDNA pyrosequencing	ONK-PVA6, ONKALO, Finland	(*359*)
Wetland	16S-rRNA sequencing	Luoshijiang Wetland, China	(*387*)
Lake sediment	*dsr*AB, *apr*A sequencing	Subglacial Lake Whillans, Antarktika	(*388*)
Deep sea hydrothermal field	16S-rRNA, dsrAB, FISH	Mat Mound, Guaymas Basin, Gulf of California	(*389*)
Estuarine sediment	16S-rRNA, *dsr*AB, DGGE	Colne estuary, Essex, UK	(*202*)
Marine shelf sediment	16S-rRNA, T-RFLP	Gulf of Finland, Baltic Sea	(*364*)
Intertidal sediment	16S-rRNA sequencing	Coast of the Genkai Sea, Japan	(*213*)
Lake water and sediment	*apr*A, 16S-rRNA sequencing	Lake Pavin, France	(*390*)
Deep sea sediment	*apr*A and *dsr*A sequencing	Peru Margin; Black sea	(*230*)
Coal oil point seep	454 sequencing, *dsr*AB	Tonya Seep, California, USA	(*224*)
Marine shelf sediment	*apr*A, DGGE	Tirez lagoon, Iberian region of La Mancha	(*391*)
Marine shelf sediment	16S-rRNA, *dsr*AB, RFLP	Nea Kameni Island, Santorini, Greece	(*231*)
Aquifer	16S-rRNA, T-RFLP	Testfeld Süd, Germany	(*366*)
Aquifer	16S-rRNA, T-RFLP	Testfeld Süd, Germany	(*392*)
Cold seep/deep sea sediment	16S-rRNA sequencing	Larsen B, western Weddell, Antarctika	(*240*)
Aquifer	16S-rRNA, DGGE	Mikkeli, Finland	(*393*)
PCB-enriched marine shelf sediment	16S-rRNA, *dsr*AB sequencing	Boston Harbor, Massachusetts, USA	(*245*)
Contaminated marine shelf sediment	16S-rDNA, DGGE	Paletta Creek, San Diego Bay, USA	(*258*)
Deep sea sediment	16S-rRNA, DGGE	Benguela upwelling System, Namibia	(*265*)
Deep-sea hypersaline anoxic lake	16S-crDNA sequencing	Lake Urania, Eastern Mediterranean	(*394*)
Meromictic lake	16S-rRNA, FISH	Gel Lake, Azerbaijan	(*276*)
Meromictic lake	16S-rRNA, DGGE	Lake Kaiike, Kamikoshiki Island, Japan	(*395*)
Meromictic lake	16S-rRNA, probe hybridization	Lake Cadagno, Switzerland	(*368*)
Marine shelf sediment	16S-rDNA, RFLP	Thermaikos Gulf, Eastern mediterranen Sea	(*396*)
Saltmarsh and estuarine/limnic sediment	16S-rRNA, probe hybridization	Colne estuary, Essex, UK	(*397*)
Deep sea sediment	16S-rRNA, dsrAB sequencing	Everest Mound, Guaymas basin, Gulf of California, Maxico	(*369*)
Lake sediment	SSU-rRNA sequencing	Lake Biwa, Japan	(*308*)

Table S4 continued

Sample type	Method	Sample location	Reference
Estuarine sediment	16S-rRNA probe hybridization	Colne estuary, Essex, UK	(*398*)
Estuarine sediment	16S-rRNA probe hybridization	Colne estuary, Essex, UK	(*399*)
River sediment	16S-rRNA probe hybridization	Tama river, Tokyo, Japan	(*400*)
Saltmarsh	16S-rRNA probe hybridization	Skidaway River, Savannah, USA	(*324*)
Marine shelf sediment	16S-rRNA, slot-blot hybridization	Smeerenburgfjorden, Svalbard, Antactica	(*323*)
Saltmarsh	16S-rDNA, dot-blot hybridization	Canary Creek Marsh, Delaware, USA	(*401*)
Meromictic lake	16S-rRNA, probe-hybrdization	Lake Kizaki, Japan	(*402*)
Cold seep/deep sea sediment	16S-rDNA, RFLP	Japan Trench	(*403*)
Deep sea sediment	16S-rRNA, RFLP	Guaymas Basin, Gulf of California, Mexico	(*331*)
Fjord water	16S-rRNA, in-situ hybridization	Mariager Fjord, Denmark	(*404*)

Table S5 Biogeography and habitats of phylotypes affiliating with the genus *Desulfonema*.

Sample type	Method	Sample location	Reference
Lake sediment	16S-rRNA, metagenomic sequencing	Cock Soda Lake, Altai, Russia	(*405*)
Marine shelf sediment	16S-rDNA sequencing	Salines de la Trinitat, Tarragona, Spain	(*406*)
Microbial mat (Lake)	16S-rRNA, metagenomic sequencing	Lake Huron, Michigan, USA	(*407*)
Methane seep/deep sea sediment	16S-rRNA, T-RFLP	Eel River Basin, California, USA	(*383*)
Deep sea hydrothermal vent system	SSU-rRNA, 454 tag sequencing	Main Endeavour Field, Juan de Fuca Ridge	(*408*)
Estuarine sediment	16S-rRNA, dsrAB, DGGE	Colne estuary, Essex, UK	(*202*)
Marine shelf sediment	16S-rRNA, T-RFLP	Aarhus Bay, Denmark	(*409*)
River sediment	16S-rRNA sequencing	Clinch River, Oak ridge, Tenessee, USA	(*410*)
Microflorae of moss pillars	16S-rRNA sequencing	Hotoke-Ike Lake, Skarvsnes, Antarktica	(*411*)
Cold mineral spring	16S-rRNA sequencing	Ust' Kachka Resort, Russia	(*219*)
Deep sea sediment	16S-rRNA, DGGE, ARDRA	Northern Bering Sea	(*412*)
Microbial mat (Cave)	16S-rRNA, FISH, RFLP	Lower Kane Cave, USA	(*413*)
Marine macrobial mat	16S-rRNA, FISH	Chilean Continental shelf	(*414*)
Microbial mat (hypersaline pond)	16S-rRNA, CARD-FISH, NanoSIMS	Exportadora De Sal, Baja California Sur, Mexico	(*257*)
Coastal saline pond	16S-rRNA, T-RFLP	saltern of Salins-de-Giraud, Camargue, France	(*415*)
Methane seep/deep sea sediment	16S-rRNA, Magneto-FISH, CARD-FISH	Eel River Basin, California, USA	(*416*)
Lake sediment	16S-rRNA, FISH	Lake Aha, China	(*256*)
Biofim (Cave)	16S-rRNA, FISH	Grotta Sulfurea, Italy	(*417*)
Deep sea sediment	16S-rDNA, RFLP	Florida Escarpment, Gulf of Mexico	(*418*)
Floodplain lake	16S-rRNA, dot-blot-hybridization	La Granja, Beni River Subbasin, Bolivia	(*295*)
Meromictic lake	16S-rRNA, DGGE	Lake Kaiike, Kamikoshiki Island, Japan	(*395*)
Intertidal sediment	16S-rRNA, DGGE, FISH	Jadebusen, Dangast, Germany	(*15*)
Cyanobacterial mat/ hypersaline lake sediment	16S-rRNA, *dsr*AB, probe hybridization	Solar Lake, Sinai, Egypt	(*314*)
Flooded rice roots/sediment	16S-rRNA, T-RFLP	Rice Research Institute, Vercelli, Italy	
Marine shelf sediment	16S-rDNA sequencing	Bassin d'Arcachon, South-West France	(*322*)
Marine shelf sediment	16S-rRNA, FISH, slot-blot hybridization	Smeerenburgfjorden, Svalbard, Antactica	(*323*)
Cyanobacterial mat/ hypersaline lake	16S-rRNA, probe hybridization	Solar Lake, Sinai, Egypt	(*419*)
PCB polluted shelf sediment	16S-rRNA, RFLP	Baltimore Harbor, Maryland, USA	(*329*)
Intertidal sediment	16S-rRNA, FISH	Dangast, Germany	(*420*)
Cyanobacterial mat/ hypersaline lake	16S-rRNA, DGGE	Solar Lake, Sinai, Egypt	(*421*)

Table S6: Properties of genome-sequenced members of the *Desulfobacteraceae*. Only completely genome-sequenced members are shown. Strains selected for proteogenomic analysis in our laboratory are highlighted.

Property	*Desulfosarcina variabilis* 3be13	*Desulfosarcina alkanivorans* PL12	*Desulfosarcina ovata* 28bB2T	*Desulfosarcina ovata* oXyS1	*Desulfosarcina widdellii* PP31	*Desulfonema magnum*	*Desulfonema limicola*	*Desulfococcus multivorans* 1be1	*Desulfococcus oleovorans* Hxd3	*Desulfatibacillum alkenivorans* AK-01	*Desulfobacula toluolica* Tol2	*Desulfobacterium autotrophicum* HRM2
**PHYSIOLOGICAL CHARACTERISTICS**												
**Organic substrates**												
Aliphatic hydrocarbons	–	C_6, C_{10}	–	–	–	–	–	–	C_{12} – C_{20}	C_{13} – C_{18}	–	–
Aromatic hydrocarbons	–	–	1	3	1	–	–	–	–	–	1	–
Fatty acids	$\leq C_{14}$[a,b]	n.d.	n.d.	$<C_4$	$<C_4$	$\leq C_{10}$[a]	$\leq C_{14}$[a]	$<C_{14}$[a,b]	C_4 – C_{18}	$<C_{16}$	$<C_4$	$<C_{16}$[a]
Other aliphatic compounds	8	8	n.d.	8	7	5	4	6	–	3[c]	8	8
Other aromatic compounds	13	–	n.d.	4	1	5	–	7	–	–	7	–
**Electron acceptors**												
Sulfate	+	+	+	+	+	+	+	+	+	+	+	+
Sulfite	+	+	n.d.	n.d.	–	–	+	+	+	+	+	–
Thiosulfate	+	+	n.d.	n.d.	+	–	+	+	+	+	+	+
**Autotrophy**	+	+	n.d.	n.d.	+	–	+	–	–	+	–	+
**Syntrophy**	–	n.d.	n.d.	n.d.	n.d.	n.d.	n.d.	–	n.d.	+	n.d.	n.d.
**Morphology**	oval, sarcina	oval, sarcina	oval, sarcina	oval, sarcina	oval, sarcina	filaments	filaments	spherical	spherical	rod-shaped	oval	oval
**Isolation habitat**	$-O_2$ marine sediment	$-O_2$ marine sediment	$-O_2$ marine sediment	Seawater in oil tank	$-O_2$ marine sediment	$-O_2$ marine sediment	$-O_2$ marine sediment	Sewage digester	Seawater in oil tank	$-O_2$ estuary sediment	$-O_2$ marine sediment	$-O_2$ marine sediment
**GENOME FEATURES**												
Size (bp)	9,639,276	7,324,708	8,413,530	7,630,248	7,297,718	8,027,777	6,908,045	4,455,399	3,944167	6,517,073	5,197,905	5,652,035
G+C content (mol %)	51.3	56.6	53.9	53,9	54.0	44.8	38.5	56.8	56.2	54.5	41.4	48.8
rRNA operons	2	6	6	6	6	1	2	3	1	2	4	6
tRNAs	58	50	52	55	54	68	61	54	47	56	48	50
Coding sequences (CDS)	8,579	6,234	6,493	6,493	6,518	9,970	6,207	3,942	3,267	5,296	4,375	4,947
Coding (%)[d]	86	86	86	86	86	87	90	87	88	87	87	88
Average CDS size (bp)[d]	960	954	971	973	923	700	1,001	986	1,066	1,080	1,031	1,017
Assigned function[d]	5,422	2,890	2,542	2,893	3,103	4,015	3,929	2,684	2,333	3,971	3,221	3,477
Conserved unknown[d]	1,423	3,344[e]	3,951[e]	3,600[e]	3,415[e]	1,121	1,173	663	405	381	654	551
Unknown[d]	1,724					4,834	1,105	595	529	944	500	873
Plasmids (size in kbp)	–	–	2 (136, 16)	–	–	–	–	–	–	–	–	1 (63)
Phage	1	n.d.	1[f]	2[f]	6[f]	2	1	–	–	–	1	1
CRISPR	3	1	4	4	–	20	8	6	2	1	3	1
Mobile elements[d]	147	71	148	109	81	351	703	45	18	79	163	98
Accession number	CP159846	AP021874	AP021876	AP021879	AP021875	CP061800	CP061799	CP015381	CP000859	CP001322	FO203503	CP001087
Reference(s)	This study, (68)	(120, 433)	(114)	(115)	(*114*)	(29, 68)	(29, 68)	(28)	(*68, 423*)	(*424, 425*)	(27, *125*)	(128, *129*)

[a] including branched-chain fatty acids, [b] including cyclohexane carboxylate, [c] only poorly utilized, [d] data extracted from respective Genbank files, [e] no differentiation between unknown/conserved unknown, [f] incomplete, n.d. not described

Table S7: Properties of selected, genome-sequenced sulfate reducing bacteria not belonging to the *Desulfobacteraceae*.

**Property**	H_2/CO_2	Aliphatic compounds	Aromatic compounds	Size (bp)	G+C content (mol %)	Coding sequences (CDS)	Mobile elements[d]	Isolation habitat	Accession	Reference
Dfm. tiedjei DSM6799	+	+	+	6,500,104	49.0	5,494	59	Sewage sludge	CP003360	(*25*)
Sb. fumaroxidans MPOB	+	+	+	4,990,251	59.0	4,064	64	Methanogenic granular sludge	CP000478	(*426*)
Dba. acetoxidans DSM11109	−	+	−	3,282,536	51.1	2,866	10	Anaerobic granular sludge	CP002629	(*427*)
Dbu. oralis HOT041	−	+	−	2,774,417	59,8	2,395	64	Oral subgingival	CP021255	(*428*)
Dca. sulfexigens DSM10523	+	−	−	4,023,512	45.4	2,794	26	Tidal flat sediment	CP003985	(*429*)
Dl. psychrophila LSv54	+	+	−	3,523,383	46.8	3,115	25	Marine sediment	CR52287	(*430*)
Gb. sulfurreducnes PCA	+	+	−	3,814,139	60.9	3,466	28	Ditch surface sediment	AE017180	(*431*)
Da. baarsii DSM2075	+	+	−	3,655,731	65.7	3,303	5	Ditch mud	CP002085	(*432*)
Dm. baculatum X	+	+	−	3,942,657	58.6	3,494	19	Water-saturated manganese carbonate ore	CP001629	(*433*)
Dh. retbaense HR100	+	+	−	2,909,567	57.3	2,552	25	Surface sediment	CP001734	(*434*)
Dv. gigas DSM1382	+	+	−	3,693,899	63.4	3,273	18	Water pond	CP006585	(*435*)
Dv. salexigens DSM2638	+	+	−	4.289.847	47.1	3,807	11	Sling mud	CP001649	
Dv. alaskensis G20	+	+	−	3,730,232	57.8	3,258	45	Oil well corrosion site	CP000112	(*436*)
Dv. vulgaris Hildenborough	+	+	−	3,570,858	63.2	3,395	28	Wealden clay	AE017285	(*437*)
Dv. desulfuricans MB	+	+	−	2,873,437	58.1	2,382	21	Rumen, sheep	CP001358	

Table S8: Relative share of protein abundance per KEGG pathway category of averaged peptide counts for total protein (TP) and constitutively formed proteins (CP) per *Desulfobacteraceae* member. Abbreviations: Dbt, *Desulfobacula toluolica* Tol2; Dcm, *Desulfococcus mutlivorans* 1be1; *Desulfonema limicola*; Dnm, *Desulfonema magnum*; Dsv, *Desulfosarcina variabilis* 3be13; Dta, *Desulfobacterium autotrophicum* HRM2.

	**Dbt**		**Dcm**		**Dnl**		**Dnm**		**Dsv**		**Dta**	
**KEGG Pathway**	TP	CP	TP	CP	TP	CP	TP	CP	TP	CP	TP	CP
1-01 Carbohydrate metabolism	16.6	16.0	16.7	15.0	20.7	20.0	20.0	16.9	19.1	16.3	16.7	16.1
1-02 Energy metabolism	25.3	26.2	35.9	41.4	42.3	43.6	35.9	38.0	32.2	38.8	31.9	33.7
1-03 Lipid metabolism	2.1	2.1	1.8	0.9	0.5	0.4	1.4	1.3	2.3	1.5	2.4	1.9
1-04 Nucleotide metabolism	2.3	2.3	1.8	1.7	2.7	2.5	4.1	4.6	3.0	3.5	2.4	2.5
1-05 Amino acid metabolism	12.4	12.8	8.3	5.8	5.0	4.8	8.7	8.7	9.4	7.7	8.2	7.4
1-06 Metabolism of other amino acids	1.5	1.7	0.9	0.8	2.7	2.9	1.9	2.1	1.4	1.6	1.7	1.8
1-07 Glycan biosynthesis and metabolism	0.1	0.0	0.7	0.6	0.3	0.3	0.2	0.2	0.2	0.1	0.2	0.2
1-08 Metabolism of cofactors and vitamins	4.4	4.3	3.4	3.5	5.4	5.7	5.7	6.1	5.1	5.8	3.8	4.0
1-09 Metabolism of terpenoids and polyketides	2.2	2.3	1.1	0.8	0.6	0.5	0.9	0.8	1.1	0.4	0.8	0.8
1-10 Biosynthesis of other secondary metabolites	3.8	4.0	0.9	0.8	2.5	2.7	1.7	1.9	1.3	1.2	1.3	1.4
1-11 Xenobiotics biodegradation and metabolism	9.6	9.5	5.0	3.5	3.5	3.5	6.9	7.0	7.1	5.5	5.1	4.6
1-12 Chemical structure transformation maps	0.0	0.0	0.0	0.0	0.0	0.0	0.0	0.0	0.0	0.0	0.0	0.0
2-01 Transcription	0.1	0.1	0.0	0.0	0.0	0.0	0.0	0.0	0.2	0.2	0.1	0.1
2-02- Translation	2.0	1.8	4.1	4.1	3.2	3.2	2.4	2.5	2.9	2.3	6.6	7.0
2-03 Folding sorting and degradation	3.6	3.8	4.2	4.8	4.1	4.3	2.4	2.7	4.7	5.6	6.7	7.2
2-04 Replication and repair	0.1	0.0	0.8	1.0	0.4	0.4	0.3	0.3	0.3	0.3	0.3	0.2
2-05 Chromosome	0.0	0.0	0.0	0.0	0.0	0.0	0.0	0.0	0.0	0.0	0.0	0.0
2-06 Information processing in viruses	0.0	0.0	0.0	0.0	0.0	0.0	0.0	0.0	0.0	0.0	0.0	0.0
3-01 Membrane transport	1.6	1.6	3.8	4.1	1.0	0.8	0.5	0.4	1.9	1.8	2.8	2.5
3-02 Signal transduction	5.6	5.0	2.6	2.4	1.9	1.6	3.0	2.8	3.5	3.2	3.6	3.5
3-03 Signaling molecules and interaction	0.0	0.0	0.0	0.0	0.0	0.0	0.0	0.0	0.0	0.0	0.0	0.0
4-01 Transport and catabolism	0.2	0.2	0.3	0.3	0.2	0.2	0.4	0.4	0.4	0.4	0.1	0.0
4-02 Cell growth and death	0.4	0.4	0.8	0.6	0.5	0.5	0.3	0.2	1.0	1.1	1.3	1.2
4-03 Cellular community-eukaryotes	0.0	0.0	0.0	0.0	0.0	0.0	0.0	0.0	0.0	0.0	0.0	0.0
4-04 Cellular community-prokaryote	3.7	4.0	5.6	6.4	1.9	1.6	2.5	2.2	2.7	2.4	2.9	2.8
4-05 Cell motility	2.5	2.0	1.1	1.4	0.6	0.5	1.0	0.9	0.4	0.3	1.1	1.1

Table S9: Number of transport systems (TS), Transporter proteins (TP) and identified transporter proteins (ID) per studied *Desulfobacteraceae* member as inferred from TCDB. Abbreviations: Dbt, *Desulfobacula toluolica* Tol2; Dcm, *Desulfococcus mutlivorans* 1be1; Dnl, *Desulfonema limicola*; Dnm, *Desulfonema magnum*; Dsv, *Desulfosarcina variabilis* 3be13; Dta, *Desulfobacterium autotrophicum* HRM2.

	Dbt			Dcm			Dnl			Dnm			Dsv			Dta		
**Subclass**	**TS**	**TP**	**ID**	**TS**	**TP**	**ID**	**TS**	**TP**	**ID**	**TS**	**TP**	**ID**	**TS**	**TP**	**ID**	**TS**	**TP**	**ID**
**1.A** Alpha-type channel	28	37	10	31	33	15	38	41	7	41	44	10	36	43	30	32	39	17
**1.B** Beta-barrel Porins	52	66	21	21	21	17	42	43	14	53	57	21	53	53	29	32	33	18
**1.C** Pore-forming toxins	2	2	1	3	3	1	3	3	2	2	2	1	4	5	2	4	4	1
**1.E** Holins	1	1	0	3	3	1	1	1	0	1	1	0	2	2	0	2	2	0
**1.P** Non-envelope Virus Penetration Complex	1	3	1	0	0	0	0	0	0	0	0	0	0	0	0	0	0	0
**2.A** Porters (uniporters, symporters, antiporters)	174	238	52	124	174	69	95	117	27	103	137	27	180	226	76	195	259	56
**2.C** Ion-gradient-driven energizers	4	10	3	3	9	8	2	5	2	1	7	2	5	8	6	2	5	3
**3.A** P-P-bond-hydrolysis-driven transporters	71	205	50	80	216	116	56	168	77	57	170	48	115	373	163	113	343	93
**3.B** Decarboxylation-driven transporters	1	2	2	0	0	0	1	4	3	1	3	3	1	3	0	2	6	3
**3.D** Oxidoreduction-driven transporters	12	63	24	9	53	34	8	42	33	6	30	14	15	86	56	12	47	25
**4.A** Phosphotransfer-driven Group Translocators (PTS)	0	0	0	0	0	0	0	0	0	0	0	0	0	0	0	2	2	9
**4.C** Acyl-CoA ligase-coupled transporters	25	25	2	28	28	1	22	22	2	31	31	2	4	4	2	27	27	4
**4.D** Polysaccharide synthase/exporters	0	0	0	5	5	0	4	4	0	6	6	0	8	8	4	6	6	0
**4.F** Choline/Ethanolamine Phosphotransferase	2	2	0	3	3	2	2	2	0	3	3	1	2	2	0	1	1	1
**4.H** Lysylphosphatidylglycerol Synthase/Flippases	0	0	0	0	0	0	0	0	0	0	0	0	0	0	0	1	1	0
**5.A** Transmembrane 2-electron transfer carriers	11	30	7	7	17	9	5	10	4	2	2	4	9	18	7	7	15	5
**5.B** Transmembrane 1-electron transfer carriers	6	19	9	3	21	14	4	18	12	3	7	5	5	12	10	8	16	8
**8.A** Auxiliary transport proteins	31	36	17	20	22	8	26	26	3	32	34	6	26	27	14	27	30	19
**8.B** Ribosomally synthesized toxins/agonists that target channels and carriers	1	1	0	0	0	0	0	0	0	1	1	0	0	0	0	1	1	1
**9.A** Transporters of unknown biochemical mechanisms	11	16	8	3	4	5	6	7	5	7	9	4	11	17	12	9	11	6
**9.B** Putative transport proteins	106	107	21	48	48	23	69	71	18	69	77	10	89	92	34	65	67	19
Total	539	863	228	391	660	323	384	584	209	419	621	158	565	979	445	548	915	288

Table S10 Diversity profiles extracted from the MAGs of the studied sites. The ten most abundant orders are displayed.

Sample Location	Order	Rank	No. detections	Median % contribution
**Benguela upwelling**				
	Totals	0	6,340	0.0
	Unknown	1	1,730	26.1
	BSN033	2	816	13.0
	Betaproteobacteriales	3	746	4.1
	Aerophobales	4	433	6.2
	**Desulfobacterales**	**5**	**350**	**4.9**
	UBA10834	6	273	3.0
	B26-1	7	237	2.3
	DHVEG-1	8	231	2.1
	Marinisomatales	9	231	3.6
	UBA7937	10	218	2.8
**Black Sea**				
	Totals	0	12,972	0.0
	Unknown	1	2,247	16.4
	Aminicenantales	2	1,380	11.2
	C00003060	3	1,018	7.5
	SM23-42	4	643	4.5
	UBA10834	5	619	4.4
	GIF9	6	572	4.2
	Pirellulales	7	566	4.1
	**Desulfobacterales**	**8**	**549**	**4.5**
	Anaerolineales	9	501	3.3
	UBA7967	10	499	2.9
**Guaymas Basin**				
	Totals	0	163,948	0.0
	Unknown	1	141,807	86.5
	**Desulfobacterales**	**2**	**6,097**	**3.9**
	Methanosarcinales	3	2,885	1.5
	Archaeoglobales	4	2,627	1.6
	Anaerolineales	5	1,886	1.1
	Candidatus Altarchaeales	6	1,693	0.6
	Thermoplasmatales	7	876	0.3
	Candidatus Cloacimonadales	8	852	0.4
	Thermotogales	9	819	0.5
	Desulfurococcales	10	787	0.3
**Gulf of Kutch**				
	Totals	0	56,819	0.0
	Unknown	1	48,312	85.7
	Nitrospinales	2	2,327	2.5
	Pseudomonadales	3	1,397	1.8
	Desulfuromonadales	4	1,207	2.2
	Balneolales	5	960	1.5
	**Desulfobacterales**	**6**	**908**	**1.6**
	Xanthomonadales	7	692	1.2
	Nitrosopumilales	8	475	0.1
	Woeseiales	9	281	0.5
	Verrucomicrobiales	10	260	0.3
	Bin61	10	323	5.3

Table S10 Continued

Sample Location	Order	Rank	No. detections	Median % contribution
**Pacific Ocean off the coast of California**				
	Totals	0	5,777	0.0
	UBA5794	1	924	14.3
	**Desulfobacterales**	**2**	**628**	**10.2**
	Polyangiales	3	509	7.7
	Ectothiorhodospirales	4	459	8.1
	Rhizobiales	5	457	6.4
	Kiloniellales	6	395	6.3
	Pseudomonadales	7	387	3.1
	HK1	8	372	5.7
	BM002	9	347	5.2
**South China Sea**				
	Totals	0	185,496	0.0
	Unknown	1	117,656	61.4
	**Desulfobacterales**	**2**	**12,013**	**6.4**
	Bacteroidales	3	8,630	4.5
	Anaerolineales	4	5,476	2.8
	Desulfobulbales	5	3,859	2.2
	Methylococcales	6	2,664	1.6
	Spirochaetales	7	2,613	1.2
	Thermoplasmatales	8	2,602	1.1
	Thiotrichales	9	2,443	1.5
	Methanosarcinales	10	2,040	0.9
**Sumatra upwelling**				
	Totals	0	5,292	0.0
	Unknown	1	1,285	25.1
	**Desulfobacterales**	**2**	**1,067**	**20.0**
	C00003060	3	572	11.0
	UBA2258	4	484	8.2
	Betaproteobacteriales	5	408	3.0
	Rhizobiales	6	391	2.8
	Aminicenantales	7	381	6.6
	Aerophobales	8	180	2.4
	SG8-4	9	143	2.3
	Dehalococcoidales	10	116	0.6
**Challenger Deep**				
	Totals	0	101,997	0.0
	Unknown	1	99,227	97.8
	Myxococcales	2	2,770	2.2

Supplementary References

Supplementary material references are provided in a separate Excel file: "Auxiliary Reference List.xlsx".

Legends for data S1 and S2

Data S1: Constitutive proteins of the six proteomically studied Desulfobacteraceae members.
Data S2: Compiled results of the metagenomics analyses.
